# Hidden Diradical: Conformational Switch for Solvatochromic NIR Emission With Unity Quantum Yield in Thiele's Hydrocarbon

**DOI:** 10.1002/anie.202524042

**Published:** 2026-01-28

**Authors:** Matteo Bevilacqua, Mattia Reato, Federico Cilento, Claudia Graiff, Sabrina Antonello, Luca Schio, Alessandro Aliprandi, Cristina Tubaro, Lorenzo Franco, Martina Dell'Angela, Dominik Munz, Marco Baron

**Affiliations:** ^1^ Dipartimento di Scienze Chimiche Università degli Studi di Padova Padova Italy; ^2^ Coordination Chemistry Saarland University Saarbrücken Germany; ^3^ Elettra‐Sincrotrone Trieste S.C.p.A Basovizza Italy; ^4^ Dipartimento di Scienze Chimiche, della Vita e della Sostenibilità Ambientale Università di Parma Parma Italy; ^5^ CNR – Istituto Officina dei Materiali (IOM) Basovizza, Trieste Italy

**Keywords:** diradicals, luminescence, NIR emitter, organic electronic materials, sudden polarization

## Abstract

We present that halogen‐substituted and air‐stable Thiele‐type diradicaloids with folded (**1**) and planar (**2**, **3**) structures can be obtained by switching the positions of fluorine and chlorine substituents. The strongly distorted *p*‐quinodimethane **1** demonstrates emission in the UV–visible/near‐infrared (UV–vis/NIR) range with a photoluminescence quantum yield (PLQY) of 100%, an exceedingly large Stokes shift of up to 2.0 eV, an excited state lifetime of 81 ns, and pronounced solvatochromic emission (from 1.81 eV in *n*‐pentane to approximately 2.0 eV in dichloromethane). Dual emission in pentane, transient absorption spectroscopy, quantum chemical calculations, as well as the comparison with compounds **2** and **3** reveal that the exceptional photophysical properties of closed‐shell **1** are thanks to interconversion with its planar conformer **1^flat^**. The key here is that the conformer **1^flat^**, which is generated upon photoexcitation, excels with a pronounced singlet diradical character (*y*_0_ = 0.78) and a dark doubly excited (DE) S1 state. Our findings delineate how to leverage conformational equilibria to design bright luminescent materials based on hidden and thus air‐stable organic diradicals.

## Graphical abstract

Halogen substitution controls a conformational switch between a folded closed‐shell and a flat singlet‐diradical conformer in an air‐stable Thiele‐diradical. This conformational switch allows for near‐unity photoluminescence quantum yield with a large Stokes shift, solvatochromism, and a long‐lived excited state. We delineate how to harness the peculiar photophysical properties of usually air‐sensitive diradicals by air‐stable proxy compounds.

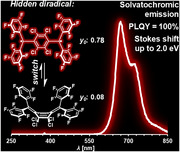

**Abbreviations**
DCM: dichloromethane
DE: doubly excited state
EAS: evolution associated spectra
ET: electron transfer
Et_2_O: diethylether
PLQY: photoluminescence quantum yield
PMMA: polymethyl methacrylate
*p*‐QDM: *para*‐quinodimethane
SCXRD: Single Crystal X‐Ray Diffractometry
SE: singly excited state
TAS: Transient Absorption Spectroscopy
TC: Thiele's hydrocarbon

## 1 Introduction

Diradicals, characterized by their two unpaired electrons [1, 2, 3, 4, 5, 6], fascinate not only with their versatile electronic structure, but also with applications encompassing conductive organic materials [7, 8, 9, 10], semiconductors for organic field‐effect transistors (OFETs) [11, 12, 13, 14], and singlet fission for organic photovoltaics [15, 16, 17]. Recent years saw the advent of emissive monoradicals, that is, doublet emitters [18, 19, 20]. Yet, the luminescence of diradicals remains poorly understood [21, 22, 23, 24, 25, 26, 27, 28, 29, 30, 31]. Their optical, electronic, and magnetic properties seem to complement though common closed‐shell luminescent molecules and monoradicals, hence offering vast potential for novel technology, including quantum information technology [32, 33, 34, 35, 36, 37, 38, 39], organic light‐emitting diodes (OLEDs) [40, 41, 42], magnetoluminescence [43, 44, 45], as well as bioimaging and phototherapy [46, 47, 48].

Thiele's hydrocarbons (TCs) are Kekulé‐type diradicals based on *para*‐quinodimethane (*p*‐QDM) [49]. They typically exhibit rather moderate diradical character as illustrated by a diradical index [50] *y*_0_ = 0.3 for the parent and air‐sensitive Thiele hydrocarbon, tetraphenyl‐*p*‐quinodimethane [51, 52, 53, 54]. Efforts have been made in recent years to modify the structure and properties of Thiele's (and phenylene‐extended Chichibabin's) [55, 56] hydrocarbons to create compounds with increased stability and tuned properties. Strategies include the preparation of carbene‐derived analogues [57, 58, 59, 60, 61, 62, 63, 64, 65], the introduction of heteroatoms in the backbone [66, 67, 68, 69], incorporation of steric constraints using extended π‐systems [70, 71, 72, 73, 74, 75, 76], as well as halogenation [51, 52, 77].

**FIGURE 1:**
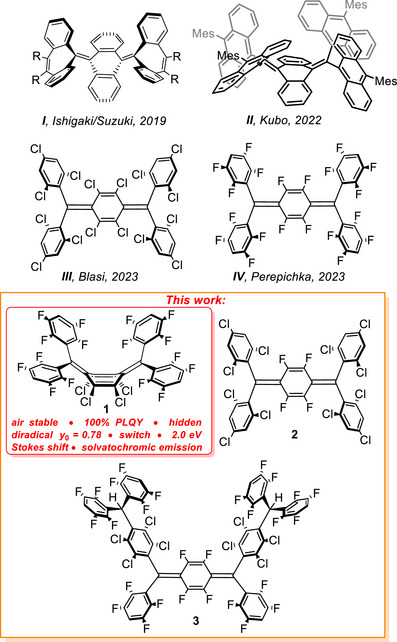
Thiele‐like diradicaloids reported by the Ishigaki/Suzuki (**I**), Kubo (**II**), Blasi (**III**), and Perepichka (**IV**) groups (top), and molecules studied herein (**1–3**, bottom).

Ishigaki/Suzuki and Kubo et al. reported strained Thiele‐like anthraquinodimethanes with sterically demanding dibenzocycloheptatrienyl (**I**, Figure 1) [76] and 9‐anthryl (**II**) [71] substituents, respectively. Both compounds showed folded molecular structures in the solid state that undergo a dynamic conformational change in solution. This conformational switch was used to develop electrochromic compounds [76] and singlet/triplet spin state control [71]. The compounds **I** and **II** are non‐luminescent, which was ascribed to non‐radiative decay due to the conformational freedom. The synthesis and characterization of perchlorinated Thiele's hydrocarbon was reported in 1991 by Castaner and Riera [77], who found that this compound behaves as a *p*‐quinoidal closed‐shell molecule of high thermal and chemical stability. More recently, Blasi et al. reported a partially chlorinated Thiele's derivative (**III**, Figure 1) that maintains thermal‐ and photostability and exhibits emission in the deep‐red/NIR region with a high photoluminescence quantum yield (PLQY) of up to 84% (in toluene), a Stokes shift of up to 0.9 eV in benzonitrile, and solvatochromism (from 0.7 eV in cyclohexane to 0.9 eV in benzonitrile) [51]. The authors attributed these observations to a zwitterionic excited state, which forms through a mechanism similar to sudden polarization occurring in olefins [78, 79, 80, 81]. In the case of common olefins, however [82, 83, 84], sudden polarization does not lead to luminescence due to competition of the slow radiative (*k*_r_) and non‐radiative rate constants (*k*_nr_) due to a conical intersection. Fluorinated Thiele's derivatives were reported by Perepichka et al. (**IV**, Figure 1). Also here, a considerable Stokes shift up to 1.5 eV (from 0.9 eV in cyclohexane to 1.5 eV in acetonitrile) was found, and the formation of a genuine diradical excited state of high polarizability was proposed in light of the positive solvatochromism [52]. This was attributed to the π‐system of the phenylene linker, which boosts the *k*_r_ value [52]. Both fluorinated and chlorinated Thiele's derivatives exhibit solvatochromic emission and large Stokes shifts, up to 1.5 and 0.9 eV in the case of **IV** and **III**, respectively [51, 52].

Inspired by the work by Blasi and Perepichka et al. and following our quantum chemical predictions [85], we were led to investigate the synthesis of mixed chloro‐fluoro‐Thiele's derivatives. Herein, we report the synthesis and characterization of three diradicaloids (**1–3**, Figure 1). Surprisingly, the structural and photophysical properties of **1** are markedly different from **2** and **3**. Compound **1** exhibits a folded *p*‐quinoidal ground state conformation [71, 72, 73, 74, 75, 76, 86, 87, 88, 89], previously unreported for Thiele's derivatives in the absence of steric constraints, along with dual photoemission in *n*‐pentane in the near‐infrared range with 100% quantum yield and a massive Stokes shift up to 2.0 eV.

## 2 Results and Discussion

### 2.1 Synthesis and Structural Analysis

**SCHEME 1:**
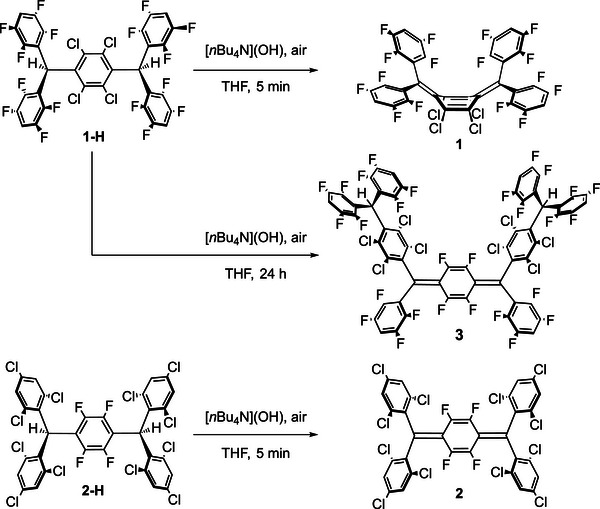
Synthesis of diradicaloids **1**, **2**, and **3**.

Compounds **1** and **2** were synthesized from precursors **1‐H** and **2‐H** through deprotonation with [*n*Bu_4_N](OH) and subsequent oxidation by air (Scheme 1). Compound **1** was isolated in modest yield (23%) due to side reactions occurring during the synthesis. Among the reaction byproducts, we isolated compound **3** in 8% yield, whose formation is further favored by longer reaction times (see Supporting Information). Compound **2** was isolated in a high yield of 87%.

**FIGURE 2:**
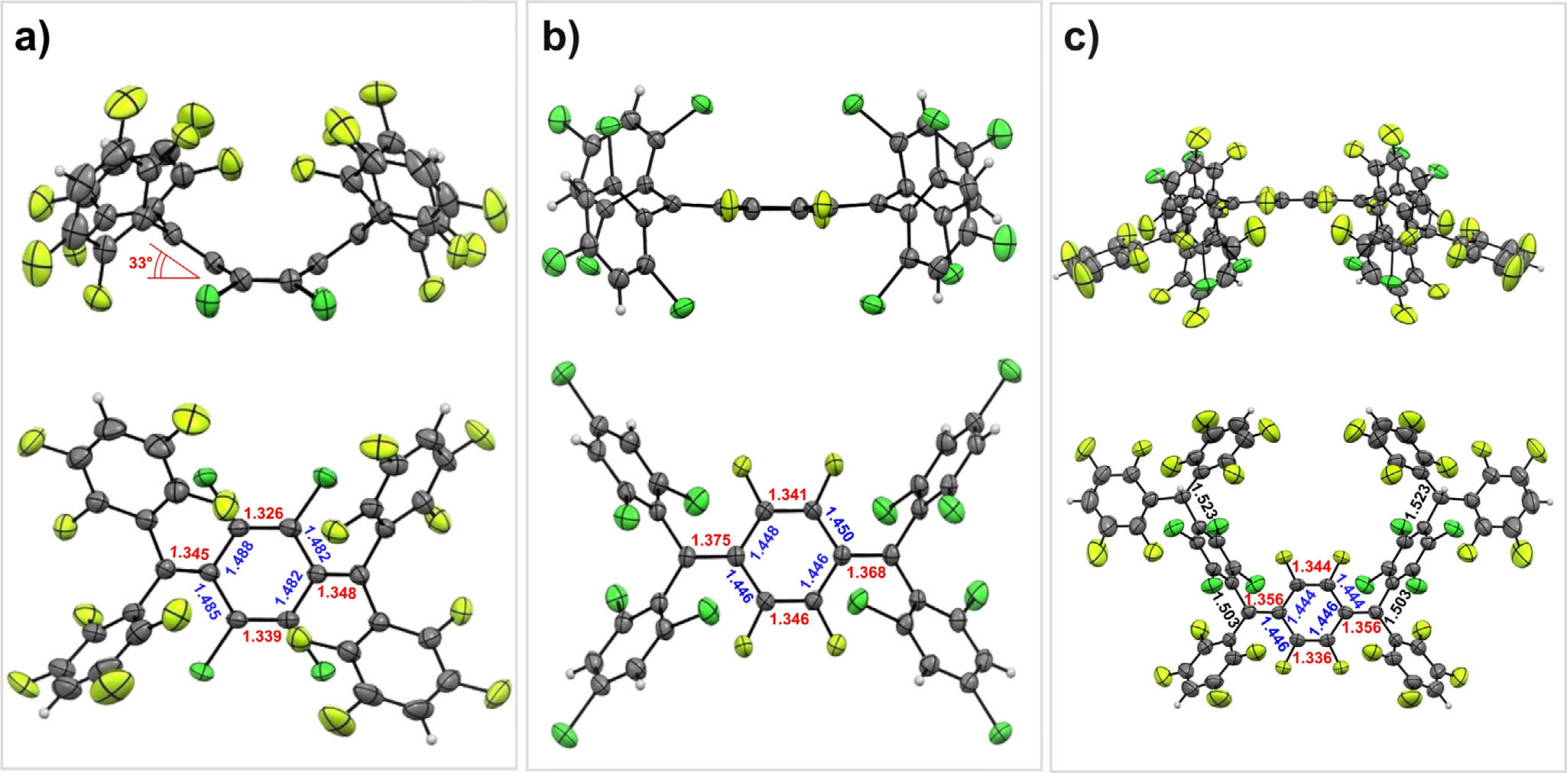
SCXRD structures of **1** (a), **2** (b), and **3** (c) with perpendicular and parallel views with respect to the central *p*‐QDM C_6_Cl_4_ (**1**) or C_6_F_4_ (**2** and **3**) bridge. Solvent molecules are omitted for clarity; thermal ellipsoids are displayed at 50% probability.

Single crystals of **1** and **2** suitable for SCXRD analysis were obtained through slow evaporation of their respective solutions in *n*‐pentane and *n*‐hexane. In the solid‐state structure of **1**, two crystallographically independent molecules are found. Compound **1** (Figure 2a) adopts a boat‐shaped conformation with corresponding dihedral angles of approximately 33° between the average plane defined by the four central chlorine‐substituted carbon atoms and the mean planes of the four‐atom units centered on the C(*sp* ^2^) carbon atom in positions 7 and 8, respectively, of the *p*‐QDM core (34.70° and 32.60°; Figures 2a and S67) [90] thus displaying a highly distorted *p*‐QDM structure. This dihedral angle is considerably larger than those (16°–18°) reported for strained Thiele's diradicaloids with other *p*‐tetrahalophenylene linkers (*p‐*C_6_F_4_, *p‐*C_6_F_2_Cl_2_, and *p‐*C_6_F_2_Br_2_) [70]. The short exocyclic (1.345(5) Å and 1.348(5) Å) C═C double bonds as well as those within the *p*‐C_6_Cl_4_ linker (1.326(5) and 1.339(5), red) in **1** are consistent with a quinoid electronic structure, and the same is true for the C─C single bonds (1.482(3)–1.488(4) Å; blue) adjacent to the diarylmethylene substituents. In stark contrast to the bent *p*‐QDM structure of **1**, compound **2** (Figure 2b) exhibits a flat conformation akin to conventional Thiele's hydrocarbons. The bond length alternation in the central *p*‐QDM bridge (1.341(3) and 1.346(5) Å; red; 1.446(3), 1.446(3), 1.448(3), and 1.450(3) Å, blue) is less pronounced and closer to that of **IV** rather than **III** (1.335, 1.335 Å **IV** red equivalent; 1.438–1.454 Å **IV** blue equivalent; 1.329, 1.354 Å **III** red equivalent; 1.429–1.455 Å **III** blue equivalent), the same is true for the exocyclic C═C bonds (1.375(3) and 1.368(3) Å, vs. 1.372 Å for **IV** and 1.401, 1.388 Å for **III**).

The NMR spectra of **1** (Figures S5–S8) and **2** (Figures S15–S18) are consistent with the quinoid structures in the solid state and provide further insights into their dynamic behavior in solution. The ^1^H NMR spectrum of compound **1** in CDCl_3_ exhibits a broad signal at 7.13 ppm (Figure S5), which is only slightly affected by temperature variations in the range of 243–303 K (Figure S8). Similar broad signals are also obtained in the ^19^F spectrum (Figure S6). This stands in contrast to **IV**, which exhibits well‐defined multiplets in both the ^1^H and ^19^F NMR spectra recorded in acetone‐d_6_. The signal broadening prevented determining the corresponding coupling constants and suggests the presence of a dynamic equilibrium for compound **1** in solution. Conformational equilibria between the *syn* and *anti p*‐QDM, as well as rotation of the exocyclic tetrafluorophenyl rings might occur as proposed by Kubo et al., who studied a “butterfly and screw flipping” equilibrium as well as the variation of spin‐state in folded π‑extended Thiele's hydrocarbons (**II**) by variable temperature (VT) NMR [71, 72]. The ^1^H NMR spectrum of **2** in CD_2_Cl_2_ at 298 K shows two broad signals at 7.44 and 7.34 ppm (Figure S15), similarly to those reported by Blasi et al. for **III**. In this case, increasing the temperature to 303 K results in a single broad signal centered at 7.37 ppm in C_2_D_2_Cl_4_ (Figure S17). The coalescence of two singlets is likely due to facile rotation of the exocyclic trichlorophenyl rings. Further, the temperature variation did not induce a substantial decrease in the signal intensity of the ^1^H spectra, indicating that the triplet spin states of compounds **1** and **2** are not readily accessible. In agreement, the compounds **1** and **2** proved EPR‐silent in toluene solution at room temperature and at −78°C (Figure S74).

Single crystals of **3** suitable for SCXRD were obtained by slowly evaporating an *n*‐hexane solution, and the structure in the solid state (Figure 2c) corroborated a planar *p*‐QDM core arrangement, consistent with the NMR, UV–vis, and emission data (vide infra). Compound **3** is derived from the coupling of two units of **1** and elimination of a 1,2,4,5‐tetrafluorobenzene molecule. The structure exhibits a planar *p*‐QDM C_6_F_4_ core with two different exocyclic substituents at each end, namely a tetrafluoroaryl and a halogenated trityl group, arranged in a *syn* configuration. The bond lengths in the central *p*‐QDM‐C_6_F_4_ core, highlighted in red and blue in Figure 2c, are similar to those in **2**, hence arguably indicating a comparable electronic structure.

### 2.2 UV–Vis/NIR Electronic Spectroscopies

**FIGURE 3:**
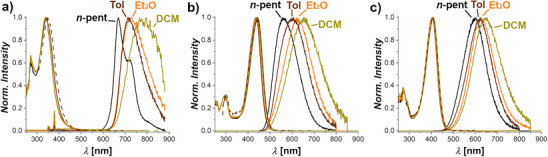
Normalized excitation (dashed lines), absorption, and emission (solid lines) spectra of **1** (a), **2** (b), and **3** (c) recorded in *n*‐pentane (*n*‐pent, black), toluene (Tol, brown), diethylether (Et_2_O, orange), and dichloromethane (DCM, green) recorded at room temperature.

UV–vis/NIR electronic absorption spectroscopy was employed to assess the optical properties of **1**, **2**, and **3** (Table 1; Figures 3 and S25–S34), with spectra recorded in *n*‐pentane, toluene, diethylether (Et_2_O), and dichloromethane (DCM). All three compounds exhibit only minor shifts in band position and intensity by changing the solvent. The spectra of **1** (Figures S26–S28) are characterized by an intense band centered at ≈340 nm, with a molar attenuation coefficient *ε* of ≈ 2.0 × 10^4^ M^−1^ cm^−1^. An additional weak band is observed at 271 nm, with *ε* ≈ 1.4 × 10^4^ M^−1^ cm^−1^. These band positions are reminiscent of those reported by Kubo et al. and Ishigaki/Suzuki et al. for their strained closed‐shell quinoidal molecules [70, 71, 72, 76]. Anthraquinone‐derived **II** (cf. Figure 1) shows two absorption peaks at 420/480 nm [71], the overcrowded tristricyclic aromatic ene reported by the same group has two absorption peaks at approximately 320/360 nm [72], and the strained hydrocarbons reported by Ishigaki/Suzuki et al. (**I** (cf. Figure 1)) feature two absorption peaks at 290/340 nm [76]. Diradicaloid **2** (Figures S30–S32) exhibits a band in the visible region centered at 440 nm, with *ε* ≈ 5.0 × 10^4^ M^−1^ cm^−1^. An additional band is present at 293 nm with *ε* ≈ 1.5 × 10^4^ M^−1^ cm^−1^. These characteristics align with those of **IV** reported by Perepichka and colleagues, thereby emphasizing the Thiele‐like nature of **2**. UV–vis and emission properties of **3** are similar to those observed for **2**. The UV–vis spectra (Figures S33, S34) show indeed peaks at 408 and 274 nm, with *ε* of ≈ 4.2 × 10^4^ M^−1^ cm^−1^ and 1.6 × 10^4^ M^−1^ cm^−1^, respectively, therefore presenting optical features rather similar to **2** than to folded *p*‐QDM‐based **1**. Structural analysis and literature examination indicate that the hypsochromic shift in absorption of compound **1** compared to compounds **2** and **3** (340 nm vs. 440 and 408 nm, respectively) can be primarily attributed to its folded molecular geometry. In conjugated π‐systems without charge separation, an increase in π‐conjugation typically results in a bathochromic shift of the spectrum (both in absorption and emission). This phenomenon is typically a consequence of a more planar conformation, which promotes a higher degree of π‐orbital overlap [91, 92, 93, 94, 95, 96].

**TABLE 1:** Main optical properties of **1–3** diradicaloids in different solvents.

Compound	Solvent	*λ*_abs_ (nm)	*ε* (M^−1^ cm^−1^)	*λ*_em_ (nm)	Δh*ν* (eV)	*τ* (ns) ± 1^a^	PLQY (%)
**1** ^b^	*n*‐Pentane *n*‐Pentane (77 K)	339 272	22 600 13 700	670 722 674^c^	1.81	81 17^d^	100
	Toluene	343 271	18 900 11 700	714	1.88	13	13
	Diethylether	339 272	22 400 14 000	727	1.95	5	3
	DCM	341 270	20 400 13 700	772^e^	ca. 2.0	–	<1
	Solid state (BaSO_4_)	–	–	685		–	–
	Solid state (10% PMMA)	–	–	680^f^		18^d^	–
**2** ^g^	*n*‐Pentane	436 293	53 000 16 200	560	0.63	14	44
	Toluene	443 293	47 700 13 300	600	0.73	9	88
	Diethylether	438 292	54 000 16 100	630	0.86	12	96
	DCM	441 293	52 900 16 400	650	0.90	10	43
	Solid state (BaSO_4_)	–	–	607		–	–
**3** ^h^	*n*‐Pentane	404 274	45 300 16 100	603	1.01	21	70
	Toluene	409	39 800	624	1.04	12	74
	Diethylether	404 274	45 600 15 900	628	1.10	13	63
	DCM	408 274	42 700 16 400	653	1.14	8	43
	Solid state (BaSO_4_)			600^i^		–	–

^a^ Lifetimes values were obtained from monoexponential decay fits.

^b^ *c* = 1.3 × 10^−5^ M for the absorbance measurements, 4.4 × 10^−6^ M for the emission measurements; *λ*_exc_ = 340 nm.

^c^ *λ*_exc_ = 375 nm.

^d^ Obtained from intensity weighted‐average lifetime for the measurements at 77 K and in PMMA film.

^e^ The maximum was estimated via a fitting with two Gaussians (Figure S35).

^f^ *λ*_exc_ = 410 nm.

^g^ *c* = 1.3 × 10^−5^ M for the absorbance measurements, 3.2 × 10^−6^ M for the emission measurements; *λ*_exc_ = 440 nm.

^h^ *c* = 1.0 × 10^−5^ M for the absorbance measurements, 2.4 × 10^−6^ M for the emission measurements; *λ*_exc_ = 400 nm.

^i^ *λ*_exc_ = 440 nm.

To assess the photo‐ and chemical stability of **1**, **2**, and **3** in solution, samples dissolved in aerated *n*‐pentane were irradiated for 8 h at 365 nm (Figures S28, S32, and S34). We did not observe notable changes in their UV–vis spectra, indicating that neither species undergoes decomposition and/or spin state or conformational transformations upon irradiation, which stands in contrast to other folded Thiele‐like diradicaloids reported in the literature [72, 97].

Further evidence for the distinct molecular structures of **1**, **2**, and **3** was obtained by their emissive properties (Table 1; Figures 3 and S35–S64). Excitation of a solution of **1** in *n*‐pentane at 340 nm results in two emission peaks centered at 670 and 722 nm, demonstrating a remarkable Stokes shift of 1.81 eV, with a unity PLQY of 100% and a decay lifetime of 81 ns (Figures S35 and S37). This represents a significant difference from planar Thiele‐like diradicaloids, which typically display a single broad emission peak, lower Stokes shifts (1.18 and 0.71 eV for **IV** and **III**, respectively, in similar solvent), and shorter decay lifetimes (29.8 and 58.2 ns for **IV** and **III**, respectively, in similar solvents) [51, 52]. Excitation spectra in *n*‐pentane (Figure S36), obtained by recording the emission at 725 nm or at 660 nm, respectively, are perfectly superimposable, thus confirming the absence of impurities contributing to the dual emission peaks of **1**. In more polar solvents, such as toluene and diethylether, a substantial decrease in PLQY to 13% and 3%, respectively, and <1% in DCM is obtained. Solvent variation also strongly affects decay lifetimes, with values decreasing to 13 and 3 ns in toluene and diethylether, respectively (Figure S37). Note that other folded *p*‐QDM scaffolds do not fluoresce in solution but only in the solid state. This was ascribed by the authors to the internal molecular motions in solution that offer a non‐radiative decay pathway and hence quench the emission [70].

Compound **2** exhibits upon excitation at 440 nm red‐shifted bands in its emission spectra (Figure S55), with a broad band centered at 560 nm in *n*‐pentane corresponding to a Stokes shift of 0.63 eV, shifting to 650 nm in DCM (Stokes shift 0.90 eV). Just as was the case for **1**, varying the solvent affects the PLQYs, with higher values found in toluene and diethylether (88% and 96%, respectively) and 40% in *n*‐pentane and DCM. The decay times of around 10 ns in all solvents thereby suggest a fluorescence pathway (Figure S57). The emission characteristics of **2** rather resemble those reported for **IV** than for **III**, consistent with its corresponding UV–vis spectra. The emission properties of **3** (Figure S60) are similar to those of **2**, with maximal PLQYs in toluene and *n*‐pentane of 70% and 74%, respectively, and similar decay lifetimes in the investigated solvents (21 and 12 ns in *n*‐pentane and toluene, respectively, Figure S62). In *n*‐pentane, the Stokes shift for **3** is 1.01 eV, whereas it is 1.13 eV in dichloromethane. Pronounced solvatochromic emissions were observed for the three compounds with values of 1.81–2.0, 0.63–0.90, and 1.01–1.13 eV for **1**, **2**, and **3**, respectively, moving from *n*‐pentane to dichloromethane.

The emission properties of **1–3** (solvatochromism, PLQY) parallel **III** and **IV** [51, 52] and are attributed to a sudden polarization mechanism [78, 79, 80, 81]. However, the Stokes shift observed for **1** (1.81 eV in *n*‐pentane) is anomalously high compared to **2** and **3** (0.63 and 1.01 eV, respectively, in *n*‐pentane). The Lippert–Mataga analysis for **1** and **2** (Figure S65 and Table S8) indicates a significant increase of the dipole moment of the excited state compared to the ground state for both compounds and reveals that the nature of the excited state is preserved when moving from a bent geometry of **1** to the more planar one of **2**. The different Stokes shifts are hence due to a switch of the ground state geometry, as is also confirmed by the absorption spectra. Indeed, large Stokes shifts occur if excited‐state intramolecular relaxation leads to an energy minimum in significantly different geometry from the ground state. A similar strategy allows the design of twisted intramolecular charge‐transfer (TICT) and planarized intramolecular charge transfer (PLICT) emitters [91, 92, 93, 94, 95, 96, 98].

We further studied the behavior of **1** with low‐temperature photoluminescence (LT‐PL). In *n*‐pentane (Figures S40–S42), cooling from room temperature to 77 K leaves the emission maximum unchanged, but the excitation spectrum undergoes a bathochromic shift (from 340 to 375 nm) with a new low‐energy band appearing at 470 nm, a position that resembles those of the **2** and **3** excitation maxima at room temperature (440 and 408 nm for **2** and **3**, respectively), suggesting preferential formation of the planar conformer (**1^flat^**) in the excited state. At low temperature, the **1^flat^** population increases and its conversion back to the folded form slows due to reduced molecular mobility in the rigid matrix. Consequently, the low‐energy excitation band of **1^flat^** also becomes observable under steady‐state conditions. This effect becomes even more pronounced in 3‐methylpentane, which forms a higher‐quality glass at 77 K, and where the excitation band emerges at 485 nm, together with a slight hypsochromic shift of the emission maximum (Figures S43–S48).

Compounds **1**–**3** are also emissive in the solid state dispersed in BaSO_4_ with broad bands found at 685, 607, and 600 nm, respectively (Figures S38, S39, S58, S59, S63, and S64). To obtain better quality excitation and emission spectra for understanding the origins of the peculiar optical properties of **1**, it was additionally investigated in a polymeric glassy matrix (polymethyl methacrylate PMMA thin film at 10% doping) (Figures S49–S51). The glass matrix indeed allows for narrower bands compared to the opaque BaSO_4_ dispersion. The PMMA film of **1** exhibits a PLQY of 15% and a slightly red‐shifted emission maximum compared to measurements in *n*‐pentane solution (from 670 to 680 nm). The excitation spectrum of the PMMA film (Figure S50) is markedly red‐shifted (from 340 to 415 nm), further indicating that the interconversion from the planar conformer (**1^flat^**), formed in the excited state, back to the bent ground‐state geometry is slowed down in the rigid matrix, in full agreement with our LT‐PL results. Notably, the low‐energy excitation band appears at 415 nm (Figure S50), which is hypsochromically shifted relative to the spectra recorded in *n*‐pentane and 3‐methylpentane at 77 K (470 and 485 nm, respectively). This suggests that, while the planar conformer is still significantly populated in the excited state within the solid matrix, the extent of stabilization of **1^flat^** is reduced in PMMA compared to the glassy solvents, likely due to differences in local polarity and free volume. The thin film photophysics corroborate the proposed conformational equilibrium and demonstrate that environmental rigidity modulates the population and relaxation dynamics of the planar conformer, influencing compound **1’**s emission properties.

### 2.3 Femtosecond‐Transient Absorption Spectroscopy

**FIGURE 4:**
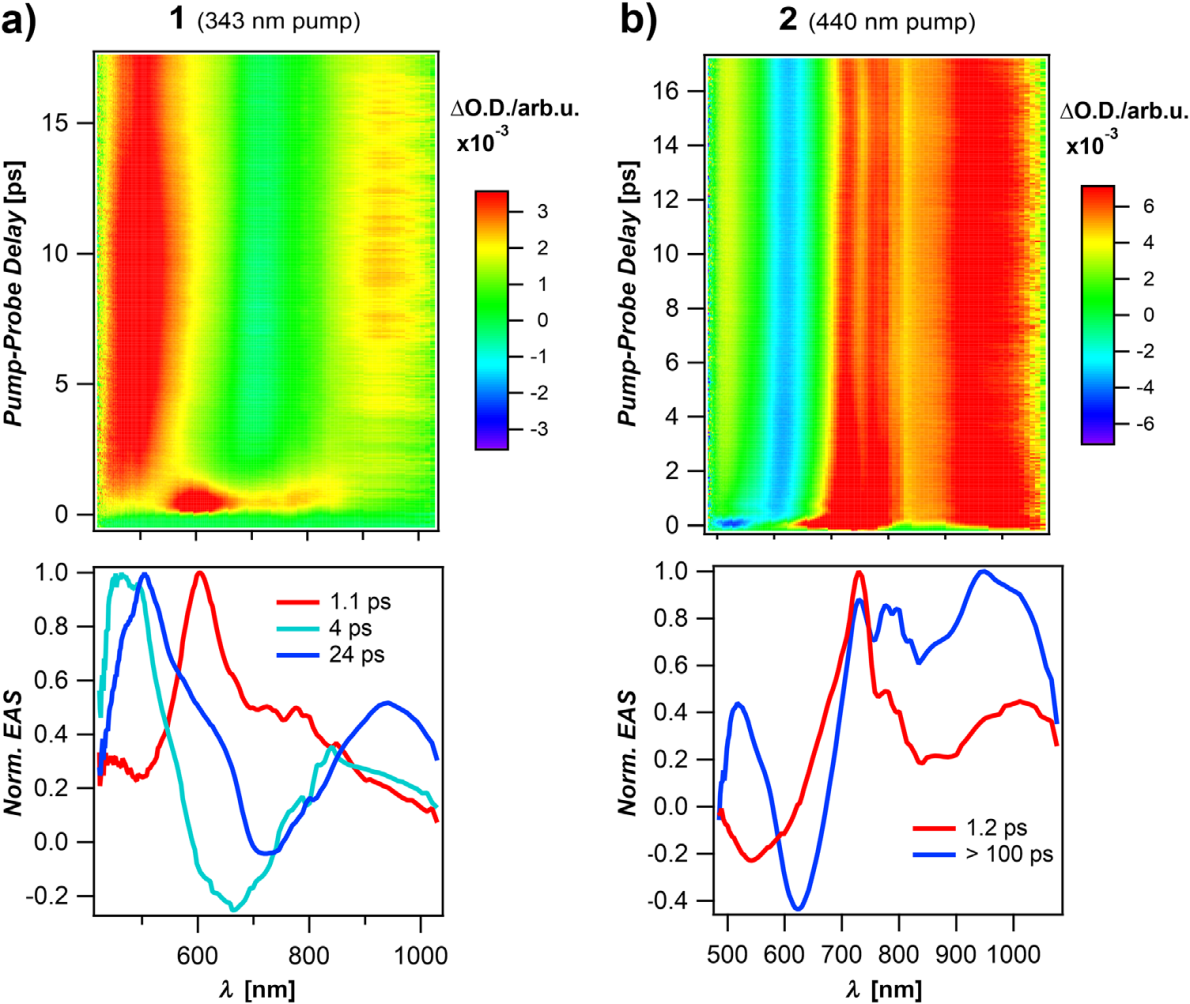
Femtosecond transient absorption (fs‐TA) maps of **1** (a, pumped at 343 nm) and **2** (b, pumped at 440 nm) in toluene. Bottom: Normalized evolution‐associated spectra (EAS) derived from sequential global fitting of the fs‐TA data. Whereas **2** shows two spectral components, one short‐lived assigned to SE and one long‐lived, **1** displays an additional intermediate.

Femtosecond‐transient absorption spectroscopy (fs‐TAS) was employed to investigate the excited‐state dynamics of compounds **1** and **2** in comparison with **III** [51]. Figure 4 presents the 2D fs‐TAS maps of **1** (excited at 343 nm) and **2** (excited at 440 nm), all measured in toluene. Each molecule was photoexcited at the respective absorption edge.

Global analysis of the fs‐TAS data was performed using a sequential kinetic model implemented in the Glotaran software package [99]. The normalized evolution associated spectra (EAS) are displayed below each corresponding fs‐TAS map. For **III** pumped at 515 nm (Figure S79), two spectrally distinct excited‐state components were identified in agreement with previous work [51]: these are a bright excited state with a lifetime of 4.6 ps and a dark excited state characterized by excited‐state absorption (ESA) at ∼700 nm.

Compound **2** shows comparable kinetics, with an initial short‐lived component with a lifetime of 1.2 ps, followed by a longer‐lived species that persists throughout the 15 ps experimental time window. Stimulated emission is observed at ∼600 nm, blue‐shifted with respect to compound **III**, as previously reported in Ref. [51]. The fs‐TAS maps of **2** (Figure 4b), **III**, and molecule **3** (Figure S79) exhibit striking similarities, suggesting that these compounds share similar excited‐state dynamics. In all cases, the stimulated emission arises from a non‐equilibrated excited‐state population at early times, prior to full vibrational and conformational relaxation, and therefore precedes the development of the Stokes shift.

In contrast, compound **1** (Figure 4a) exhibits significantly slower excited‐state evolution. Upon excitation at 343 nm, three distinct species were resolved, with lifetimes of 1.1, 4, and 24 ps. These species were also observed using pump wavelengths of 440 and 410 nm (Figure S80). These findings indicate that the initially populated excited state in **1** does not arise directly from photoexcitation but rather through internal relaxation processes, likely involving conformational changes. This behavior contrasts with compounds **2**, **III**, and **3**, whose excited‐state populations form rapidly, consistent with a rigid and planar ground‐state structure that requires minimal geometric reorganization.

### 2.4 Electrochemical Properties

**FIGURE 5:**
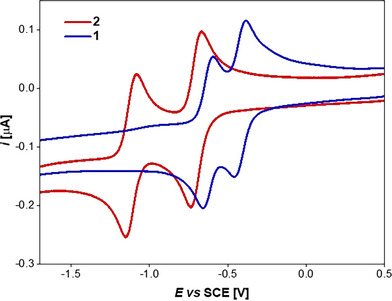
Cyclic voltammetry of **1** (blue line) and **2** (red line) obtained in DCM and 0.1 M [N*^n^* Bu_4_][PF_6_] with a glassy carbon electrode; *v* = 0.2 Vs^−1^.

The redox properties of **1** and **2** were assessed by cyclic voltammetry (CV) measurements in DCM (Figure 5). Both samples exhibit two reversible reduction peaks at rather low potentials, corresponding to the formation of the mono‐ and dianions. The first reduction of **1** occurs at *E*_1/2_ = −0.43 V versus SCE, whereas **2** is less easily reduced (*E*_1/2_ = −0.70 V). This observation suggests that either (i) the lowest unoccupied molecular orbital's (LUMO's) energy is rather controlled by the inductive effect of the four aryl rings than by the halides attached to the *p*‐QDM core [51, 52], or (ii) that the π‐donor properties of the in‐plane fluorine substituents considerably exceed the π‐donor capabilities of the Cl‐substituents, which do not align with the central C6 ring.

Notably, the separation between the first and second redox processes differs between the two molecules, measuring 590 mV for **2** and only 203 mV for **1**. This marked difference is likely due to the compound's steric profile, which controls the conformation (planarization vs. boat) both in the neutral form **1** as well as in the corresponding one‐electron reduced monoradical **1^●−^**. The latter is expected to be planar for enhanced electron delocalization through conjugation [100]. Thus, the smaller energy gap between the two redox processes observed for **1** is ascribed to a conformational change of the molecule upon one electron injection. This analysis is further supported by the estimated heterogenous electron transfer (ET) rate constant *k*°_het_ for the two consecutive ETs, determined by analyzing the change in cathodic–anodic peak separation (∆*E*_p_ = *E*_p,an_ − *E*_p,cat_) as a function of the voltammetry potential scan rate *v* (Figure S78) [101]. The first reduction step is a relatively slow ET process, with *k*°_het_ of 0.012 cm s^−1^, whereas the second ET exhibits much faster kinetics with *k*°_het_ = 0.050 cm s^−1^. This is different for **2**, where both ETs proceed with similar kinetics, characterized by a rate constant of 0.022 cm s^−1^. A low *k*°_het_ value is associated with a high intrinsic activation barrier and, as predicted by Marcus theory, a larger reorganization energy involved in radical anion formation. Since the contribution of solvent reorganization to the activation energy is expected to be similar for **1** and **2**, the observed difference is attributed to variations in the inner reorganization energy, that is structural changes such as bond length and bond angle modifications. In short, the electrochemical analysis reveals that the injection of the first electron into **1** induces a conformational change that renders the second ET significantly faster.

Regarding the oxidation [51, 52], the peak potential for the first oxidation of **2** is found at *E*_p_ = 2.00 V versus SCE (Figure S76), which allows us to conclude that the electrochemical HOMO–LUMO energy gap is about 2.67 eV (464 nm), which is consistent with the optical energy gap in DCM (485 nm estimated from the peak onset of the absorption spectra). A less pronounced oxidation peak was observed for **1**, allowing us to conclude that the electrochemical HOMO‐LUMO energy gap of **1** is larger than that of **2**.

The redox properties of compound **3** (Figure S77) are similar to those of **2**, with two reduction peaks and one oxidation peak. The first reduction potential is intermediate and in between those of **1** and **2** (*E*_1/2_ = −0.58 V vs. SCE), revealing that both the substituents on the central as well as on the exocyclic rings impact the LUMO's energy. Intermediate is also the value of the potential separation between the first and the second reduction (306 mV), and the oxidation peak is observed at *E*_p_ = +2.21 V vs. SCE.

### 2.5 Quantum‐Chemical Calculations

**SCHEME 2:**
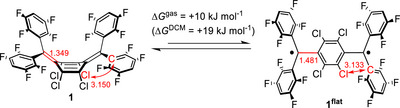
Computed structural parameters (values given in Å) and equilibrium between **1** and **1^flat^**.

Density functional theory (DFT; PBE0‐D4, TPSSh‐D4, ωB97X‐V), ab initio (NEVPT2 and CASPT2K//CASSCF) as well as multiconfigurational pair density functional theory (CI‐sr‐ctPBE0) calculations [102, 103, 104] were conducted to understand the interplay between diradical character, conformation, solvation, and emission. Computations using the ωB97X‐V functional (Tables S17 and S18) confirm thereby that compounds **2** and **3** are flat both in solution as well as in the solid state. In stark contrast, quinodimethane **1** prefers the boat conformation (Δ*G* = +10 kJ mol^−1^; Scheme 2). Solvation, which was modelled implicitly for DCM, further shifts the conformational equilibrium (Δ*G* ^DCM^ = +19 kJ mol^−1^) toward the bent conformer, and the same is true for the adiabatic energy difference to the triplet excited state (**1^t^**; Δ*G* ^gas^ = +12 kJ mol^−1^, Δ*G* ^DCM^ = +19 kJ mol^−1^).

The computations show high spin contamination (<*S* ^2^> = 1.1) for isomer **1^flat^** and an aromatic central benzene ring without substantial bond length alternation (*d* ^C–C^ ∼ 1.41–1.40 Å; Tables S10 and S11), indicating a high degree of diradical character. In stark contrast, the boat‐conformer **1** shows a closed‐shell quinoid electronic structure (<*S* ^2^> = 0). The enhanced π‐overlap of the fluorine‐ (in **2**) in respect to the chlorine (in **1**) lone‐pairs with the central ring aids in the dearomatization and hence stabilization of the quinoid resonance form (Tables S10 and S11); a similar effect is in play for the flanking aryl groups, where fluorine substitution aids in electron delocalization (**1** vs. **III**) [49]. Further, the larger van der Walls radius of chlorine (1.75 Å) than fluorine (1.47 Å) and carbon (1.70 Å) elongates the exocyclic C═C bonds (**1**, *d* ^C═C^ = 1.349 Å; **1^flat^**, *d* ^C═C^ = 1.481 Å; **2**, *d* ^C═C^ = 1.468 Å; Figure S81), hence also stabilizing the diradical form in **1^flat^**. This elongation also increases the average distances to the *ipso*‐carbon atoms (**1**, *d* ^Cl–Cipso^ = 3.150 Å; **1^flat^**, *d* ^Cl–Cipso^ = 3.133 Å; **2**, *d* ^F–Cipso^ = 2.799 Å) and releases steric pressure, just as is the case for the conformational change to the boat conformer. In short, molecule **1** adopts a bent conformation to mitigate the high diradical character of **1^flat^** through minimizing *π*‐orbital overlap and steric pressure [105].

We then modeled the single‐electron excitation of diradicaloids **1**, **1^flat^**, and **2** by TDDFT (Table S16). In line with the experiment, TDDFT predicts the absence of a substantial solvatochromic effect for the intense single‐electron HOMO–LUMO transition in **1** (gas phase, pentane, DCM: *E*(S1) = 3.47 eV), **1^flat^** (gas phase: *E*(S1) = 2.09 eV; pentane: *E*(S1) = 2.05 eV; DCM: *E*(S1) = 2.04 eV), and **2** (gas phase: *E*(S1) = 2.85 eV; pentane: *E*(S1) = 2.71 eV; DCM: *E*(S1) = 2.70 eV). These computations hence further corroborate that emission in compound **1** does not occur from this singly excited (SE) state.

**FIGURE 6:**
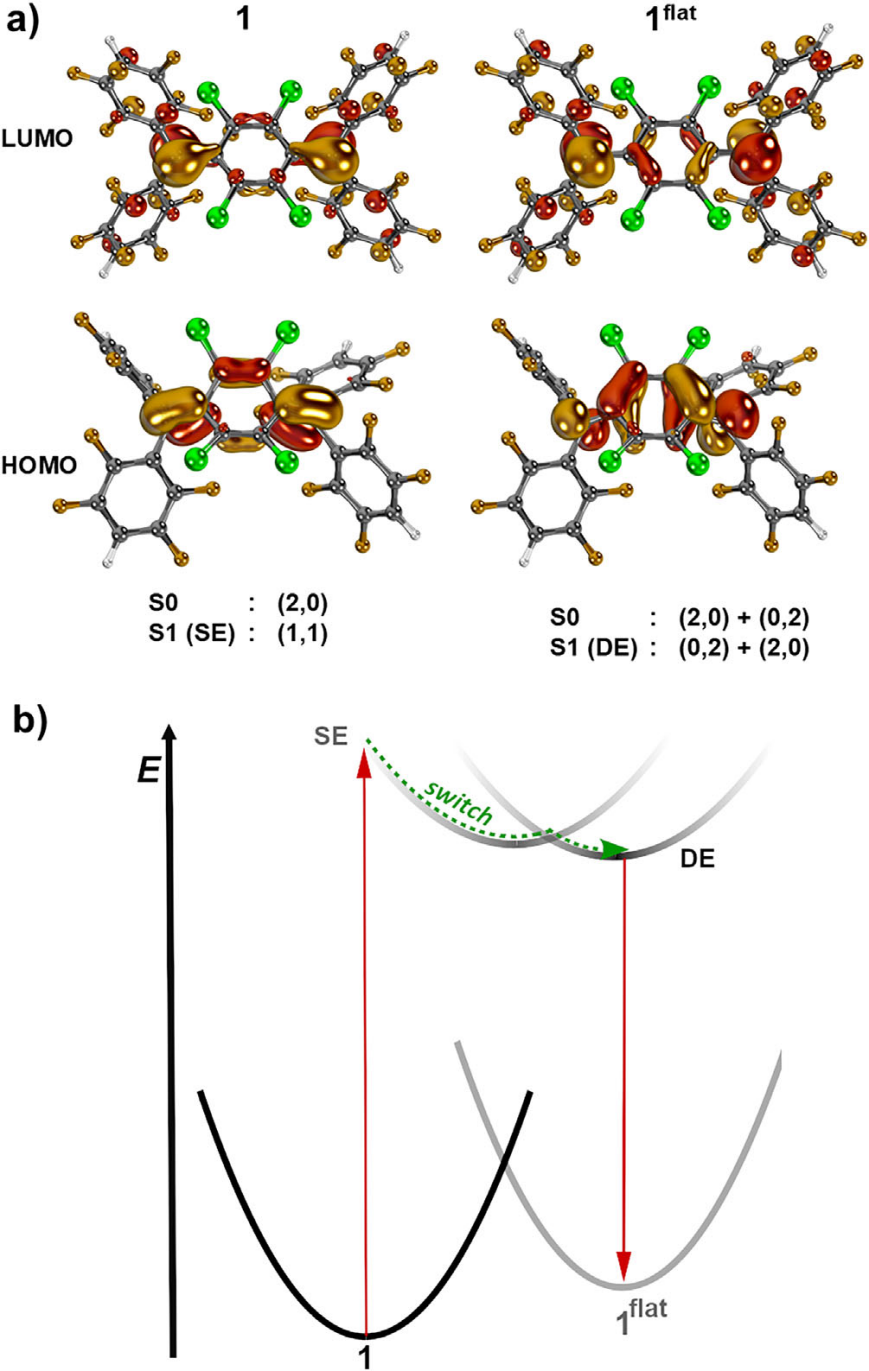
Idealized nature of the S0→S1 transitions in **1** (top, left) and 1^flat^ (top, right) according to CASSCF(14,14) as well as simplified potential energy diagram (bottom).

Therefore, the electronic structures of compounds **1**, **1^flat^**, and **2** as well as **3** (SI) were further explored by NEVPT2/CASSCF, CASPT2K/CASSCF and CI‐srDFT calculations, which all allow for modeling doubly excited (DE) states. The S0 ground state of the boat conformer **1** is essentially closed‐shell in character with only a very minor degree of diradical character *y*_0_ = 0.08 according to CASSCF(14,14)/def2‐TZVPP calculations (Figure 6, top).

The S0→S1 transition (NEVPT2//CASSCF(14,14) relates to the H→L (π→π*) transition within the quinodimethane to the SE S1 state ((HOMO)^1^(LUMO)^1^ configuration with a weight of *c* ^2^ = 0.51 (class I chromophore) [106]. The LUMO lacks significant molecular overlap between the exocyclic diarylmethylene group and the linker, hence facilitating conformational relaxation of the boat‐like structure to the flat isomer. Indeed, optimizing the structural parameters of the S1 excited state of **1** by CASSCF(6,6) affords a flat conformer (Figure S92), which prevents both radiative‐ and non‐radiative relaxation to boat‐conformer **1**. In stark contrast, the electronic structure of the S0 ground state of **1^flat^** is dominated by two configurations, namely (HOMO)^2^(LUMO)^0^ with a weight of *c* ^2^ = 0.49 and (HOMO)^0^(LUMO)^2^ with a weight of *c* ^2^ = 0.31 according to CASSCF(14,14). These zwitterionic resonance structures render **1^flat^** strongly diradicaloid in nature (class III‐type chromophore; *y*_0_ = 0.78) [107]. The vertical excited S1 state of **1^flat^** represents tetraradical character with substantial weight of the (HOMO)^0^(LUMO)^2^ and (HOMO)^2^(LUMO)^0^ configurations, rendering it a DE and consequently dark state. Notably, the vertical S2 state, which is of SE character, is predicted to be only slightly higher in energy (Δ*E* = 0.18 eV; CI‐sr‐ctPBE0), whereas the T1 excited state of **1^flat^** is close in energy to the singlet ground state (Δ*E* = 0.15 eV). Opposed to the HOMO and LUMO in **1**, the LUMO in **1^flat^** is non‐bonding between the exocyclic radical and the central phenylene ring. This suggests that the S1 excited state exhibits only a low barrier for rotation around this C─C bond (Figure S91), thereby generating an asymmetric excited conformer with non‐negligible dipole moment (sudden polarization) [52, 80, 81] as suggested by conformational scans at the CASSCF(6,6) level of theory. Overall, the computations therefore reveal that excitation of **1** triggers the geometric change to a flat conformer. This conformer **1^flat^** shows high diradical character in the S0 ground state and a long‐lived doubly excited (DE) S1 state of zwitterionic character, which leads to strong positive solvatochromism in the emission spectra.

## 3 Conclusion

This work adds a new dimension to the photophysics of diradicals, namely the interplay of two conformers of quinodimethanes. These isomers relate to a bent, closed‐shell quinoid **1** as well as a flat structure of exceptional high diradical character **1^flat^** (*y*_0_ = 0.78). As demonstrated by comparison with the quinodimethanes **2** and **3**, the conformational switch is controlled by halogen‐decoration, which adjusts the diradical character in the flat isomer. Single electron excitation of the quinoid and air‐stable conformer **1** leads to a conformational change to the flat isomer **1^flat^** in a doubly‐excited S1 state. This long‐lived dark state is of zwitterionic character and provides a photoluminescence quantum yield near unity (PLQY = 100%) with overall large positive solvatochromism (emission from 670 nm = 1.85 eV in *n*‐pentane to approximately 772 nm = 1.6 eV in DCM) and exceedingly large Stokes shifts (between 1.81 eV in *n*‐pentane to even 2.0 eV in DCM). We hence delineate a strategy on how to harness the unique photophysical properties of usually highly air‐sensitive genuine diradicals with air‐stable closed‐shell compounds.

## Conflicts of Interest

The authors declare no conflict of interest.

## Supporting information




**Supporting File 1**: The authors have cited additional references within the Supporting Information [1–35].

**Supporting File 2**: anie71303‐sup‐0002‐SuppMat.zip.

## Data Availability

The data that support the findings of this study are available in the Supporting Information of this article.
