# Supplementary material for: Hidden Diradical: Conformational Switch for Solvatochromic NIR Emission With Unity Quantum Yield in Thiele's Hydrocarbon
Source: Angew Chem Int Ed Engl. 2026 Jan 28;65(10):e24042. doi: 10.1002/anie.202524042 (PMC12955538; doi:10.1002/anie.202524042)
Supplement: Supplementary file 1 — Supporting File 1: The authors have cited additional references within the Supporting Information [1–35]. [file ANIE-65-e24042-s001.pdf]

Supporting Information

Hidden Diradical: Conformational Switch for Solvatochromic NIR Emission with Unity Quantum Yield in Thiele's Hydrocarbon

Matteo Bevilacqua,[a,e] Mattia Reato,[a] Federico Cilento,[b] Claudia Graiff,[c] Sabrina Antonello,[a] Luca Schio,[d] Alessandro Aliprandi,[a] Cristina Tubaro,[a] Lorenzo Franco,[a] Martina Dell'Angela,[d]* Dominik Munz[e]* and Marco Baron[a]*

[a] Dr. M. Bevilacqua, Dr. M. Reato, Prof. Dr. S. Antonello, Prof. Dr. A. Aliprandi, Prof. Dr. C. Tubaro, Prof. Dr. L. Franco, Prof. Dr. M. Baron
Dipartimento di Scienze Chimiche
Università Degli Studi di Padova
Via Marzolo 1, 35131, Padova, Italy
E-mail: marco.baron@unipd.it

[b] Dr. F. Cilento
Elettra-Sincrotrone Trieste S.C.p.A.
34149 Basovizza, Italy

[c] Prof. Dr. C. Graiff
Dipartimento di Scienze Chimiche, della Vita e della Sostenibilità Ambientale
Università di Parma
Parco Area delle Scienze 17/a, I-43124 Parma, Italy

[d] Dr. L. Schio, Prof. Dr. M. Dell'Angela
CNR – Istituto Officina dei Materiali (IOM)
S.S. 14 km 163.5, Area Science Park, Basovizza, Trieste, I-34149 Italy
E-mail: dellangela@iom.cnr.it

[e] Dr. M. Bevilacqua, Prof. Dr. D. Munz
Coordination Chemistry
Saarland University
Campus C4.1, D-66123 Saarbrücken, Germany
E-mail: dominik.munz@uni-saarland.de

Table of contents

1. Materials and methods S1
2. Synthetic procedures S3
3. NMR spectra S6
4. UV-Vis electronic absorption spectra S19
5. Emission and excitation spectra and lifetime measurements S26
6. Crystallographic data S44
7. Thermogravimetric analysis S49
8. EPR spectra of **1** and **2** S51
9. Cyclic voltammetry of **1**, **2** and **3** S52
10. fs-TAS of **III**, **3** and **1** S54
11. Computational Details S56
12. References S92

1. Materials and methods

All analyses and operations were performed under ambient conditions unless otherwise specified. 1,2,4,5-tetrafluorobenzene, 1,3,5-trichlorobenzene, aluminum trichloride, hydrochloric acid (1 M), sodium sulfate and tetrabutylammonium hydroxide (55% w/w) were purchased from Sigma-Aldrich and used as received. Acetonitrile (CH_3CN), tetrahydrofuran (THF), dichloromethane (DCM), chloroform ($CHCl_3$), diethyl ether (Et_2O), methanol (MeOH), *n*-hexane, *n*-pentane and deuterated solvents were purchased from Sigma-Aldrich. Unless otherwise noted, all the purchased solvents were anhydrous and of high purity-grade and were used as received. THF was distilled prior to use. Compound **III** was prepared according to literature procedure.[1]

^{1}H and ^{13}C NMR spectra were recorded on a Bruker Avance 400 MHz (400 MHz for ^{1}H, 100 MHz for ^{13}C) instrument, ^{19}F NMR spectra were recorded on a Bruker Avance 200 MHz (188 MHz for ^{19}F) instrument; chemical shifts (δ) are reported in parts per million (ppm) relative to the solvent signal in case of ^{1}H and ^{13}C spectra. In case of ^{19}F the signal of 1,3,5-trifluorobenzene was used as reference. The multiplicities are reported as followed: singlet (s), doublet (d), triplet (t), quartet (q), quintet (qu), septuplet (st), multiplet (m). The coupling constants (J) are reported in Hz.

Absorption spectra were recorded with a Perkin-Elmer λ950 or λ650 spectrophotometer. Emission spectra were recorded with an Edinburgh photoluminescence spectrometer model FLS1000, with double monochromators, equipped with a 450W Xe arc lamp as the excitation source. A photomultiplier tube R13456 with a spectral response from 185 to 980 nm was used as detector. For the emission spectra, the step and dwell time were set at 1 nm and 0.1 s, respectively, and the slit was kept at 0.1 nm and 3 nm for excitation and emission monochromators, respectively. Photoluminescence Quantum yields (*PLQY*) were calculated by measuring the emission spectrum of samples dissolved in the indicated solvent in a $BaSO_4$ coated integration sphere, mounted in the FLS1000 instrument. A cuvette containing the analyzed solvent was used for the measurement of the reference spectrum. The excitation wavelength was fixed according to UV-Vis bands of samples. The emission spectra were recorded in the range 330–980 nm. The formula used for the calculation of *PLQY* is:

$$\boldsymbol{PLQY} = \frac{\int \boldsymbol{I}_{\boldsymbol{em,sample}}}{\int \boldsymbol{I}_{\boldsymbol{abs,solvent}} - \int \boldsymbol{I}_{\boldsymbol{abs,sample}}}$$

where $\int \boldsymbol{I}_{\boldsymbol{em,sample}}$ is the integrated emission intensity of the sample, $\int \boldsymbol{I}_{\boldsymbol{abs,sample}}$ and $\int \boldsymbol{I}_{\boldsymbol{abs,solvent}}$ are the integrated absorption of the sample and solvent, respectively.

The crystallographic data for compounds **1-H**, **2-H**, **1**, **2** and **3** were collected on a Bruker D8 Venture Photon II single-crystal diffractometer working with monochromatic Mo-K_α radiation. The structures were solved and refined against F^2 with SHELXL-2018/3 with anisotropic thermal parameters for all non-hydrogen atoms.[2,3] Idealized geometries were assigned to the hydrogen atoms. Crystallographic data were deposited with the Cambridge Crystallographic Data Centre as supplementary publication. Copy of the data (CCDC Deposition Numbers 2433178-2433182 for

compounds **2-H**, **1-H**, **2**, **1**, **3** respectively) can be obtained free of charge on application to the CCDC, 12 Union Road, Cambridge CB2 1EZ, U.K. (fax, (+44) 1223 336033; e-mail, deposit@ccdc.cam.ac.uk).[4]

Femtosecond transient absorption spectroscopy (fs-TAS). Experiments were conducted at the T-ReX laboratory (FERMI@Elettra, Trieste) using a Ti:sapphire laser system (Coherent Legend Elite DUO He+ USP), generating ≈35 fs pulses at a 1 kHz repetition rate with a central wavelength of 795 nm and a spectral bandwidth of 30 nm. Pump pulses were produced using an optical parametric amplifier (OPA), while a broadband probe supercontinuum was generated by focusing a small portion of the laser output into a CaF_2 window. The resulting probe covered the 500–1000 nm spectral range. Global analysis was carried out using a sequential kinetic model with the Glotaran software package.

The electrochemical experiments were carried out in DCM containing 0.1 M tetrabutylammonium hexafluorophosphate $TBAPF_6$, under an Ar atmosphere, in a glass cell thermostated at 25 ± 1 °C (unless otherwise specified). A 0.12 mm-radius glassy carbon disk prepared and activated as previously described,[5] was the working electrode, whereas a Pt wire was the counter electrode. An Ag wire, separated from the main electrolytic compartment by a VycorR frit, was used as a quasi-reference electrode. At the end of each experiment, the potential of the latter was calibrated against the ferrocenium/ferrocene redox couple (Fc^+/Fc) (in DCM/0.1 M $TBAPF_6$, $E°$ = 0.460 V versus the KCl saturated calomel electrode, SCE). To minimize the ohmic drop between the working and the reference electrodes, careful feedback correction was applied. A SP-300 electrochemical workstation (BioLogic, France) was used.

Thermogravimetric analyses (TGAs) of samples **1**, **2** and **3**, were carried out with a Q5000IR TGA (TA Instruments) under dinitrogen by an isotherm at 100 °C for 10 minutes followed by heating at 20 °C min^{-1} rate until 1000 °C.

EPR spectra were recorded on a Bruker ECS 106 X-band spectrometer using a 4108 TMH cavity (9.5 GHz). The temperature was varied in the 130–350 K range by a dinitrogen flow cryostat and a temperature controller Bruker BVT2000. Samples of **1** and **2** were analyzed in toluene solutions ($c \approx 2\times10^{-4}$ M). Prior to EPR measurement, the solutions were degassed by thorough dinitrogen bubbling. Typical spectrometer settings were: field sweep 200 Gauss, microwave power 6 mW, field modulation amplitude 1 Gauss, scan time 40 s, 30 scans.

2. Synthetic procedures

Synthesis of 1,2,4,5-tetrafluoro-3,6-bis(dichloromethyl)benzene

This molecule has been synthetized according to a modified literature procedure.[6] A mixture of 1,2,4,5-tetrafluorobenzene (1.0 g, 6.7 mmol) and aluminium chloride (5.0 g, 32.5 mmol) in anhydrous chloroform (25 mL) was refluxed under inert atmosphere for 72 h. The reaction mixture was cooled to room temperature, diluted with chloroform (100 mL) and poured into a mixture of aqueous hydrochloric acid 1 M (100 mL) and ice-water (100 mL). The organic layer was separated, dried over sodium sulfate and concentrated to give a brown sticky solid. The solid was dispersed in 15 mL of *n*-pentane and the mixture filtrated on celite. The filtrate was concentrated at reduced pressure, affording 1.1 g (52%) of the product as a sticky yellowish solid. ^{1}H NMR (400 MHz, $CDCl_3$): δ 6.96 (br, 2H) ppm. ^{19}F NMR (200 MHz, $CDCl_3$): δ –139.49 (s, 4F) ppm.

Synthesis of 1,2,4,5-tetrachloro-3,6-bis(dichloromethyl)benzene

This molecule was synthetized according to a modified literature procedure.[1] A mixture of 1,2,4,5-tetrachlorobenzene (1.0 g, 4.5 mmol) and aluminium chloride (3.0 g, 22.6 mmol) in anhydrous chloroform (12 mL) was refluxed under inert atmosphere for 72 h. The reaction mixture was cooled to room temperature, diluted with chloroform (40 mL), and poured into a mixture of aqueous hydrochloric acid 1 M (50 mL) and ice-water (50 mL). The organic layer was separated, dried over sodium sulfate and concentrated to give a beige solid. The solid was washed with 5 mL of *n*-pentane and dried at reduced pressure, affording 1.4 g (80% yield) of the product as a beige solid. ^{1}H NMR (400 MHz, $CDCl_3$): δ 7.63 (br, 2H) ppm.

*Synthesis of **1-H***

Into an ACE pressure tube (15 mL), equipped with a magnetic stir bar, 300 mg of 1,2,4,5-tetrachloro-3,6-bis(dichloromethyl)benzene (0.8 mmol), 1.5 g of 1,2,4,5-tetrafluorobenzene (1.2 mL, 10.0 mmol) and 630 mg of aluminium chloride (4.7 mmol) were introduced under inert atmosphere. The mixture was heated at 90 °C under stirring for 21 h. The reaction mixture was cooled to room temperature, diluted with DCM (200 mL), and poured into a mixture of aqueous hydrochloric acid 1 M (100 mL) and ice-water (100 mL). The organic layer was separated, dried over sodium sulfate and concentrated to give a brown solid. The solid was dissolved in 2 mL of DCM, and 200 mL of *n*-hexane were added, affording a dispersion of the solid. The insoluble solid was filtered over celite and the filtrate was collected. The filtrate was concentrated at reduced pressure and loaded onto a chromatographic silica column, using *n*-hexane as eluent (R_f: 0.25), affording 216 mg (33% yield) of **1-H** as white crystalline solid. Crystals suitable for SCXRD were obtained by slow evaporation of a DCM solution of **1-H**. ^{1}H NMR (400 MHz, $CDCl_3$): δ 7.10 (m, 4H), δ 6.77 (br, 2H). ^{19}F NMR (200 MHz, $CDCl_3$): δ –138.89 (m, 8F), δ –141.38 (m, 8F) ppm. ^{13}C {^{1}H} NMR (101 MHz, $CDCl_3$): δ 147.40 (tt, ^{1}J = 12 Hz, ^{2}J = 4 Hz, C_2), δ 146.45 (dt, ^{1}J = 15 Hz, ^{2}J = 4 Hz, C_3), δ 144.93 (tt, ^{1}J = 12 Hz, ^{2}J = 4 Hz, C_5), δ 144.27 (dt, ^{1}J = 15 Hz, ^{2}J = 4 Hz, C_6), δ 136.15 (s, C_9), δ 134.78 (s, C_8), δ 111.34 (t, J = 13 Hz, C_4), δ 106.20 (t, J = 22 Hz, C_1), 38.18 (s, C_7) ppm. UV-Vis ($CHCl_3$, 1.7×10^{-3} M): 273 nm (4827 M^{-1} cm^{-1}).

*Synthesis of **2-H***

Into ACE pressure tube (15 mL) equipped with a magnetic stir bar, 304 mg of 1,2,4,5-tetrafluoro-3,6-bis(dichloromethyl)benzene (1.0 mmol), 1.3 g (8.4 mmol) of 1,3,5-trichlorobenzene and 850 mg of aluminium chloride (6.4 mmol) were introduced. The mixture was heated to 90 °C for 21 h. After this time, the hot mixture was poured into a mixture of aqueous hydrochloric acid 1 M (100 mL) and ice-water (100 mL), and the product extracted with $CHCl_3$ (3 × 100 mL). The organic phase was separated, dried over sodium sulfate, and concentrated at reduced pressure to give a brownish solid. The solid was dispersed in 50 mL of *n*-hexane. The mixture was filtered over celite and the filtrate concentrated at reduced pressure. The obtained solution was loaded onto a chromatographic silica column, using *n*-hexane as eluent (R_f: 0.2). The solvent was removed at reduced pressure, affording 317 mg (31% yield) of **2-H** as a brownish solid. Crystals suitable for SCXRD were obtained from by slow evaporation of a $CHCl_3$ solution of **2-H.** ^1H NMR (400 MHz, $CDCl_3$): δ 7.32 (m, 8H), δ 6.23 (m, 2H). ^{19}F NMR (200 MHz, $CDCl_3$): δ –141. 13 (s, 1F), – 141.78 (m, 1F), –142.46 (m, 1F) – 143.16 (s, 1F) ppm. ^{13}C {^1H} NMR (101 MHz, $CDCl_3$): δ 137.12 (C_4, C_8), 134.16 (C_1) 132.63 (C_3, C_5), 129.58 (C_2, C_6), 118.11 (C_9), 42.82 (C_7) ppm. UV-Vis (*n*-pentane, 1.3 × 10^{-4} M): 275 nm (5065 M^{-1} cm^{-1}), 348 nm (125 M^{-1} cm^{-1}), 363 nm (230 M^{-1} cm^{-1}), 384 nm (392 M^{-1} cm^{-1}), 407 nm (333 M^{-1} cm^{-1}).

*Synthesis of **1***

In a one-neck round-bottom flask (250 mL) equipped with a magnetic stir bar, 127 mg of **1-H** (0.15 mmol) were dissolved in 150 mL of freshly distilled THF at room temperature. 760 µL of a solution of tetrabutylammonium hydroxide (55% w/w) (1.5 mmol) was added dropwise over 5 minutes, affording a red solution. Immediately after the addition of the base, the solvent was removed by a rotary evaporator, affording an orange solid. The solid residue was dispersed in 50 mL of DCM:hex (1:1) and purified by flash column silica chromatography, collecting all colored fractions (R_f: 0.1 - 0.3 in *n*-hexane), affording a yellow solid. UV-Vis, NMR and TLC (*n*-hexane) analyses of the solid indicated the presence of starting materials and the product. Therefore, a second separation was performed, using *n*-hexane as eluent (R_f: 0.1), affording 11 mg of **1** (23 % yield) as ayellow solid. Crystals suitable for SCXRD were obtained by slow evaporation of an *n*-pentane solution of **1**. ^1H NMR (400 MHz, $CDCl_3$): δ 7.13 (br, 4H) ppm. ^{19}F NMR (200 MHz, $CDCl_3$): δ –137.79 (br, 8F), –138.33 (br, 8F) ppm. ^{13}C {^1H} NMR (101 MHz, $CDCl_3$): δ 147.30 (br, C_2), 145.28 (br, C_3), 144.81 (br, C_5), 142.80 (br, C_6), 138.15 (s, C_9), 130.64 (s, C_8), 121.31 (br, C_7), 117.90 (br, C_4), 107.68 (t, J = 22 Hz, C_1) ppm. UV-Vis ($CHCl_3$, 3.4 × 10^{-5} M): 271 nm (14190 M^{-1} cm^{-1}), 341 nm (22955 M^{-1} cm^{-1}).

*Synthesis of **2***

In a one-neck round-bottom flask (250 mL) equipped with a magnetic stir bar, 100 mg of **2-H** (0.11 mmol) were dissolved in 100 mL of freshly distilled THF at room temperature. 520 µL of a solution of tetrabutylammonium hydroxide (55% w/w) (1.10 mmol) was added dropwise over 5 minutes, affording a red solution that turned blue within 2 minutes. After 5 minutes, the solvent was removed by a rotary evaporaotr, affording a blue solid. The solid residue was dispersed in 100 mL of DCM:hex (1:1) and purified by flash column silica chromatography (R_f: 0.4), using DCM:hex (1:1) as eluent. The solvent was removed at reduced pressure, affording 86 mg (87% yield) of **2** as an orange solid. Crystals suitable for SCXRD were obtained by slow evaporation of an *n*-hexane solution of **2**. ^1H NMR (400 MHz, $CDCl_3$): δ 7.38 (m, 4H), 7.27 (m, 4H) ppm. ^1H NMR (400 MHz, CD_2Cl_2): δ 7.44 (m, 4H), 7.33 (m, 4H) ppm. ^1H NMR (400 MHz,

$C_2D_2Cl_4$): δ 7.43 (m, 4H), 7.33 (m, 4H) ppm. ^{19}F NMR (200 MHz, $CDCl_3$): δ –138.53 (br, 8F) ppm. ^{13}C {^{1}H} NMR (101 MHz, $CDCl_3$): δ 144.34 (C_4), 144.8 (C_8), 139.4 (C_1), 135.93 (C_3), 134.59 (C_5), 129.57 (C_2), 128.52 (C_6), 125.60 (C_9), 66.02 (C_7) ppm. UV-Vis ($CHCl_3$, 1.3 × 10^{-5} M): 293 nm (16507 M^{-1} cm^{-1}), 438 nm (51567 M^{-1} cm^{-1}).

*Synthesis of **3***

In a one-neck round-bottom flask (250 mL) equipped with a magnetic stir bar, 400 mg of **1-H** (0.48 mmol) were dissolved in 80 mL of freshly distilled THF at room temperature. A solution of tetrabutylammonium hydroxide (55% w/w, 4.8 mmol, 2.4 mL) was added dropwise, affording a red solution. The solution was left under stirring at room temperature for 24 h. Volatiles were removed by a rotary evaporator, affording a purple solid. The solid was dispersed in 50 mL of DCM and purified by a flash column silica chromatography, collecting all colored fractions (R_f: 0.1 - 0.3 in *n*-hexane), affording an orange solid. The crude product was further purified by a second column, using hex:DCM (8:2) as eluent, and collecting the diradicaloid fraction (R_f: 0.25), affording 28 mg of **3** (8 % yield) as an orange solid. Crystals suitable for SCXRD were obtained by slow evaporation of an *n*-pentane solution of **3**. ^{1}H NMR (400 MHz, CD_2Cl_2): δ 7.11 (m, 6H), 6.75 (m, 2H) ppm. ^{1}H NMR (400 MHz, $CDCl_3$): δ 7.17 (m, 6H), 6.77 (m, 2H) ppm. ^{19}F NMR (200 MHz, CD_2Cl_2): δ –139.06 (m, 4F),–139.84 (m, 12F),–140.37 (d, *J* = 10 Hz, 1F),–140.74 (s, 1F),–141.15 (d, *J* = 10 Hz, 1F),–142.04 (m, 8F) ppm. ^{19}F NMR (200 MHz, $CDCl_3$): δ –138.41 (m, 4F),–138.85 (m, 12F),–139.78 (d, *J* = 10 Hz, 1F),–140.03 (s, 1F),–140.16 (s, 1F),–140.45 (s, 1F),–141.44 (m, 8F) ppm. ^{13}C{^{1}H} NMR (101 MHz, CD_2Cl_2): δ 147.72 (C_2, m), 147.36 (C_3, m), 147.04 (C_5, m), 146.51 (C_6, br), 145.24 (C_{16}, m), 144.91 (C_{17}, m), 144.63 (C_{19}, m), 144.25 (C_{20}, m), 143.94 (C_{21}, m), 141.77 (C_{14}, m), 138.09 (C_{10}, s), 137.04 (C_{12}, s), 134.34 (C_9, s), 133.90 (C_{13}, s), 124.13 (C_8, m), 123.69 (C_4, m), 117.74 (C_{22}, t), 117.42 (C_{23}, t), 108.14 (C_{18}, t), 106.52 (C_1, t), 38.31 (C_7, s) ppm. The quaternary C_{11} and C_{15} signals could not be detected due to low intensity. UV-Vis (DCM, 1.0 × 10^{-5} M): 274 nm (16383 M^{-1} cm^{-1}), 408 nm (42708 M^{-1} cm^{-1}).

3. NMR spectra

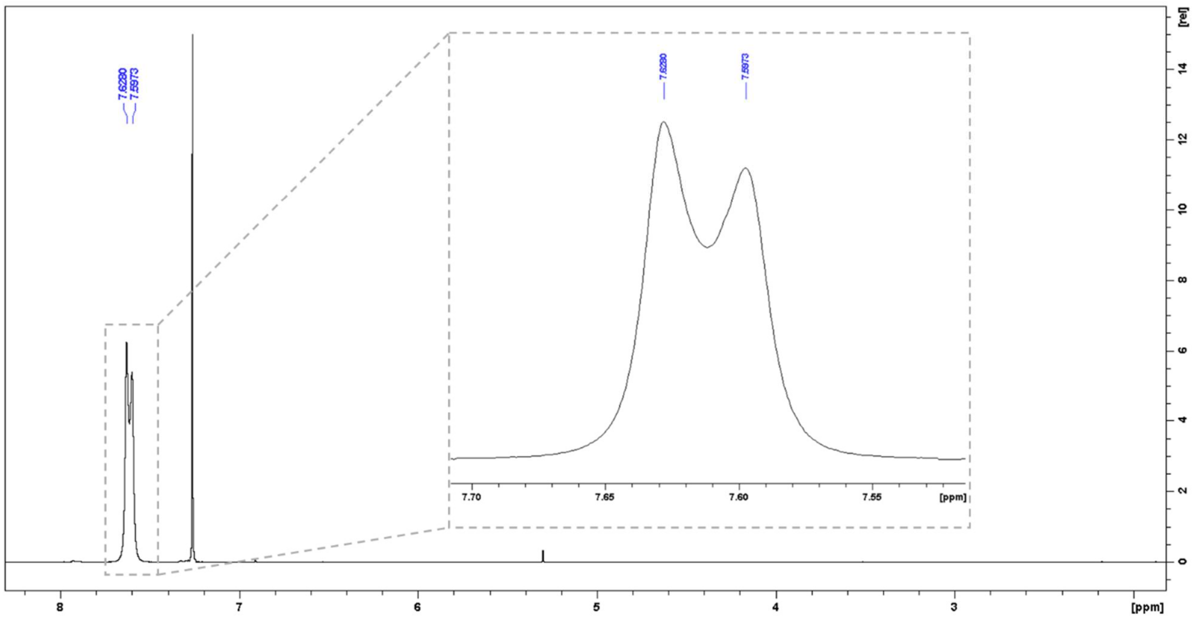

Figure S1: ^{1}H NMR (400 MHz, $CDCl_3$) spectrum of 1,2,4,5-tetrachloro-3,6-bis(dichloromethyl)benzene.

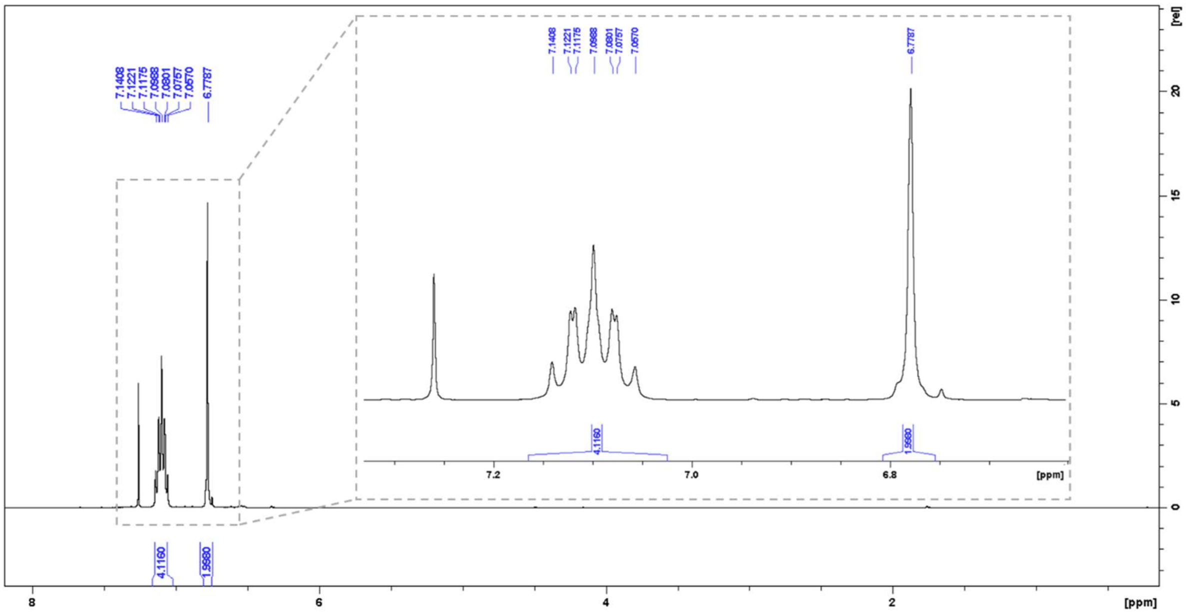

Figure S2: ^{1}H NMR (400 MHz, $CDCl_3$) spectrum of **1-H**.

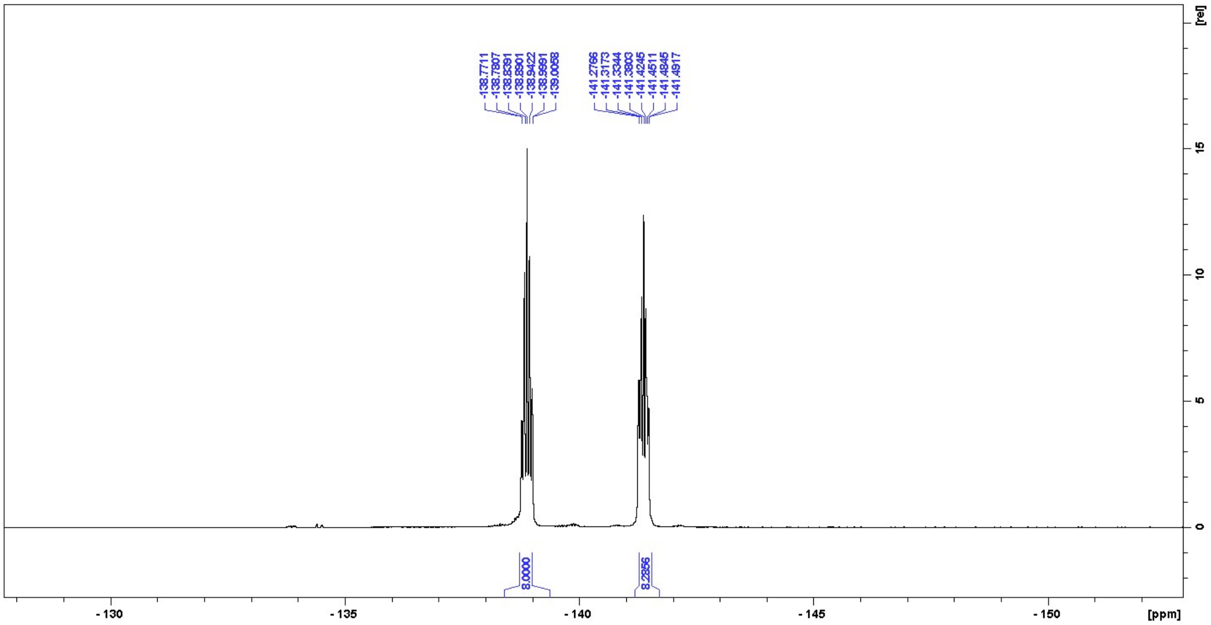

Figure S3: ^{19}F NMR (200 MHz, $CDCl_3$) spectrum of **1-H**.

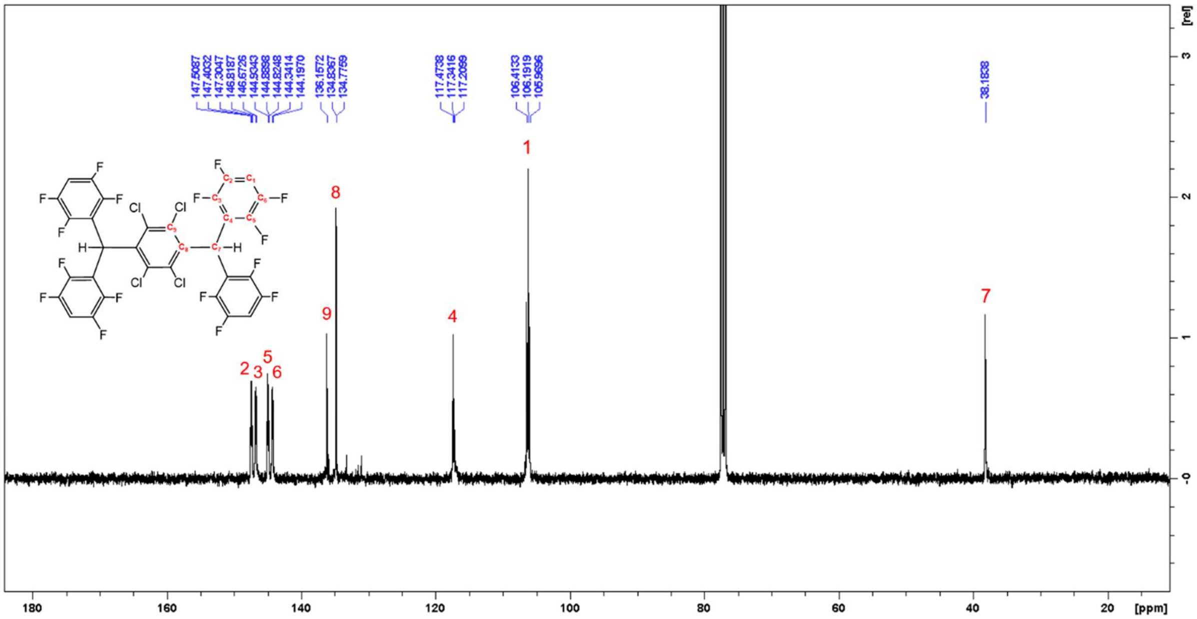

Figure S4: $^{13}C\{^1H\}$ NMR (101 MHz, $CDCl_3$) spectrum of **1-H**.

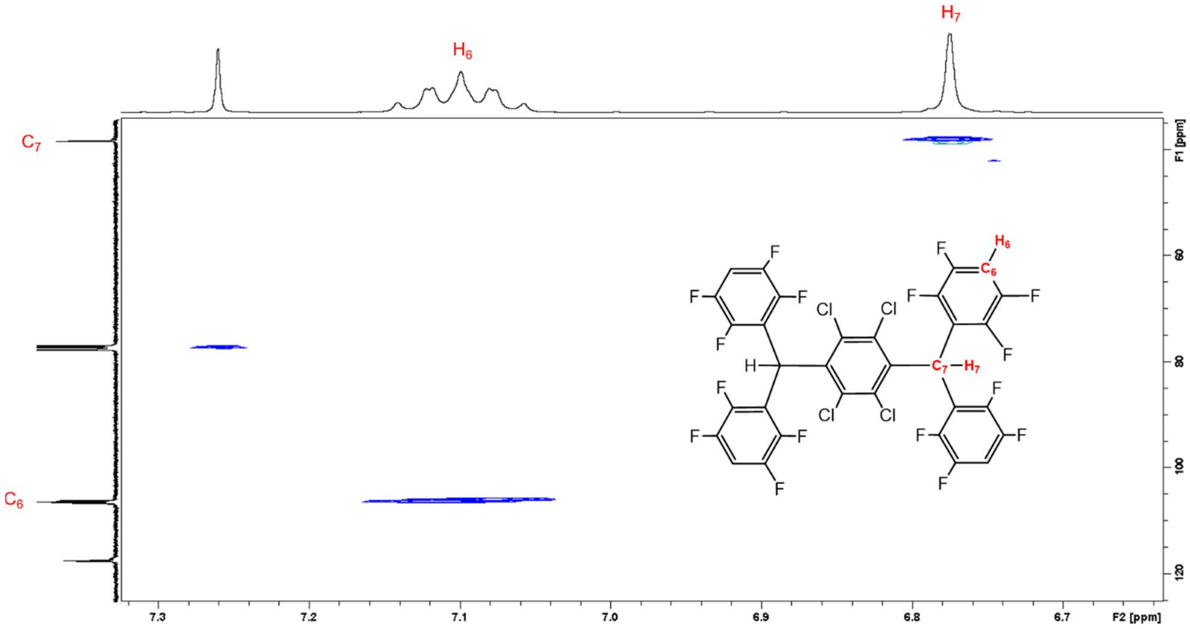

Figure S4: HSQC NMR ($CDCl_3$) spectrum of **1-H**.

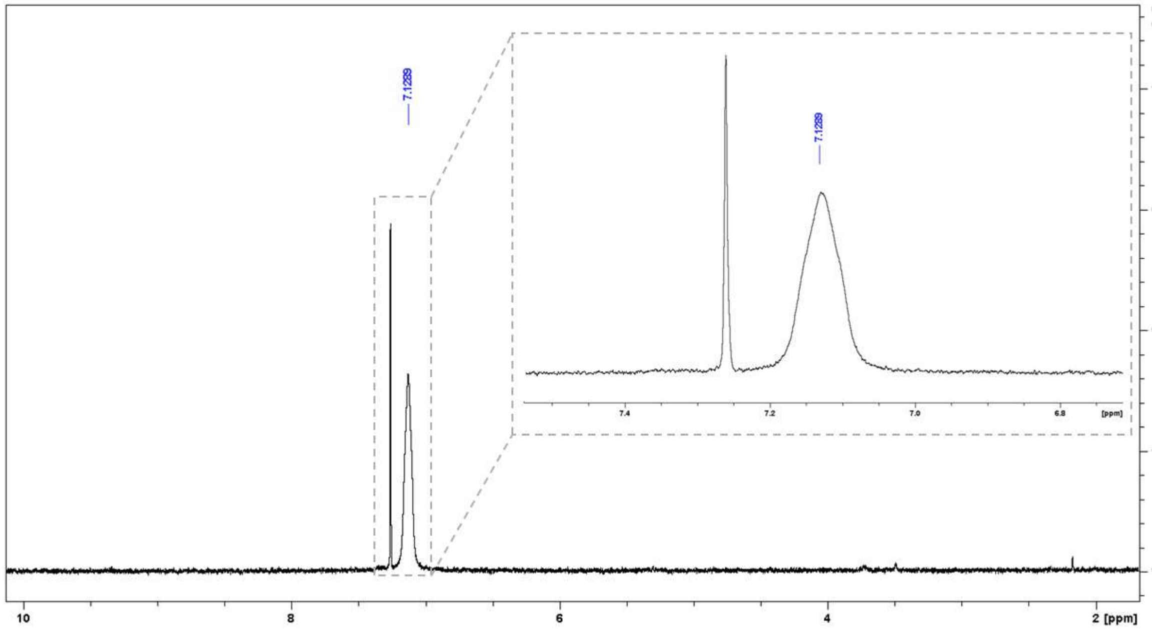

Figure S5: ^{1}H NMR (400 MHz, $CDCl_3$) spectrum of **1**.

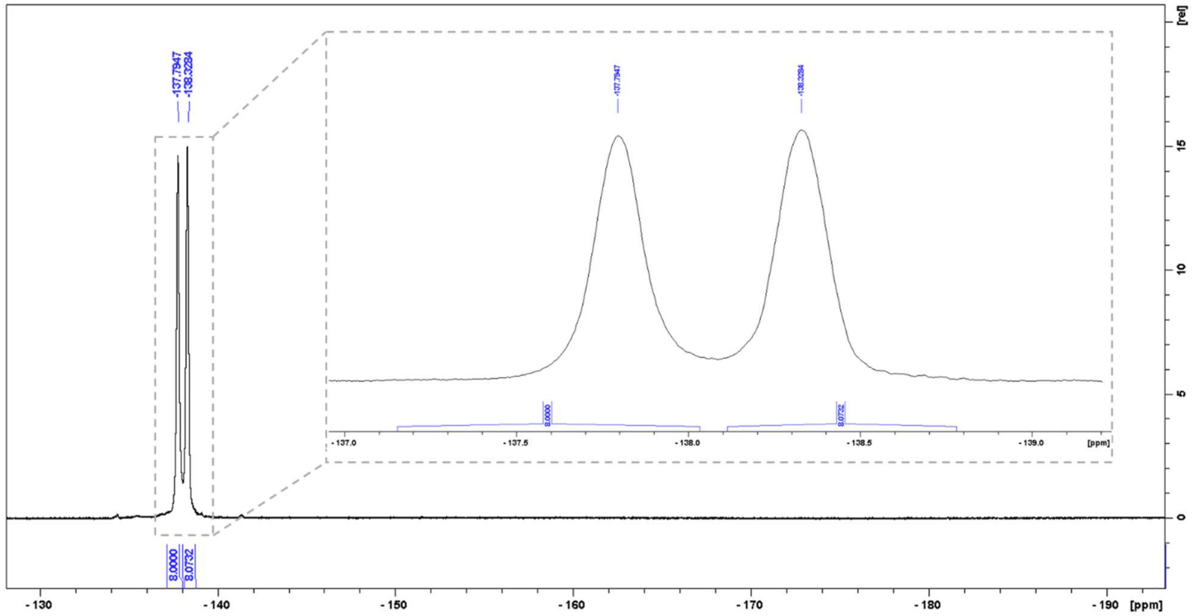

Figure S6: ^{19}F NMR (200 MHz, $CDCl_3$) spectrum of **1**.

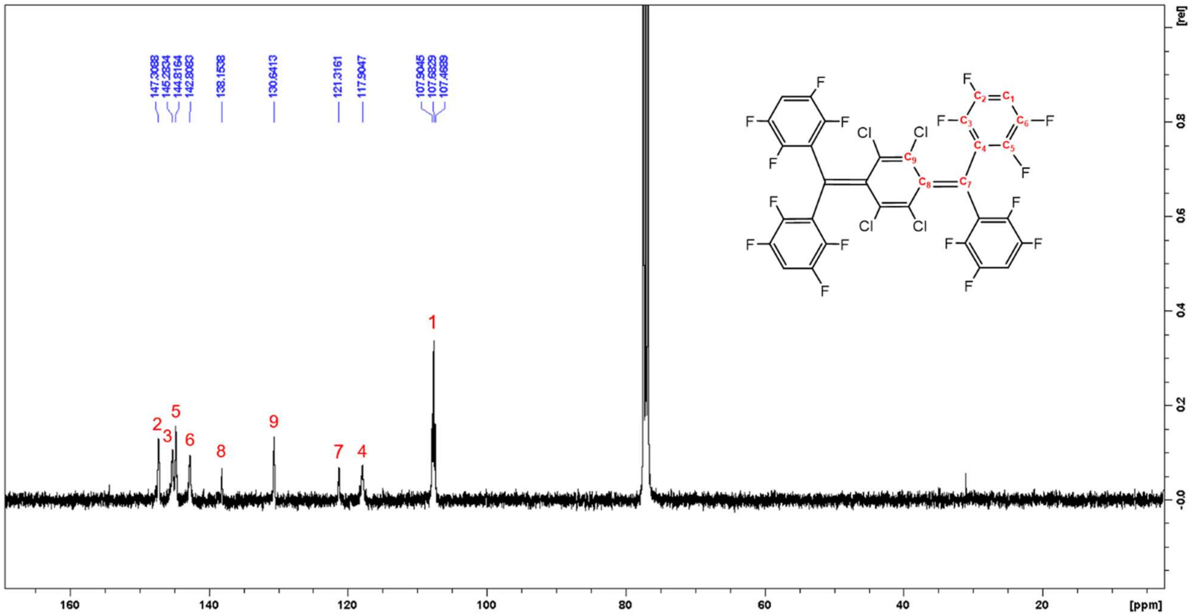

Figure S7: $^{13}C\{^1H\}$ NMR (101 MHz, $CDCl_3$) spectrum of **1.**

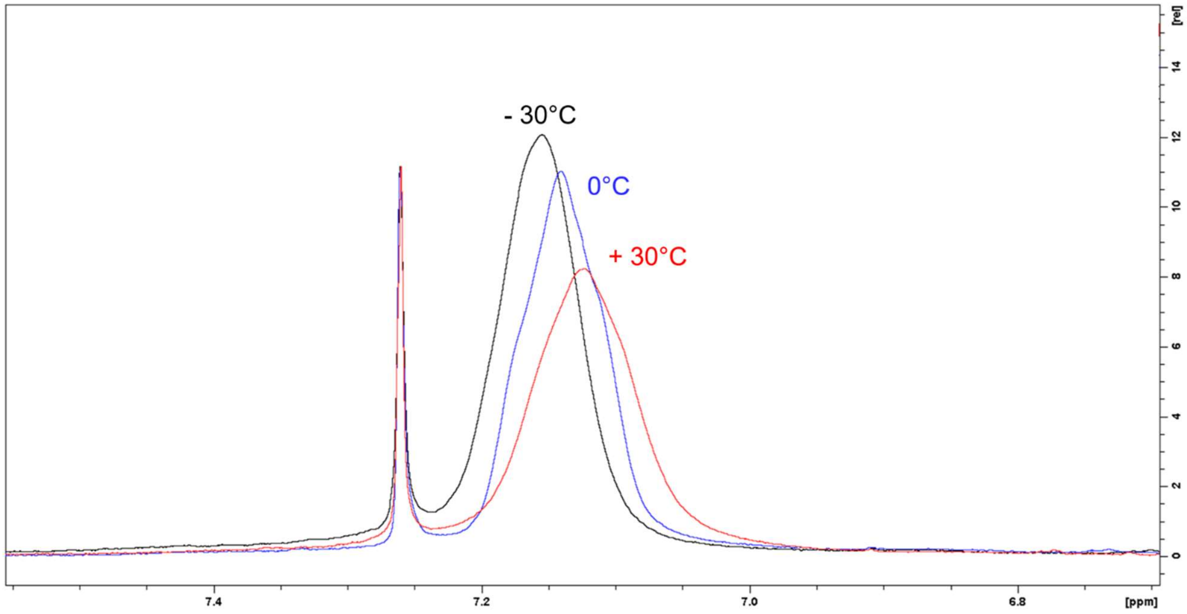

Figure S8: ^{1}H NMR (300 MHz, $CDCl_3$) spectrum of **1** at −30 °C (black), 0 °C (blue) and +30 °C (red).

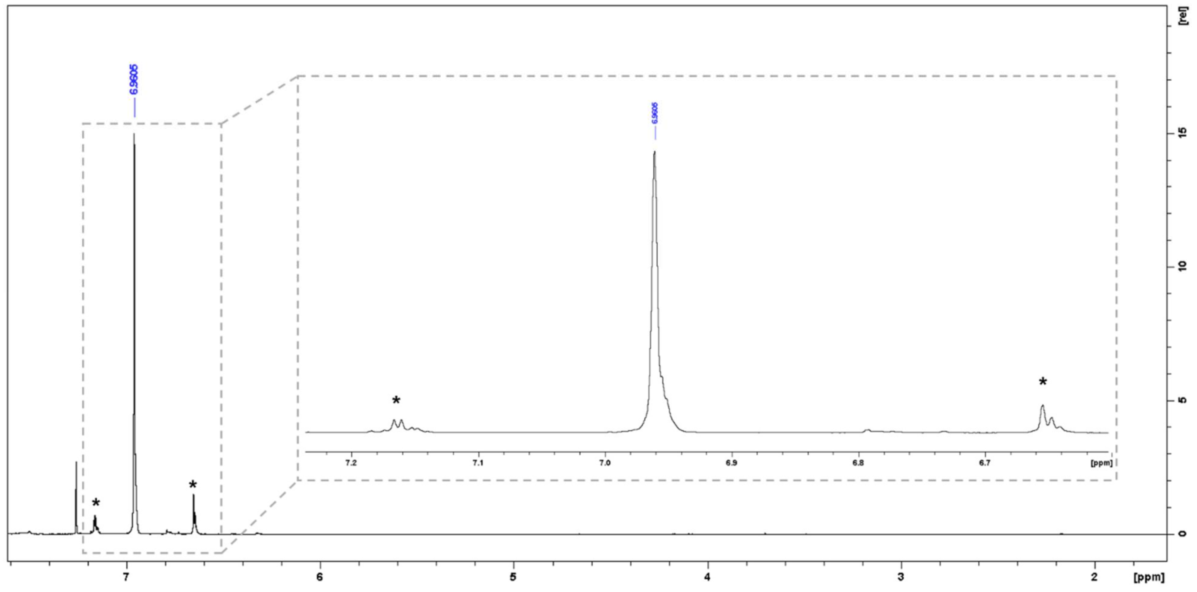

Figure S9: ^{1}H NMR (400 MHz, $CDCl_3$) spectrum of 1,2,4,5-tetrafluoro-3,6-bis(dichloromethyl)benzene. Unknown impurities are highlighted with *.

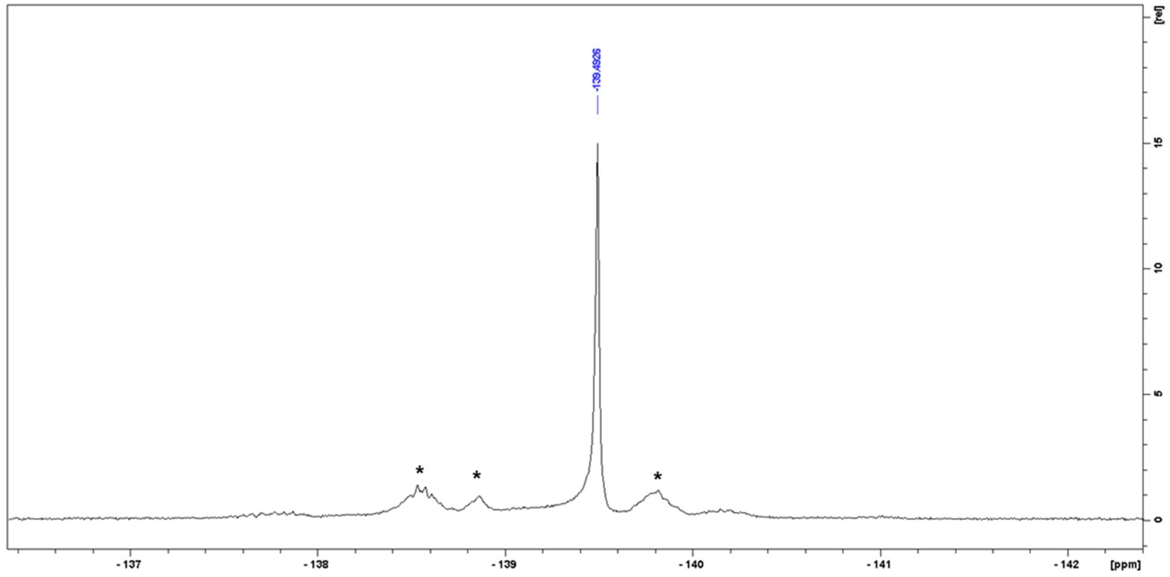

Figure S10: ^{19}F NMR (200 MHz, $CDCl_3$) spectrum of 1,2,4,5-tetrafluoro-3,6-bis(dichloro-methyl)benzene. Unknown impurities are highlighted with *.

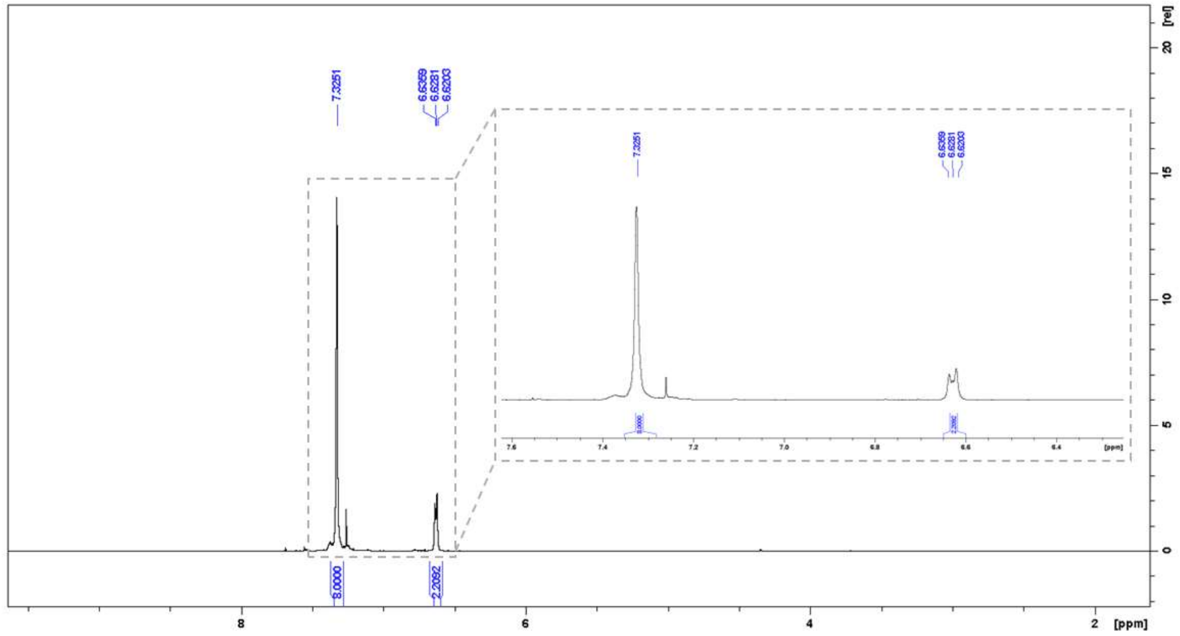

Figure S11: ^{1}H NMR (400 MHz, $CDCl_3$) spectrum of **2-H**.

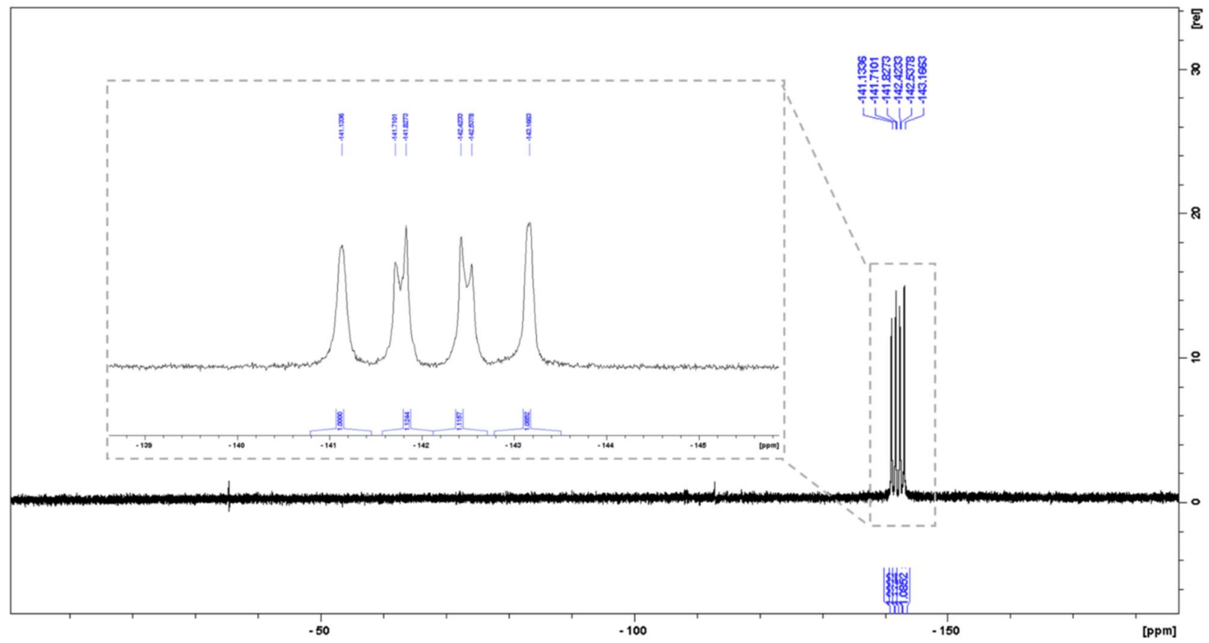

Figure S12: ^{19}F NMR (200 MHz, $CDCl_3$) spectrum of **2-H**.

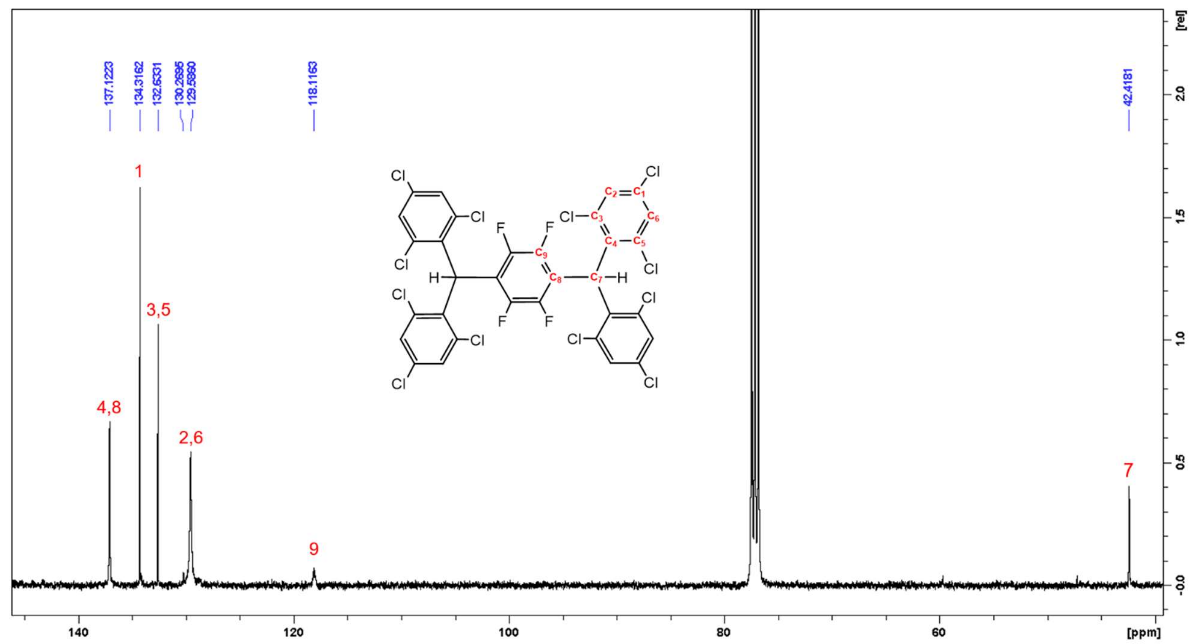

Figure S13: $^{13}C\{^1H\}$ NMR (101 MHz, $CDCl_3$) spectrum of **2-H**.

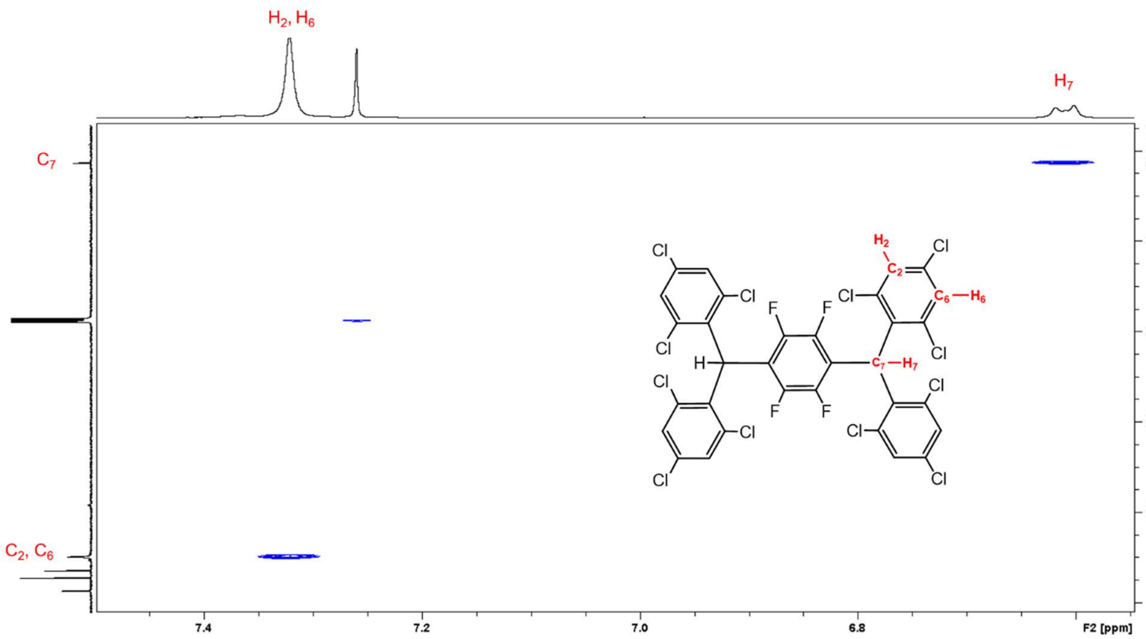

Figure S14: HSQC NMR ($CDCl_3$) spectrum of **2-H**.

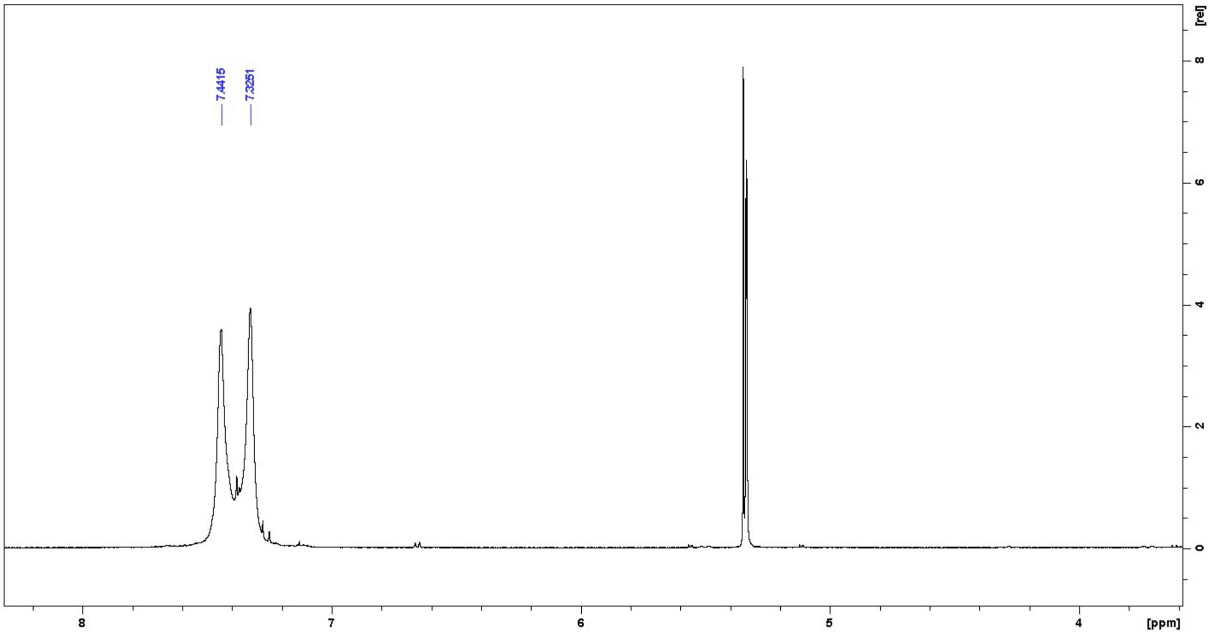

Figure S15: ^{1}H NMR (400 MHz, CD_2Cl_2) spectrum of **2**

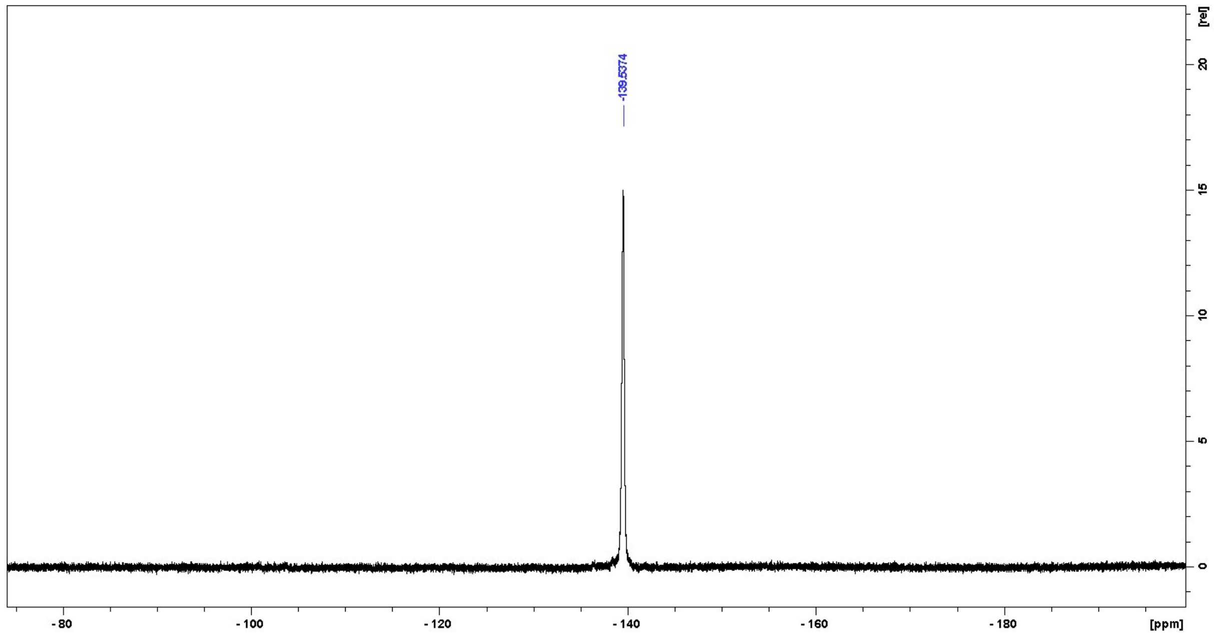

Figure S16: ^{19}F NMR (200 MHz, CD_2Cl_2) spectrum of **2**.

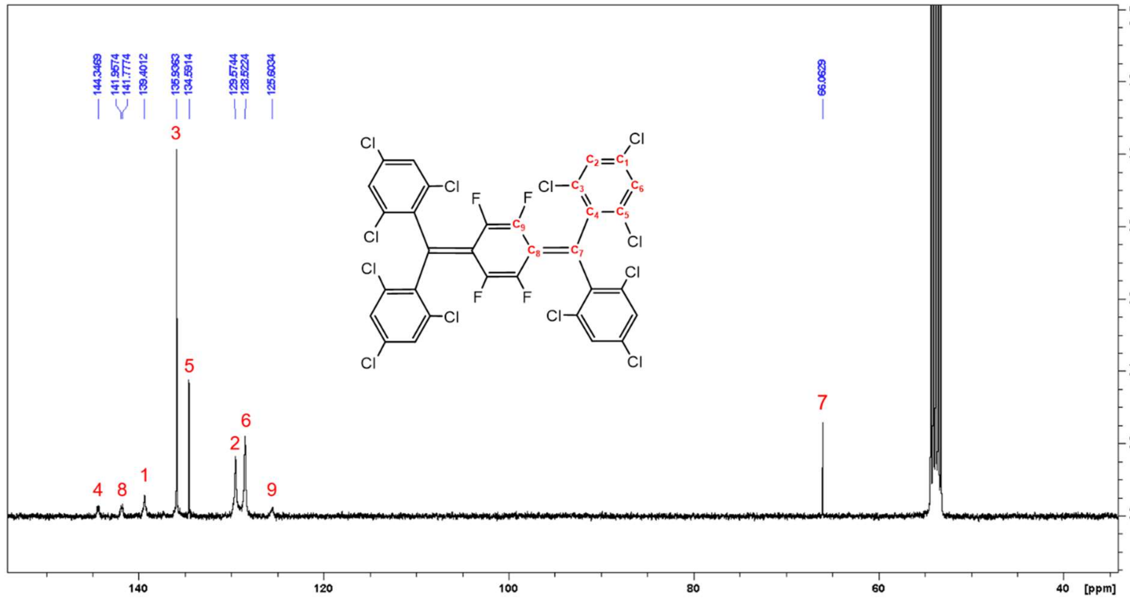

Figure S17: $^{13}C\{^1H\}$ NMR (101 MHz, $CDCl_3$) spectrum of **2**.

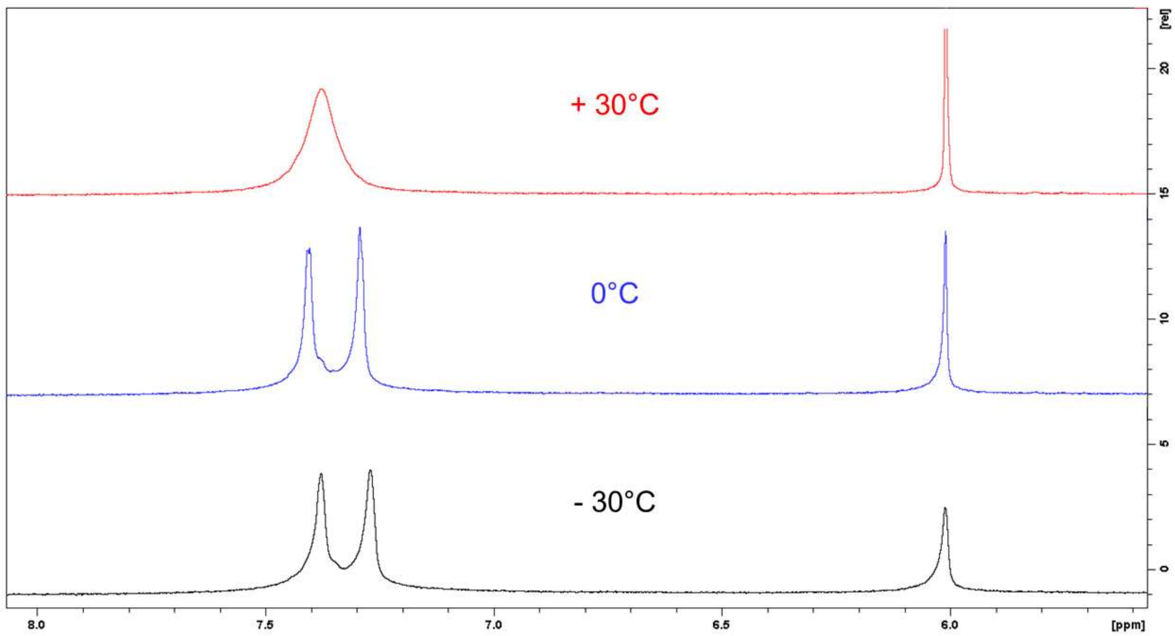

Figure S18: ^{1}H NMR (300 MHz, $C_2D_2Cl_4$) spectrum of **2** at –30 °C (black), 0 °C (blue) and +30 °C (red).

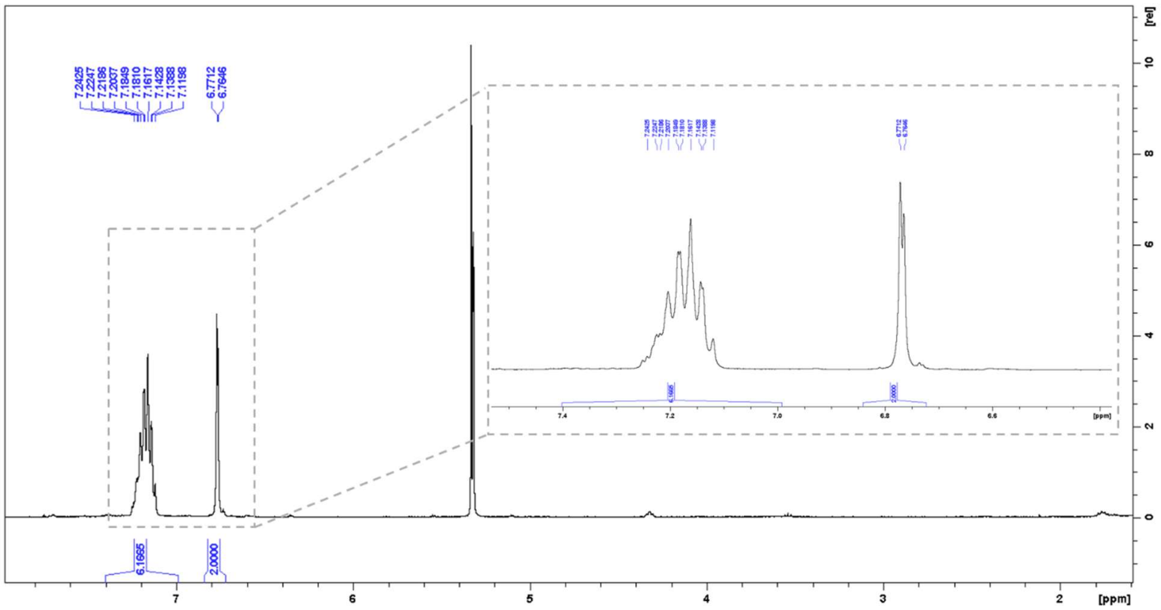

Figure S19: ^{1}H NMR (400 MHz, CD_2Cl_2) spectrum of **3**.

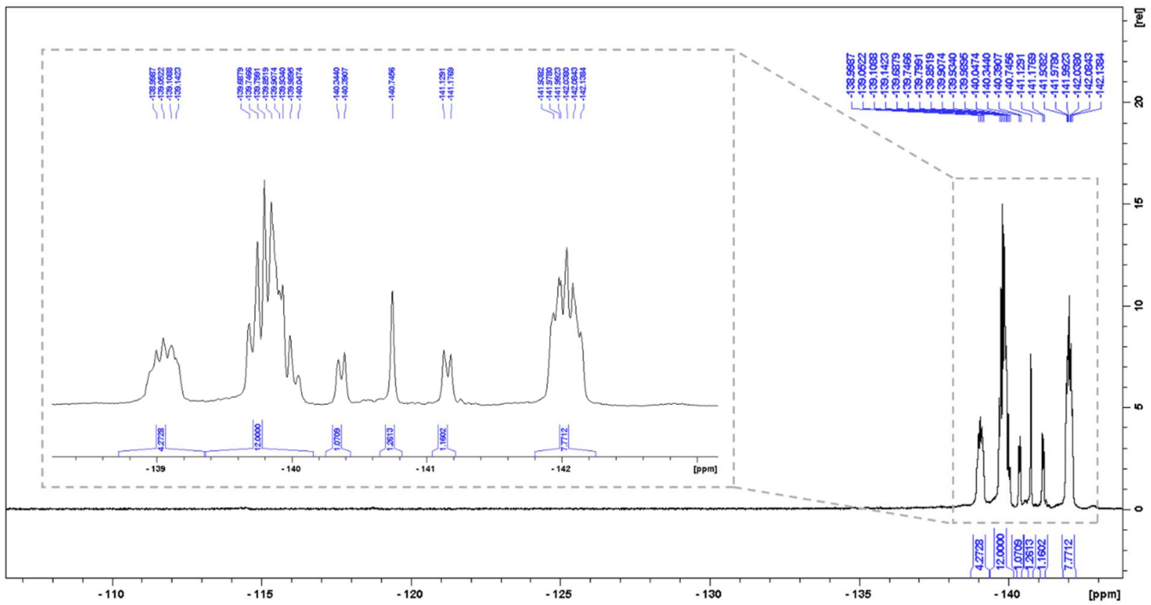

Figure S20: ^{19}F NMR (200 MHz, CD_2Cl_2) spectrum of **3**.

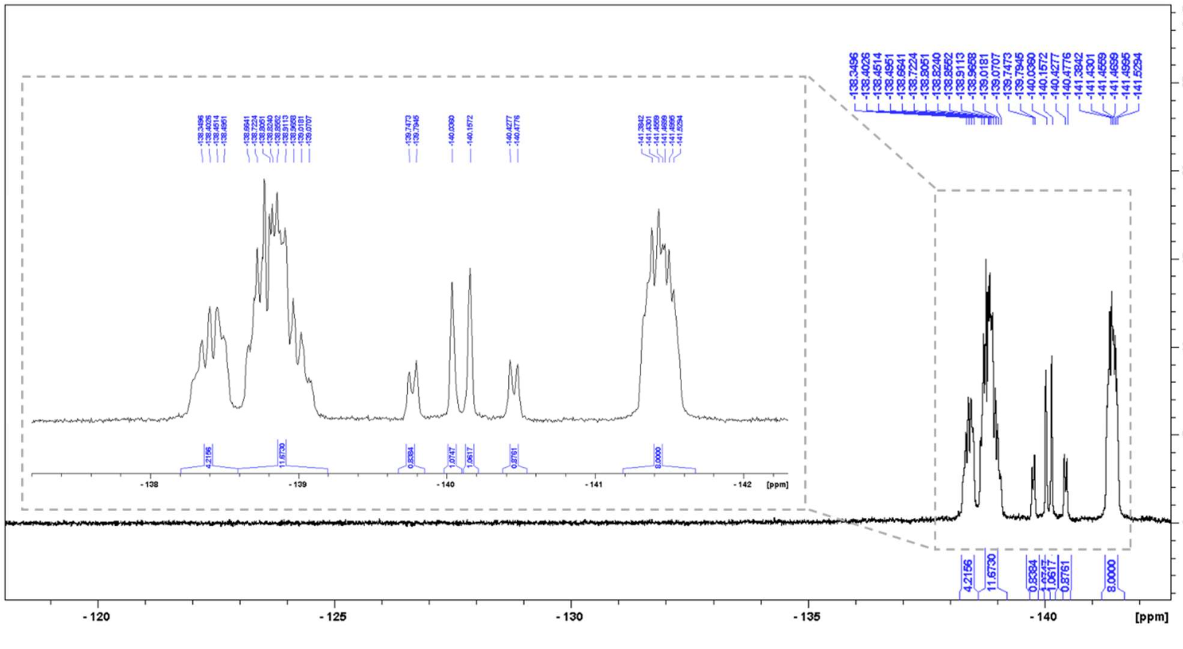

Figure S21: ^{19}F NMR (200 MHz, $CDCl_3$) spectrum of **3**.

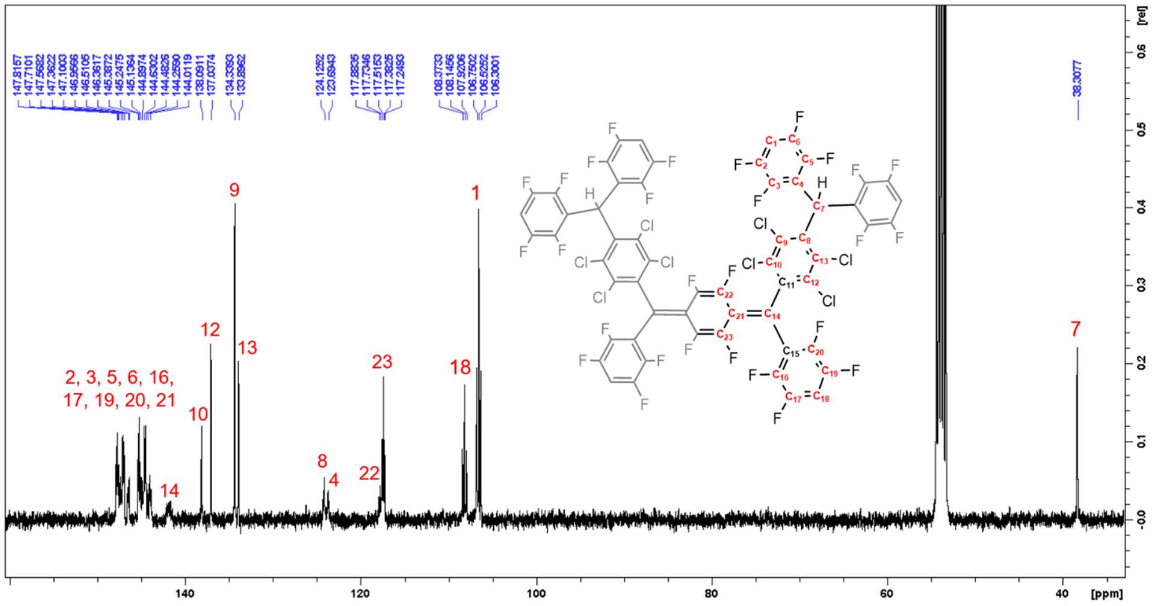

Figure S22: $^{13}C\ \{^1H\}$ NMR (101 MHz, CD_2Cl_2) spectrum of **3**.

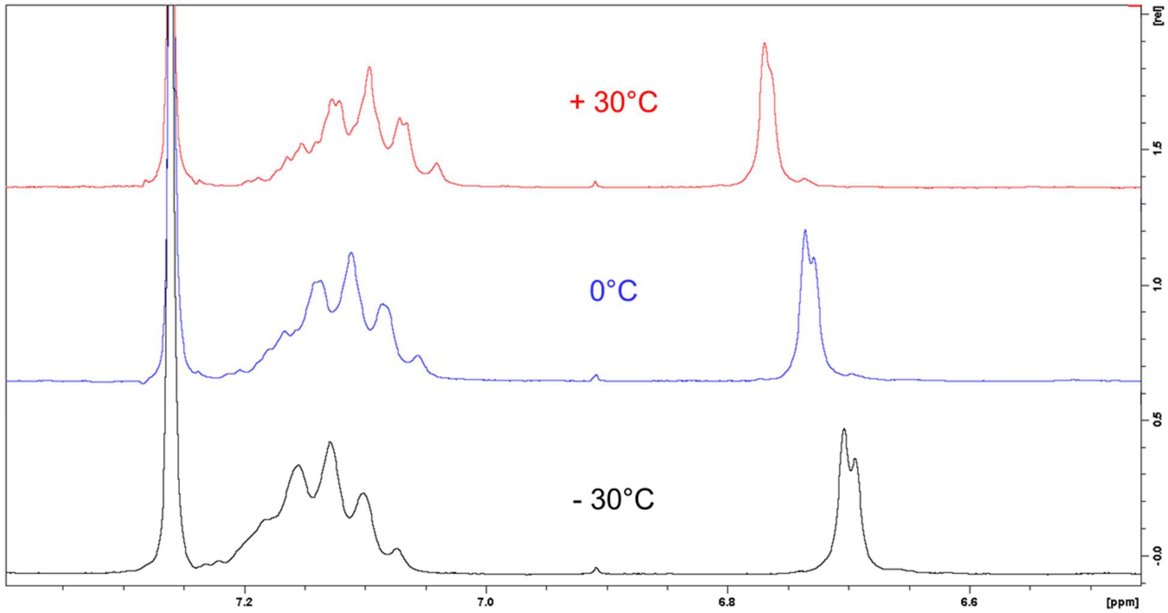

Figure S23: ^1H NMR (300 MHz, $CDCl_3$) spectrum of **3** at −30 °C (black), 0 °C (blue) and +30 °C (red).

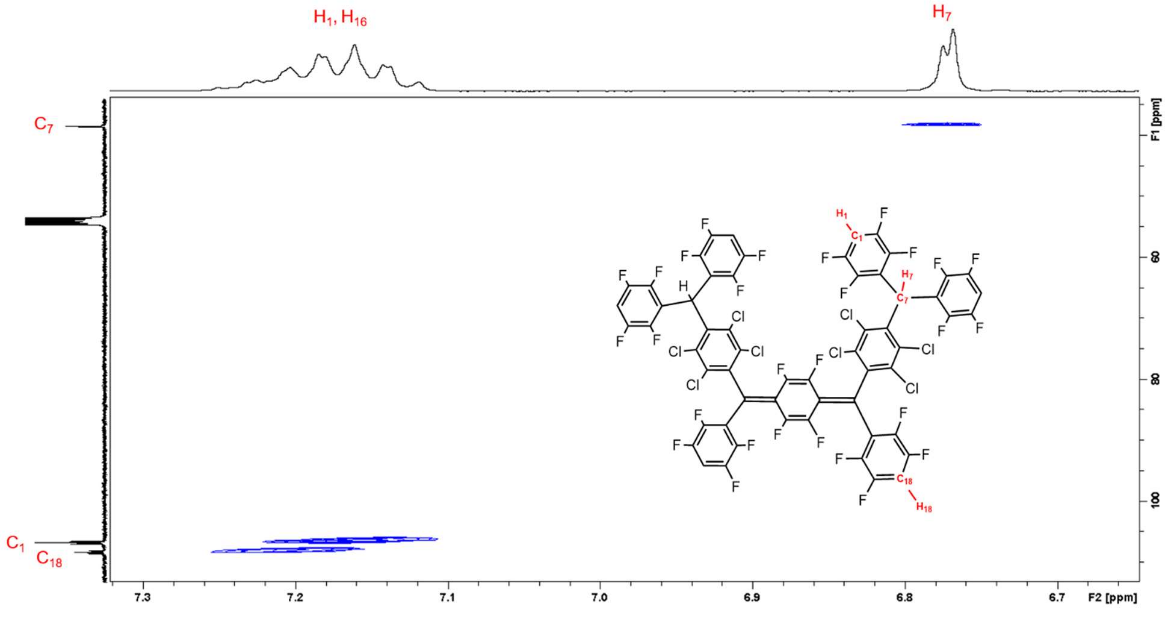

Figure S24: HSQC NMR (CD_2Cl_2) spectrum of **3**.

4. UV-Vis electronic absorption spectra

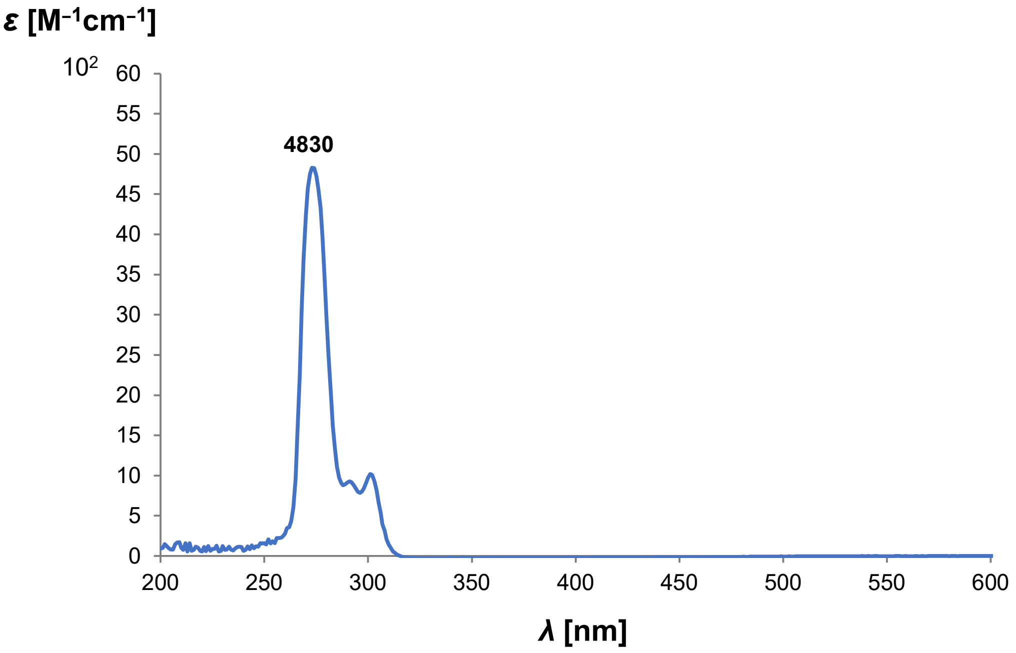

Figure S25: UV-Vis spectrum of **1-H** dissolved in $CHCl_3$, $c = 1.7 \times 10^{-3}$ M.

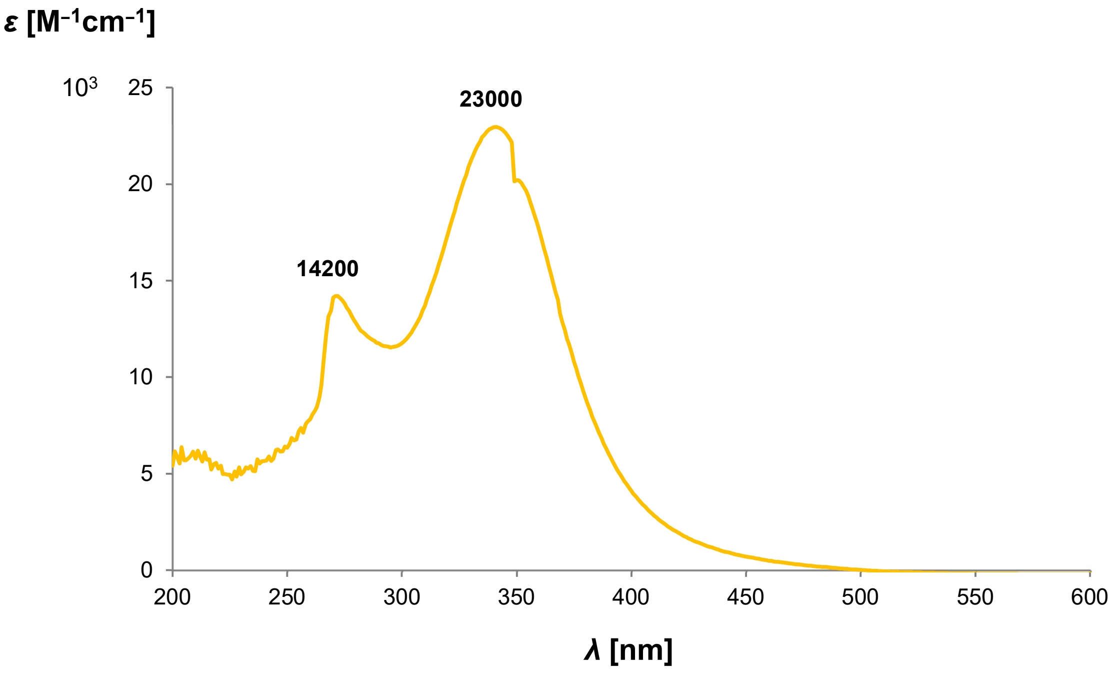

Figure S26: UV-Vis spectrum of **1** dissolved in $CHCl_3$, $c = 3.4 \times 10^{-5}$ M.

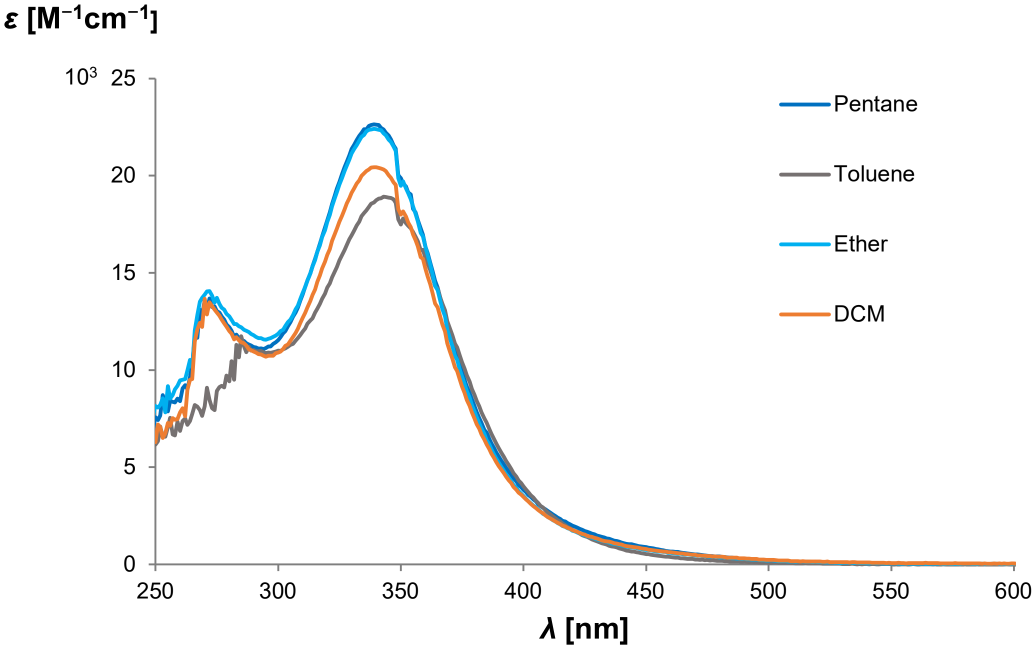

Figure S27: UV-Vis spectra of **1** dissolved in *n*-pentane, toluene, Et_2O and DCM, $c = 1.3 \times 10^{-5}$ M.

Table S1: Absorption bands of **1** dissolved in *n*-pentane, toluene, Et_2O and DCM, $c = 1.3 \times 10^{-5}$ M.

Pentane	ε (339 nm)	22600	$M^{-1} cm^{-1}$
	ε (272 nm)	13700	$M^{-1} cm^{-1}$
Toluene	ε (343 nm)	18900	$M^{-1} cm^{-1}$
	ε (271 nm)	11700	$M^{-1} cm^{-1}$
Ether	ε (339 nm)	22400	$M^{-1} cm^{-1}$
	ε (272 nm)	14000	$M^{-1} cm^{-1}$
DCM	ε (341 nm)	20400	$M^{-1} cm^{-1}$
	ε (270 nm)	13700	$M^{-1} cm^{-1}$

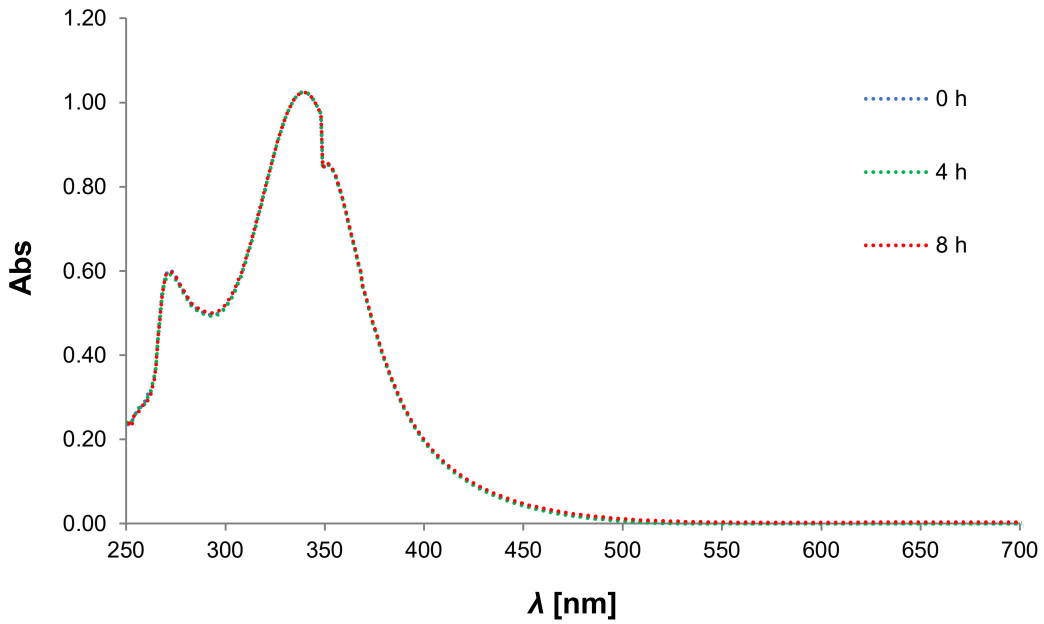

Figure S28: Superimposed UV-Vis spectra of **1** dissolved in *n*-pentane after 0 h, 4 h and 8 h of irradiation at 365 nm.

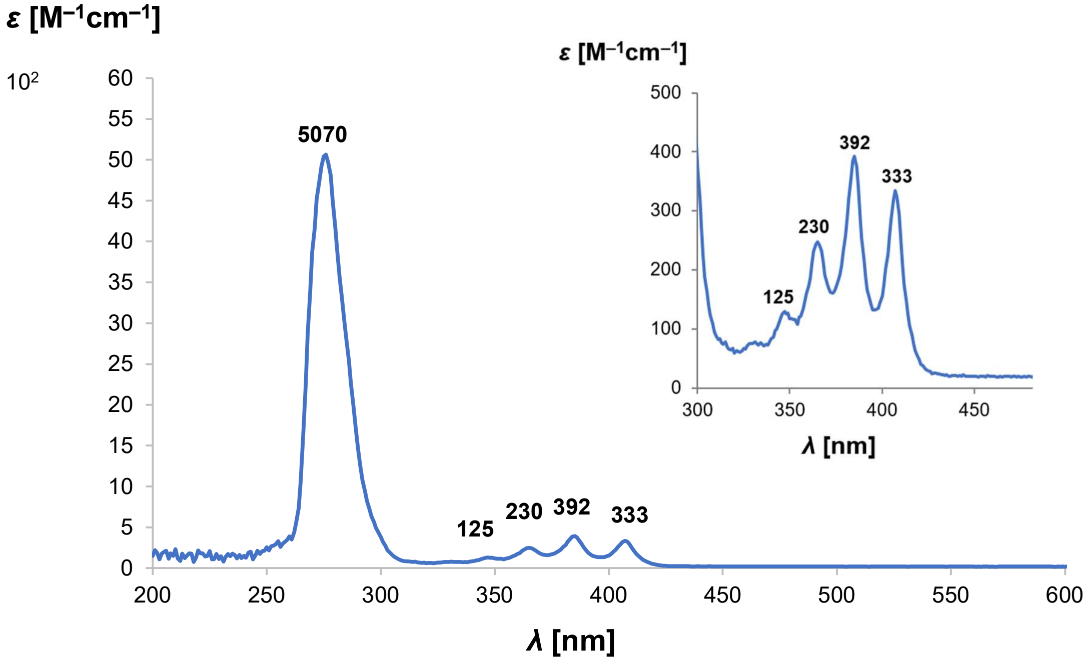

Figure S29: UV-Vis spectrum of **2-H** dissolved in *n*-pentane, $c = 1.3 \times 10^{-4}$ M.

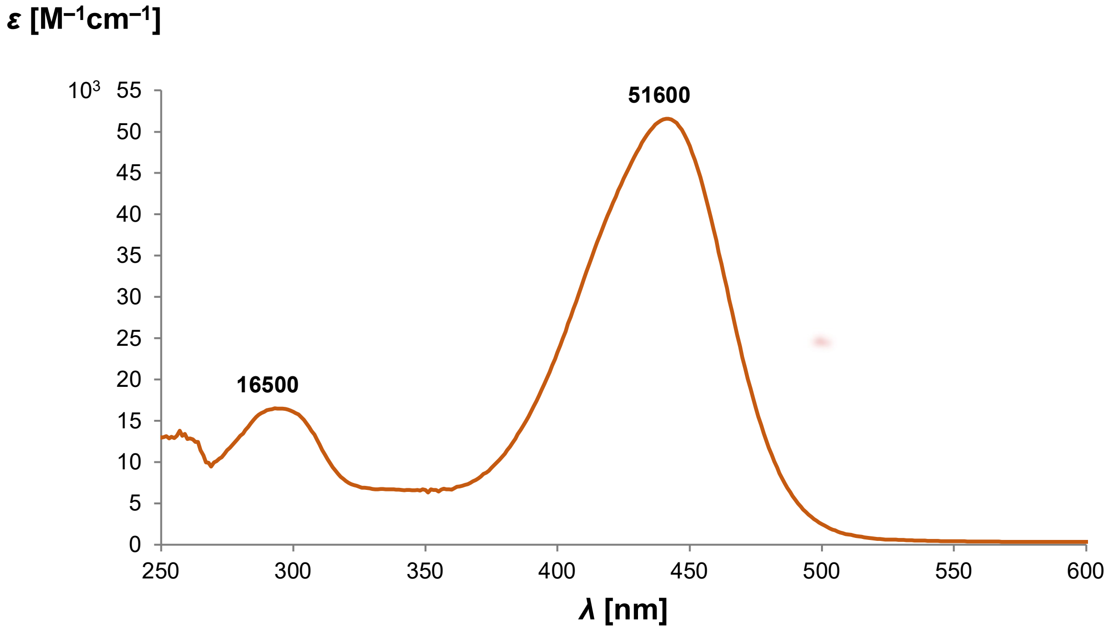

Figure S30: UV-Vis spectrum of **2** dissolved in $CHCl_3$, $c = 1.3 \times 10^{-5}$ M.

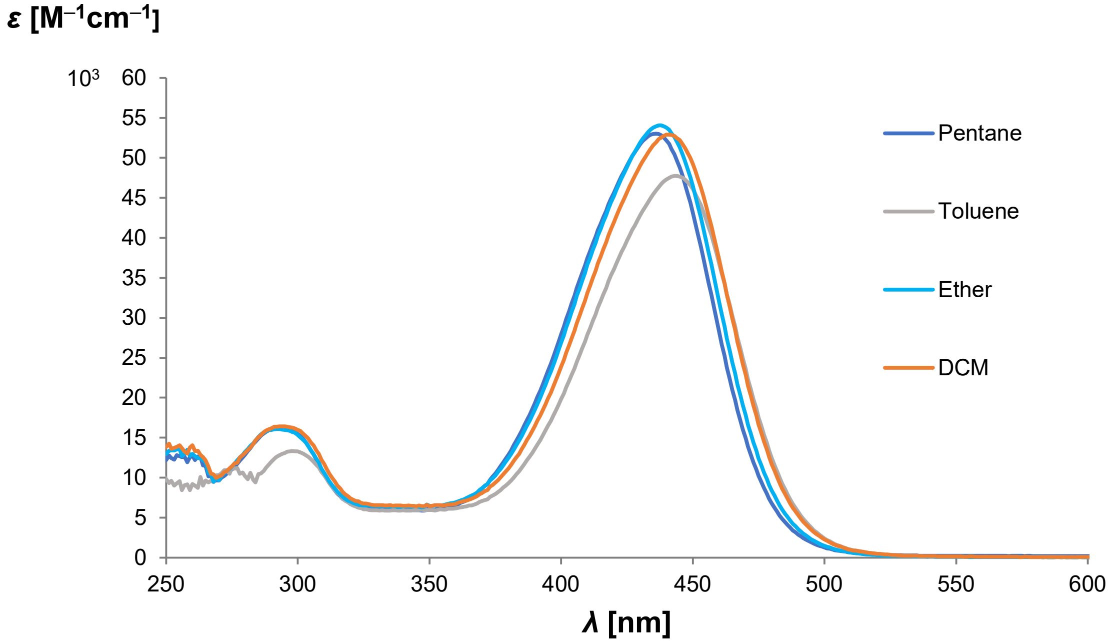

Figure S31: Superimposed UV-Vis spectra of **2** dissolved in *n*-pentane, toluene, Et_2O and DCM, $c = 1.3 \times 10^{-5}$ M.

Table S2: UV-Vis data related to **2** dissolved in *n*-pentane, toluene, Et_2O and DCM, *c* = 1.3 × 10^{-5} M.

Pentane	ε (436 nm)	53000	M^{-1} cm^{-1}
	ε (293 nm)	16200	M^{-1} cm^{-1}
Toluene	ε (443 nm)	47700	M^{-1} cm^{-1}
	ε (293 nm)	13300	M^{-1} cm^{-1}
Ether	ε (438 nm)	54000	M^{-1} cm^{-1}
	ε (292 nm)	16100	M^{-1} cm^{-1}
DCM	ε (441 nm)	52900	M^{-1} cm^{-1}
	ε (293 nm)	16400	M^{-1} cm^{-1}

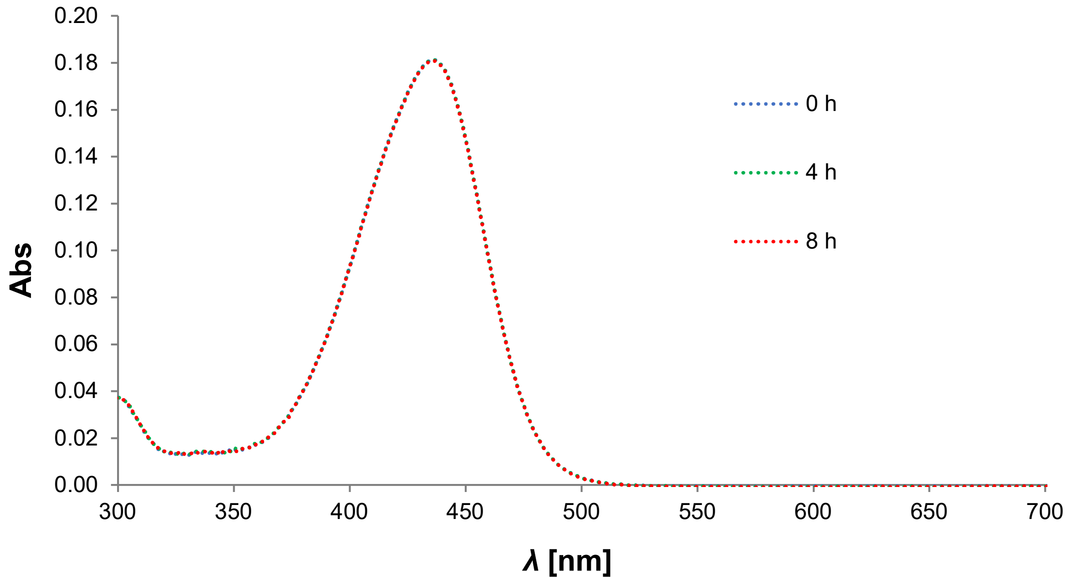

Figure S32: Superimposed UV-Vis spectra of **2** dissolved in *n*-pentane after 0 h, 4 h and 8 h of irradiation at 365 nm.

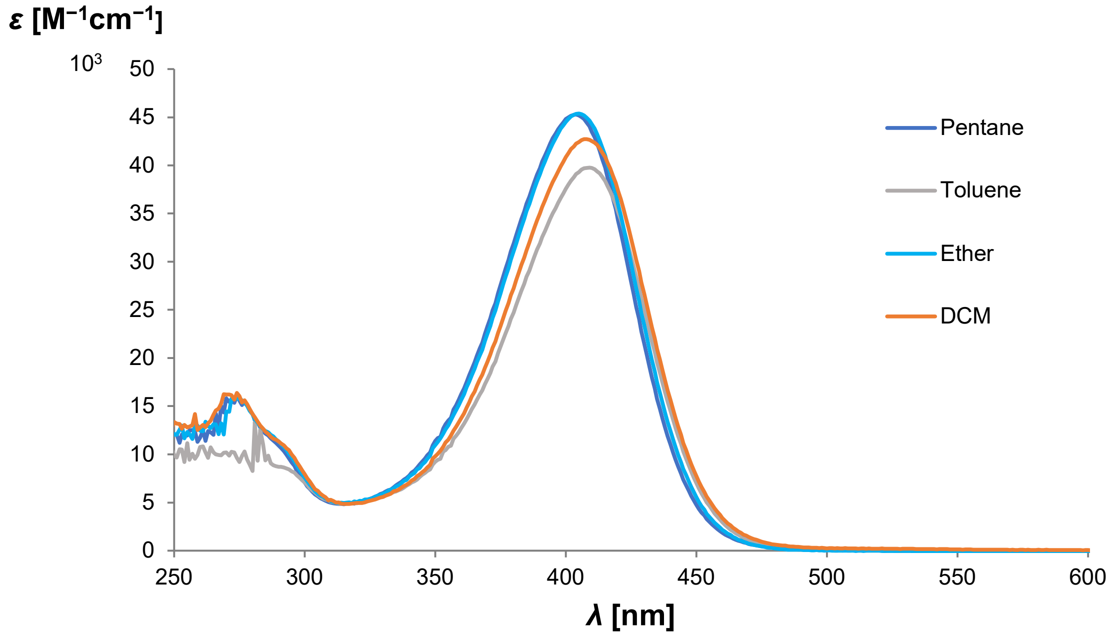

Figure S33: UV-Vis spectra of **3** dissolved in *n*-pentane, toluene, Et_2O and DCM, $c = 1.0 \times 10^{-5}$ M.

Table S3: Absorption bands of **3** dissolved in *n*-pentane, toluene, Et_2O and DCM, $c = 1.0 \times 10^{-5}$ M.

Pentane	ε (404 nm)	45300	M^{-1} cm^{-1}
	ε (274 nm)	16100	M^{-1} cm^{-1}
Toluene	ε (409 nm)	39800	M^{-1} cm^{-1}
Ether	ε (404 nm)	45600	M^{-1} cm^{-1}
	ε (274 nm)	15900	M^{-1} cm^{-1}
DCM	ε (408 nm)	42700	M^{-1} cm^{-1}
	ε (274 nm)	16400	M^{-1} cm^{-1}

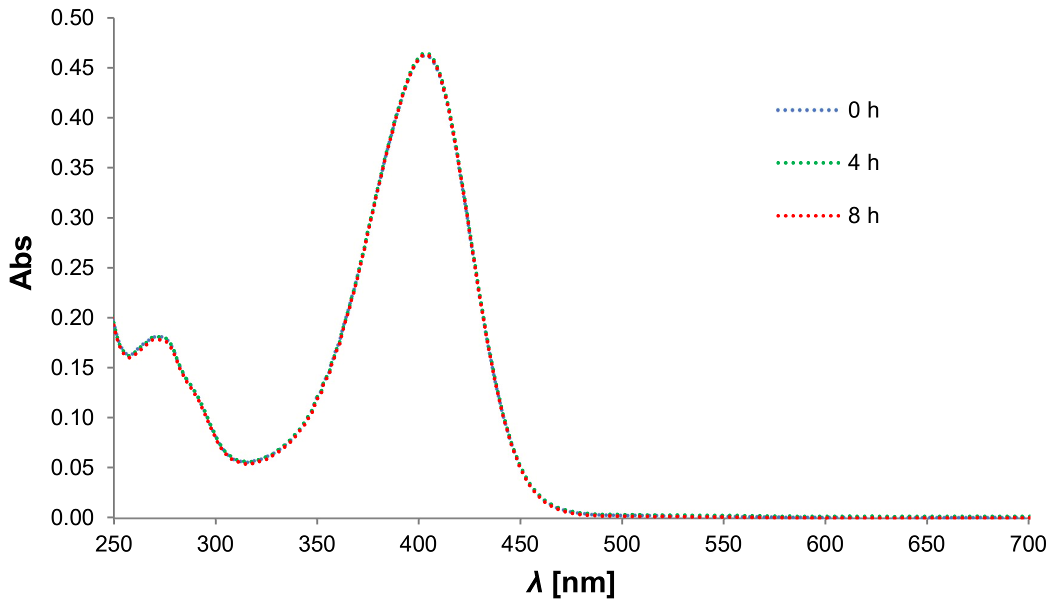

Figure S34: Superimposed UV-Vis spectra of **3** dissolved in *n*-pentane after 0 h, 4 h and 8 h of irradiation at 365 nm.

5. Emission and excitation spectra and lifetime measurements

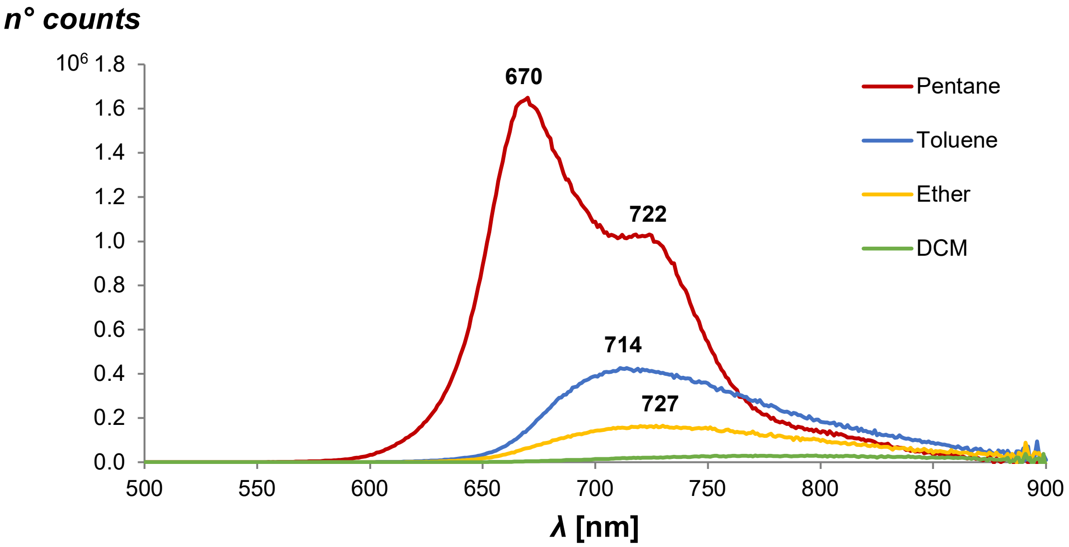

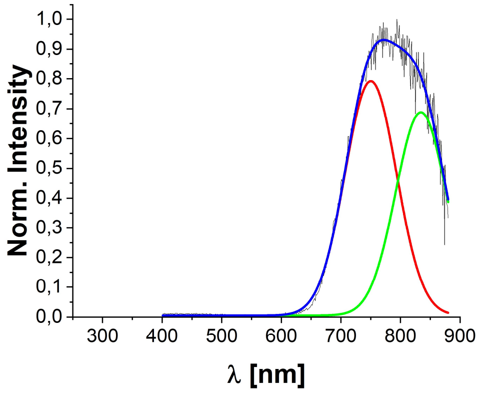

Figure S35: Top, superimposed emission spectra of **1** dissolved in *n*-pentane, toluene, Et_2O, DCM, $c = 4.4 \times 10^{-6}$ M. Bottom, fitting of the emission data for compound **1** in DCM to better estimate the maximum (772 nm). The experimental curve was deconvoluted with two gaussians ($R^2 = 0.99$).

Table S4: Emission data related to **1** dissolved in *n*-pentane, toluene and Et_2O, $c = 4.4 \times 10^{-6}$ M.

Solvent	λ_{em} [nm]	*PLQY* [%]	τ [ns]
Pentane	670<br>722	100	81
Toluene	714	13	13
Ether	727	3	5
DCM	772	< 1	-

λ_{exc}	340 nm
slits	2-1.7 nm

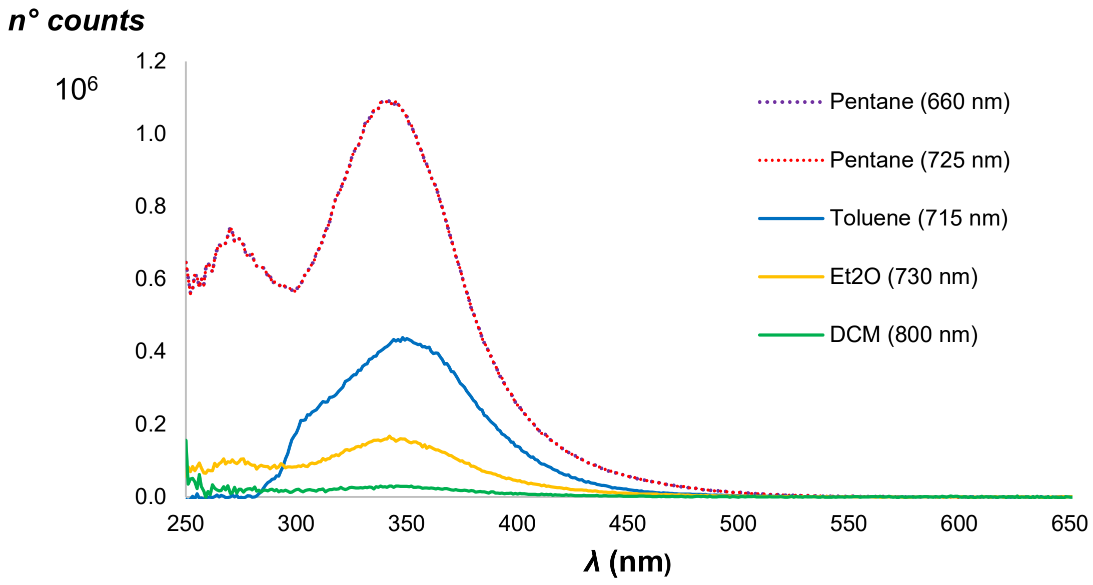

Figure S36: Superimposed excitation spectra of **1** dissolved in *n*-pentane, toluene, Et_2O, DCM, $c = 4.4 \times 10^{-6}$ M.

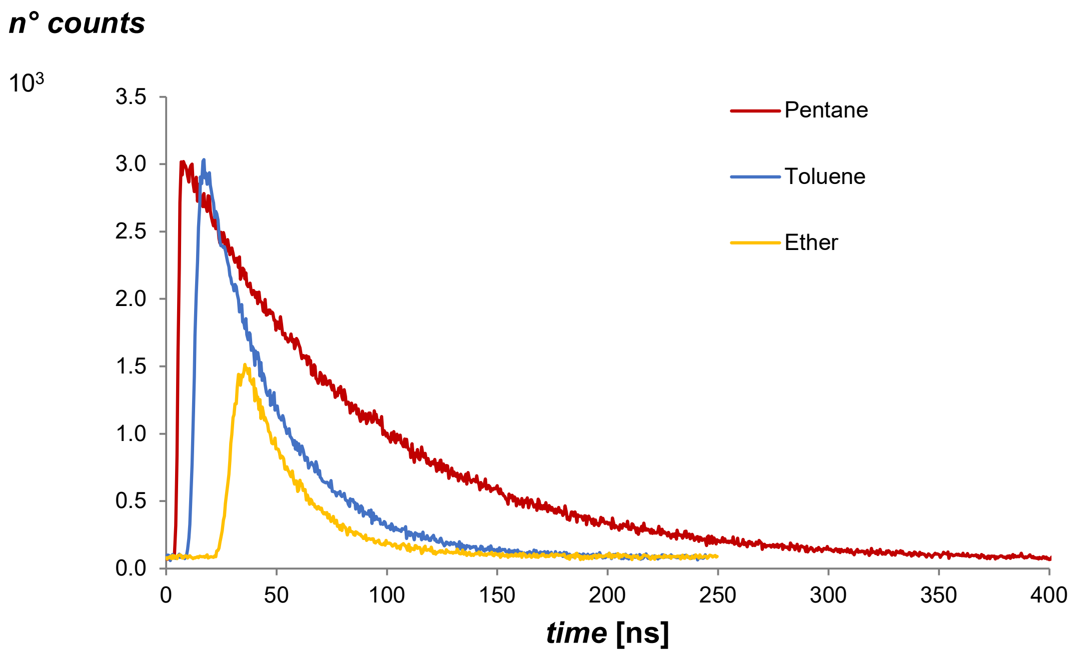

Figure S37: Superimposed decay times of **1** dissolved in *n*-pentane, toluene and Et_2O, $c = 4.4 \times 10^{-6}$ M.

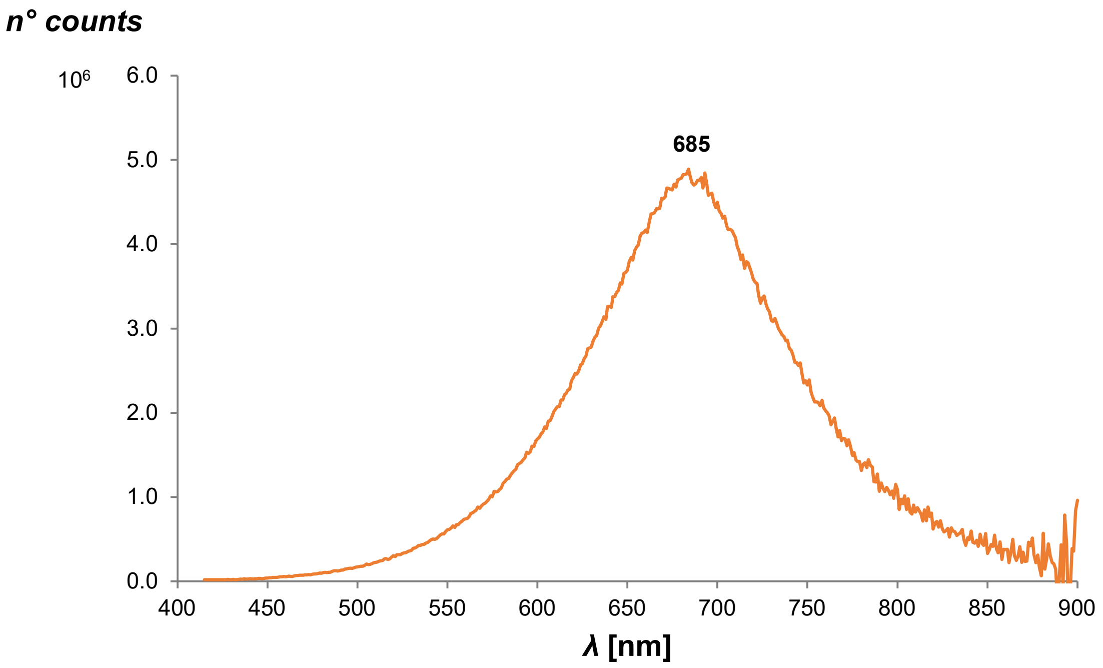

Figure S38: Emission spectrum of **1** in solid state, dispersed in $BaSO_4$, λ_{exc}: 340 nm.

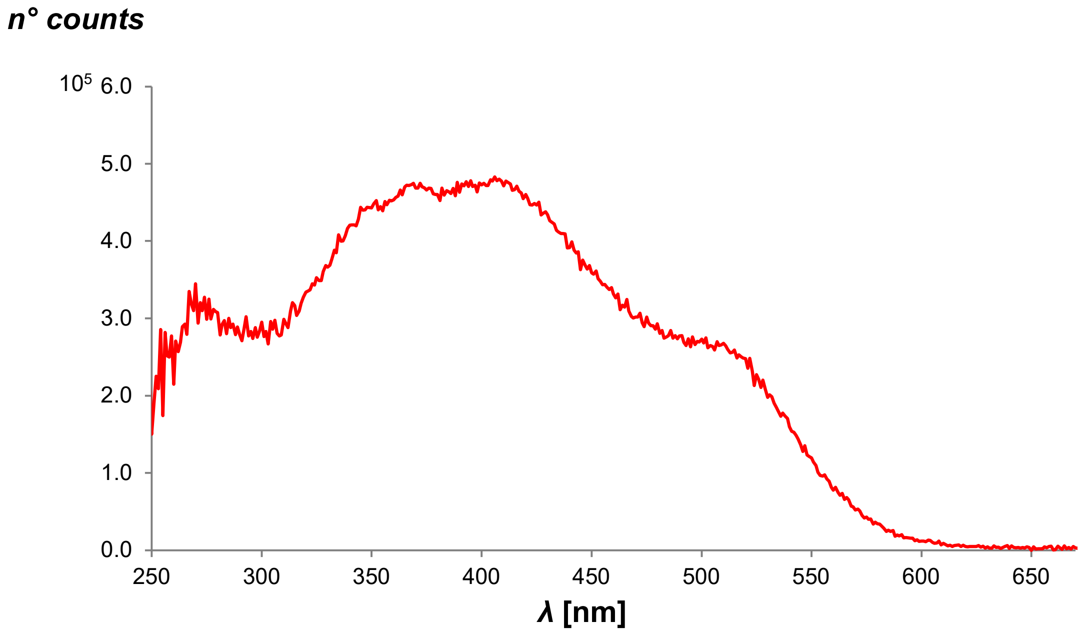

Figure S39: Excitation spectrum of **1** in the solid state, dispersed in $BaSO_4$, λ_{em}: 685 nm.

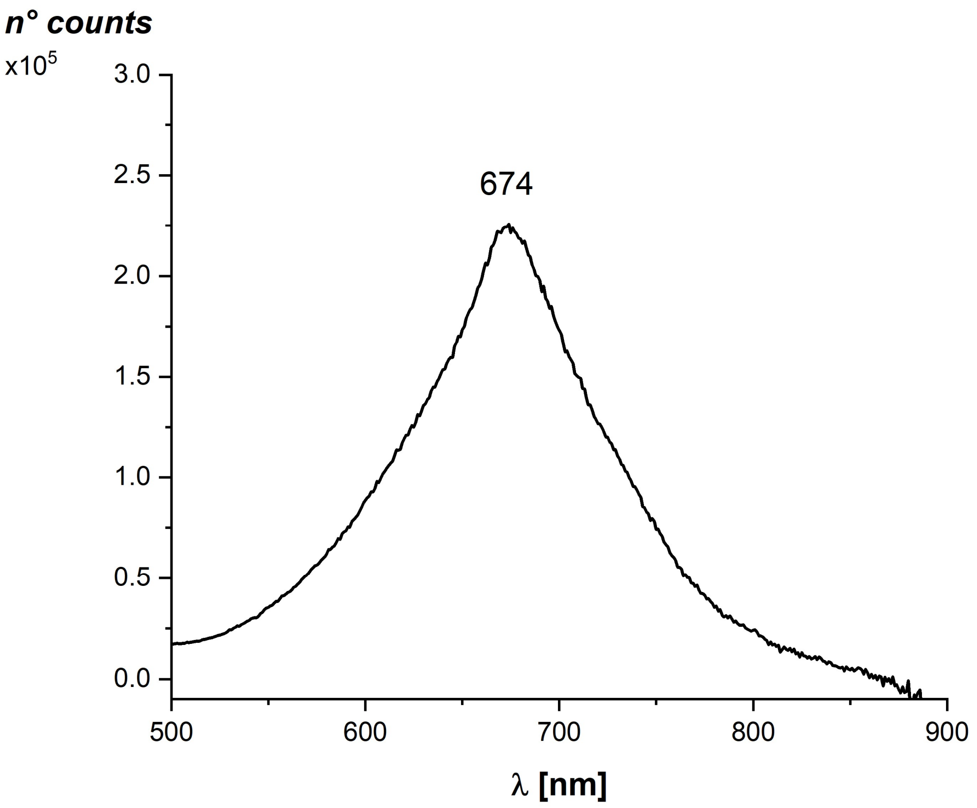

Figure S40: Emission spectrum of **1** in *n*-pentane matrix at 77 K, λ_{exc}: 375 nm.

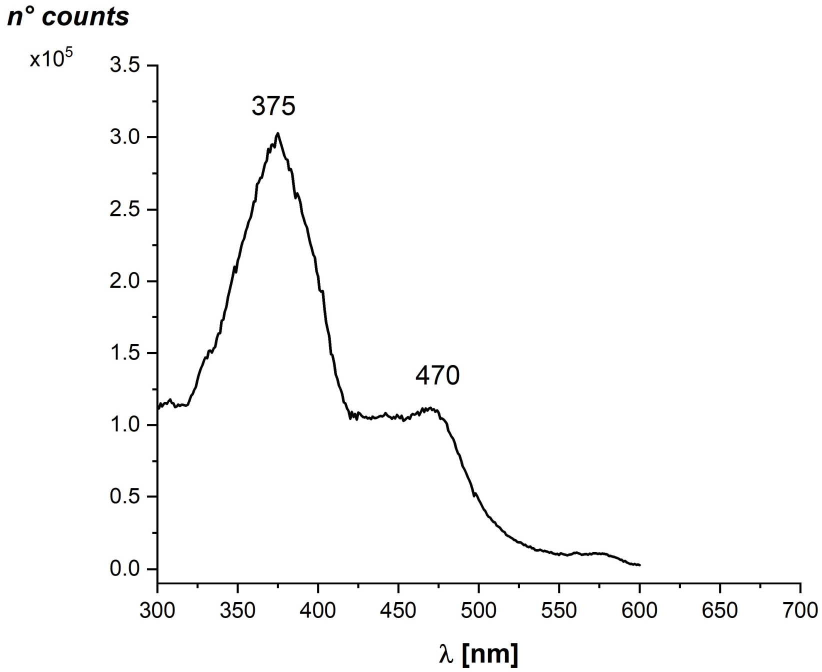

Figure S41: Excitation spectrum of **1** in *n*-pentane matrix at 77 K, λ_{em}: 670 nm.

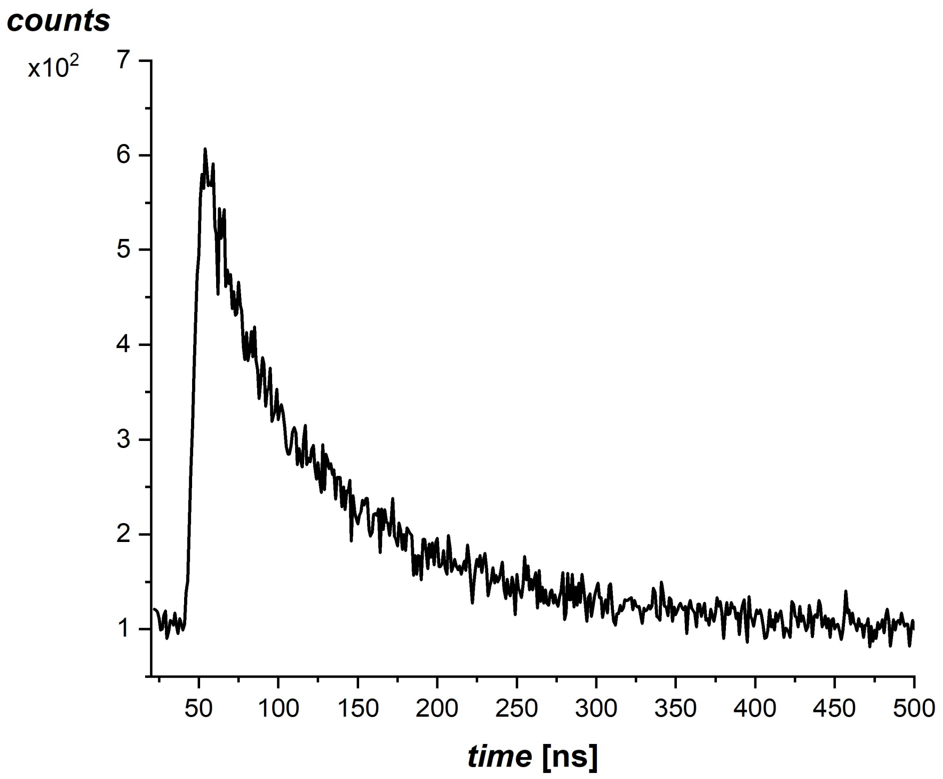

Figure S42: Decay times of **1** dissolved in *n*-pentane at 77 K, $c = 4.4 \times 10^{-6}$ M.

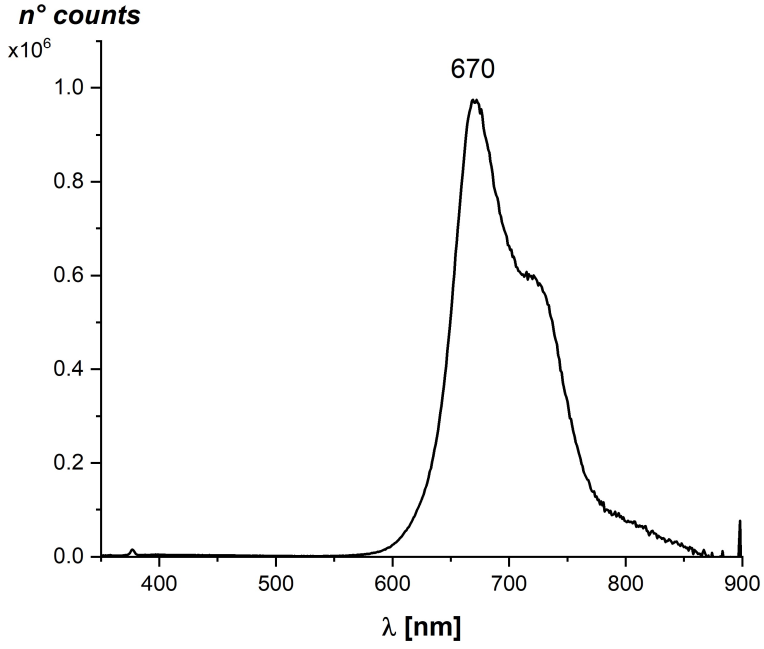

Figure S43. Emission spectrum of **1** dissolved in 3-methylpentane at room temperature, $c = 4.4 \times 10^{-6}$ M, λ_{exc}: 340 nm.

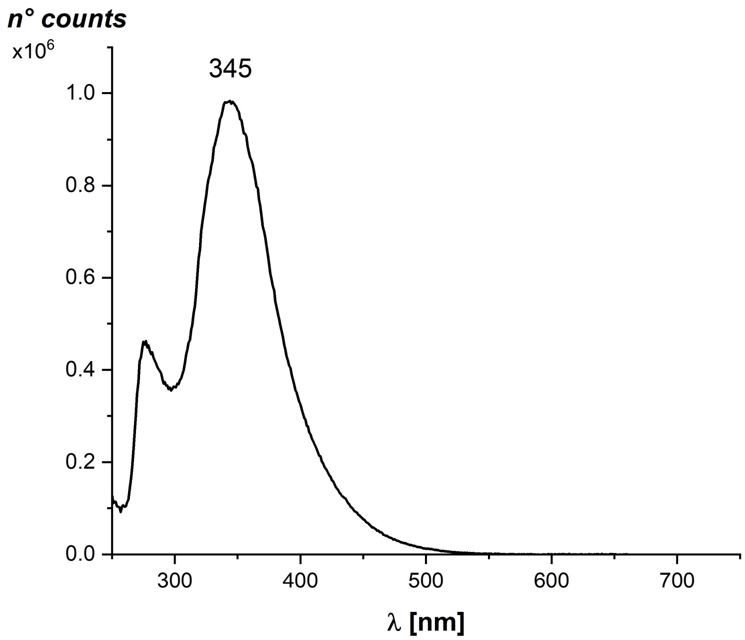

Figure S44. Excitation spectra of **1** dissolved in 3-methylpentane at room temperature, *c* = 4.4×10^{-6} M, λ_{em}: 670 nm.

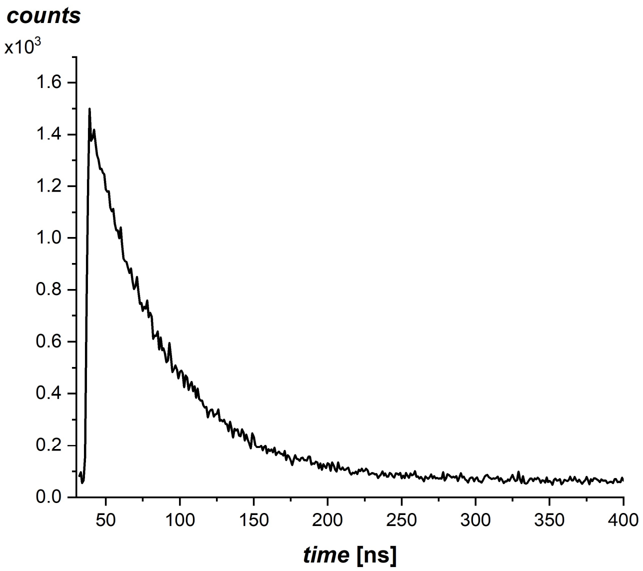

Figure S45: Decay times of **1** dissolved in 3-methylpentane at room temperature *c* = 4.4×10^{-6} M. τ = 48 ns., obtained from monoexponential decay fit.

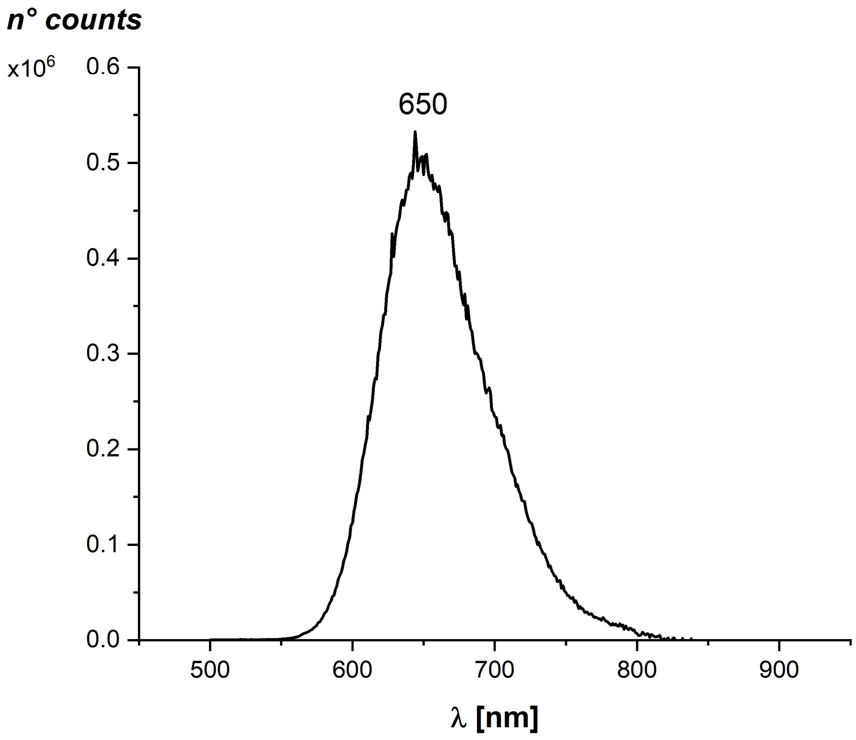

Figure S46: Emission spectrum of **1** in 3-methylpentane matrix at 77 K, $c = 4.4 \times 10^{-6}$ M, λ_{exc}: 485 nm.

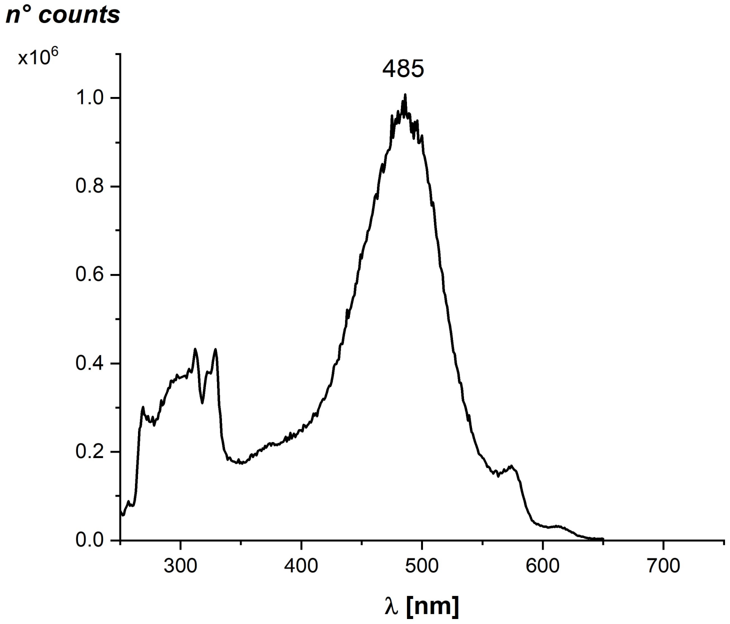

Figure S47. Excitation spectrum of **1** in 3-methylpentane matrix at 77 K, $c = 4.4 \times 10^{-6}$ M, λ_{em}: 670 nm.

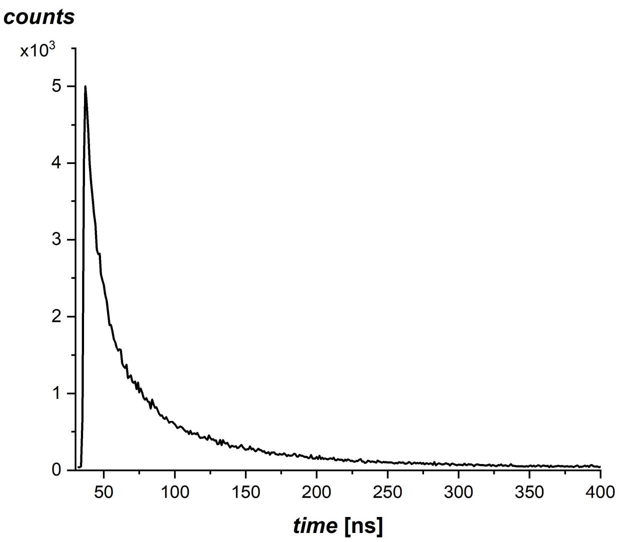

Figure S48: Decay times of **1** dissolved in 3-methylpentane at 77 K, $c = 4.4 \times 10^{-6}$ M, $\tau = 54$ ns, obtained from intensity weighted-average lifetime.

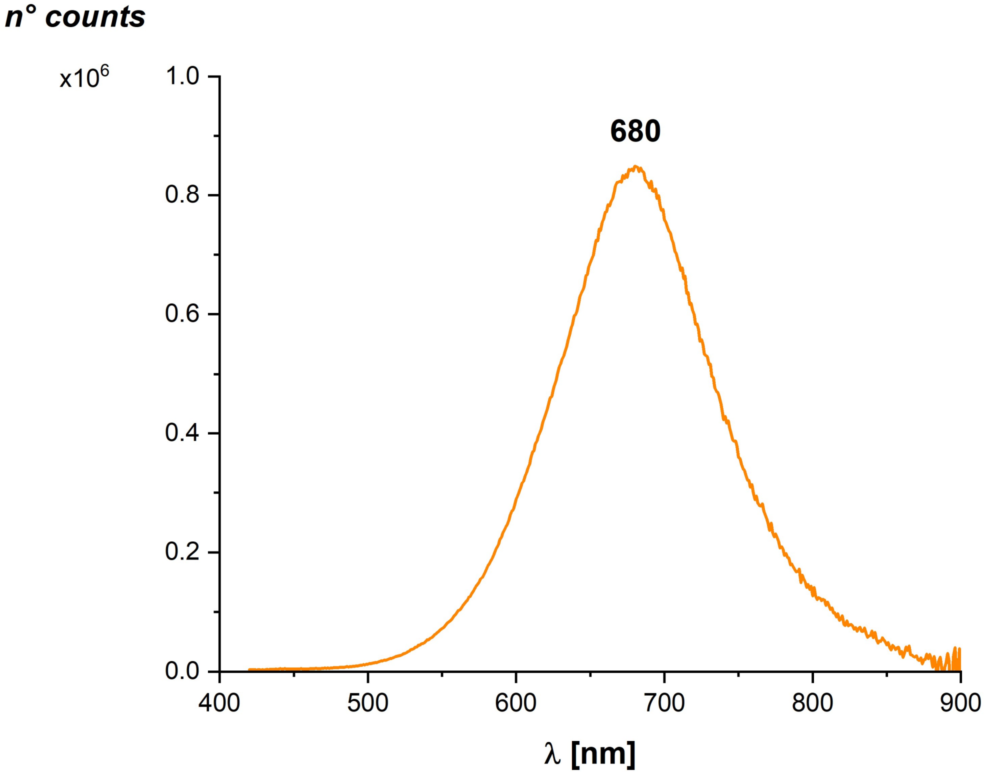

Figure S49: Emission spectrum of **1** in PMMA doped film (10%), λ_{exc}: 410 nm.

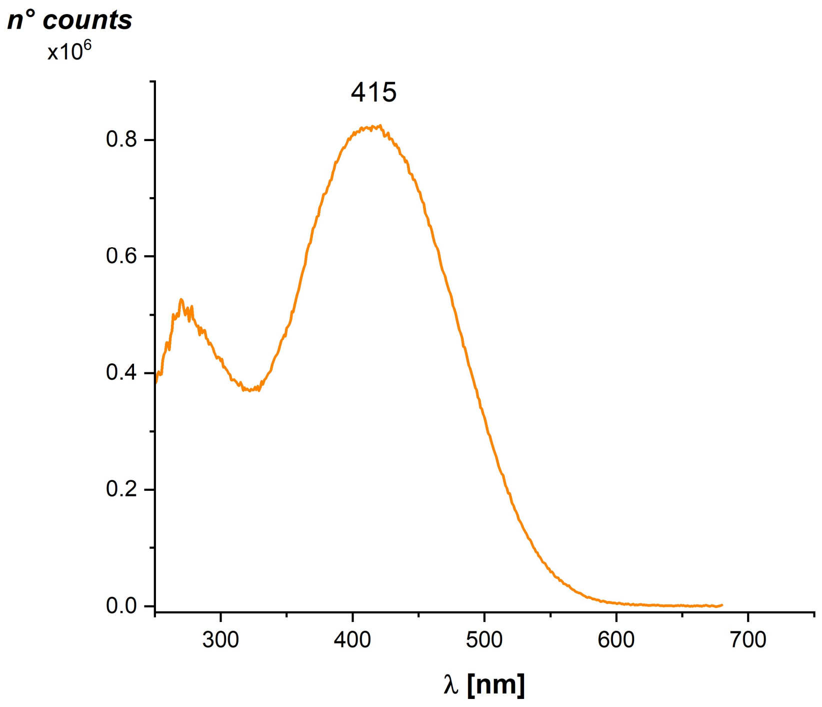

Figure S50: Excitation spectrum of **1** in PMMA doped film (10%), λ_{em}: 690 nm.

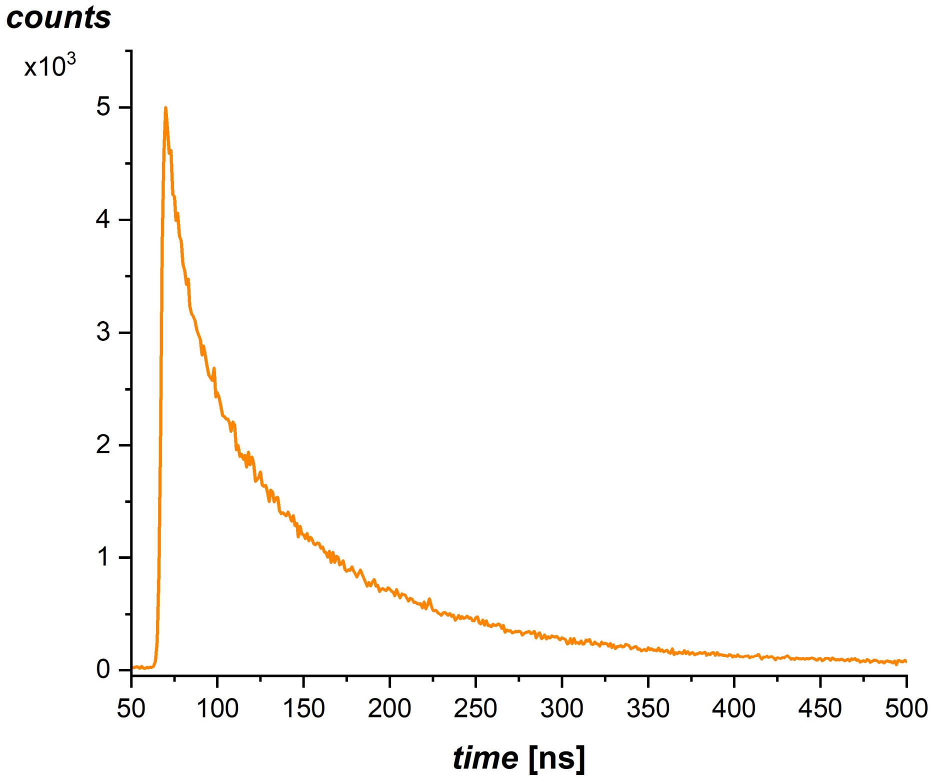

Figure S51: Decay time of **1** in PMMA doped film (10%).

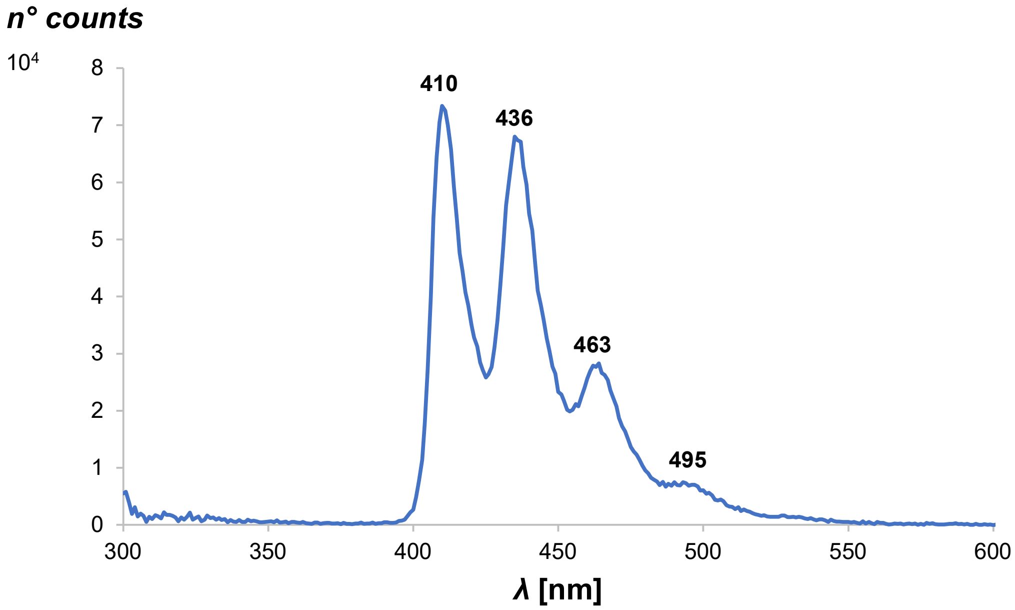

Figure S52: Emission spectrum of **2-H** in *n*-pentane, c = 1.4 × 10^{-5} M, λ_{exc}: 275 nm.

Table S5: Emission bands of **2-H** dissolved in *n*-pentane, c = 4.4 × 10^{-6} M.

**Solvent**	λ_{em} **[nm]**	***PLQY*** **[%]**	τ **[ns]**
Pentane	410<br>436<br>463<br>495	9	2

λ_{exc}	286 nm
slits	10 nm

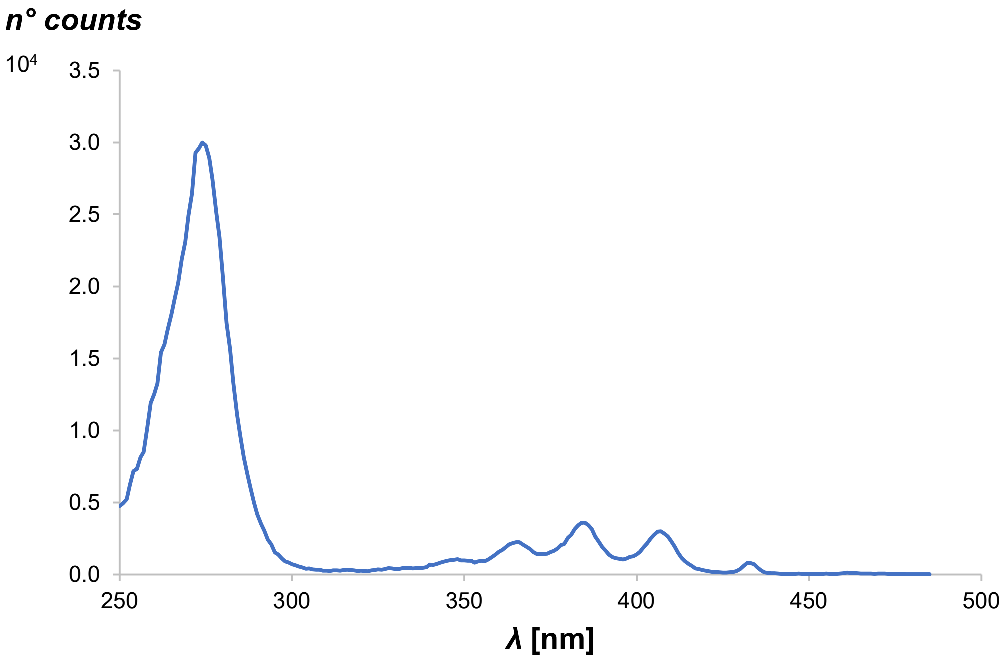

Figure S53: Excitation spectrum of **2-H** in *n*-pentane*, c* = 1.4 × 10^{-5} M, λ_{exc}: 495 nm.

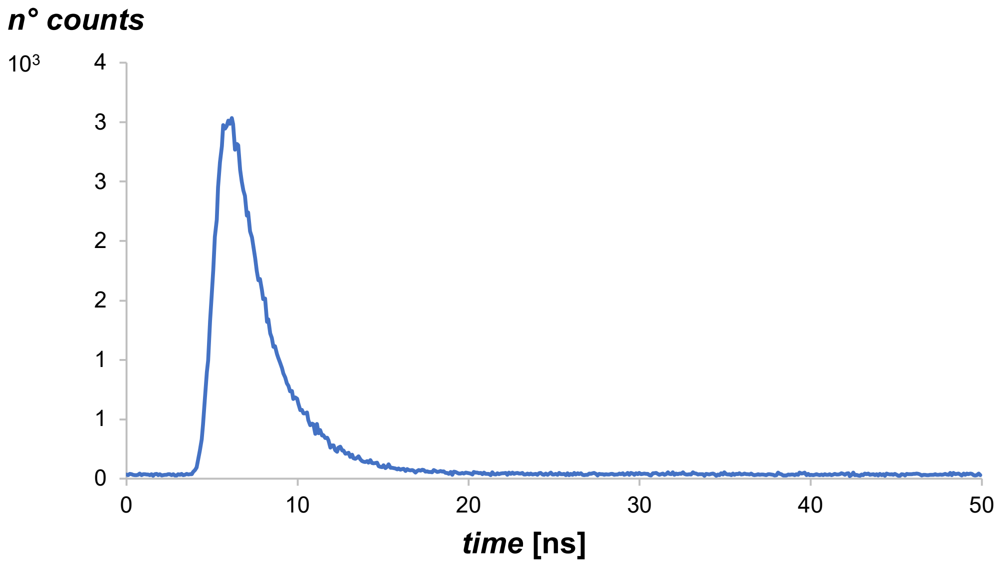

Figure S54: Plotted lifetime spectrum of **2-H** in *n*-pentane*, c* = 4.4 × 10^{-6} M, λ_{exc}: 286 nm, λ_{em}: 410 nm.

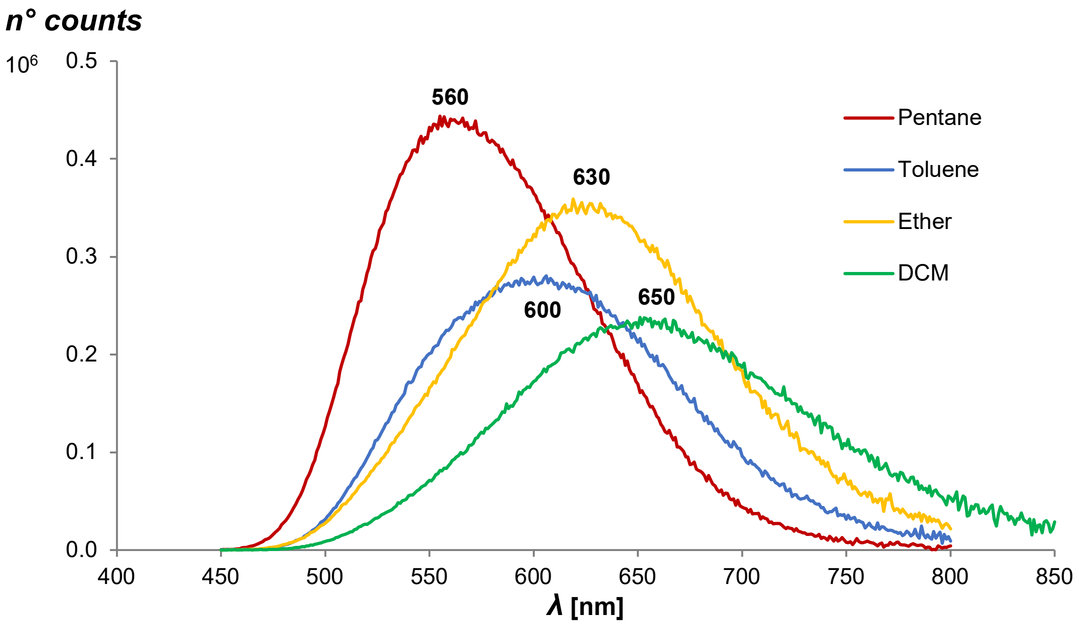

Figure S55: Emission spectra of **2** dissolved in *n*-pentane, toluene, Et_2O and DCM, c = 3.2 × 10^{-6} M.

Table S6: emission data related to **2** dissolved in *n*-pentane, toluene, Et_2O and DCM, c = 3.2 × 10^{-6} M.

Solvent	λ_{em} [nm]	*PLQY* [%]	τ [ns]
Pentane	560	44	14
Toluene	600	88	9
Ether	630	96	12
DCM	650	43	10

λ_{exc}	440 nm
slits	1-0.5 nm

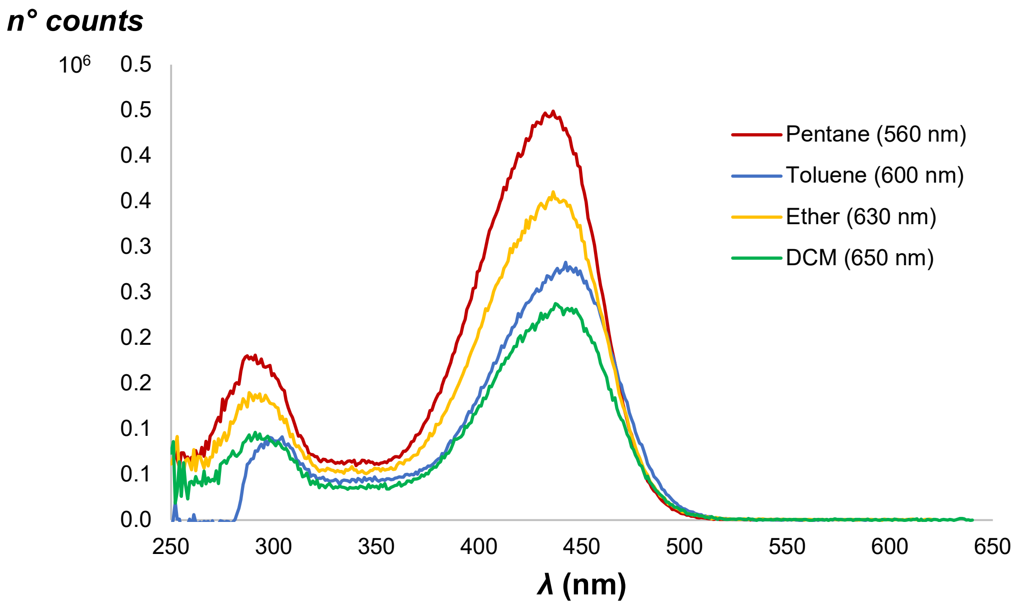

Figure S56: Excitation spectra of **2** dissolved in *n*-pentane, toluene, Et_2O and DCM, $c = 3.2 \times 10^{-6}$ M.

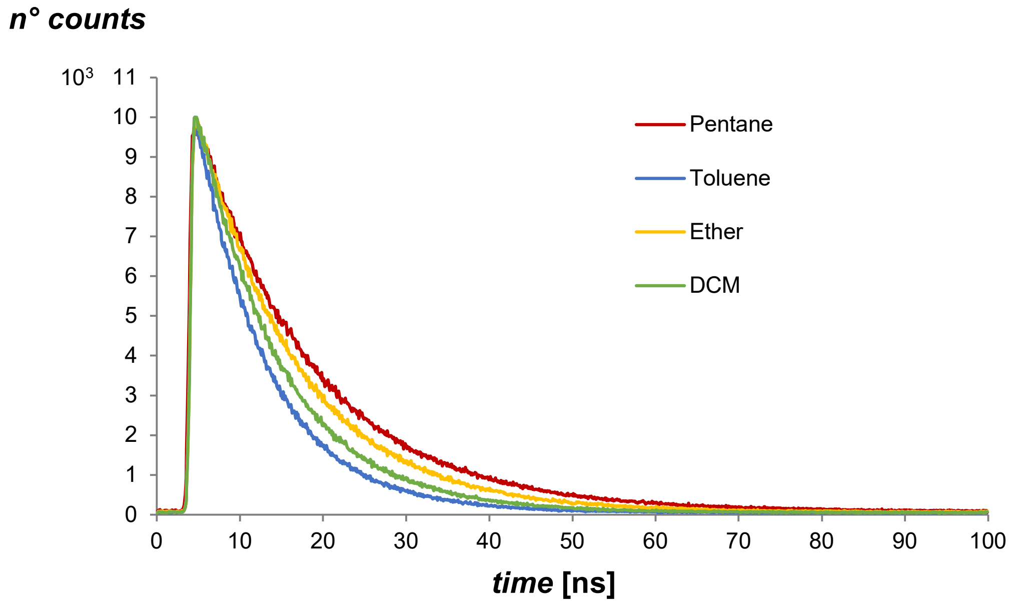

Figure S57: Lifetimes of **2** dissolved in *n*-pentane, toluene, Et_2O and DCM, $c = 3.2 \times 10^{-6}$ M.

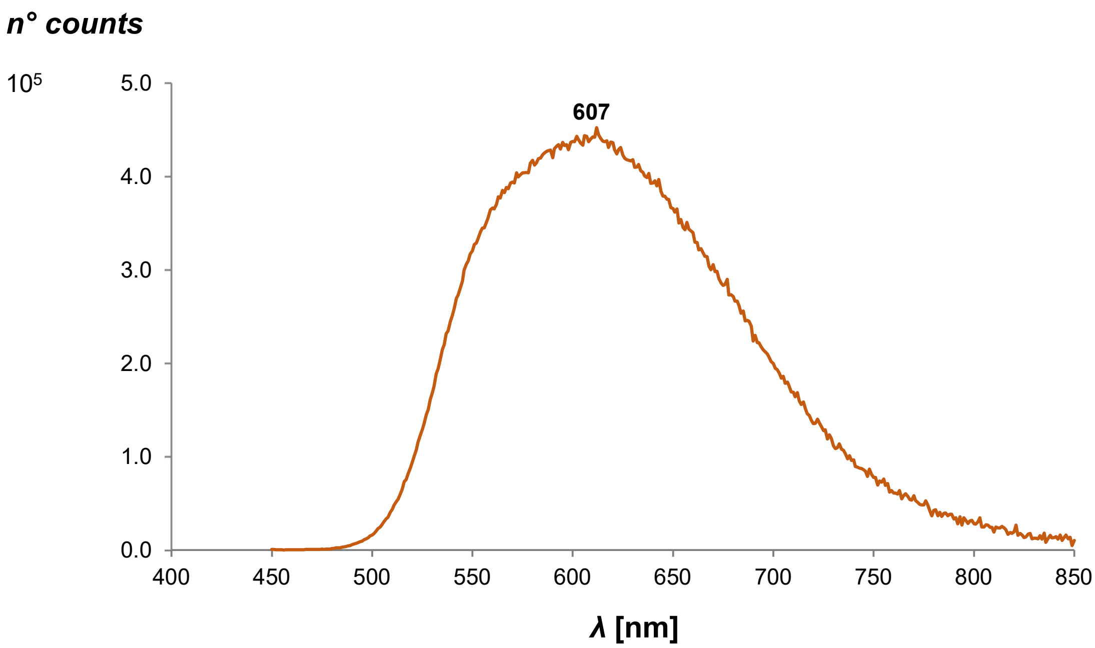

Figure S58: Emission spectrum of **2** in the solid state, dispersed in $BaSO_4$, λ_{exc}: 440 nm.

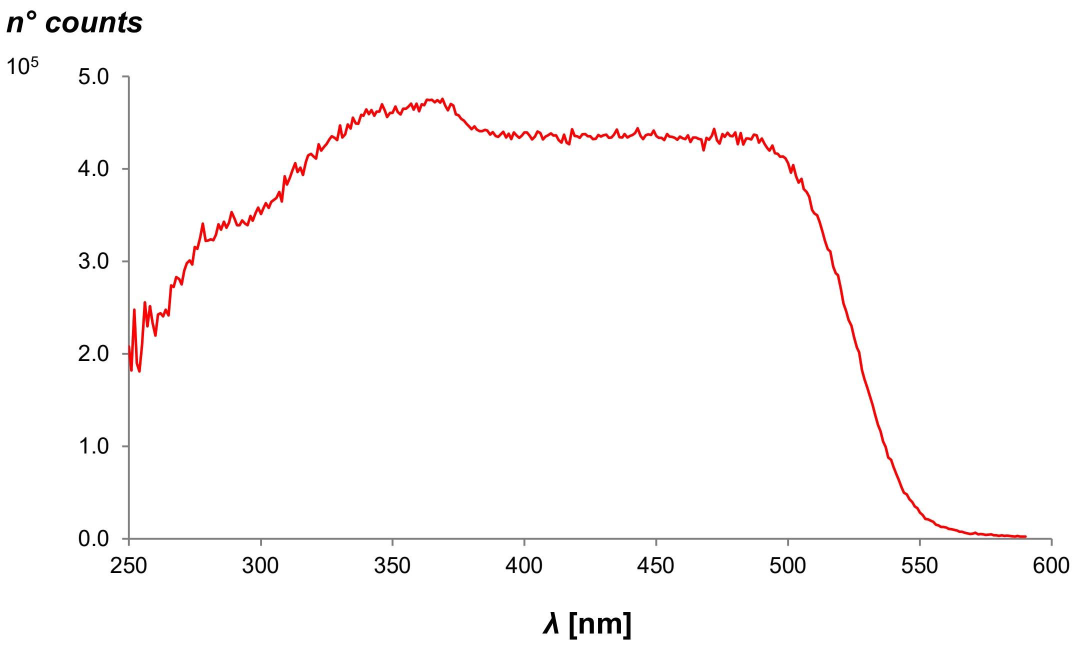

Figure S59: Excitation spectrum of **2** in the solid state, dispersed in $BaSO_4$, λ_{em}: 600 nm.

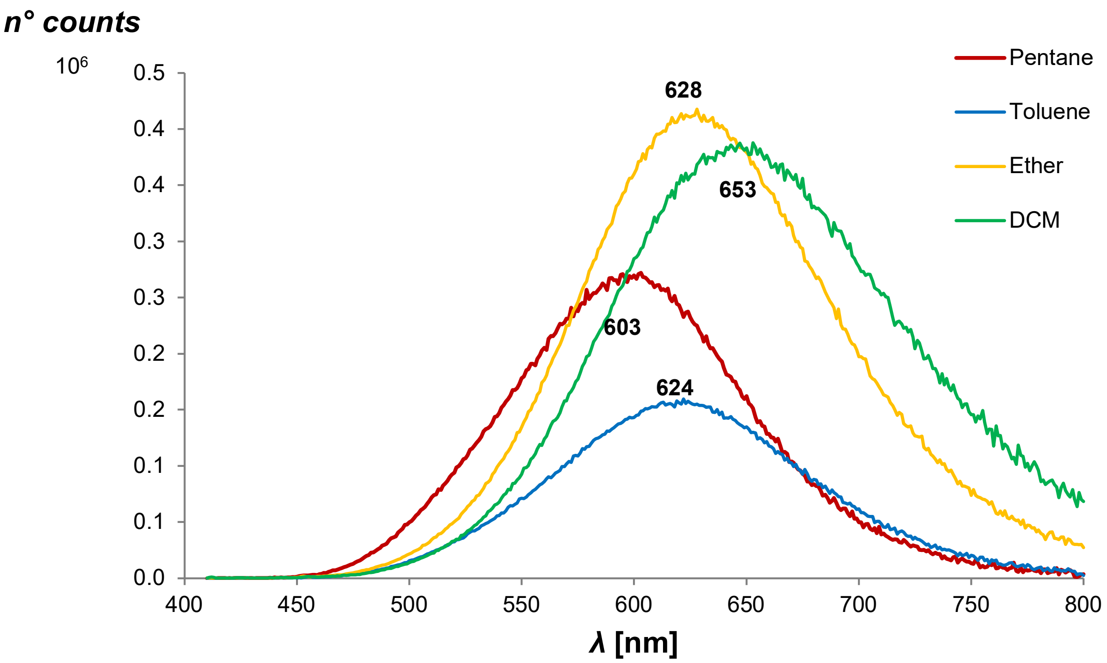

Figure S60: Superimposed emission spectra of **3** dissolved in *n*-pentane, toluene, Et_2O, DCM, $c = 2.4 \times 10^{-6}$ M.

Table S7: Emission data related to **3** dissolved in *n*-pentane, toluene, Et_2O, DCM, $c = 2.4 \times 10^{-6}$ M.

Solvent	λ_{em} [nm]	*PLQY* [%]	τ [ns]
Pentane	603	70	21
Toluene	624	74	12
Ether	628	63	13
DCM	653	43	8

λ_{exc}	400 nm
slits	1-0.7 nm

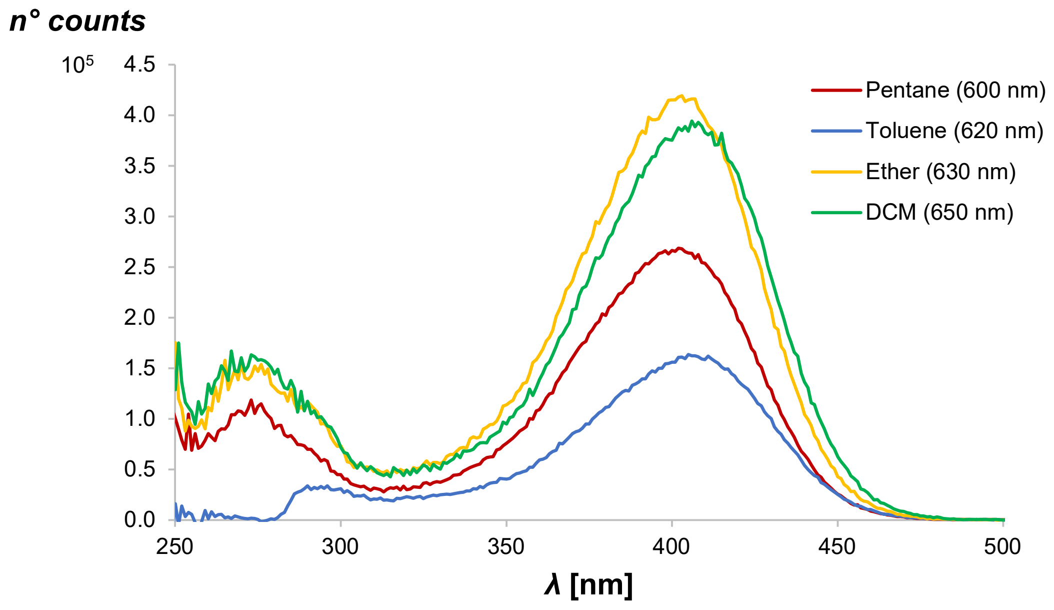

Figure S61: Superimposed excitation spectra of **3** dissolved in *n*-pentane, toluene, Et_2O, DCM, $c = 2.4 \times 10^{-6}$ M.

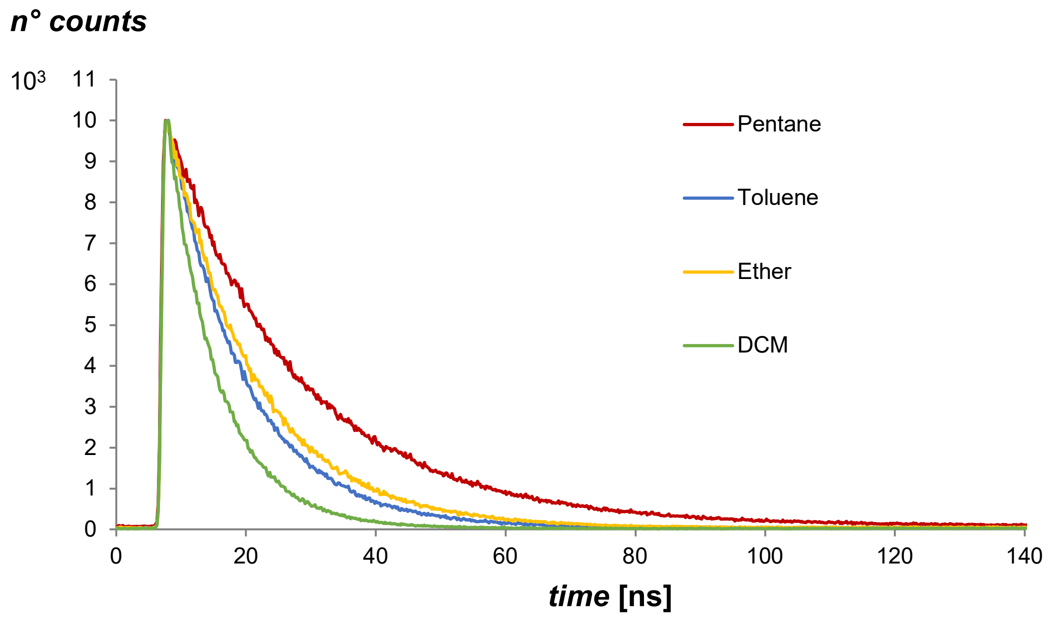

Figure S62: Superimposed decay times of **3** dissolved in *n*-pentane, toluene, Et_2O, DCM, $c = 2.4 \times 10^{-6}$ M.

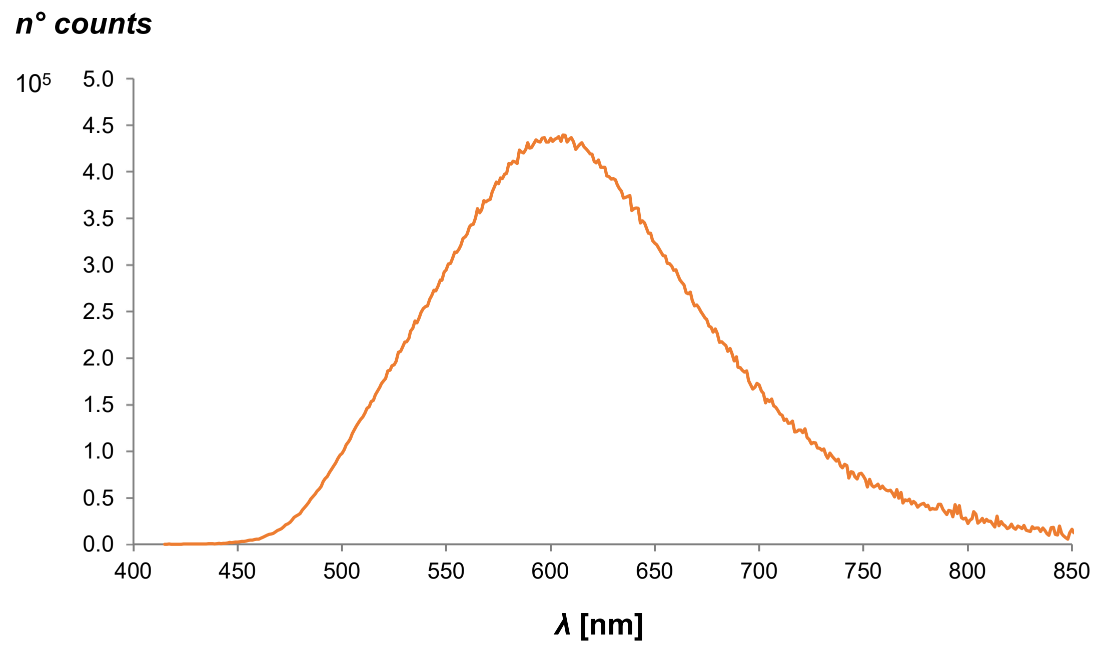

Figure S63: Emission spectrum of **3** in the solid state, diluted in $BaSO_4$, λ_{exc}: 440 nm.

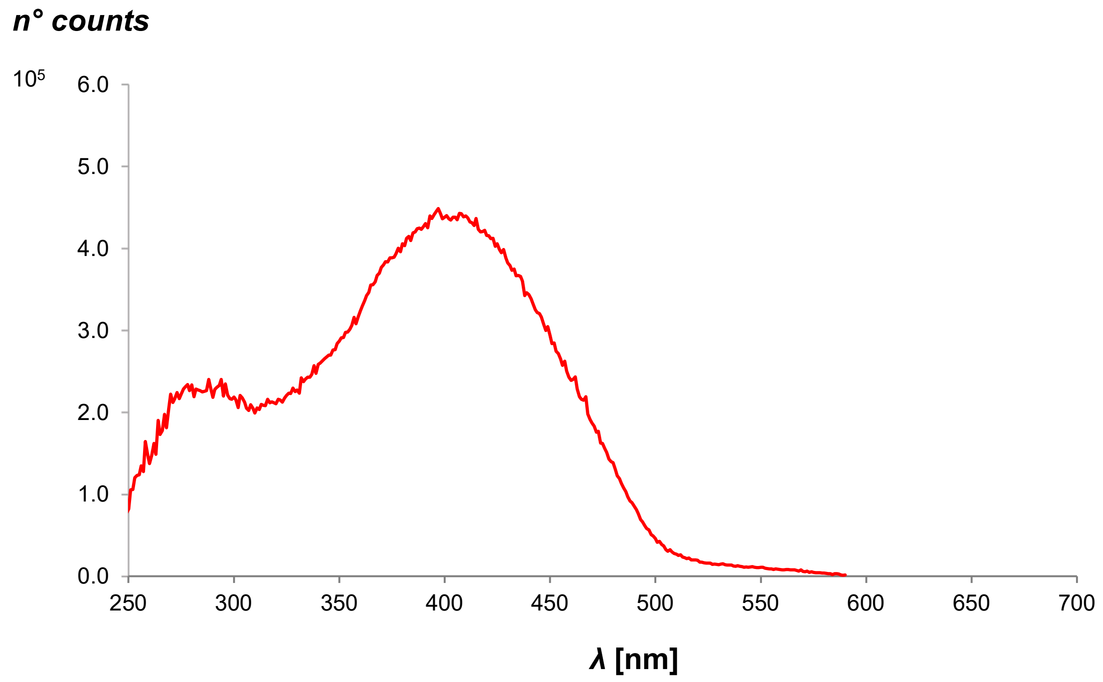

Figure S64: Excitation spectrum of **3** in the solid state, diluted in $BaSO_4$, λ_{em}: 600 nm.

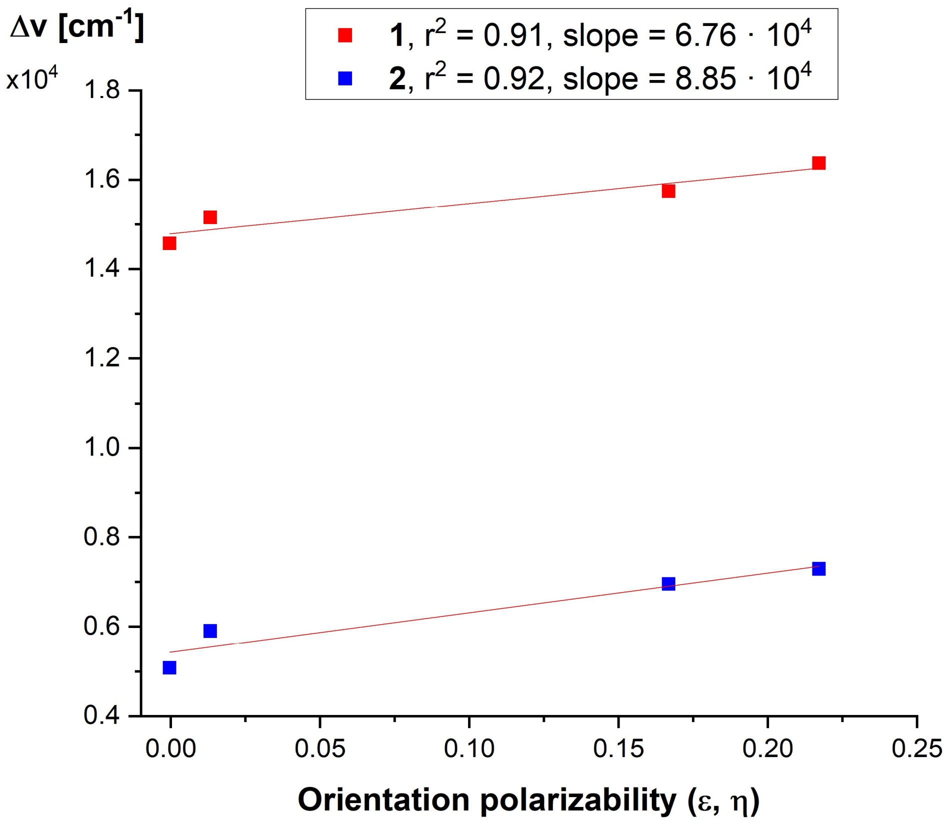

Figure S65. Linear solvatochromism model plots for **1** and **2**, showing Stokes Shifts plotted versus the solvents' orientation polarizability (Lippert-Mataga model).[7–9]

Table S8. Ground- and excited-state dipole moments for **1** and **2**. To approximate the Onsager-Radius, the vdW cavity obtained by the Gaussian charge scheme used by default in ORCA's CPCM module was used and converted to an ideal sphere to obtain the corresponding idealized radius. Dipole moments of the ground states in the gas-phase were obtained at the PBE0/def2-TZVPP//PBE0-D4/def2-SVP level of theory.

Compound	*a* [Å]	*mf*(ε, η) [cm^{-1}]	μ_g [Debye]	μ_e [Debye]
**1**	10.457	6757	1.80	29.30
**2**	10.842	8854	0.01	33.67

6. Crystallographic data

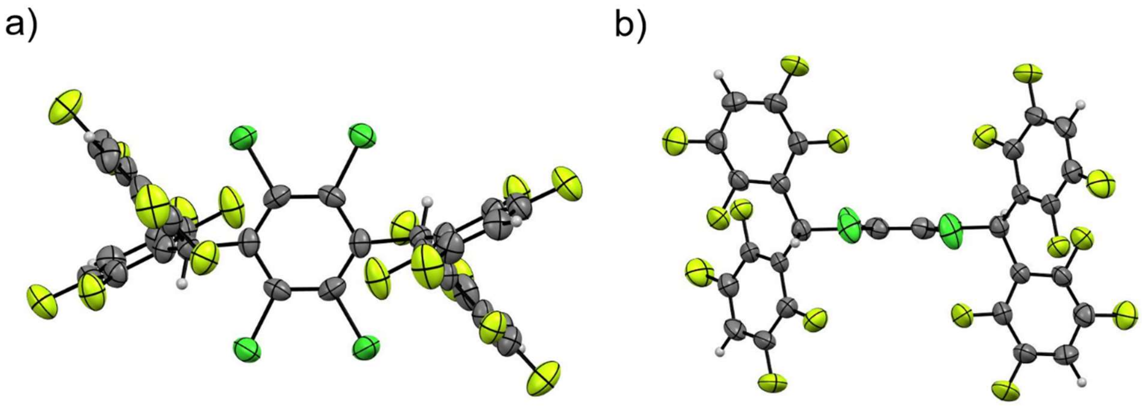

Figure S66: Molecular structure of **1-H** in the solid state with views a) perpendicular and b) parallel to the central C_6Cl_4 bridge unit. Solvent molecules are omitted for clarity. Thermal ellipsoids are displayed at 50% probability. Color code: green (Cl), yellowish (F), gray (C), white (H).

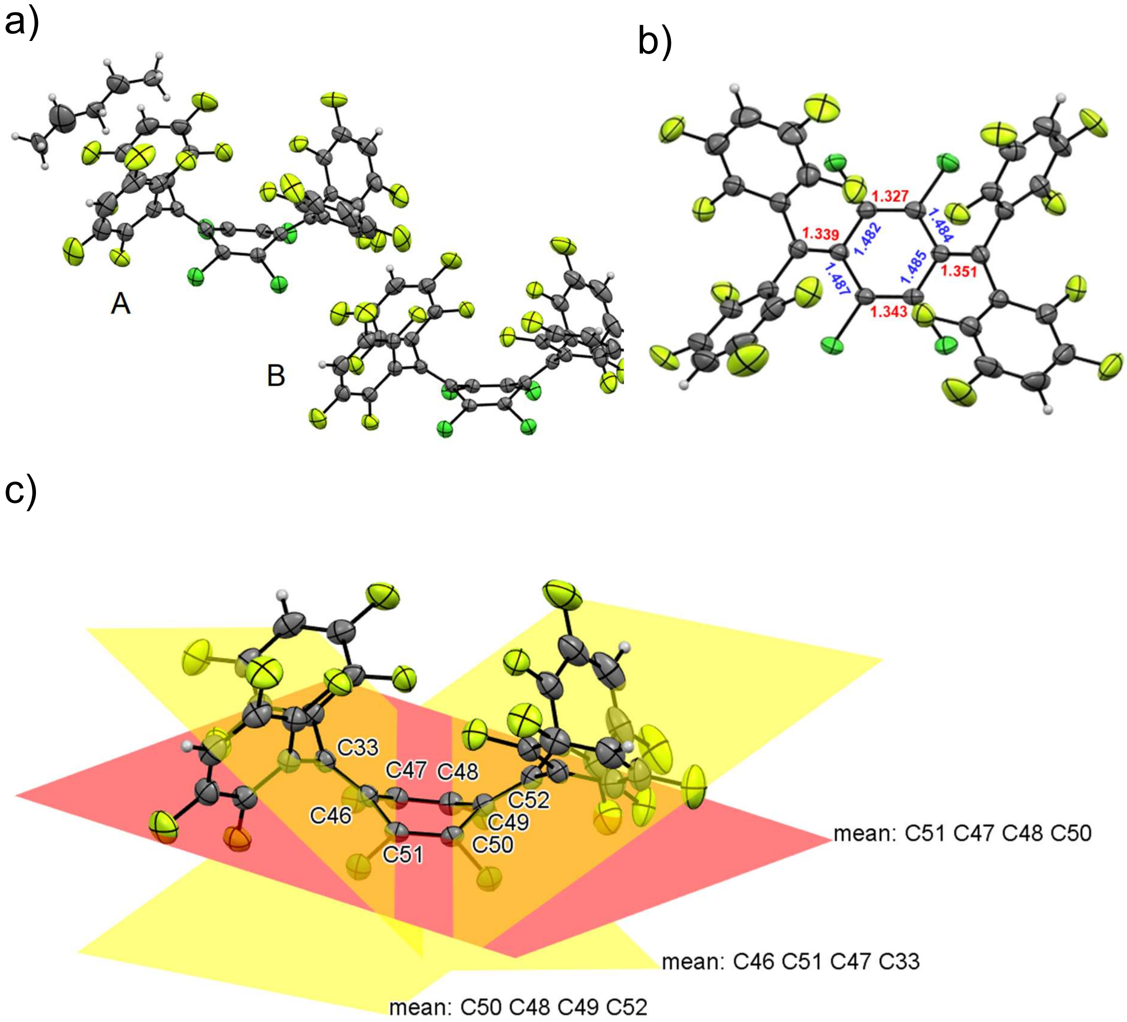

Figure S67: a) Solid state structure of **1** showing the two independent molecules (A and B) and the *n*-hexane co-crystallized solvent molecule. b) Molecular structure of **1** in the solid state (molecule A, not shown in the main text of the article) with view normal to the central C_6Cl_4 bridge unit. Solvent molecules are omitted for clarity. Color code: green (Cl), yellowish (F), gray (C), white (H). c) the dihedral angle of the folded compound **1** is defined as the angle between the average planes defined by C47, C48, C50, C51 and C33, C46, C47, C51 or C48, C49, C50, C52 (showed here for molecule B).The values of the so defined dihedral angles are 31.03 ° and 33.63 ° for molecule A and 34.70 ° and 32.60 ° for molecule B. Thermal ellipsoids are displayed at 50% probability.

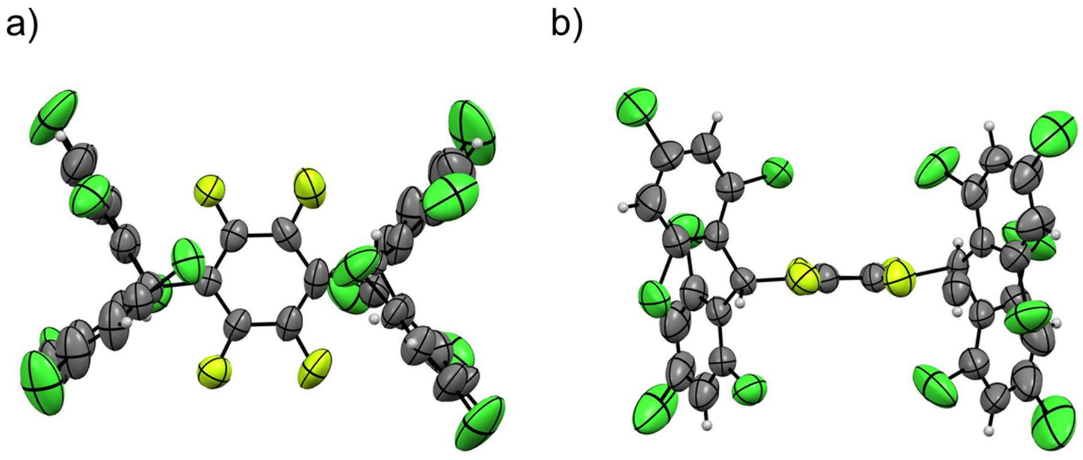

Figure S68: Molecular structure of **2-H** in the solid state with views a) perpendicular and b) parallel to the central C_6F_4 bridge unit. Solvent molecules are omitted for clarity. Thermal ellipsoids are displayed at 50% probability. Color code: green (Cl), yellowish (F), gray (C), white (H).

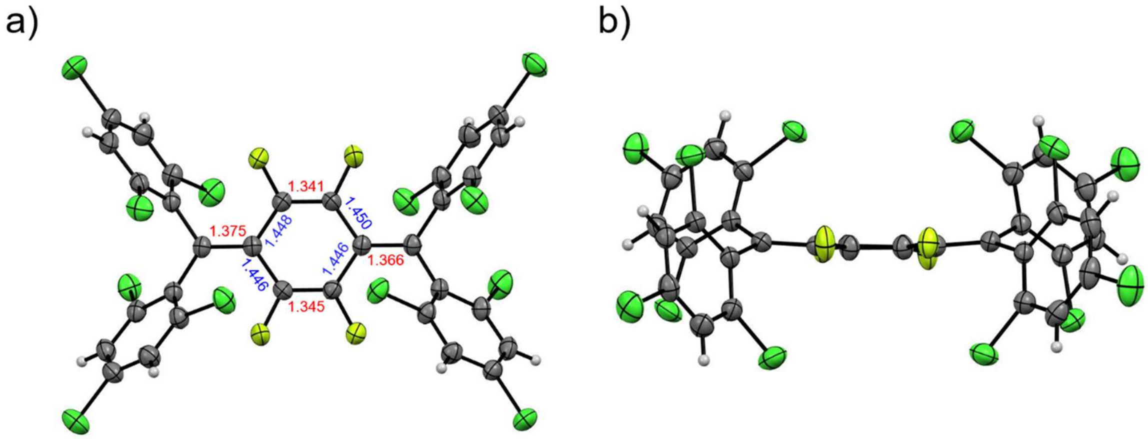

Figure S69: Molecular structure of **2** in the solid state with views a) perpendicular and b) parallel to the central C_6Cl_4 bridge unit. Solvent molecules are omitted for clarity. Thermal ellipsoids are displayed at 50% probability. Color code: green (Cl), yellowish (F), gray (C), white (H).

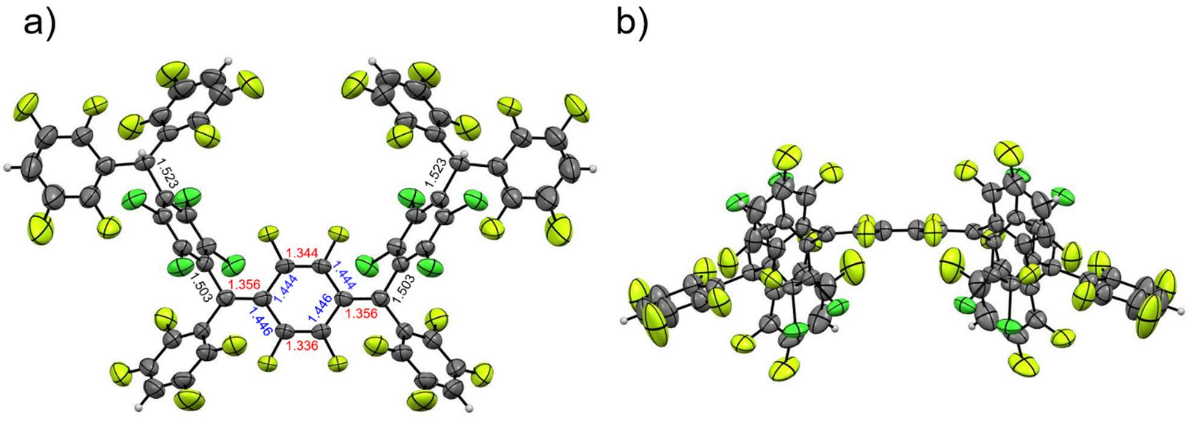

Figure S70: Molecular structure of **3** in the solid state with views a) perpendicular and b) parallel to the central C_6F_4 bridge unit. Solvent molecules are omitted for clarity. Thermal ellipsoids are displayed at 50% probability. Color code: green (Cl), yellowish (F), gray (C), white (H).

Table S9. Crystallographic details for compounds **1-H**, **2-H**, **1**, **2** and **3**.

Compound	**1-H**	**2-H**	**1**	**2**	**3**
Formula	$C_{33}H_{8}Cl_{6}F_{16}$	$C_{34}H_{12}Cl_{18}F_{4}$	$C_{66.50}H_{14}Cl_{8}F_{32}$	$C_{38}H_{22}Cl_{12}F_{4}$	$C_{64}H_{22}Cl_{8}F_{28}$
Formula weight	921.09	1134.54	1704.38	979.95	1606.41
Crystal system	Triclinic	Monoclinic	Monoclinic	Monoclinic	Orthorhombic
Space group	P-1	C c	C2/c	$P2_1/c$	Pbcm
a/Å	12.0571(9)	15.9954(9)	44.249(2)	11.8062(7)	14.6619(8)
b/Å	12.7550(8)	22.0613(12)	10.1440(5)	22.4550(12)	12.7343(7)
c/Å	13.0372(9)	12.9068(7)	29.7984(14)	15.3101(10)	35.4165(18)
α/°	80.606(3)	90.00	90.00	90.00	90.00
β/°	64.887(3)	104.071(2)	95.322(2)	97.919(2)	90.00
γ/°	68.978(3)	90.00	90.00	90.00	90.00
Volume/Å^3	1694.5(2)	4417.9(4)	13317.6(11)	4020.1(4)	6612.6(6)
T (K)	200	300	200	300	290
Z	2	4	8	4	4
D_{calc}/g·cm^{-3}	1.805	1.706	1.700	1.619	1.614
F(000)	904	2232	6696	1960	3176
μ(Mo-Kα)/mm^{-1}	6.23	11.60	4.71	8.75	4.61
Reflections collected	90058	125884	143390	173949	75725
Unique reflections	6944	9024	13602	9975	6898
Observed reflections [I > 2σ(I)]	4924 [R_{int} = 0.0572]	5526 [R_{int} = 0.0452]	9564 [R_{int} = 0.0574]	8267 [R_{int} = 0.0352]	3954 [R_{int} = 0.0615]
R [I > 2σ(I)]	*R*1 = 0.0820, *wR*2 = 0.2326	*R*1 = 0.0324, *wR*2 = 0.0918	*R1 = 0.0571 wR2 = 0.1557*	*R1 = 0.0481 wR2 = 0.1177*	*R1 = 0.0547 wR2 = 0.1578*
R [all data]	*R1 = 0.1091, wR2 = 0.2600*	*R*1 = 0.0382, *wR*2 = 0.0961	*R1 = 0.0850 wR2 = 1776*	*R1 = 0.0584 wR2 = 0.1258*	*R1 = 0.1037 wR2 = 0.2103*

$$R1 = (\Sigma||F_o| - |F_c||/\Sigma|F_o|);\ wR2 = \{\Sigma[w(F_o^2 - F_c^2)^2]/\Sigma[w(F_o^2)^2]\}^{1/2}$$

7. Thermogravimetric analysis

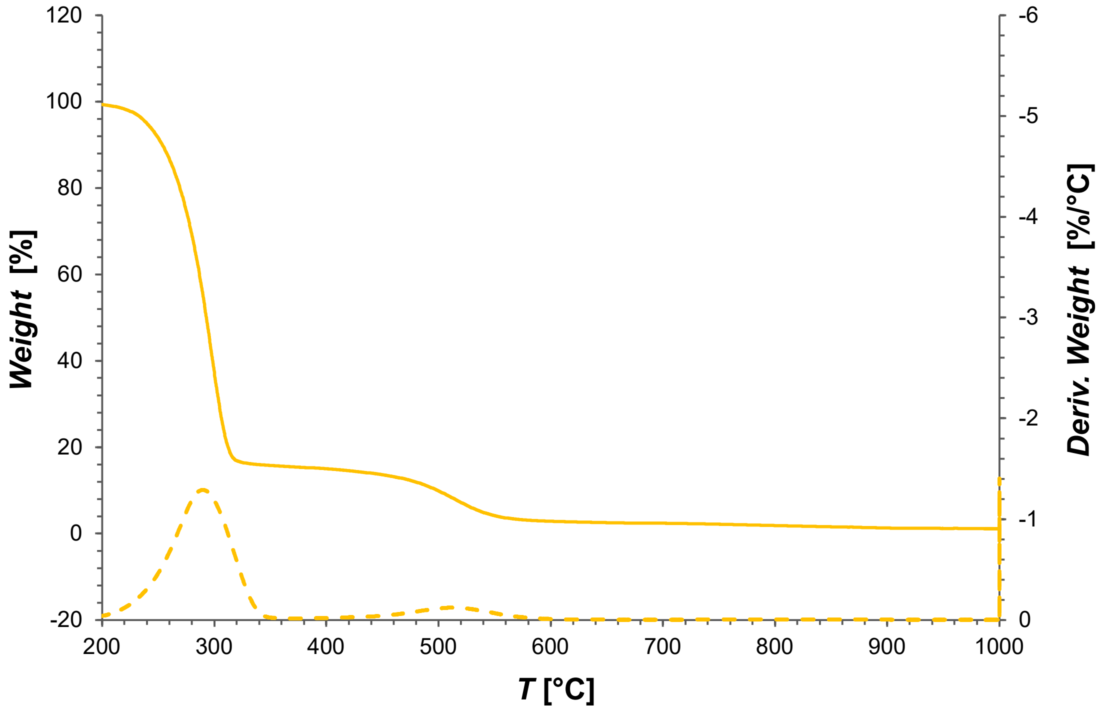

Figure S71: TGA analysis of **1**.

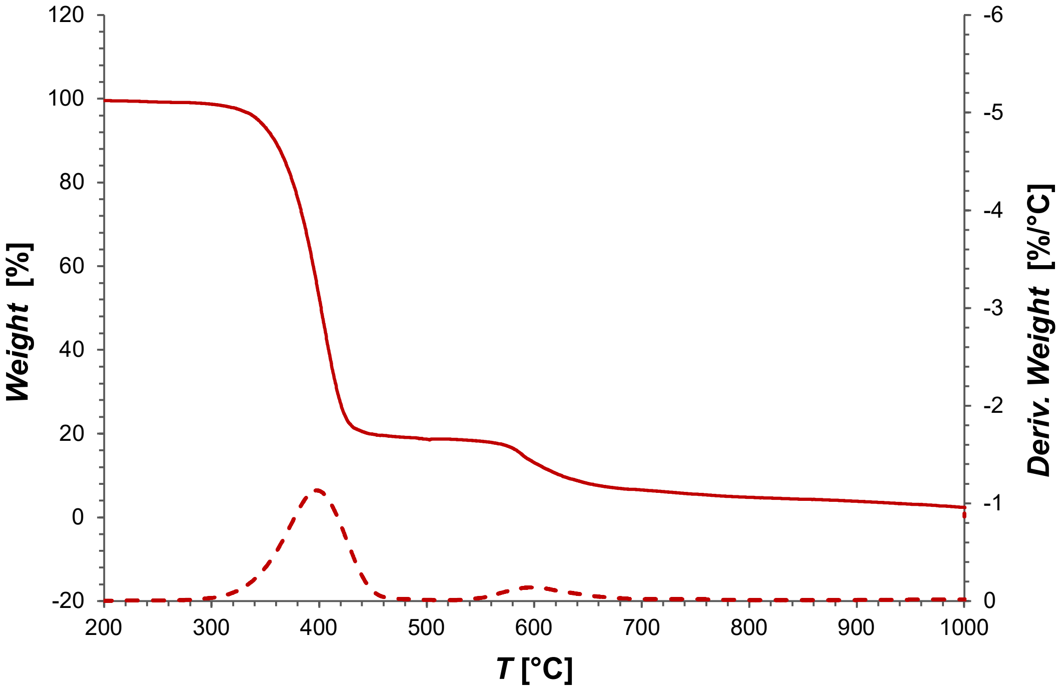

Figure S72: TGA analysis of **2**.

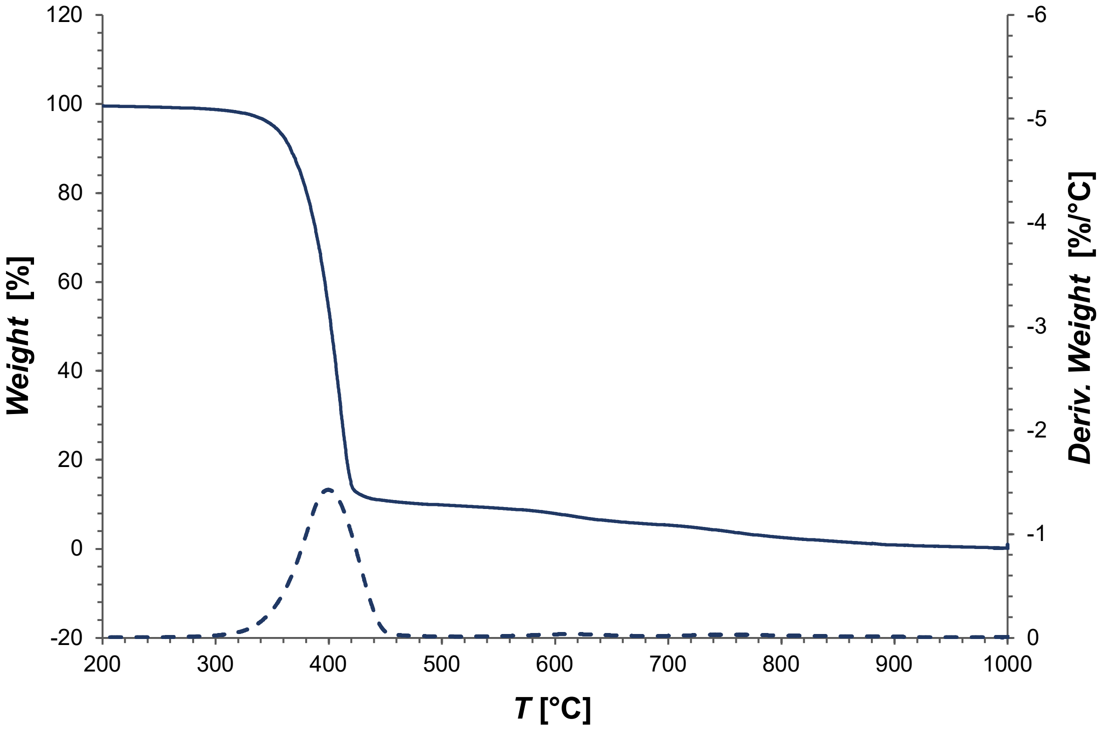

Figure S73: TGA analysis of **3**.

8. EPR spectra of 1 and 2

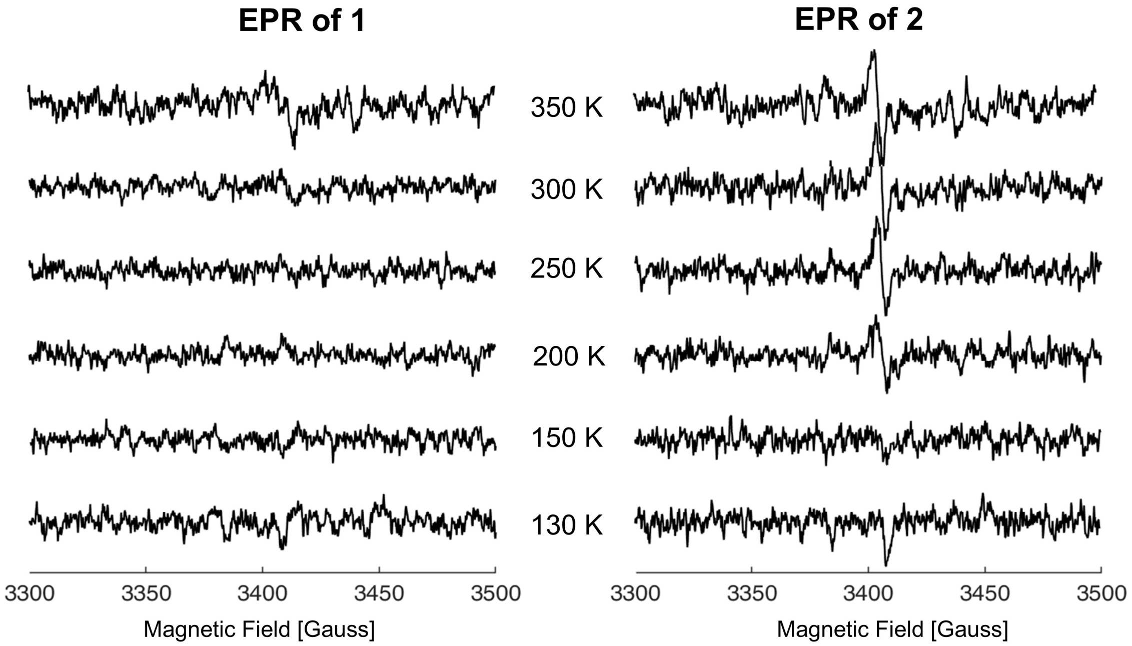

Figure S74: EPR spectra of **1** and **2** dissolved in toluene at 350 K, 300 K, 250 K, 200 K, 150 K and 130 K.

9. Cyclic voltammetry of 1, 2 and 3

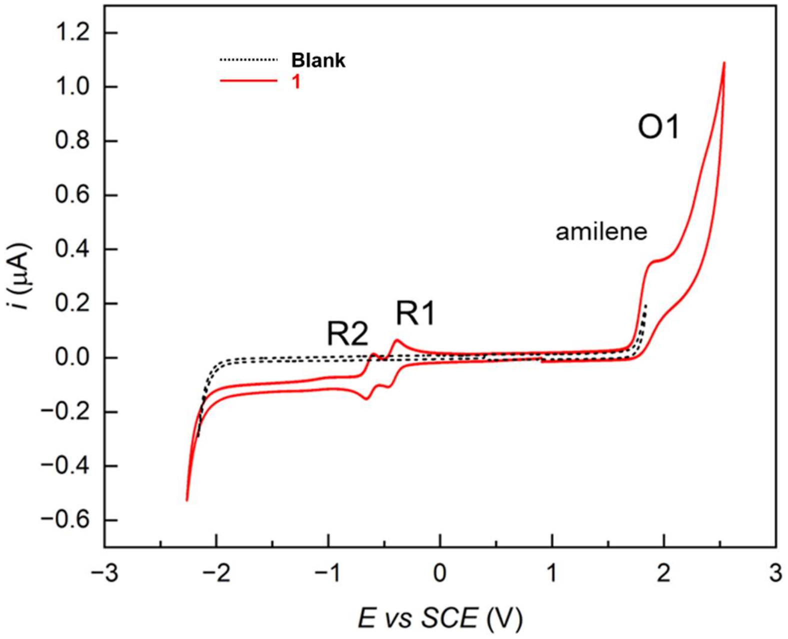

Figure S75: Cyclic voltammetry of **1** obtained in DCM and $TBAPF_6$ 0.1 M on a glassy carbon electrode, *v* = 0.2 V/s.

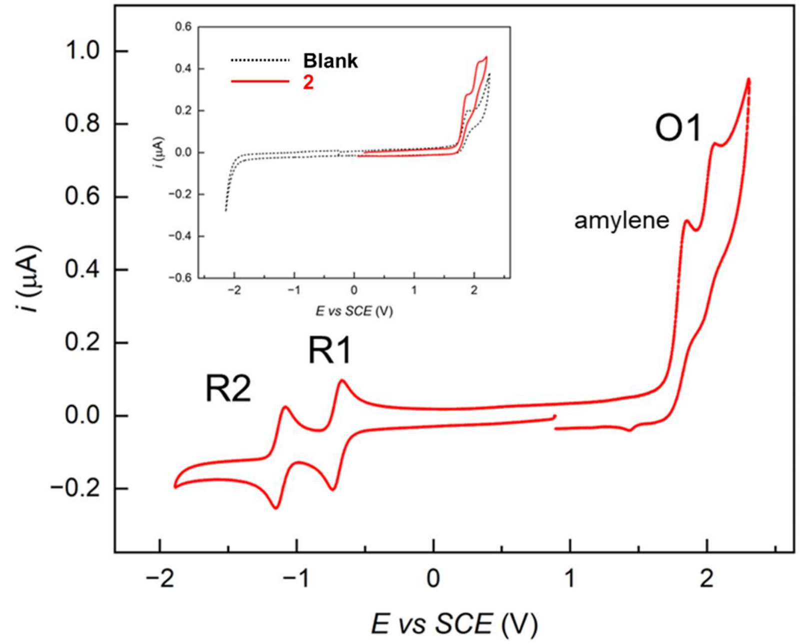

Figure S76: Cyclic voltammetry of **2** obtained in DCM and $TBAPF_6$ 0.1 M on a glassy carbon electrode, *v* = 0.2 V/s.

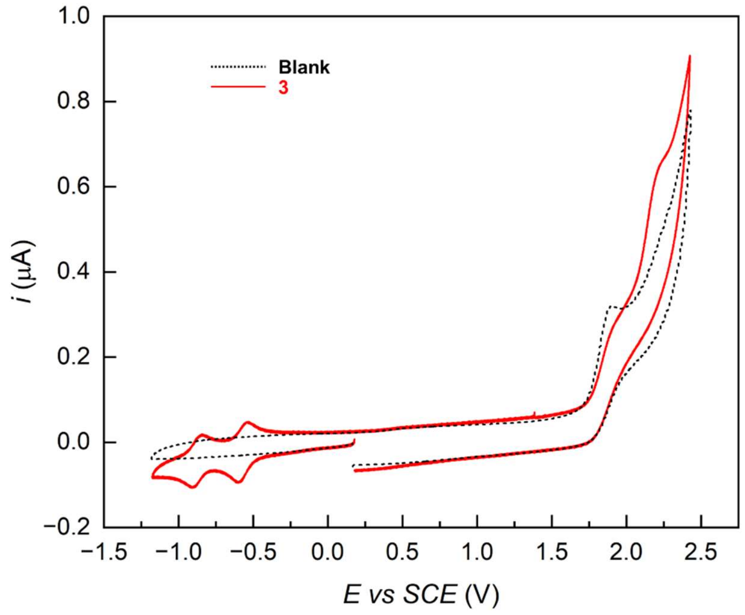

Figure S77: Cyclic voltammetry of **3** obtained in DCM and $TBAPF_6$ 0.1 M on a glassy carbon electrode, v = 0.2 V/s.

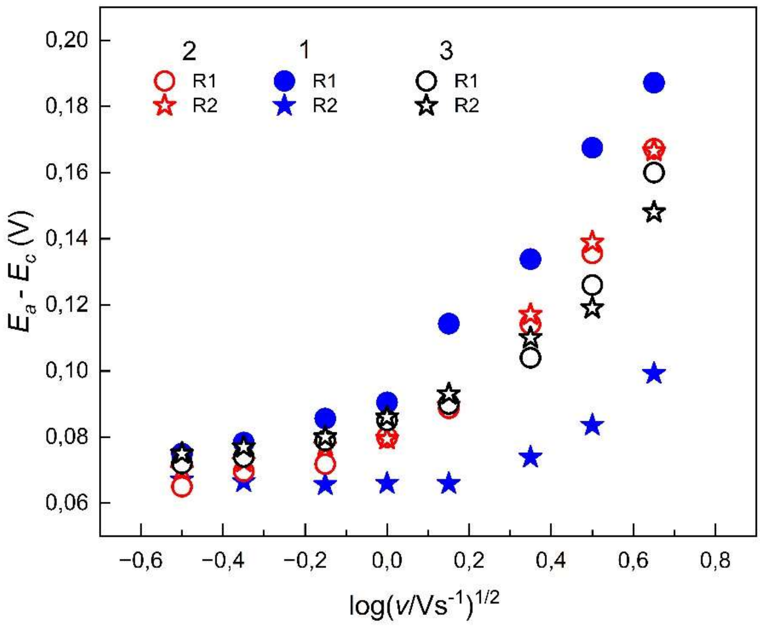

Figure S78: Plot of the variation of anodic-cathodic peaks separation for the first (R1) and second (R2) reduction with the square root of scan rate for compounds **1**, **2** and **3**. See the legend for symbol assignments.

10. fs-TAS of III, 3 and 1.

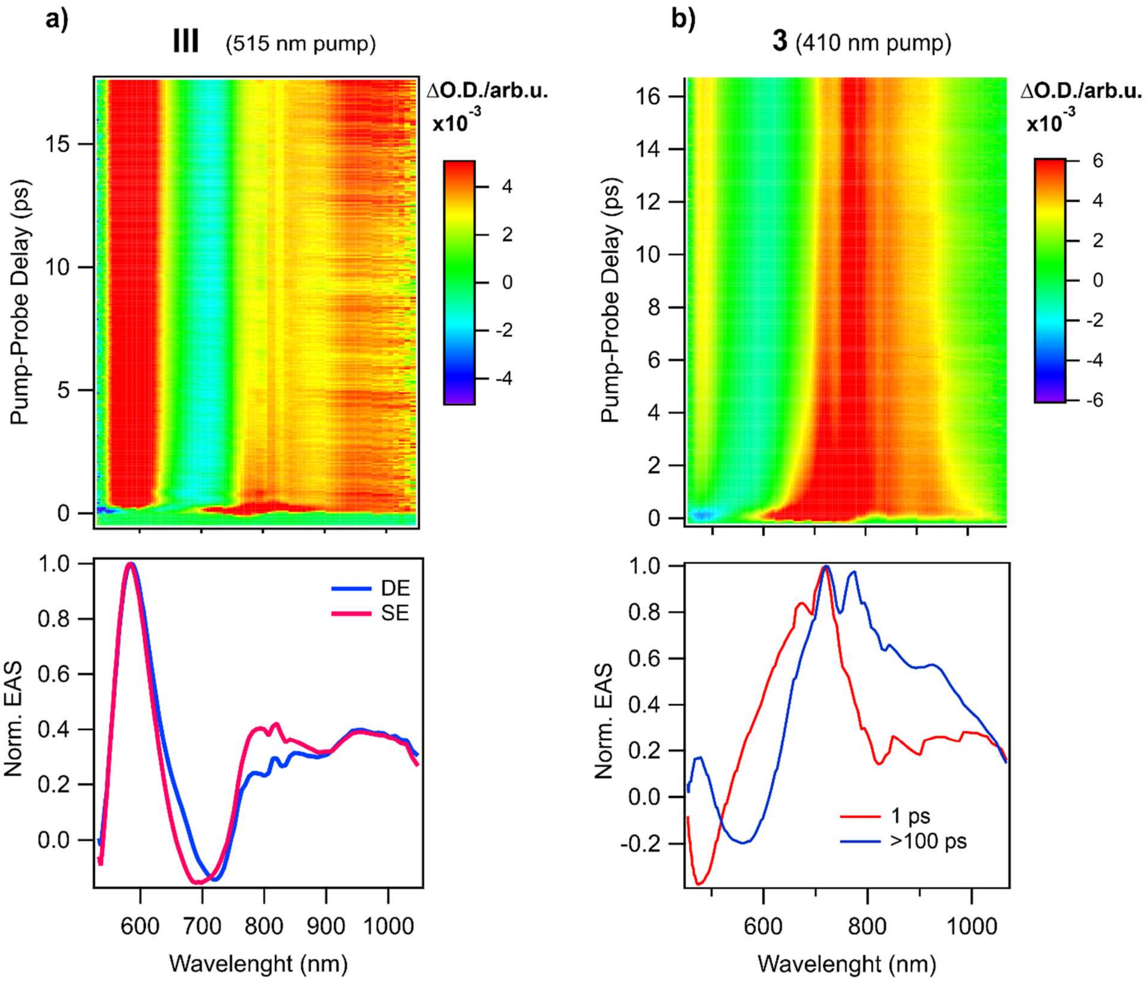

Figure S79: Top: Femtosecond transient absorption (fs-TA) maps of **III**[1] (pumped at 515 nm) and of **3** (pumped at 410 nm) in toluene. Bottom: Normalized evolution-associated spectra (EAS) derived from sequential global fitting of the fs-TA data.

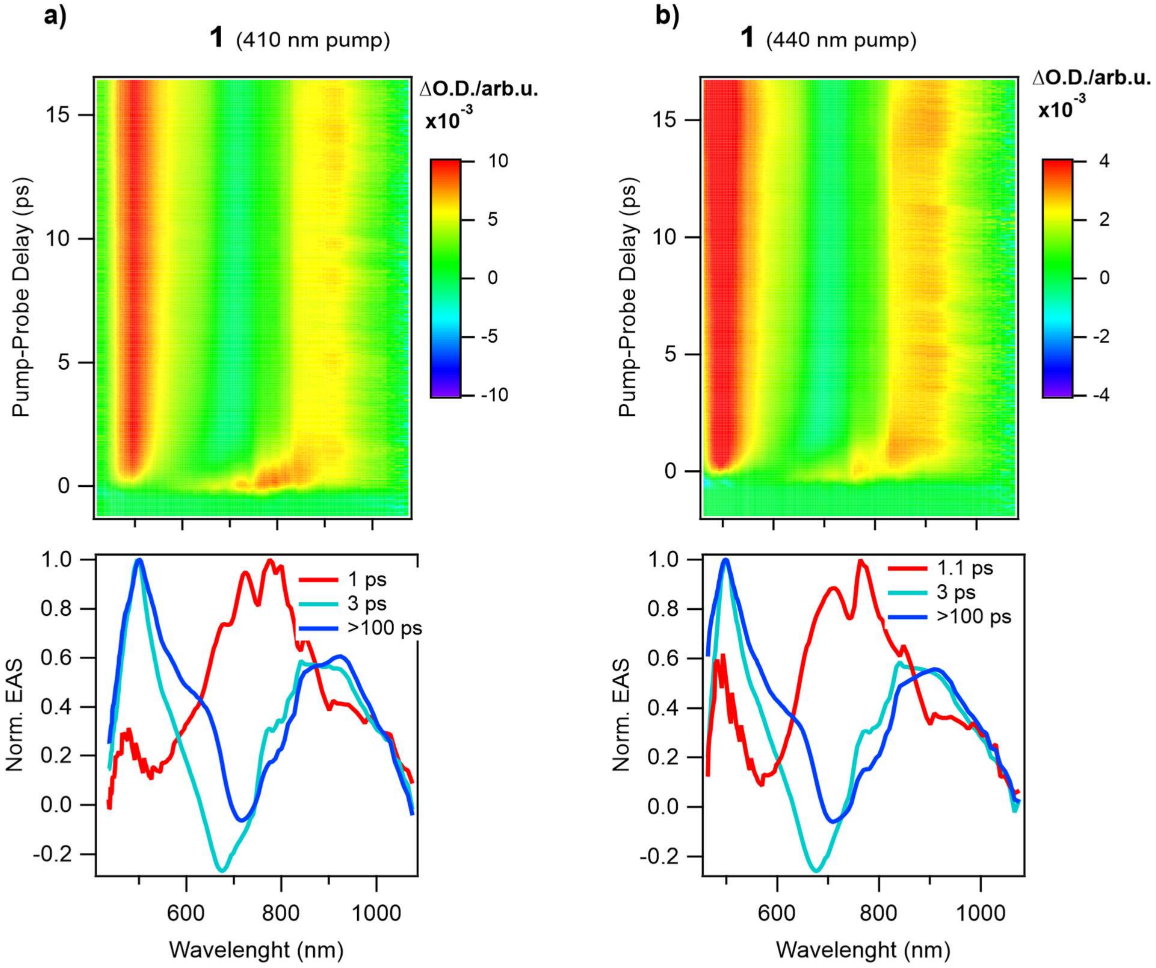

Figure S80: Top: Femtosecond transient absorption (fs-TA) maps of molecule **1** in toluene pumped at 440 nm and 410 nm. Bottom: Normalized evolution-associated spectra (EAS) derived from sequential global fitting of the fs-TA data. The EAS retrieved from the global fitting do not show variations with pump.

11. Computational Details

All calculations were performed using ORCA v. 6.0.1 and 6.1.0 (CASPT2K, MCPDFT, CI-scr-ctDFT).[10] For the calculation of the structural parameters and in order to gauge the influence of HF-exchange, four different functionals were evaluated (PBE-D_4,[11,12] PBE0-D4,[13] TPSSh-D4[14,15] and ωB97X-V[16,17]) using the def2-SVP[18] basis set. The RIJCOSX[19] approximation and the related auxiliary basis set (*def2/J*) were used to speed up the calculations. Tighter-than-default scf (*tightscf*) and optimization (*tightopt*) convergence criteria were employed. The unrestricted formalism (*UKS*) was applied, in case complemented with broken-symmetry calculations starting from the triplet excited states to converge to the open-shell diradical(oid) electronic structures. All optimized geometries were verified as true minima by the absence of imaginary frequencies in the harmonic vibrational analysis; in case of ωB97X-V, the hessian was computed numerically. The energies of all structures were refined by single-point calculations at the def2-TZVPP level of theory, and solvation energies in dichloromethane were computed by Open-COSMO-RS.[20]

The CASSCF optimizations were performed with CAS(6,6) and the def2-SVP basis set. Optimization to the excited states was conducted based on the state-averaged wavefunctions, where the weight of the undesired states was set to zero.

The CASSCF single-point calculations [CAS(14,14)] for the assessment of the vertical excited states were performed with the def2-TZVPP basis set using the experimentally determined solid-state structures with the position of all hydrogen atoms optimized (*optimizehydrogens true*); in case of **1^{flat}**, the optimized unrestricted PBE0-D4 structural parameters were used. Absorption spectra were simulated at the NEVPT2,[21] CASPTK,[22] MCPDFT[23] and CI-sr-ctDFT[24,25] (ctPBE0, ctPBE)[26] levels of theory (where applicable: RIJOCSX, def2-TZVPP, def2/J, def2-TZVPP/C, *tightscf*) using state average-CAS(14,14)and CAS(6,6) under the inclusion of 10 singlet, 6 triplet, and 1 quintet root. In case of CASPT2K, intruder states rendered the states' energies despite massive level-shifting unreliable (weight <0.35); the CI-sr-ctPBE calculations proved opposed to CI-sr-ctPBE difficult-to-impossible to converge.

Absorption spectra were additionally computed by TD-DFT (50 roots, Tamm-Dancoff approximation, RIJOCSX, def2-TZVPP, def2/J, *tightscf*, solid state structural parameters) as well as *ab-initio* computations at the DLPNO-STEOM-CCSD/def2-TZVP(-f) level of theory (RIJOCSX, def2-SVP/C, def2/J, *tightscf,* solid state structural parameters).

Molecular orbitals were visualized with IBOView[27] or Chemcraft.[28]

Computation of Diradical Character of 1^flat^:

1.) Diradical character y_0 (and tetraradical character y_1) based on NOON) of LUNO (HONO, respectively), *i.e.* based on occupation number n_{occ}, taking all configurations and their character of CASSCF(14,14), no state averaging, into account.[29] The manuscript shows these y_0 values for comparability with our previous work.[30]

n_{occ}(HONO−1) = 1.93

n_{occ}(HONO) = 1.22

n_{occ}(LUNO) = 0.78

n_{occ}(LUNO+1) = 0.07

y_0 = 0.78 = (1− 1.22)

y_1 = 0.07 = (1− 1.93)

2.) Diradical character y_0 based on NOON of LUNO (HONO, respectively), *i.e.* based on occupation number n_{occ}, taking all configurations and their character of "ideal" CASSCF(2,2), no state averaging, into account:[29]

n_{occ}(HONO) = 1.23

n_{occ}(LUNO) = 0.77

y_0 = 0.77 = (2 − 1.23)

3.) Diradical character y_0 based on twice the weight of the antibonding double excitation configuration in CASSCF(14,14), no state averaging, according to Salem, Yamaguchi and Nakano;[31–33] note that ORCA plots by default the c^2 values, *i.e.* the configuration weight:

$c_{22222220000000} = c_2^2$ = 0.51164

$c_{22222202000000} = c_0^2$ = 0.31658

y_0 = 0.31658 × 2 = 0.63

4.) Diradical character y_0 based on twice the weight of the antibonding double excitation configuration in ideal CASSCF(2,2), no state averaging, according to Salem, Yamaguchi and Nakano;[31–33] note that ORCA plots by default the c^2 values, *i.e.* the configuration weight:

c_2^2 = 0.61519

c_0^2 = 0.38481

y_0 = 0.38481 × 2 = 0.77

5.) Diradical character y_0 based on the weight of the doubly excited configuration according to Schaefer,[34,35] $\frac{2 \times c_0^2}{c_0^2 + c_2^2}$, CASSCF(14,14), no state averaging:

$c_{22222220000000}$ = c_2^2 = 0.51164

$c_{22222202000000}$= c_0^2 = 0.31658

$$\frac{\mathbf{2} \times \boldsymbol{c_0^2}}{\boldsymbol{c_0^2} + \boldsymbol{c_2^2}} = \frac{\mathbf{2} \times \mathbf{0.31658}}{\mathbf{0.31658} \quad + \mathbf{0.51164}} = \mathbf{0.76}$$

6.) Diradical character y_0 based on the weight of the doubly excited configuration according to Schaefer,[34,35] $\frac{2 \times c_0^2}{c_0^2 + c_2^2}$, ideal CASSCF(2,2), no state averaging:

c_2^2 = 0.61519

c_0^2 = 0.38481

$$\frac{\mathbf{2} \times \boldsymbol{c_0^2}}{\boldsymbol{c_0^2} + \boldsymbol{c_2^2}} = \frac{\mathbf{2} \times \mathbf{0.38481}}{\mathbf{0.38481} \quad + \mathbf{0.61519}} = \mathbf{0.77}$$

7.) Diradical character y_0 based on the weight of the doubly excited configuration according to Neese,[34] $y_0 = \frac{2 \times \sqrt{c_0^2 c_2^2}}{c_0^2 + c_2^2}$, CASSCF(14,14), no state averaging; note that ORCA plots by default the c^2 values, *i.e.* the configuration weight:

$c_{22222220000000}$ = c_2^2 = 0.51164

$c_{22222202000000}$= c_0^2 = 0.31658

$$\boldsymbol{y_0} = \frac{\mathbf{2} \times \sqrt{\boldsymbol{c_0^2 c_2^2}}}{\boldsymbol{c_0^2} + \boldsymbol{c_2^2}} = \frac{\mathbf{2} \times \sqrt{(\mathbf{0.31658}) \quad \times (\mathbf{0.51164})}}{(\mathbf{0.31658}) \quad + (\mathbf{0.51164})} = \mathbf{0.97}$$

8.) Diradical character y_0 based on the weight of the doubly excited configuration according to Neese,[34] $y_0 = \frac{2 \times \sqrt{c_0^2 c_2^2}}{c_0^2 + c_2^2}$, ideal CASSCF(2,2), no state averaging; note that ORCA plots by default the c^2 values, *i.e.* the configuration weight:

c_2^2 = 0.61519

c_0^2 = 0.38481

$$\boldsymbol{y_0} = \frac{\mathbf{2} \times \sqrt{\boldsymbol{c_0^2 c_2^2}}}{\boldsymbol{c_0^2} + \boldsymbol{c_2^2}} = \frac{\mathbf{2} \times \sqrt{(\mathbf{0.38481}) \quad (\mathbf{0.61519})}}{(\mathbf{0.38481}) \quad + (\mathbf{0.61519})} = \mathbf{0.97}$$

Table S10: Structural benchmark for **1** (boat conformer), values are given in [°] or [Å].

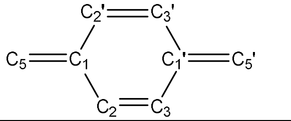	scXRD	ωB97X-V	PBE0-D4	TPSSh-D4
C2-C2‘-C1-C5 (av)	175	171.6	173.1	172.65
C3‘-C1‘-C3 (av)	110.6	112	112.4	112.4
C5-C1-C1‘ (av)	144.35	138.9	142.9	144.2
RMSD(angles)		3.796	1.727	1.711
C1-C1‘	2.7895	2.725	2.725	2.725
C1-C5(av)	1.346	1.349	1.357	1.365
C1-C2(av)	1.4848	1.492	1.475	1.4745
C2-C3(av)	1.3335	1.346	1.351	1.357
RMSD(bond length)		0.033	0.034	0.0360

Table S11: Structural benchmark for **2** (flat conformer), values are given in [°] or [Å].

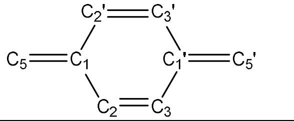	scXRD	ωB97X-V	PBE0-D4	TPSSh-D4
C2-C2'-C1-C5 (av)	178.1	179.7	180	
C3'-C1'-C3 (av)	111.85	112.9	113.7	113.7
C5-C1-C1' (av)	176.05	178.9	180	179.6
RMSD(angles)		1.9820024	2.7468163	102.85205
C1-C1'	2.964	2.968	2.936	2.941
C1-C5(av)	1.371	1.363	1.377	1.386
C1-C2(av)	1.4475	1.466	1.446	1.445
C2-C3(av)	1.3435	1.348	1.355	1.361
RMSD(bond length)		0.0105178	0.0075388	0.0133853

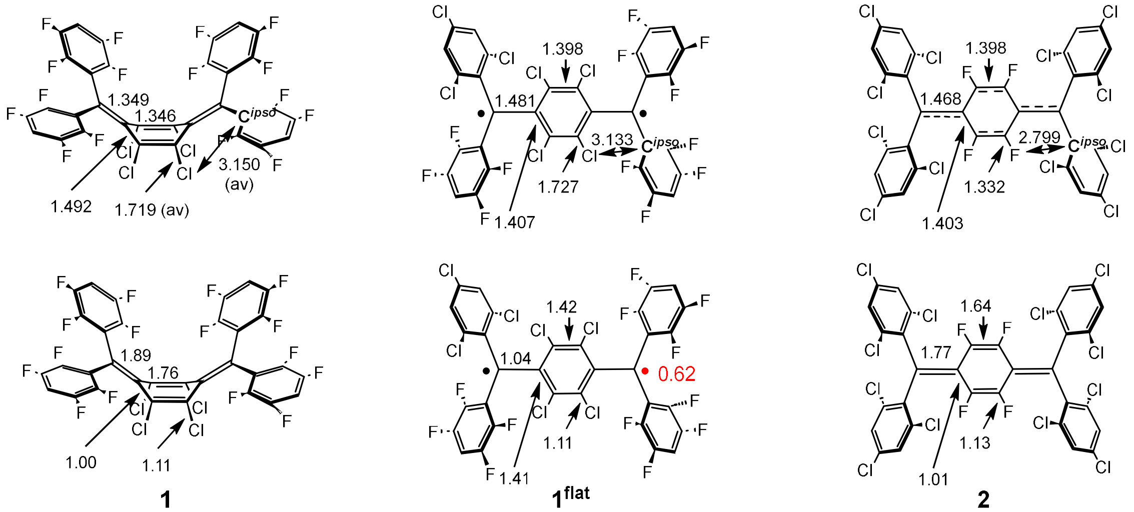

1 $\mathbf{1}^{flat}$ **2**

Figure S81: Computed (ωB97X-V/def2-SVP) structural parameters for **1**, $\mathbf{1}^{flat}$ and **2**; values are given in [Å] (top) and Löwdin atomic spin density for $\mathbf{1}^{flat}$ (given in red) and Mayer bond orders for **1**, $\mathbf{1}^{flat}$ and **2** (ωB97X-V/def2-SVP); values are given in [a.u.] (bottom).

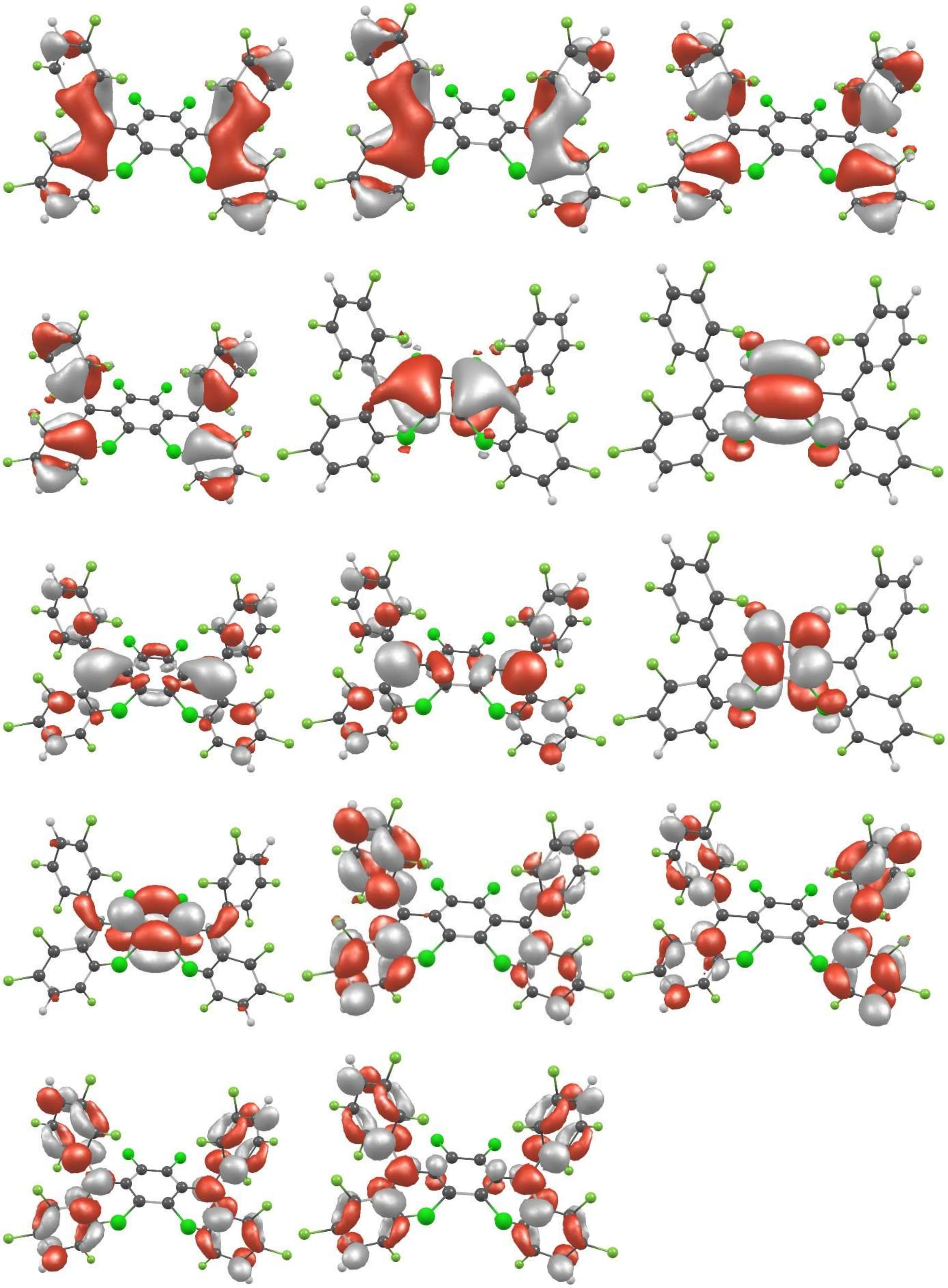

Figure S82: Active space for $\mathbf{1}^{\mathbf{flat}}$ as obtained at the CASSCF(14,14)/def2-TZVPP level of theory with and without state averaging.

ROOT 0: **No State-Averaging** 0.51164 [0]: 22222220000000 0.31658 [8]: 22222202000000

```
    0.00946 [ 58290]: 21222211000010
    0.00911 [   534]: 22221120110000
    0.00884 [  1309]: 22220220020000
    0.00872 [201656]:12222211000001
    0.00812 [    44]: 22222111100000
    0.00756 [   155]: 22222020200000
    0.00569 [  1407]: 22220202020000
    0.00547 [   632]: 22221102110000
    0.00468 [   253]: 22222002200000
    0.00460 [  2860]: 22212211001000
    0.00450 [ 14366]: 22122211000100
    0.00337 [ 19114]: 22112220001100

---------------------------------------------
CAS-SCF STATES FOR BLOCK  0
MULT= 5 NROOTS= 1
---------------------------------------------

ROOT   0:  E=   -4643.9553263637 Eh
    0.55811 [     0]: 22222111100000
    0.25530 [   127]: 22221211010000
    0.01246 [   881]: 22220111120000
    0.01244 [   470]: 22221011210000
    0.00609 [ 43713]: 21222111100010
    0.00575 [163979]: 12222111100001
    0.00365 [ 13652]: 22112111101100
    0.00308 [  1556]: 22212111100100
    0.00307 [  9490]: 22122111101000
    0.00288 [ 44292]: 21212211010010
    0.00284 [164558]: 12221211010001
    0.00262 [   188]: 22221120110000

---------------------------------------------
CAS-SCF STATES FOR BLOCK  1
MULT= 3 NROOTS= 6
---------------------------------------------

ROOT
    0.79297 [     0]: 22222211000000
    0.01415 [   167]: 22222011200000
    0.01411 [   525]: 22221111110000
    0.01062 [  1293]: 22220211020000
    0.00882 [ 57498]: 21222211000010
    0.00830 [199940]: 12222211000001
    0.00577 [   385]: 22221212000000
    0.00517 [ 18811]: 22112211001100
    0.00446 [ 14035]: 22122211001000
    0.00446 [  2741]: 22212211000100
    0.00324 [219832]: 12122211000110
    0.00321 [ 77388]: 21122211001010
    0.00307 [     8]: 22222201010000
    0.00305 [ 62276]: 21212211000110
    0.00303 [204714]: 12212211001010
    0.00295 [204718]: 12212211000101
    0.00288 [ 62274]: 21212211001001
    0.00287 [269556]: 11222211000011
    0.00286 [    12]: 22222201000001
    0.00280 [219830]: 12122211001001
    0.00268 [ 77392]: 21122211000101
    0.00258 [269549]: 11222211001100

ROOT   1:
    0.17534 [  2730]: 22212221000000
    0.11789 [ 14032]: 22122212000000
    0.09386 [     3]: 22222210001000
    0.05909 [  2737]: 22212212000000
    0.04799 [ 14025]: 22122221000000
    0.04341 [    10]: 22222201000100
    0.04118 [     9]: 22222201001000
    0.03589 [ 57496]: 21222211001000
    0.02071 [  2743]: 22212211000001
    0.01950 [ 14037]: 22122211000010
    0.01702 [199938]: 12222211000100
    0.01383 [199937]: 12222211001000
    0.01103 [     4]: 22222210000100
    0.01007 [  2742]: 22212211000010
    0.00880 [199930]: 12222220001000
    0.00776 [199964]: 12222202001000
    0.00758 [  2739]: 22212211010000
    0.00754 [ 57489]: 21222220001000
    0.00751 [  2769]: 22212202000010
    0.00722 [ 57490]: 21222220000100
    0.00697 [ 14031]: 22122220000001
    0.00653 [  2735]: 22212220000010
    0.00551 [ 14038]: 22122211000001
    0.00513 [ 57497]: 21222211000100
    0.00434 [ 57523]: 21222202001000
    0.00389 [ 57524]: 21222202000100
    0.00337 [  2736]: 22212220000001
    0.00337 [  3885]: 22211121110000
    0.00315 [   388]: 22221211001000
    0.00306 [  3114]: 22212021200000
    0.00286 [ 14061]: 22122202010000
    0.00263 [  5178]: 22210221020000
    0.00261 [ 14065]: 22122202000001

ROOT   2:
    0.17291 [ 14025]: 22122221000000
    0.11906 [  2737]: 22212212000000
    0.09363 [     4]: 22222210000100
    0.05886 [ 14032]: 22122212000000
    0.04886 [  2730]: 22212221000000
    0.04420 [     9]: 22222201001000
    0.04078 [    10]: 22222201000100
    0.02844 [ 57497]: 21222211000100
    0.02139 [ 14038]: 22122211000001
    0.02091 [199938]: 12222211000100
    0.01981 [199937]: 12222211001000
    0.01905 [  2742]: 22212211000010
    0.01129 [199931]: 12222220000100
    0.01111 [     3]: 22222210001000
    0.00927 [199965]: 12222202000100
    0.00913 [ 14037]: 22122211000010
    0.00754 [ 14034]: 22122211010000
    0.00721 [ 14064]: 22122202000010
    0.00716 [  2736]: 22212220000001
    0.00624 [ 57489]: 21222220001000
    0.00623 [ 14030]: 22122220000010
    0.00621 [  2743]: 22212211000001
    0.00514 [ 57490]: 21222220000100
    0.00367 [ 14031]: 22122220000001
    0.00343 [ 57523]: 21222202001000
    0.00333 [ 15180]: 22121121110000
    0.00316 [   389]: 22221211000100
    0.00302 [ 14409]: 22122021200000
    0.00290 [  2766]: 22212202010000
    0.00281 [  2770]: 22212202000001
    0.00264 [ 57496]: 21222211001000
    0.00263 [ 57524]: 21222202000100
    0.00260 [ 16473]: 22120221020000

ROOT   3:
    0.22714 [   378]: 22221221000000
    0.18469 [   387]: 22221211010000
    0.16684 [     2]: 22222210010000
    0.08764 [    36]: 22222111100000
    0.03238 [ 57486]: 21222221000000
    0.03190 [199934]: 12222212000000
    0.01286 [    11]: 22222201000010
    0.01167 [  2740]: 22212211001000
    0.01135 [ 14036]: 22122211000100
    0.00897 [     6]: 22222210000001
    0.00750 [   391]: 22221211000001
    0.00697 [  2734]: 22212220000100
    0.00695 [ 14028]: 22122220001000
    0.00621 [ 57495]: 21222211010000
    0.00520 [   854]: 22221011210000
    0.00484 [   762]: 22221021200000
    0.00369 [   188]: 22222010210000
    0.00362 [  1601]: 22220111120000
    0.00340 [199939]: 12222211000010
    0.00323 [ 57499]: 21222211000001
    0.00321 [199933]: 12222220000001
    0.00314 [ 58411]: 21221211010010
    0.00313 [200853]: 12221211010001
    0.00287 [ 57491]: 21222220000010
    0.00275 [ 14062]: 22122202001000
    0.00273 [  2768]: 22212202000100

ROOT   4:
    0.14002 [199927]: 12222221000000
    0.10914 [ 57493]: 21222212000000
    0.07142 [     8]: 22222201010000
    0.06243 [   385]: 22221212000000
    0.05636 [     5]: 22222210000010
    0.05211 [   380]: 22221220010000
    0.04228 [    29]: 22222120100000
    0.03937 [    12]: 22222201000001
    0.03920 [ 14035]: 22122211001000
    0.03900 [  2741]: 22212211000100
    0.02982 [    63]: 22222102100000
    0.02675 [   414]: 22221202010000
    0.01208 [ 57498]: 21222211000010
    0.01136 [199940]: 12222211000001
    0.01064 [ 14029]: 22122220000100
    0.01062 [  2733]: 22212220001000
    0.00887 [  2767]: 22212202001000
    0.00854 [ 14063]: 22122202000100
    0.00645 [199936]: 12222211010000
    0.00445 [199966]: 12222202000010
    0.00350 [ 57488]: 21222220010000
    0.00348 [ 57522]: 21222202010000
    0.00339 [  2740]: 22212211001000
    0.00324 [ 14036]: 22122211000100
    0.00311 [ 57492]: 21222220000001
    0.00283 [   384]: 22221220000001
    0.00276 [201082]: 12221121110000
    0.00252 [   390]: 22221211000010
    0.00250 [200311]: 12222021200000

ROOT   5:
    0.18419 [ 57486]: 21222221000000
    0.14107 [199934]: 12222212000000
    0.08493 [   387]: 22221211010000
    0.06539 [     6]: 22222210000001
    0.05832 [    11]: 22222201000010
    0.04824 [    36]: 22222111100000
    0.04438 [  2740]: 22212211001000
    0.04371 [ 14036]: 22122211000100
    0.03602 [   378]: 22221221000000
    0.01236 [  2734]: 22212220000100
    0.01225 [ 14028]: 22122220001000
    0.01144 [199939]: 12222211000010
    0.01053 [ 57499]: 21222211000001
    0.00915 [ 14062]: 22122202001000
    0.00905 [  2768]: 22212202000100
    0.00552 [     2]: 22222210010000
    0.00411 [ 57495]: 21222211010000
    0.00407 [269535]: 11222212000010
    0.00374 [269509]: 11222221000001
    0.00348 [ 57525]: 21222202000010
    0.00345 [ 58641]: 21221121110000
    0.00326 [ 57870]: 21222021200000
    0.00305 [199933]: 12222220000001
    0.00286 [199963]: 12222202010000
    0.00273 [ 57519]: 21222210000011
    0.00271 [199987]: 12222201000011
    0.00258 [ 59934]: 21220221020000
    0.00254 [ 57491]: 21222220000010

---------------------------------------------
CAS-SCF STATES FOR BLOCK  2
MULT= 1 NROOTS=10
---------------------------------------------

ROOT   0:  E=   -4644.1238092434 Eh
    0.48788 [     0]: 22222220000000
    0.30955 [     8]: 22222202000000
    0.01248 [ 58291]: 21222211000001
    0.01221 [201655]: 12222211000010
    0.00888 [   534]: 22221120110000
```

```
0.00862 [   155]: 22222020200000
0.00788 [   423]: 22221211010000
0.00674 [  2860]: 22212211001000
0.00666 [  1309]: 22220220020000
0.00659 [ 14366]: 22122211000100
0.00570 [   253]: 22222002200000
0.00521 [   632]: 22221102110000
0.00397 [  1407]: 22220202020000
0.00361 [    13]: 22222201000010
0.00311 [ 19114]: 22112220001100
0.00251 [    44]: 22222111100000

ROOT   1:
0.22080 [   414]: 22221221000000
0.16234 [     3]: 22222100010000
0.13107 [     8]: 22222202000000
0.12554 [   423]: 22221211010000
0.08907 [     0]: 22222220000000
0.03764 [    44]: 22222111100000
0.01699 [201650]: 12222212000000
0.00933 [ 14366]: 22122211000100
0.00908 [  2860]: 22212211001000
0.00836 [    13]: 22222201000010
0.00657 [  1296]: 22220222000000
0.00534 [   427]: 22221211000001
0.00467 [201655]: 12222211000010
0.00432 [   798]: 22221021200000
0.00416 [ 58287]: 21222211010000
0.00347 [    21]: 22222200020000
0.00325 [   203]: 22222010210000
0.00293 [   890]: 22221011210000
0.00273 [ 58291]: 21222211000001

ROOT   2:
0.11718 [  2857]: 22212212000000
0.10952 [  2850]: 22212221000000
0.09397 [ 14355]: 22122221000000
0.07968 [ 14362]: 22122212000000
0.06824 [     4]: 22222210001000
0.06752 [    11]: 22222201001000
0.04451 [ 58288]: 21222211001000
0.03528 [201653]: 12222211001000
0.03069 [     5]: 22222210000100
0.02780 [  2862]: 22212211000010
0.02390 [    12]: 22222201000100
0.02151 [  2863]: 22212211000001
0.01984 [ 58289]: 21222211000100
0.01903 [ 14367]: 22122211000010
0.01854 [ 14368]: 22122211000001
0.01219 [201654]: 12222211000100
0.00826 [  2859]: 22212211010000
0.00711 [ 14364]: 22122211010000
0.00368 [   424]: 22221211001000

ROOT   3:
0.11618 [ 14362]: 22122212000000
0.10870 [ 14355]: 22122221000000
0.09483 [  2850]: 22212221000000
0.08087 [  2857]: 22212212000000
0.06754 [     5]: 22222210000100
0.06714 [    12]: 22222201000100
0.04713 [201654]: 12222211000100
0.03214 [ 58289]: 21222211000100
0.03149 [     4]: 22222210001000
0.02611 [ 14367]: 22122211000010
0.02407 [    11]: 22222201001000
0.02283 [ 14368]: 22122211000001
0.01983 [  2863]: 22212211000001
0.01805 [  2862]: 22212211000010
0.01726 [201653]: 12222211001000
0.01517 [ 58288]: 21222211001000
0.00821 [ 14364]: 22122211010000
0.00714 [  2859]: 22212211010000
0.00369 [   425]: 22221211000100

ROOT   4:
0.19661 [ 58285]: 21222212000000
0.16535 [201643]: 12222221000000
0.08521 [     6]: 22222210000010
0.08113 [ 14365]: 22122211001000
0.08092 [  2861]: 22212211000100
0.06117 [    14]: 22222201000001
0.03237 [    10]: 22222201010000
0.02485 [ 58290]: 21222211000010
0.02075 [201656]: 12222211000001
0.01486 [201652]: 12222211010000
0.01345 [     1]: 22222211000000
0.00732 [  2860]: 22212211001000
0.00645 [ 14366]: 22122211000100
0.00619 [   421]: 22221212000000
0.00514 [   426]: 22221211000010
0.00419 [127886]: 20222212000010
0.00348 [ 58733]: 21222012200000
0.00345 [414590]: 02222221000001
0.00344 [202798]: 12221121110000
0.00337 [ 59504]: 21221112110000
0.00330 [ 58310]: 21222210000020
0.00317 [206387]: 12212221000100
0.00308 [221501]: 12122221001000
0.00291 [202027]: 12222021200000
0.00286 [204091]: 12220221020000
0.00275 [201704]: 12222201000002
0.00268 [ 78163]: 21122212001000
0.00262 [ 63049]: 21212212000100
0.00251 [ 60797]: 21220212020000

ROOT   5:
0.20200 [ 58278]: 21222221000000
0.17820 [201650]: 12222212000000
0.07813 [  2860]: 22212211001000
0.07798 [     7]: 22222210000001
0.07757 [ 14366]: 22122211000100
0.06868 [    13]: 22222201000010
0.02917 [   423]: 22221211010000
0.01994 [201655]: 12222211000010
0.01830 [ 58291]: 21222211000001
0.01341 [   414]: 22221221000000
0.01094 [ 58287]: 21222211010000
0.00867 [    44]: 22222111100000
0.00794 [     8]: 22222202000000
0.00402 [ 59433]: 21221121110000
0.00357 [ 58662]: 21222021200000
0.00324 [127859]: 20222221000010
0.00318 [202098]: 12222012200000
0.00315 [ 60726]: 21220221020000
0.00302 [202869]: 12221112110000
0.00290 [414617]: 02222212000001
0.00278 [   427]: 22221211000001
ROOT   6:
0.62365 [     1]: 22222211000000
0.08909 [   421]: 22221212000000
0.06036 [201643]: 12222221000000
0.04357 [    10]: 22222201010000
0.01282 [     6]: 22222210000010
0.00895 [   182]: 22222011200000
0.00691 [   450]: 22221202010000
0.00667 [    14]: 22222201000001
0.00644 [   561]: 22221111110000
0.00641 [    37]: 22222120100000
0.00468 [ 14359]: 22122220000100
0.00424 [  2853]: 22212220001000
0.00394 [271219]: 11222222000000
0.00306 [ 19141]: 22112211001100
0.00301 [  1336]: 22220211020000
0.00260 [    34]: 22222200000011

ROOT   7:
0.49826 [    36]: 22222121000000
0.23198 [    45]: 22222111010000
0.05354 [     2]: 22222210100000
0.04651 [   422]: 22221211100000
0.00875 [    49]: 22222111000001
0.00858 [   526]: 22221122000000
0.00488 [    83]: 22222101020000
0.00339 [ 58433]: 21222111010010
0.00336 [201799]: 12222111010001
0.00300 [ 19379]: 22112121001100
0.00287 [ 58395]: 21222121000010
0.00285 [201761]: 12222121000001

ROOT   8:
0.40400 [    44]: 22222111100000
0.15776 [     8]: 22222202000000
0.15062 [     0]: 22222220000000
0.06345 [   423]: 22221211010000
0.02735 [ 58278]: 21222221000000
0.00927 [     7]: 22222210000001
0.00766 [  1665]: 22220111120000
0.00691 [201650]: 12222212000000
0.00488 [   890]: 22221011210000
0.00488 [ 58428]: 21222111100010
0.00459 [   554]: 22221112100000
0.00453 [201794]: 12222111100001
0.00401 [   632]: 22221102110000
0.00367 [  2860]: 22212211001000
0.00364 [ 14366]: 22122211000100
0.00363 [    13]: 22222201000010
0.00320 [     3]: 22222100010000
0.00303 [   414]: 22221221000000
0.00286 [   253]: 22222002200000
0.00266 [ 19470]: 22112111101100
0.00254 [ 14503]: 22122111101000
0.00254 [  2999]: 22212111100100
0.00252 [    78]: 22222101110000

ROOT   9:
0.46748 [     2]: 22222210100000
0.25703 [   422]: 22221211100000
0.08448 [    36]: 22222121000000
0.01706 [    45]: 22222111010000
0.00759 [  1324]: 22220212100000
0.00631 [ 58286]: 21222211100000
0.00479 [   432]: 22221210100010
0.00419 [    16]: 22222200110000
0.00355 [ 59198]: 21221211100010
0.00335 [202564]: 12221211100001
0.00281 [ 19161]: 22112210101100
0.00261 [   460]: 22221201100001
0.00251 [  1356]: 22220210120000
```

Figure S83: Configurations of states for **1^{flat}** as obtained at the saCASSCF(14,14)/def2-TZVPP level of theory.

```
--------------------------------------------
CAS-SCF STATES FOR BLOCK  0 MULT= 5 NROOTS= 1
--------------------------------------------

ROOT   0:
0.85922 [     0]: 22222111100000
0.05793 [   127]: 22221211010000
0.00593 [163892]: 12222211010000
0.00265 [163978]: 12222111100010
0.00264 [  1555]: 22212111101000
0.00264 [  9491]: 22122111100100

--------------------------------------------
CAS-SCF STATES FOR BLOCK  1 MULT= 3 NROOTS= 6
--------------------------------------------
```

ROOT 0:
0.91626 [0]: 22222211000000
0.00636 [525]: 22221111110000
0.00421 [167]: 22222011200000
0.00334 [8]: 22222201010000
0.00324 [199939]: 12222211000010
0.00304 [14036]: 22122211000100
0.00300 [2740]: 22212211001000
0.00283 [57499]: 21222211000001

ROOT 1:
0.53184 [28]: 22222121000000
0.14499 [14025]: 22122221000000
0.09590 [2737]: 22212212000000
0.04637 [37]: 22222111010000
0.03515 [3]: 22222210001000
0.02332 [10]: 22222201000100
0.00575 [57497]: 21222211000100
0.00552 [386]: 22221211100000
0.00521 [14034]: 22122211010000
0.00459 [41]: 22222111000001
0.00416 [1]: 22222101100000
0.00345 [199937]: 12222211001000
0.00342 [2742]: 22212211000010
0.00297 [388]: 22221211001000
0.00262 [519]: 22221112010000

ROOT 2:
0.62771 [378]: 22221221000000
0.14405 [2]: 22222100010000
0.04873 [57493]: 21222212000000
0.04435 [387]: 22221211010000
0.01820 [199936]: 12222211010000
0.00928 [36]: 22222111100000
0.00740 [11]: 22222201000010
0.00671 [391]: 22221211000001
0.00653 [2741]: 22212211000100
0.00603 [14035]: 22122211001000
0.00520 [2733]: 22212220001000
0.00503 [14029]: 22122220000100
0.00280 [762]: 22221021200000

ROOT 3:
0.39854 [2730]: 22212221000000
0.24430 [14032]: 22122212000000
0.10656 [4]: 22222210000100
0.06926 [9]: 22222201001000
0.01461 [57496]: 21222211001000
0.01235 [2739]: 22212211010000
0.00991 [199938]: 12222211000100
0.00987 [14037]: 22122211000010
0.00894 [35]: 22222112000000
0.00880 [389]: 22221211000100
0.00711 [2743]: 22212211000001
0.00583 [57490]: 21222220000100
0.00513 [14061]: 22122202010000
0.00399 [2735]: 22212220000010
0.00396 [199930]: 12222220001000
0.00350 [381]: 22221220001000
0.00345 [57524]: 21222202000100
0.00330 [14031]: 22122220000001
0.00327 [2769]: 22212202000010
0.00314 [14027]: 22122220010000
0.00291 [3885]: 22211121110000
0.00269 [415]: 22221202001000

ROOT 4:
0.27280 [28]: 22222121000000
0.23856 [14025]: 22122221000000
0.15202 [2737]: 22212212000000
0.07553 [3]: 22222210001000
0.05301 [10]: 22222201000100
0.03481 [37]: 22222111010000
0.03080 [1]: 22222210100000
0.00947 [57497]: 21222211000100
0.00704 [14034]: 22122211010000
0.00690 [2742]: 22212211000010
0.00612 [199937]: 12222211001000
0.00595 [388]: 22221211001000
0.00532 [386]: 22221211100000
0.00483 [14038]: 22122211000001
0.00379 [57489]: 21222220001000
0.00327 [2766]: 22212202010000

ROOT 5:
0.41243 [385]: 22221212000000
0.17147 [57486]: 21222221000000
0.13114 [8]: 22222201010000
0.04415 [5]: 22222210000010
0.02365 [380]: 22221220010000
0.02075 [14036]: 22122211000100
0.02062 [2740]: 22212211001000
0.01425 [199934]: 12222212000000
0.01341 [12]: 22222201000001
0.01264 [29]: 22222120100000
0.00916 [199929]: 12222220010000
0.00586 [199963]: 12222202010000
0.00542 [57495]: 21222211010000
0.00520 [414]: 22221202010000
0.00515 [63]: 22222102100000
0.00466 [14028]: 22122220001000
0.00446 [2734]: 22212220000100
0.00442 [2768]: 22212202000100
0.00416 [384]: 22221220000001
0.00392 [14062]: 22122202001000
0.00283 [390]: 22221211000010

CAS-SCF STATES FOR BLOCK 2 MULT= 1 NROOTS=10

ROOT 0: E= -4656.8879811569 Eh
0.65057 [0]: 22222220000000
0.26955 [8]: 22222202000000
0.00630 [423]: 22221211010000
0.00524 [201656]: 12222211000001
0.00470 [58290]: 21222211000010
0.00426 [534]: 22221120110000
0.00422 [2861]: 22212211000100
0.00392 [14365]: 22122211001000
0.00287 [155]: 22222020200000

ROOT 1:
0.40112 [8]: 22222202000000
0.18283 [414]: 22221221000000
0.16621 [0]: 22222220000000
0.10053 [3]: 22222210010000
0.01765 [423]: 22221211010000
0.01698 [201643]: 12222221000000
0.01487 [58285]: 21222212000000
0.00850 [44]: 22222111100000
0.00756 [14365]: 22122211001000
0.00697 [2861]: 22212211000100
0.00537 [13]: 22222201000010
0.00444 [201652]: 12222211010000
0.00276 [1296]: 22220222000000
0.00269 [21]: 22222200020000

ROOT 2: 1
0.76149 [1]: 22222211000000
0.06344 [421]: 22221212000000
0.03440 [10]: 22222201010000
0.01908 [58278]: 21222221000000
0.01181 [37]: 22222120100000
0.01072 [201650]: 12222212000000
0.00918 [450]: 22221202010000
0.00902 [6]: 22222210000010
0.00901 [416]: 22221220010000
0.00635 [14358]: 22122220001000
0.00577 [2854]: 22212220000100
0.00444 [71]: 22222102100000

ROOT 3:
0.54117 [36]: 22222121000000
0.13061 [14355]: 22122221000000
0.10288 [2857]: 22212212000000
0.05770 [45]: 22222111010000
0.02942 [4]: 22222210001000
0.02543 [12]: 22222201000100
0.00846 [58289]: 21222211000100
0.00729 [14364]: 22122211010000
0.00568 [49]: 22222111000001
0.00535 [2862]: 22212211000010
0.00487 [201653]: 12222211001000
0.00473 [424]: 22221211001000
0.00335 [14368]: 22122211000001
0.00275 [526]: 22221122000000
0.00251 [422]: 22221211100000

ROOT 4:
0.36368 [2850]: 22212221000000
0.27763 [14362]: 22122212000000
0.10028 [5]: 22222210000100
0.08082 [11]: 22222201001000
0.02290 [58288]: 21222211001000
0.01884 [2859]: 22212211010000
0.01631 [14367]: 22122211000010
0.01436 [425]: 22221211000100
0.01428 [201654]: 12222211000100
0.01062 [2863]: 22212211000001
0.00673 [43]: 22222112000000
0.00281 [4005]: 22211121110000
0.00275 [9]: 22222201100000

ROOT 5:
0.27094 [36]: 22222121000000
0.22838 [14355]: 22122221000000
0.18430 [2857]: 22212212000000
0.06594 [4]: 22222210001000
0.05770 [12]: 22222201000100
0.04223 [45]: 22222111010000
0.01524 [58289]: 21222211000100
0.01141 [2862]: 22212211000010
0.01139 [14364]: 22122211010000
0.01124 [2]: 22222210100000
0.00947 [424]: 22221211001000
0.00903 [201653]: 12222211001000
0.00720 [14368]: 22122211000001
0.00294 [49]: 22222111000001

ROOT 6:
0.28426 [421]: 22221212000000
0.24916 [58278]: 21222221000000
0.08643 [10]: 22222201010000
0.08071 [201650]: 12222212000000
0.07114 [6]: 22222210000010
0.04537 [14366]: 22122211000100
0.04534 [2860]: 22212211001000
0.02441 [14]: 22222201000001
0.01140 [58287]: 21222211010000
0.01018 [1]: 22222211000000
0.00576 [426]: 22221211000010
0.00418 [201655]: 12222211000010
0.00411 [58291]: 21222211000001
0.00301 [416]: 22221220010000
0.00262 [527]: 22221121100000

ROOT 7:
0.36532 [414]: 22221221000000
0.15107 [8]: 22222202000000
0.06627 [58285]: 21222212000000
0.06624 [0]: 22222220000000
0.05459 [201643]: 12222221000000
0.04794 [423]: 22221211010000
0.04754 [3]: 22222210010000
0.03027 [44]: 22222111100000
0.02654 [7]: 22222210000001
0.02293 [2861]: 22212211000100
0.02071 [14365]: 22122211001000
0.01577 [201652]: 12222211010000

0.01008 [13]: 22222201000010
0.00582 [427]: 22221211000001

ROOT 8:
0.77436 [43]: 22222112000000
0.04558 [38]: 22222120010000
0.03199 [9]: 22222201100000
0.01411 [5]: 22222210000100
0.01041 [415]: 22221220100000
0.00979 [528]: 22221121010000
0.00891 [449]: 22221202100000
0.00798 [58390]: 21222122000000
0.00745 [72]: 22222102010000
0.00703 [11]: 22222201001000
0.00466 [14470]: 22122121001000
0.00431 [2966]: 22212121000100
0.00409 [42]: 22222120000001

ROOT 9:
0.70409 [2]: 22222210100000
0.12438 [422]: 22221211100000
0.02772 [201651]: 12222211100000
0.02222 [14355]: 22122221000000
0.01273 [12]: 22222201000100
0.01205 [4]: 22222210001000
0.01032 [2857]: 22212212000000
0.00977 [36]: 22222121000000
0.00533 [16]: 22222200110000
0.00303 [432]: 22221210100010

Figure S84: Configurations of states for **1**$^{\mathbf{flat}}$ as obtained at the CI-sr-ctPBE0 saCASSCF(14,14)/def2-TZVPP level of theory.

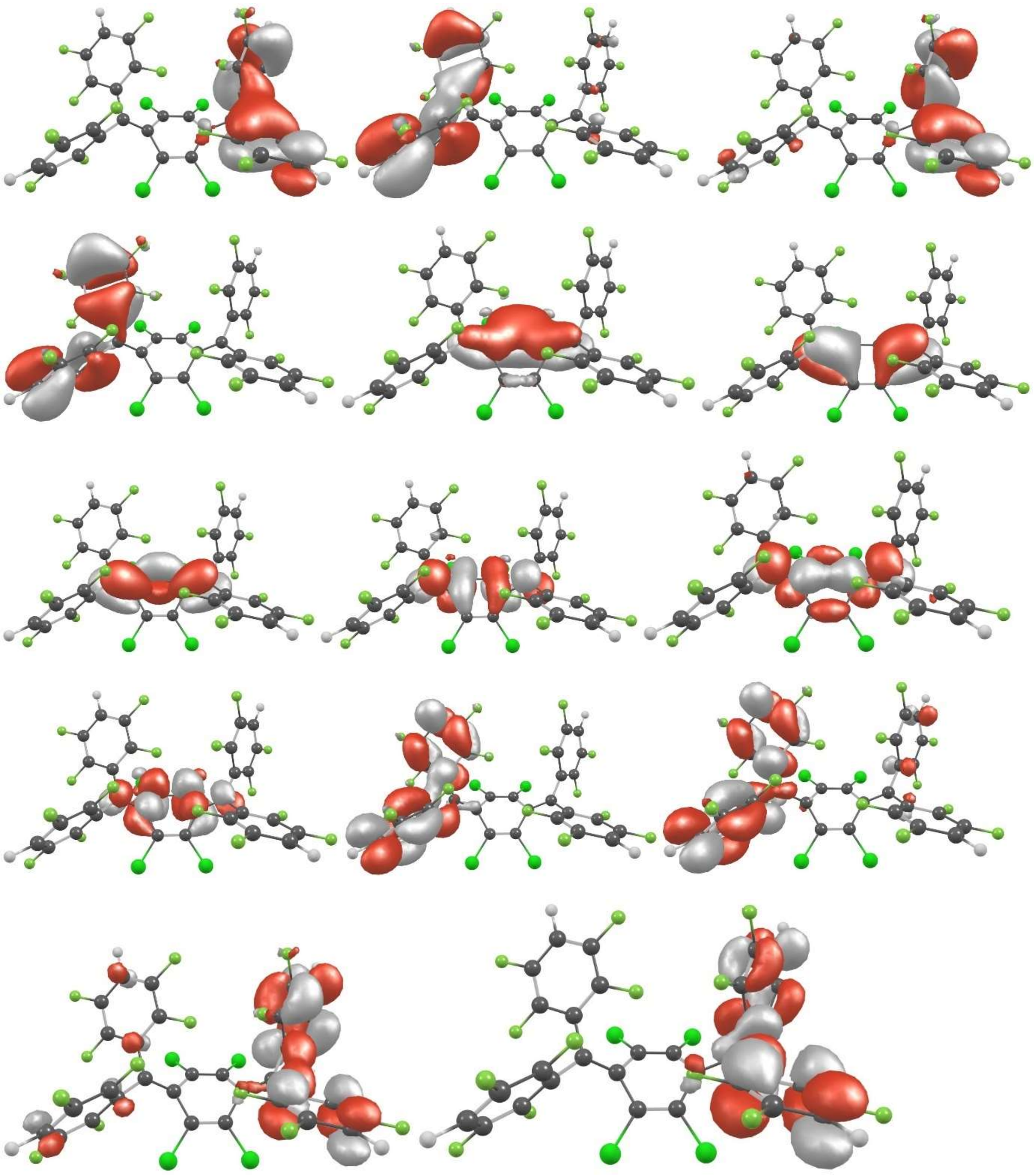

Figure S85: Active space for **1** as obtained at the saCASSCF(14,14)/def2-TZVPP level of theory.

```
ROOT   0:  No state averaging
      0.79977 [     0]: 22222220000000
      0.02257 [    44]: 22222111100000
      0.01668 [   423]: 22221211010000
      0.01289 [ 63037]: 21212220001100
      0.01160 [221524]: 12122220000011
      0.01107 [     8]: 22222202000000
      0.00721 [   155]: 22222020200000
      0.00592 [   527]: 22221121100000
      0.00559 [   148]: 22222022000000
      0.00550 [    51]: 22222110110000
      0.00506 [  1309]: 22220220020000
      0.00497 [    15]: 22222200200000
      0.00442 [  7608]: 22202220002000
      0.00400 [127872]: 20222220002000
      0.00400 [  1296]: 22220222000000
      0.00370 [  1298]: 22220221010000
      0.00360 [ 34237]: 22022220000002
      0.00351 [414609]: 02222220000020
      0.00351 [414611]: 02222220000002
      0.00347 [  7612]: 22202220000200
      0.00330 [   534]: 22221120110000
      0.00323 [ 34235]: 22022220000020
      0.00320 [   150]: 22222021010000
      0.00308 [   428]: 22221210200000
      0.00277 [   434]: 22221210020000
      0.00276 [    21]: 22222200020000
      0.00266 [127876]: 20222220000200
----------------------------------------------
CAS-SCF STATES FOR BLOCK  0
MULT= 5 NROOTS= 1
----------------------------------------------
ROOT   0:
      0.77957 [     0]: 22222111100000
      0.03525 [   127]: 22221211010000
      0.01763 [   188]: 22221120110000
      0.01493 [   881]: 22220111120000
      0.01060 [ 47874]: 21212111101010
      0.00891 [ 27084]: 22022111100200
      0.00764 [     6]: 22222110110000
      0.00499 [ 61521]: 21122111100200
      0.00386 [   280]: 22221102110000
      0.00385 [   182]: 22221121100000
      0.00384 [     9]: 22222110100010
```

```
      0.00382 [   861]: 22220112110000
      0.00375 [  5507]: 22202111100020
      0.00360 [  5500]: 22202111102000
      0.00322 [ 13653]: 22112111101010
      0.00288 [107788]: 20222111102000
      0.00268 [364959]: 02222111100002

---------------------------------------------
CAS-SCF STATES FOR BLOCK  1
MULT= 3 NROOTS= 6
---------------------------------------------
ROOT   0:
      0.61842 [     0]: 22222211000000
      0.13734 [    29]: 22222120100000
      0.04934 [   380]: 22221220010000
      0.01408 [    63]: 22222102100000
      0.01123 [   167]: 22220011200000
      0.00981 [   378]: 22221221000000
      0.00834 [  1293]: 22220211020000
      0.00803 [ 62273]: 21212211001010
      0.00688 [ 33922]: 22022211000200
      0.00602 [   525]: 22221111110000
      0.00454 [    28]: 22222121000000
      0.00384 [ 77390]: 21122211000200
      0.00305 [   414]: 22221202010000
      0.00273 [  7515]: 22202211000020
      0.00271 [  7508]: 22202211002000
      0.00266 [     7]: 22222201100000
      0.00252 [   492]: 22221121010000

ROOT   1:
      0.39975 [     1]: 22222210100000
      0.35266 [    28]: 22222121000000
      0.03108 [    29]: 22222120100000
      0.01270 [   161]: 22220012100000
      0.01193 [    69]: 22222101200000
      0.01088 [    30]: 22222120010000
      0.00954 [   379]: 22221220100000
      0.00724 [    36]: 22222111100000
      0.00640 [  1313]: 22220210120000
      0.00601 [  1510]: 22220121020000
      0.00516 [ 62293]: 21212210101010
      0.00490 [  1288]: 22220211110000
      0.00463 [ 62511]: 21212121001010
      0.00450 [ 33942]: 22022210100200
      0.00399 [ 34139]: 22022121000200
      0.00388 [   387]: 22221211010000
      0.00281 [   380]: 22221220010000
      0.00275 [   378]: 22221221000000
      0.00270 [  2735]: 22212220000010
      0.00267 [   530]: 22221111020000
      0.00252 [ 77410]: 21122210100200

ROOT   2:
      0.35326 [   378]: 22221221000000
      0.20400 [   380]: 22221220010000
      0.12951 [     2]: 22222100010000
      0.08646 [    29]: 22222120100000
      0.01234 [   525]: 22221111110000
      0.00992 [   386]: 22221211100000
      0.00759 [     0]: 22222211000000
      0.00730 [   379]: 22221220100000
      0.00531 [    37]: 22222111010000
      0.00512 [   382]: 22221220000100
      0.00502 [   518]: 22221112100000
      0.00436 [ 63799]: 21211221001010
      0.00393 [ 35371]: 22021221000200
      0.00323 [   762]: 22221021200000
      0.00316 [   757]: 22221022010000
      0.00316 [   419]: 22221201200000
      0.00304 [     4]: 22222210000100
      0.00299 [   492]: 22221121010000
      0.00263 [ 63833]: 21211220011010
      0.00253 [   440]: 22221200210000

ROOT   3:
      0.23675 [  2733]: 22212220001000
      0.09559 [  2730]: 22212221000000
      0.08862 [  2735]: 22212220000010
      0.07552 [ 57491]: 21222220000010
      0.04550 [  2731]: 22212220100000
      0.03662 [ 57489]: 21222220001000
      0.03609 [     3]: 22222210001000
      0.03587 [ 57486]: 21222221000000
      0.02474 [ 14030]: 22122220000010
      0.02243 [    31]: 22222120001000
      0.01771 [  2732]: 22212220010000
      0.01680 [ 57487]: 21222220100000
      0.01470 [     5]: 22222210000010
      0.01423 [ 14028]: 22122220001000
      0.01341 [ 14025]: 22122221000000
      0.01095 [    33]: 22222120000010
      0.00971 [  2878]: 22212111101000
      0.00613 [ 57488]: 21222220010000
      0.00599 [ 14026]: 22122220100000
      0.00532 [  2767]: 22212202001000
      0.00418 [  2880]: 22212111100010
      0.00359 [  5213]: 22210220021000
      0.00305 [ 57636]: 21222111100010
      0.00268 [ 40499]: 22012220001200
      0.00254 [   380]: 22221220010000

ROOT   4:
      0.25377 [ 57489]: 21222220001000
      0.15219 [  2735]: 22212220000010
      0.12001 [  2733]: 22212220001000
      0.11625 [ 57491]: 21222220000010
      0.06858 [ 14028]: 22122220001000
      0.02987 [ 14030]: 22122220000010
      0.02289 [  2730]: 22212221000000
      0.01123 [     5]: 22222210000010
      0.00855 [ 57634]: 21222111101000
      0.00706 [     3]: 22222210001000
      0.00621 [  2731]: 22212220100000
      0.00597 [  2880]: 22212111100010
      0.00587 [    33]: 22222120000010
      0.00466 [101473]: 21022220001200
      0.00447 [ 57523]: 21222202001000
      0.00443 [ 57486]: 21222221000000
      0.00414 [ 57636]: 21222111100010
      0.00407 [  2878]: 22212111101000
      0.00366 [ 59969]: 21220220021000
      0.00334 [  2769]: 22212202000010
      0.00304 [ 69058]: 21202220001020
      0.00255 [    31]: 22222120001000

ROOT   5:
      0.55569 [ 14029]: 22122220000100
      0.15778 [ 57490]: 21222220000100
      0.03055 [ 14027]: 22122220010000
      0.01954 [ 14174]: 22122111100100
      0.01194 [   382]: 22221220000100
      0.01115 [    32]: 22222120000100
      0.01109 [     4]: 22222210000100
      0.01021 [ 14063]: 22122202000100
      0.00964 [ 84171]: 21112220001110
      0.00875 [ 16509]: 22120220020100
      0.00857 [ 57488]: 21222220010000
      0.00561 [ 57635]: 21222111100100
      0.00530 [ 16475]: 22120221010100
      0.00523 [ 14949]: 22121211010100
      0.00510 [ 14025]: 22122221000000
      0.00508 [ 14432]: 22122020200100
      0.00436 [ 14028]: 22122220001000
      0.00435 [ 14089]: 22122200200100
      0.00425 [ 14406]: 22122022000100
      0.00415 [199931]: 12222220000100
      0.00331 [150729]: 20122220002100
      0.00311 [ 14983]: 22121210020100
      0.00286 [ 57524]: 21222202000100
      0.00251 [ 14026]: 22122220100000
      0.00250 [ 59970]: 21220220020100

---------------------------------------------
CAS-SCF STATES FOR BLOCK  2
MULT= 1 NROOTS=10
---------------------------------------------

ROOT   0:
      0.79397 [     0]: 22222220000000
      0.02692 [    44]: 22222111100000
      0.01410 [     8]: 22222202000000
      0.01120 [  1309]: 22220220020000
      0.00995 [ 63038]: 21212220001010
      0.00882 [ 34232]: 22022220000200
      0.00731 [  1298]: 22220221010000
      0.00714 [   423]: 22221211010000
      0.00699 [   155]: 22222020200000
      0.00600 [    15]: 22222200200000
      0.00590 [   148]: 22222022000000
      0.00493 [ 78155]: 21122220000200
      0.00414 [   434]: 22221210020000
      0.00335 [  7608]: 22202220002000
      0.00325 [  7615]: 22202220000020
      0.00301 [ 19115]: 22112220001010
      0.00283 [127872]: 20222220002000

ROOT   1:
      0.24421 [     8]: 22222202000000
      0.15791 [     2]: 22222210100000
      0.12921 [    36]: 22222121000000
      0.07005 [    44]: 22222111100000
      0.03829 [   148]: 22222022000000
      0.03716 [    15]: 22222200200000
      0.02359 [   534]: 22221120110000
      0.02246 [   415]: 22221220100000
      0.01879 [   423]: 22221211010000
      0.01553 [     9]: 22222201100000
      0.01132 [   527]: 22221121100000
      0.01067 [    38]: 22222120010000
      0.01052 [   155]: 22222020200000
      0.01014 [   421]: 22221212000000
      0.00574 [  1296]: 22220222000000
      0.00569 [    51]: 22222110110000
      0.00457 [     1]: 22222211000000
      0.00400 [    43]: 22222112000000
      0.00381 [   149]: 22222021100000
      0.00376 [   414]: 22221221000000
      0.00357 [   434]: 22221210020000
      0.00342 [  1298]: 22220221010000
      0.00328 [ 63136]: 21212202001010
      0.00309 [   632]: 22221102110000
      0.00308 [   449]: 22221202100000
      0.00277 [ 34330]: 22022202000200
      0.00273 [    21]: 22222200020000
      0.00252 [   150]: 22222021010000

ROOT   2:  eV  52246.0 cm**-1
      0.26836 [   414]: 22221221000000
      0.15885 [     1]: 22222211000000
      0.06027 [   526]: 22221122000000
      0.05293 [201643]: 12222221000000
      0.04816 [     3]: 22222210010000
      0.04591 [   422]: 22221211100000
      0.04099 [   429]: 22221210110000
      0.03165 [    43]: 22222112000000
      0.03156 [   528]: 22221121010000
      0.02209 [    16]: 22222200110000
      0.01845 [    45]: 22222111010000
      0.00767 [     9]: 22222201100000
      0.00602 [  2850]: 22212221000000
      0.00561 [   421]: 22221212000000
      0.00489 [   455]: 22221201200000
      0.00450 [  1304]: 22220220110000
      0.00419 [   539]: 22221120020000
      0.00392 [   527]: 22221121100000
      0.00372 [   561]: 22221111110000
      0.00369 [   415]: 22221220100000
      0.00333 [ 64591]: 21211221001010
      0.00326 [   423]: 22221211010000
      0.00312 [     6]: 22222210000010
```

0.00296 [201644]: 12222220100000
0.00292 [35785]: 22021221000200
0.00281 [1336]: 22220211020000
0.00275 [631]: 22221102200000
0.00260 [554]: 22221112100000

ROOT 3:
0.51447 [1]: 22222211000000
0.09596 [414]: 22221221000000
0.03338 [9]: 22222201100000
0.03097 [2850]: 22212221000000
0.02474 [526]: 22221122000000
0.02142 [422]: 22221211100000
0.01692 [528]: 22221121010000
0.01112 [429]: 22221210110000
0.00901 [3]: 22222210010000
0.00867 [6]: 22222210000010
0.00830 [201643]: 12222221000000
0.00817 [14355]: 22122221000000
0.00598 [4]: 22222210001000
0.00588 [37]: 22222120100000
0.00584 [2855]: 22212220000010
0.00538 [63065]: 21212211001010
0.00529 [2851]: 22212220100000
0.00517 [34259]: 22022211000200
0.00515 [16]: 22222200110000
0.00514 [1336]: 22220211020000
0.00492 [182]: 22220011200000
0.00479 [14359]: 22122220000100
0.00453 [58281]: 21222220001000
0.00427 [36]: 22222121000000
0.00409 [421]: 22221212000000
0.00402 [58278]: 21222221000000
0.00345 [455]: 22221201200000
0.00339 [45]: 22222111010000
0.00324 [539]: 22221120020000
0.00289 [78182]: 21122211000200
0.00278 [201644]: 12222220100000

ROOT 4:
0.19845 [2850]: 22212221000000
0.06575 [4]: 22222210001000
0.06305 [2860]: 22212211001000
0.05357 [58278]: 21222221000000
0.04497 [2857]: 22212212000000
0.03870 [2862]: 22212211000010
0.02120 [58288]: 21222211001000
0.02019 [2851]: 22212220100000
0.01991 [6]: 22222210000010
0.01713 [58290]: 21222211000010
0.01475 [2858]: 22212211100000
0.01347 [11]: 22222201001000
0.01311 [2963]: 22212121100000
0.01308 [58285]: 21222212000000
0.01188 [14355]: 22122221000000
0.00969 [2971]: 22212120101000
0.00864 [39]: 22222120001000
0.00844 [13]: 22222201000010
0.00821 [2965]: 22212121001000
0.00705 [14365]: 22122211001000
0.00620 [46]: 22222111001000
0.00609 [2973]: 22212120100010
0.00566 [14367]: 22122211000010
0.00562 [2859]: 22212211010000
0.00548 [2967]: 22212121000010
0.00544 [14362]: 22122212000000
0.00521 [58286]: 21222211100000
0.00468 [148]: 22222022000000
0.00465 [58279]: 21222220100000
0.00460 [2866]: 22212210101000
0.00446 [2962]: 22212122000000
0.00442 [15]: 22222200200000
0.00441 [48]: 22222111000010
0.00423 [9]: 22222201100000
0.00391 [2868]: 22212210100010
0.00373 [58391]: 21222121100000
0.00355 [3]: 22222210010000

0.00339 [201643]: 12222221000000
0.00337 [52]: 22222110101000
0.00337 [44]: 22222111100000
0.00319 [2990]: 22212112100000
0.00313 [58399]: 21222120101000
0.00311 [2969]: 22212120200000
0.00301 [3734]: 22211221010000
0.00300 [41]: 22222120000010
0.00296 [3746]: 22211220011000
0.00292 [43]: 22222112000000
0.00288 [58393]: 21222121001000
0.00285 [36]: 22222121000000
0.00274 [58401]: 21222120100010
0.00251 [7593]: 22202221001000

ROOT 5:
0.19180 [415]: 22221220100000
0.11381 [421]: 22221212000000
0.05824 [423]: 22221211010000
0.04492 [527]: 22221121100000
0.04360 [10]: 22222201010000
0.04163 [534]: 22221120110000
0.04047 [15]: 22222200200000
0.03869 [44]: 22222111100000
0.03137 [148]: 22222022000000
0.03011 [38]: 22222120010000
0.02896 [201644]: 12222220100000
0.02360 [2]: 22222210100000
0.02269 [155]: 22222020200000
0.02015 [51]: 22222110110000
0.01110 [1296]: 22220222000000
0.00831 [149]: 22222021100000
0.00548 [422]: 22221211100000
0.00505 [4]: 22222210001000
0.00466 [434]: 22221210020000
0.00414 [50]: 22222110200000
0.00391 [1298]: 22220221010000
0.00377 [2860]: 22212211001000
0.00372 [449]: 22221202100000
0.00371 [8]: 22222202000000
0.00365 [7]: 22222210000001
0.00346 [632]: 22221102110000
0.00345 [21]: 22222200020000
0.00340 [176]: 22222012100000
0.00302 [36]: 22222121000000
0.00282 [201649]: 12222220000001
0.00252 [1407]: 22220202020000

ROOT 6:
0.15795 [2]: 22222210100000
0.12341 [36]: 22222121000000
0.10593 [2850]: 22212221000000
0.06527 [14355]: 22122221000000
0.05179 [4]: 22222210001000
0.03239 [201643]: 12222221000000
0.02302 [201644]: 12222220100000
0.02095 [2860]: 22212211001000
0.02085 [7]: 22222210000001
0.01789 [58279]: 21222220100000
0.01511 [2851]: 22212220100000
0.01389 [58290]: 21222211000010
0.00974 [14356]: 22122220100000
0.00952 [421]: 22221212000000
0.00948 [148]: 22222022000000
0.00932 [39]: 22222120001000
0.00927 [58288]: 21222211001000
0.00834 [77]: 22222101200000
0.00800 [44]: 22222111100000
0.00656 [2853]: 22212220001000
0.00625 [527]: 22221121100000
0.00594 [15]: 22222200200000
0.00469 [11]: 22222201001000
0.00459 [2965]: 22212121001000
0.00437 [201649]: 12222220000001
0.00407 [176]: 22222012100000
0.00394 [1]: 22222211000000
0.00393 [14367]: 22122211000010

0.00391 [155]: 22222020200000
0.00380 [2862]: 22212211000010
0.00329 [415]: 22221220100000
0.00327 [8]: 22222202000000
0.00324 [14366]: 22122211000100
0.00317 [2971]: 22212120101000
0.00304 [534]: 22221120110000
0.00296 [2855]: 22212220000010
0.00277 [422]: 22221211100000
0.00272 [58285]: 21222212000000
0.00261 [5]: 22222210000100

ROOT 7:
0.23481 [58278]: 21222221000000
0.06080 [58288]: 21222211001000
0.05793 [2]: 22222210100000
0.04326 [2850]: 22212221000000
0.04123 [2860]: 22212211001000
0.03859 [58290]: 21222211000010
0.03653 [2862]: 22212211000010
0.02825 [14356]: 22122220100000
0.02172 [6]: 22222210000010
0.02160 [36]: 22222121000000
0.01806 [14365]: 22122211001000
0.01505 [201644]: 12222220100000
0.01111 [7]: 22222210000001
0.01054 [14367]: 22122211000010
0.01032 [58399]: 21222120101000
0.00919 [1]: 22222211000000
0.00876 [2857]: 22212212000000
0.00834 [2971]: 22212120101000
0.00787 [14355]: 22122221000000
0.00773 [58401]: 21222120100010
0.00614 [2973]: 22212120100010
0.00582 [58279]: 21222220100000
0.00523 [58393]: 21222121001000
0.00504 [58285]: 21222212000000
0.00456 [148]: 22222022000000
0.00436 [13]: 22222201000010
0.00424 [2967]: 22212121000010
0.00381 [41]: 22222120000010
0.00376 [201643]: 12222221000000
0.00361 [59174]: 21221220011000
0.00318 [2851]: 22212220100000
0.00317 [14476]: 22122120101000
0.00314 [60726]: 21220221020000
0.00307 [11]: 22222201001000
0.00306 [102222]: 21022221000200
0.00302 [58289]: 21222211000100
0.00283 [44]: 22222111100000
0.00280 [58418]: 21222112100000
0.00277 [3746]: 22211220011000
0.00272 [77]: 22222101200000
0.00259 [14362]: 22122212000000
0.00251 [59176]: 21221220010010

ROOT 8:
0.09526 [201643]: 12222221000000
0.08442 [3]: 22222210010000
0.07908 [7]: 22222210000001
0.06376 [2]: 22222210100000
0.06324 [414]: 22221221000000
0.05543 [14355]: 22122221000000
0.04133 [58278]: 21222221000000
0.02388 [44]: 22222111100000
0.02209 [201649]: 12222220000001
0.02096 [5]: 22222210000100
0.02042 [42]: 22222120000001
0.01779 [9]: 22222201100000
0.01765 [36]: 22222121000000
0.01667 [201644]: 12222220100000
0.01441 [37]: 22222120100000
0.01228 [155]: 22222020200000
0.01171 [8]: 22222202000000
0.00914 [429]: 22221210110000
0.00909 [15]: 22222200200000
0.00903 [16]: 22222200110000

0.00834 [148]: 22222022000000
0.00786 [528]: 22221121010000
0.00722 [4]: 22222210001000
0.00716 [40]: 22222120000100
0.00703 [45]: 22222111010000
0.00594 [43]: 22222112000000
0.00534 [1]: 22222211000000
0.00534 [420]: 22221220000001
0.00516 [2857]: 22212212000000
0.00451 [2860]: 22212211001000
0.00428 [201647]: 12222220000100
0.00413 [201650]: 12222212000000
0.00405 [10]: 22222201010000
0.00404 [58285]: 21222212000000
0.00329 [2862]: 22212211000010
0.00317 [201651]: 12222211100000
0.00285 [14363]: 22122211100000
0.00278 [526]: 22221122000000

0.00267 [2850]: 22212221000000
0.00251 [14359]: 22122220000100

ROOT 9:
0.27629 [14355]: 22122221000000
0.12613 [14366]: 22122211000100
0.06019 [4]: 22222210001000
0.04040 [14477]: 22122120100100
0.03925 [58279]: 21222220100000
0.03697 [58289]: 21222211000100
0.02325 [14356]: 22122220100000
0.02177 [2853]: 22212220001000
0.02145 [201643]: 12222221000000
0.01524 [5]: 22222210000100
0.01223 [37]: 22222120100000
0.01116 [58400]: 21222120100100
0.01048 [1]: 22222211000000
0.00797 [36]: 22222121000000

0.00785 [15252]: 22121220010100
0.00682 [39]: 22222120001000
0.00651 [14364]: 22122211010000
0.00610 [14362]: 22122212000000
0.00600 [58283]: 21222220000010
0.00563 [14372]: 22122210100100
0.00563 [2851]: 22212220100000
0.00498 [2850]: 22212221000000
0.00451 [40]: 22222120000100
0.00427 [12]: 22222201000100
0.00396 [2]: 22222210100000
0.00379 [14575]: 22122102100100
0.00372 [2860]: 22212211001000
0.00361 [14495]: 22122112100000
0.00343 [14360]: 22122220000010
0.00331 [16803]: 22120221020000
0.00326 [84917]: 21112221001010
0.00314 [14363]: 22122211100000

Figure S86: Configurations of states for **1** as obtained at the CASSCF(14,14)/def2-TZVPP level of theory with and without state averaging.

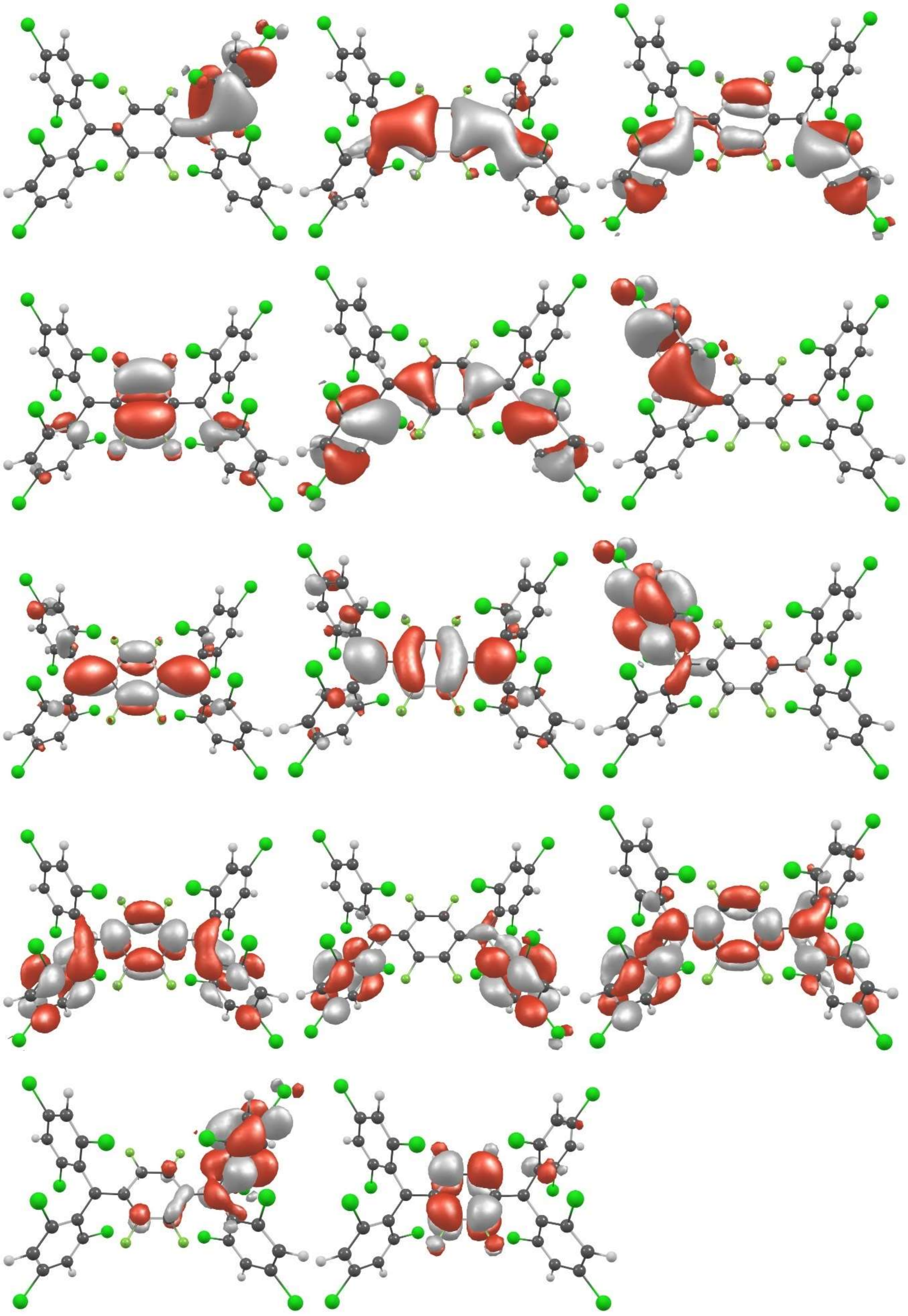

Figure S87: Active space for **2** as obtained at the saCASSCF(14,14)/def2-TZVPP level of theory.

ROOT 0: **No state averaging**
0.78654 [0]: 22222220000000
0.05660 [8]: 22222202000000
0.03295 [44]: 22222111100000
0.01057 [414611]: 02222220000002
0.00549 [155]: 22222020200000
0.00438 [201656]: 12222211000001
0.00354 [201767]: 12222120100001
0.00340 [34226]: 22022220010010
0.00330 [148]: 22222022000000
0.00322 [34223]: 22022220020000
0.00304 [127876]: 20222220000200
0.00269 [15253]: 22121220010010
0.00268 [3750]: 22211220002000
0.00252 [127877]: 20222220000110
0.00252 [59179]: 21221220001100

CAS-SCF STATES FOR BLOCK 0 MULT= 5 NROOTS= 1

ROOT 0:
0.79779 [0]: 22222111100000
0.01173 [364957]: 02222111100020
0.00872 [43690]: 21222120100100
0.00777 [1536]: 22212120100001
0.00755 [43688]: 21222120110000
0.00733 [5509]: 22202111100002
0.00602 [10377]: 22121111111000
0.00460 [23]: 22222101100100
0.00424 [10382]: 22121111101100
0.00418 [188]: 22221120110000
0.00392 [43703]: 21222112100000
0.00348 [21]: 22222101110000
0.00332 [7]: 22222110101000
0.00315 [43782]: 21222102100100
0.00308 [44599]: 21221111110100
0.00295 [13660]: 22112111100002
0.00280 [27080]: 22022111102000
0.00255 [886]: 22220111102000

CAS-SCF STATES FOR BLOCK 1 MULT= 3 NROOTS= 6

ROOT 0:
0.76776 [0]: 22222211000000
0.01775 [167]: 22222011200000
0.01127 [412913]: 02222211000020
0.00929 [28]: 22222121000000
0.00858 [57490]: 21222220000100
0.00773 [2736]: 22212220000001
0.00759 [57488]: 21222220010000
0.00699 [7517]: 22202211000002
0.00571 [14948]: 22121211011000
0.00435 [10]: 22222201000100
0.00428 [57493]: 21222212000000
0.00410 [1]: 22222210100000
0.00403 [14953]: 22121211001100
0.00382 [380]: 22221220010000
0.00380 [3]: 22222210001000
0.00336 [8]: 22222201010000
0.00286 [58410]: 21221211010100
0.00281 [18819]: 22112211000002
0.00273 [57524]: 21222202000100
0.00267 [33918]: 22022211002000

ROOT 1:
0.28818 [29]: 22222120100000
0.17937 [28]: 22222121000000
0.10486 [1]: 22222210100000
0.05594 [57486]: 21222221000000
0.04562 [378]: 22221221000000
0.04119 [2]: 22222210010000
0.03375 [63]: 22222102100000
0.01276 [4]: 22222210000100
0.01237 [35]: 22222112000000
0.00905 [57495]: 21222211010000
0.00786 [36]: 22222111100000
0.00643 [7]: 22222201100000
0.00600 [57635]: 21222111100100
0.00517 [57633]: 21222111110000
0.00410 [413150]: 02222120100020
0.00404 [57497]: 21222211000100
0.00387 [57599]: 21222121100000
0.00352 [2743]: 22212211000001
0.00330 [2881]: 22212111100001
0.00261 [7754]: 22202120100002
0.00260 [389]: 22221211000100
0.00255 [413130]: 02222121000020

ROOT 2:
0.20288 [378]: 22221221000000
0.13808 [2]: 22222210010000
0.13743 [29]: 22222120100000
0.10773 [57486]: 21222221000000
0.02804 [381]: 22221220001000
0.02157 [14027]: 22122220010000
0.02125 [4]: 22222210000100
0.01928 [57495]: 21222211010000
0.01714 [28]: 22222121000000
0.01571 [14029]: 22122220000100
0.01502 [63]: 22222102100000
0.01069 [57489]: 21222220001000
0.00934 [1]: 22222210100000
0.00913 [389]: 22221211000100
0.00621 [380]: 22221220010000
0.00602 [2743]: 22212211000001
0.00486 [379]: 22221220100000
0.00478 [57497]: 21222211000100
0.00468 [762]: 22221021200000
0.00455 [2732]: 22212220010000
0.00445 [35]: 22222112000000
0.00416 [199932]: 12222220000010
0.00410 [415]: 22221202001000
0.00393 [7]: 22222201100000
0.00329 [2734]: 22212220000100
0.00323 [382]: 22221220000100
0.00321 [188]: 22222010210000
0.00313 [14061]: 22122202010000
0.00299 [30]: 22222120010000
0.00290 [414362]: 02221221000020
0.00286 [14032]: 22122212000000

ROOT 3:
0.15658 [380]: 22221220010000
0.13018 [14028]: 22122220001000
0.11473 [14025]: 22122221000000
0.07435 [382]: 22221220000100
0.06898 [2730]: 22212221000000
0.03706 [3]: 22222210001000
0.02673 [2733]: 22212220001000
0.02077 [57488]: 21222220010000
0.01674 [57486]: 21222221000000
0.01570 [14062]: 22122202001000
0.01466 [414]: 22221202010000
0.01398 [4]: 22222210000100
0.01371 [29]: 22222120100000
0.01194 [14027]: 22122220010000
0.01152 [385]: 22221212000000
0.00864 [14026]: 22122220100000
0.00639 [381]: 22221220001000
0.00620 [58410]: 21221211010100
0.00616 [57490]: 21222220000100
0.00591 [416]: 22221202000100
0.00485 [379]: 22221220100000
0.00433 [14029]: 22122220000100
0.00410 [58370]: 21221221010000
0.00365 [2767]: 22212202001000
0.00362 [57493]: 21222212000000
0.00347 [388]: 22221211001000
0.00336 [783]: 22221020210000
0.00307 [10]: 22222201000100
0.00287 [8]: 22222201010000
0.00287 [14431]: 22122020201000
0.00276 [14409]: 22122021200000
0.00272 [14034]: 22122211010000
0.00271 [378]: 22221221000000
0.00265 [2731]: 22212220100000
0.00253 [77387]: 21122211001100

ROOT 4:
0.50493 [2730]: 22212221000000
0.10214 [14025]: 22122221000000
0.04087 [6]: 22222210000001
0.03349 [2739]: 22212211010000
0.02418 [57486]: 21222221000000
0.02257 [2741]: 22212211000100
0.02062 [381]: 22221220001000
0.01427 [14027]: 22122220010000
0.01121 [4]: 22222210000100
0.01100 [3114]: 22212021200000
0.00733 [57499]: 21222211000001
0.00725 [14034]: 22122211010000
0.00705 [419447]: 02212221000020
0.00530 [14029]: 22122220000100
0.00481 [14036]: 22122211000100
0.00468 [20332]: 22111221011000
0.00396 [2732]: 22212220010000
0.00367 [62226]: 21212222000000
0.00300 [20337]: 22111221001100
0.00288 [378]: 22221221000000
0.00265 [391]: 22221211000001
0.00265 [199932]: 12222220000010

ROOT 5:
0.14960 [57486]: 21222221000000
0.13309 [381]: 22221220001000
0.08814 [14027]: 22122220010000
0.05067 [4]: 22222210000100
0.04731 [14025]: 22122221000000
0.04642 [2730]: 22212221000000
0.04463 [14028]: 22122220001000
0.03429 [14029]: 22122220000100
0.02072 [57489]: 21222220001000
0.01995 [378]: 22221221000000
0.01873 [2732]: 22212220010000
0.01356 [2733]: 22212220001000
0.01275 [415]: 22221202001000
0.01214 [29]: 22222120100000
0.01048 [3]: 22222210001000
0.00778 [14061]: 22122202010000
0.00746 [14032]: 22122212000000
0.00688 [199932]: 12222220000010
0.00684 [2734]: 22212220000100
0.00611 [2739]: 22212211010000
0.00573 [57488]: 21222220010000
0.00571 [6]: 22222210000001
0.00544 [14062]: 22122202001000
0.00494 [387]: 22221211010000
0.00465 [9]: 22222201001000
0.00434 [57497]: 21222211000100
0.00351 [379]: 22221220100000
0.00350 [14063]: 22122202000100
0.00342 [57870]: 21222021200000
0.00340 [57523]: 21222202001000
0.00340 [380]: 22221220010000
0.00307 [77383]: 21122211010100
0.00290 [784]: 22221020201000
0.00279 [58414]: 21221211001100
0.00260 [2741]: 22212211000100
0.00256 [58409]: 21221211011000
0.00251 [57487]: 21222220100000

CAS-SCF STATES FOR BLOCK 2 MULT= 1 NROOTS=10

ROOT 0: E= -7128.8551863415 Eh
0.73483 [0]: 22222220000000
0.06199 [8]: 22222202000000
0.01549 [155]: 22222020200000
0.01154 [58289]: 21222211000100
0.01044 [414609]: 02222220000020
0.00999 [58287]: 21222211010000
0.00659 [7617]: 22202220000002
0.00623 [2863]: 22212211000001
0.00616 [58278]: 21222221000000
0.00556 [15251]: 22121220011000
0.00463 [423]: 22221211010000
0.00364 [15256]: 22121220001100
0.00354 [414]: 22221221000000
0.00321 [43]: 22222112000000
0.00267 [19122]: 22112220000002
0.00258 [44]: 22222111100000

ROOT 1:
0.34622 [8]: 22222202000000
0.13700 [58278]: 21222221000000
0.09654 [3]: 22222210010000
0.08021 [414]: 22221221000000
0.03914 [5]: 22222210000100
0.02336 [36]: 22222121000000
0.01893 [2]: 22222210100000
0.01811 [44]: 22222111100000
0.01151 [127854]: 20222222000000
0.00841 [58287]: 21222211010000
0.00813 [253]: 22222002200000
0.00715 [23]: 22222200010100
0.00711 [0]: 22222220000000
0.00564 [59160]: 21221222000000
0.00517 [425]: 22221211000100
0.00501 [414707]: 02222202000020
0.00445 [21]: 22222200020000
0.00427 [14365]: 22122211001000
0.00311 [58662]: 21222021200000
0.00304 [30]: 22222200000200
0.00300 [7715]: 22202202000002
0.00299 [201643]: 12222221000000
0.00298 [58289]: 21222211000100
0.00290 [201655]: 12222211000010
0.00269 [15349]: 22121202011000
0.00256 [14355]: 22122221000000

ROOT 2:
0.53631 [1]: 22222211000000
0.08303 [14355]: 22122221000000
0.04763 [36]: 22222121000000
0.03039 [43]: 22222112000000
0.02850 [44]: 22222111100000
0.01797 [58285]: 21222212000000
0.01513 [10]: 22222201010000
0.01382 [421]: 22221212000000
0.01377 [201643]: 12222221000000
0.01241 [2850]: 22212221000000
0.01023 [12]: 22222201000100
0.00899 [182]: 22222011200000
0.00727 [416]: 22221220010000
0.00669 [414636]: 02222211000020
0.00548 [37]: 22222120100000
0.00426 [2859]: 22212211010000
0.00410 [7644]: 22202211000002
0.00330 [15278]: 22121211011000
0.00307 [6]: 22222210000010
0.00290 [14358]: 22122220001000
0.00273 [4]: 22222210001000
0.00268 [9]: 22222201100000
0.00261 [201648]: 12222220000010

ROOT 3:
0.60656 [2850]: 22212221000000
0.05216 [1]: 22222211000000
0.04921 [14355]: 22122221000000
0.04106 [2859]: 22212211010000
0.03269 [2861]: 22212211000100
0.01477 [63018]: 21212222000000
0.01310 [3234]: 22212021200000
0.01249 [14364]: 22122211010000
0.00853 [421373]: 02212221000020
0.00842 [7]: 22222210000001
0.00513 [20662]: 22111221011000
0.00390 [421]: 22221212000000
0.00340 [58291]: 21222211000001
0.00334 [2899]: 22212201010100
0.00332 [20667]: 22111221001100
0.00325 [2858]: 22212211100000
0.00319 [14366]: 22122211000100
0.00256 [3732]: 22211222000000
0.00253 [201643]: 12222221000000
0.00252 [40992]: 22012221002000

ROOT 4:
0.23660 [44]: 22222111100000
0.12076 [36]: 22222121000000
0.11283 [43]: 22222112000000
0.08471 [9]: 22222201100000
0.06958 [2]: 22222210100000
0.06244 [1]: 22222211000000
0.02334 [14355]: 22122221000000
0.02154 [414]: 22221221000000
0.01238 [3]: 22222210010000
0.01043 [149]: 22222021100000
0.00824 [421]: 22221212000000
0.00630 [58278]: 21222221000000
0.00560 [2850]: 22212221000000
0.00357 [201643]: 12222221000000
0.00357 [58400]: 21222120100100
0.00354 [50]: 22222110200000
0.00345 [414965]: 02222111100020
0.00324 [58398]: 21222120110000
0.00318 [2974]: 22212120100001
0.00315 [58285]: 21222212000000
0.00312 [148]: 22222022000000
0.00310 [425]: 22221211000100
0.00274 [2859]: 22212211010000
0.00258 [80]: 22222101100100
0.00254 [14366]: 22122211000100
0.00250 [423]: 22221211010000

ROOT 5:
0.11068 [414]: 22221221000000
0.07629 [14355]: 22122221000000
0.06489 [423]: 22221211010000
0.05322 [421]: 22221212000000
0.04756 [14362]: 22122212000000
0.04703 [424]: 22221211001000
0.04209 [58278]: 21222221000000
0.03921 [425]: 22221211000100
0.03858 [14364]: 22122211010000
0.02864 [5]: 22222210000100
0.02736 [14365]: 22122211001000
0.02471 [14366]: 22122211000100
0.02005 [4]: 22222210001000
0.01966 [10]: 22222201010000
0.01692 [1]: 22222211000000
0.01400 [44]: 22222111100000
0.01340 [58285]: 21222212000000
0.01306 [11]: 22222201001000
0.01018 [2857]: 22212212000000
0.00957 [2859]: 22212211010000
0.00832 [2850]: 22212221000000
0.00805 [43]: 22222112000000
0.00752 [422]: 22221211100000
0.00723 [12]: 22222201000100
0.00597 [14363]: 22122211100000
0.00573 [2860]: 22212211001000
0.00489 [58287]: 21222211010000
0.00408 [3]: 22222210010000
0.00406 [2861]: 22212211000100
0.00401 [58289]: 21222211000100
0.00374 [58288]: 21222211001000
0.00309 [201643]: 12222221000000
0.00251 [36]: 22222121000000

ROOT 6:
0.10638 [14355]: 22122221000000
0.07594 [414]: 22221221000000
0.07179 [14365]: 22122211001000
0.06895 [424]: 22221211001000
0.06015 [58278]: 21222221000000
0.04547 [14362]: 22122212000000
0.03588 [14364]: 22122211010000
0.03419 [4]: 22222210001000
0.03402 [421]: 22221212000000
0.02492 [58285]: 21222212000000
0.02453 [14366]: 22122211000100
0.02296 [58288]: 21222211001000
0.02252 [423]: 22221211010000
0.02123 [11]: 22222201001000
0.01567 [2860]: 22212211001000
0.01497 [425]: 22221211000100
0.01359 [2850]: 22212221000000
0.01326 [5]: 22222210000100
0.01240 [1]: 22222211000000
0.01112 [10]: 22222201010000
0.01010 [2857]: 22212212000000
0.00947 [2859]: 22212211010000
0.00938 [58287]: 21222211010000
0.00605 [58289]: 21222211000100
0.00509 [12]: 22222201000100
0.00407 [2861]: 22212211000100
0.00369 [201643]: 12222221000000
0.00305 [36]: 22222121000000

ROOT 7:
0.31934 [201643]: 12222221000000
0.20214 [201655]: 12222211000010
0.09999 [201650]: 12222212000000
0.03223 [6]: 22222210000010
0.01879 [13]: 22222201000010
0.01797 [201654]: 12222211000100
0.01469 [414]: 22221221000000
0.01057 [1]: 22222211000000
0.01046 [58290]: 21222211000010
0.01020 [201656]: 12222211000001
0.00689 [201653]: 12222211001000
0.00686 [202027]: 12222021200000
0.00684 [414589]: 02222221000010
0.00671 [36]: 22222121000000
0.00592 [8]: 22222202000000
0.00559 [58278]: 21222221000000
0.00541 [426]: 22221211000010
0.00526 [201652]: 12222211010000
0.00470 [202122]: 12222011200010
0.00361 [201755]: 12222122000000
0.00303 [421]: 22221212000000
0.00287 [201651]: 12222211100000
0.00280 [201760]: 12222121000010
0.00279 [3]: 22222210010000
0.00264 [202098]: 12222012200000
0.00263 [213174]: 12202221000002

ROOT 8:
0.37869 [36]: 22222121000000
0.14416 [2]: 22222210100000
0.07733 [14355]: 22122221000000
0.03768 [44]: 22222111100000
0.03280 [9]: 22222201100000
0.02738 [4]: 22222210001000
0.01051 [14364]: 22122211010000
0.01012 [424]: 22221211001000
0.00873 [416]: 22221220010000
0.00871 [5]: 22222210000100
0.00848 [58278]: 21222221000000
0.00768 [2850]: 22212221000000
0.00749 [10]: 22222201010000
0.00715 [414]: 22221221000000
0.00514 [414874]: 02222121000020
0.00449 [201655]: 12222211000010

```
0.00425 [     8]: 22222202000000
0.00422 [    45]: 22222111010000
0.00392 [    37]: 22222120100000
0.00392 [     3]: 22222210010000
0.00346 [ 58288]: 21222211001000
0.00331 [   176]: 22222012100000
0.00308 [  7882]: 22202121000002
0.00290 [     0]: 22222220000000
0.00283 [    71]: 22222102100000
0.00267 [    43]: 22222112000000
0.00262 [201650]: 12222212000000
0.00250 [    38]: 22222120010000

ROOT   9:
0.42551 [   414]: 22221221000000
0.07525 [   423]: 22221211010000
0.06747 [     3]: 22222210010000
0.05042 [ 14365]: 22122211001000
0.02693 [     8]: 22222202000000
0.02366 [    11]: 22222201001000
0.01468 [201655]: 12222211000010
0.01413 [    36]: 22222121000000
0.01343 [     0]: 22222220000000
0.01024 [     5]: 22222210000100
0.01018 [ 58289]: 21222211000100
0.01004 [  2860]: 22212211001000
0.00963 [ 14357]: 22122220010000
0.00873 [   798]: 22221021200000
0.00799 [201650]: 12222212000000
0.00709 [ 14362]: 22122212000000
0.00692 [   425]: 22221211000100
0.00643 [    44]: 22222111100000
0.00585 [416162]: 02221221000020
0.00584 [     2]: 22222210100000
0.00453 [  1296]: 22220222000000
0.00407 [   461]: 22221201020000
0.00392 [  2863]: 22212211000001
0.00356 [  9170]: 22201221000002
0.00354 [   417]: 22221220001000
0.00295 [ 58287]: 21222211010000
```

Figure S88: Configurations of states for **2** as obtained at the CASSCF(14,14)/def2-TZVPP level of theory with and without state averaging.

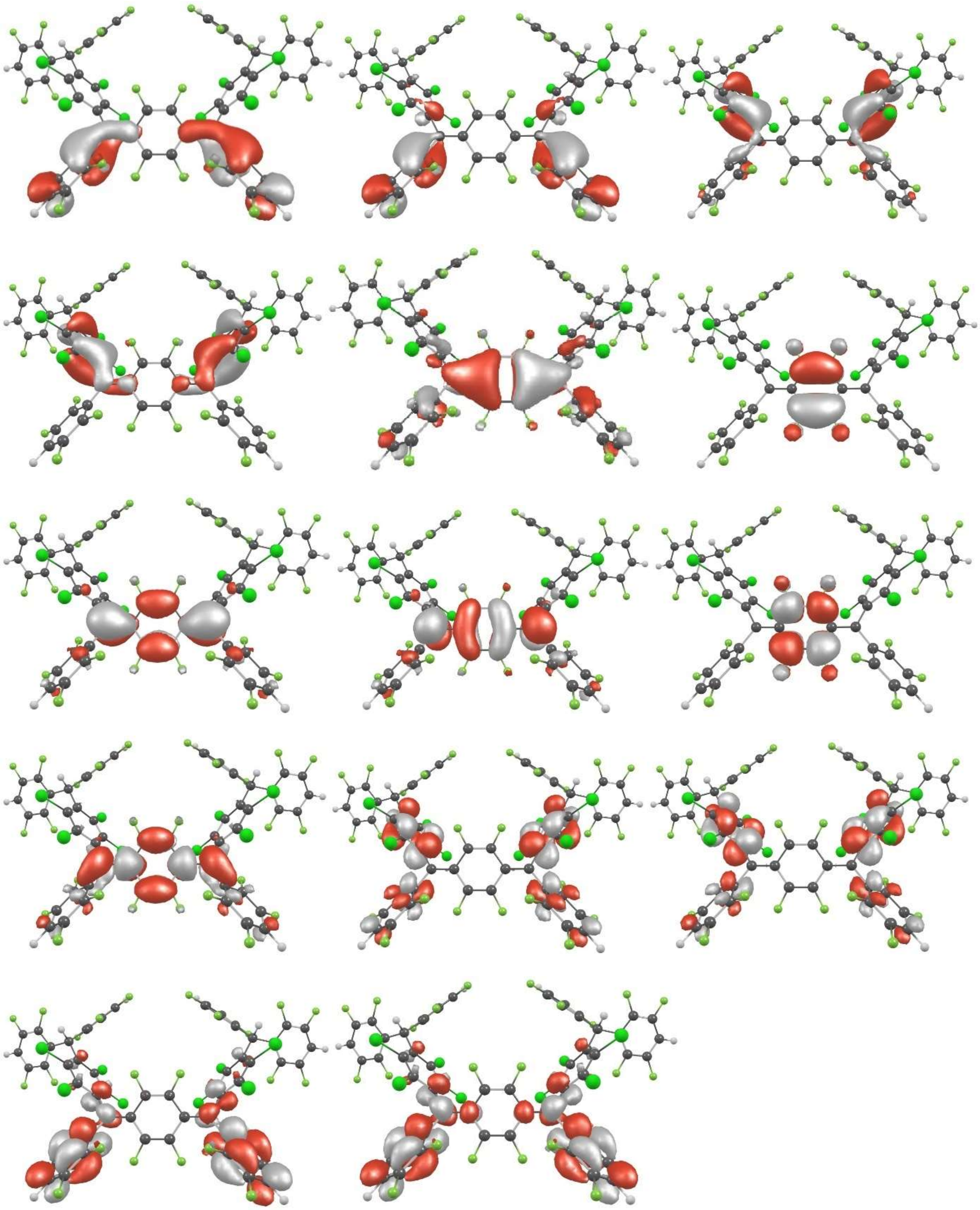

Figure S89: Activate space for **3** as obtained at the saCASSCF(14,14)/def2-TZVPP level of theory.

ROOT 0: **No state averaging**
0.79825 [0]: 22222220000000
0.05129 [8]: 22222202000000
0.03262 [44]: 22222111100000
0.01376 [3746]: 22211220011000
0.01146 [78156]: 21122220000110
0.01066 [414611]: 02222220000002
0.00528 [155]: 22222020200000
0.00400 [201656]: 12222211000001
0.00365 [148]: 22222022000000
0.00352 [1314]: 22220220002000
0.00346 [7608]: 22202220002000
0.00345 [7603]: 22202220020000
0.00334 [1309]: 22220220020000
0.00325 [201767]: 12222120100001
0.00299 [34232]: 22022220000200
0.00294 [127876]: 20222220000200
0.00281 [34235]: 22022220000020
0.00273 [127879]: 20222220000020
0.00270 [15]: 22222200200000

CAS-SCF STATES FOR BLOCK 0 MULT= 5 NROOTS= 1

ROOT 0: E= -8665.0396831182 Eh
0.74670 [0]: 22222111100000
0.06995 [127]: 22221211010000
0.02266 [188]: 22221120110000
0.00862 [13652]: 22112111101100
0.00637 [280]: 22221102110000
0.00562 [228277]: 11222111100011
0.00405 [470]: 22221011210000
0.00367 [881]: 22220111120000
0.00343 [1472]: 22212211001000
0.00311 [163896]: 12222211000001
0.00251 [1471]: 22212211010000

CAS-SCF STATES FOR BLOCK 1 MULT= 3 NROOTS= 3

ROOT 0: E= -8665.2307093185 Eh
0.79155 [0]: 22222211000000
0.01802 [29]: 22222120100000
0.01536 [380]: 22221220010000
0.01230 [167]: 22222011200000
0.00913 [18811]: 22112211001100
0.00777 [8]: 22222201010000
0.00594 [269556]: 11222211000011
0.00573 [525]: 22221111110000
0.00491 [414]: 22221202010000
0.00384 [491]: 22221121100000
0.00383 [385]: 22221212000000
0.00330 [43]: 22222110110000
0.00322 [1293]: 22220211020000
0.00252 [33922]: 22022211000200
0.00252 [7512]: 22202211000200

ROOT 1:
0.69911 [28]: 22222121000000
0.08091 [1]: 22222210100000
0.06690 [37]: 22222111010000
0.01457 [386]: 22221211100000
0.00786 [19049]: 22112121001100
0.00533 [41]: 22222111000001
0.00490 [269794]: 11222121000011
0.00467 [75]: 22222101020000
0.00323 [490]: 22221122000000
0.00274 [161]: 22222012100000

ROOT 2:
0.44700 [378]: 22221221000000
0.24976 [2]: 22222210010000
0.03397 [387]: 22221211010000
0.01804 [36]: 22222111100000
0.01791 [199932]: 12222220000010
0.01205 [57492]: 21222220000001
0.01093 [57489]: 21222220001000
0.00717 [2730]: 22212221000000
0.00709 [762]: 22221021200000
0.00605 [14031]: 22122220000001
0.00590 [199931]: 12222220000100
0.00584 [2734]: 22212220000100
0.00566 [199936]: 12222211010000
0.00537 [20337]: 22111221001100
0.00437 [391]: 22221211000001
0.00420 [188]: 22222010210000
0.00404 [2739]: 22212211010000
0.00403 [425]: 22221201020000
0.00372 [383]: 22221220000010
0.00364 [57488]: 21222220010000
0.00321 [271082]: 11221221000011
0.00308 [498]: 22221120110000
0.00286 [18845]: 22112210011100

CAS-SCF STATES FOR BLOCK 2 MULT= 1 NROOTS=10

ROOT 0: E= -8665.2906243399 Eh
0.75496 [0]: 22222220000000
0.06409 [8]: 22222202000000
0.01880 [423]: 22221211010000
0.01207 [44]: 22222111100000
0.01133 [155]: 22222020200000
0.00852 [19114]: 22112220001100
0.00737 [414]: 22221221000000
0.00538 [271245]: 11222220000011
0.00409 [534]: 22221120110000
0.00286 [2859]: 22212211010000
0.00251 [201652]: 12222211010000

ROOT 1:
0.37499 [8]: 22222202000000
0.22624 [414]: 22221221000000
0.15933 [3]: 22222210010000
0.01310 [21]: 22222200020000
0.01153 [2850]: 22212221000000
0.01121 [0]: 22222220000000
0.01099 [1296]: 22220222000000
0.00882 [423]: 22221211010000
0.00588 [148]: 22222022000000
0.00551 [201656]: 12222211000001
0.00540 [58290]: 21222211000010
0.00522 [253]: 22222002200000
0.00426 [19212]: 22112202001100
0.00413 [201643]: 12222221000000
0.00350 [3732]: 22211222000000
0.00330 [25]: 22222200010001
0.00321 [177]: 22222012010000
0.00298 [202525]: 12221222000000
0.00292 [534]: 22221120110000
0.00288 [20667]: 22111221001100
0.00279 [271343]: 11222202000011

ROOT 2:
0.09029 [45]: 22222111010000
0.02212 [526]: 22221122000000
0.01542 [2]: 22222210100000
0.00863 [83]: 22222101020000
0.00815 [19379]: 22112121001100
0.00761 [422]: 22221211100000
0.00693 [49]: 22222111000001
0.00509 [271510]: 11222121000011
0.00406 [2962]: 22212122000000
0.00331 [201755]: 12222122000000
0.00259 [533]: 22221120200000

ROOT 3:
0.67064 [1]: 22222211000000
0.05509 [14355]: 22122221000000
0.04802 [421]: 22221212000000
0.02694 [10]: 22222201010000
0.02548 [58278]: 21222221000000
0.01432 [6]: 22222210000010
0.00802 [182]: 22222011200000
0.00675 [19141]: 22112211001100
0.00409 [271272]: 11222211000011
0.00392 [2853]: 22212220001000
0.00372 [416]: 22221220010000
0.00368 [37]: 22222120100000
0.00341 [58283]: 21222220000010
0.00330 [58282]: 21222220000100
0.00320 [420]: 22221220000001
0.00312 [14359]: 22122220000100
0.00292 [201645]: 12222220010000
0.00261 [14360]: 22122220000010
0.00255 [2857]: 22212212000000
0.00251 [51]: 22222110110000

ROOT 4:
0.24661 [58278]: 21222221000000
0.11916 [201650]: 12222212000000
0.08297 [201655]: 12222211000010
0.07424 [58291]: 21222211000001
0.04601 [6]: 22222210000010
0.03233 [58287]: 21222211010000
0.02631 [5]: 22222210000100
0.02524 [58288]: 21222211001000
0.02272 [201654]: 12222211000100
0.02134 [14]: 22222201000001
0.02111 [426]: 22221211000010
0.01898 [11]: 22222201001000
0.01654 [14365]: 22122211001000
0.01403 [421]: 22221212000000
0.01161 [1]: 22222211000000
0.01116 [425]: 22221211000100
0.01076 [10]: 22222201010000
0.00454 [2861]: 22212211000100
0.00373 [14368]: 22122211000001
0.00350 [58662]: 21222021200000
0.00327 [58398]: 21222120110000
0.00310 [84916]: 21112221001100
0.00286 [271224]: 11222221000010

ROOT 5:
0.28124 [201643]: 12222221000000
0.11810 [58285]: 21222212000000
0.07903 [58290]: 21222211000010
0.05959 [201656]: 12222211000001
0.03590 [4]: 22222210001000
0.02945 [7]: 22222210000001
0.02841 [201652]: 12222211010000
0.02427 [201653]: 12222211001000
0.02413 [13]: 22222201000010
0.02298 [14366]: 22122211000100
0.01720 [12]: 22222201000100
0.01499 [424]: 22221211001000
0.01394 [427]: 22221211000001
0.01378 [58289]: 21222211000100
0.01210 [414]: 22221221000000
0.01157 [8]: 22222202000000
0.00961 [2860]: 22212211001000
0.00449 [2850]: 22212221000000
0.00428 [423]: 22221211010000
0.00388 [202027]: 12222021200000
0.00337 [228281]: 12112221001100
0.00328 [201763]: 12222120110000
0.00294 [14367]: 22122211000010
0.00283 [14362]: 22122212000000
0.00276 [202798]: 12221121110000

ROOT 6:
0.31184 [2850]: 22212221000000
0.10178 [2860]: 22212211001000
0.09987 [14366]: 22122211000100
0.09190 [14362]: 22122212000000
0.05403 [414]: 22221221000000
0.02005 [2863]: 22212211000001
0.01989 [58289]: 21222211000100
0.01884 [14367]: 22122211000010

0.01609 [4]: 22222210001000
0.01601 [2859]: 22212211010000
0.01215 [424]: 22221211001000
0.01114 [7]: 22222210000001
0.00999 [8]: 22222202000000
0.00963 [12]: 22222201000100
0.00806 [427]: 22221211000001
0.00564 [13]: 22222201000010
0.00561 [201653]: 12222211001000
0.00524 [201643]: 12222221000000
0.00424 [3234]: 22212021200000
0.00281 [4005]: 22211121110000
0.00280 [2970]: 22212120110000
0.00279 [423]: 22221211010000

ROOT 7:
0.26276 [14355]: 22122221000000
0.12680 [2861]: 22212211000100
0.11311 [14365]: 22122211001000
0.09372 [2857]: 22212212000000
0.02930 [14368]: 22122211000001
0.02421 [58288]: 21222211001000
0.02233 [5]: 22222210000100
0.02008 [14364]: 22122211010000
0.01809 [2862]: 22212211000010
0.01568 [425]: 22221211000100
0.01489 [6]: 22222210000010
0.01306 [421]: 22221212000000
0.01302 [1]: 22222211000000
0.01286 [58278]: 21222221000000
0.01036 [11]: 22222201001000
0.00793 [426]: 22221211000010
0.00677 [14]: 22222201000001
0.00624 [201654]: 12222211000100
0.00587 [10]: 22222201010000
0.00367 [14739]: 22122021200000
0.00285 [14475]: 22122120110000

ROOT 8:
0.68840 [2]: 22222210100000
0.08868 [422]: 22221211100000
0.02843 [36]: 22222121000000
0.02352 [16]: 22222200110000
0.01196 [2858]: 22212211100000
0.01104 [201651]: 12222211100000
0.00770 [19161]: 22112210101100
0.00636 [1324]: 22220212100000
0.00594 [156]: 22222020110000
0.00493 [271292]: 11222210100011
0.00430 [20]: 22222200100001
0.00307 [45]: 22222111010000

ROOT 9:
0.25329 [414]: 22221221000000
0.15368 [2850]: 22212221000000
0.07753 [2860]: 22212211001000
0.07222 [14366]: 22122211000100
0.02775 [4]: 22222210001000
0.02767 [3]: 22222210010000
0.02557 [423]: 22221211010000
0.02247 [58289]: 21222211000100
0.02149 [14367]: 22122211000010
0.01625 [8]: 22222202000000
0.01525 [2859]: 22212211010000
0.01421 [13]: 22222201000010
0.01409 [7]: 22222210000001
0.01275 [14362]: 22122212000000
0.00950 [14357]: 22122220010000
0.00938 [12]: 22222201000100
0.00901 [0]: 22222220000000
0.00802 [201653]: 12222211001000
0.00682 [44]: 22222111100000
0.00648 [58285]: 21222212000000
0.00642 [2863]: 22212211000001
0.00531 [424]: 22221211001000
0.00479 [461]: 22221201020000
0.00444 [201656]: 12222211000001
0.00429 [58290]: 21222211000010
0.00361 [798]: 22221021200000
0.00303 [427]: 22221211000001

Figure S90: Configurations of states for **3** as obtained at the CASSCF(14,14)/def2-TZVPP level of theory without state averaging.

Table S12: Energies and order of states for **1**^{flat} as obtained by various methods. The NEVPT2 and CASPT2K calculations indicate vast dynamical correlation, hence affording unreliable energies (as well as intruder states).

Method	***State***	***Energy* [eV]**	***Character***
**CI-sr-ctPBE0**	S0	0	Open-shell singlet
	S1	2.38	Doubly Excited
	S2	2.60	H→L
	S3	3.26	H-1→L (CT character)
	S4	3.34	H-3→L (CT character)
	S5	3.39	H-2→L (CT character)
	T1	0.15	H→L
	T2	3.24	H-2→L (CT character)
	T3	3.30	H-3→L (CT character)
	T4	3.31	H-1→L (CT character)
**CI-sr-ctPBE**	Not converged		
**sr-ctPBE0**	Not converged		
**sr-ctPBE**	Not converged		
**CI-ctPBE**	Not converged		
**CI-ctPBE0**	S0	0	Open-shell singlet
	S1	0.85	H→L
	S2	1.25	Doubly Excited
	S3	3.21	H-1→L (CT character)
	S4	3.52	H→L+1 (CT character)
	T1	0.40	H→L
	T2	3.12	H-1→L (CT character)
	T3	3.41	H→L+1 (CT character)

**NEVPT2**	S0	0	Open-shell singlet
	S1	1.47	H→L (small CT character)
	S2	1.96	Doubly Excited (considerable tetraradical & small CT character)
	S3	2.64	H-1→L (CT, considerable tetraradical)
	S4	3.09	very mixed
	T1	0.13	H→L
	T2	2.852	very mixed
	T3	3.06	very mixed
	T4	3.07	very mixed
	Q1	3.97	H-1→L+1
**CASPT2K**	S0	0	Open-shell singlet
	S1	3.98	H-1→L
	S2	5.08	H-3→L
	T1	0.16	H→L

Table S13: Energies and order of states for **1** as obtained by various methods. The NEVPT2 and CASPT2K calculations indicate vast dynamical correlation, hence affording potentially unreliable energies (as well as intruder states).

Method	***State***	***Energy* [eV]**	***Character***
**CI-sr-ctPBE0**	Not converged		
**CI-sr-ctPBE**	Not converged		
**sr-ctPBE0**	Not converged		
**sr-ctPBE**	Not converged		
**CI-ctPBE**	converged		
**CI-ctPBE**	S0	0	Closed-shell singlet
	S1	3.03	H→L
	S2	3.52	H-1→L (CT character)
	S3	3.82	H-3→L
	S4	4.09	H-4→L
	T1	2.46	H→L
	T2	3.12	H-1→L (CT character)
	T3	3.56	H→L+2
	T4	3.85	H-6→L (CT character)
**CI-ctPBE0**	S0		Closed-shell singlet
	S1	3.76	H→L
	S2	4.48	H-1→L (CT character)
	S3	4.98	H-3→L
	S4	5.36	H-5→L (CT character)
	T1	3.32	H→L
	T2	4.18	H-1→L (CT character)
	T3	4.82	H-2→L+1 (CT character)
**NEVPT2**	S0	0	Closed-shell singlet
	S1	4.10	H-2→L (CT character)
	S2	4.91	H→L+1 (CT character)
	T1	3.03	H→L
	T2	3.86	H→L+1 (CT character)

	T3	4.66	H-2→L (CT character)
**CASPT2K**	Not converged		

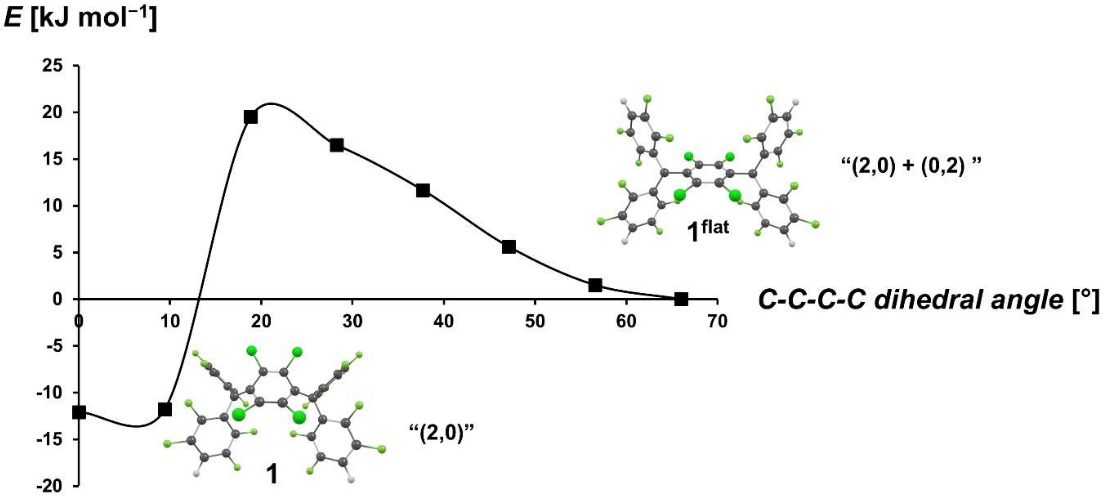

Figure S91: Potential energy scan at the CASSCF(6,6)/def2-SVP level of theory for the conformational flip of **1** to $\mathbf{1}^{\mathbf{flat}}$.

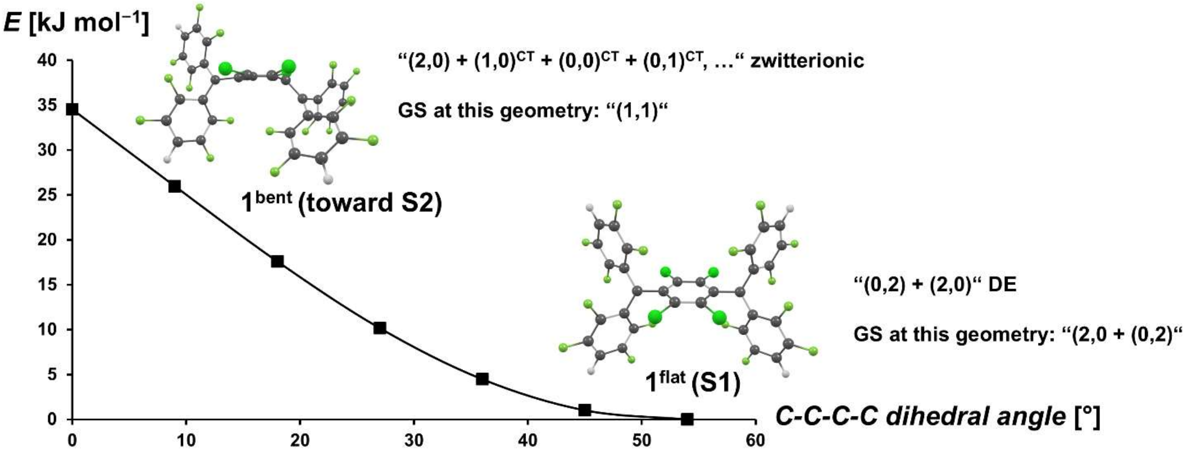

Figure S92: Potential energy scan at the CASSCF(6,6)/def2-SVP level of theory for the conformational of the S1 of $\mathbf{1}^{\mathbf{flat}}$ excited state leading to zwitterionic character (toward S2).

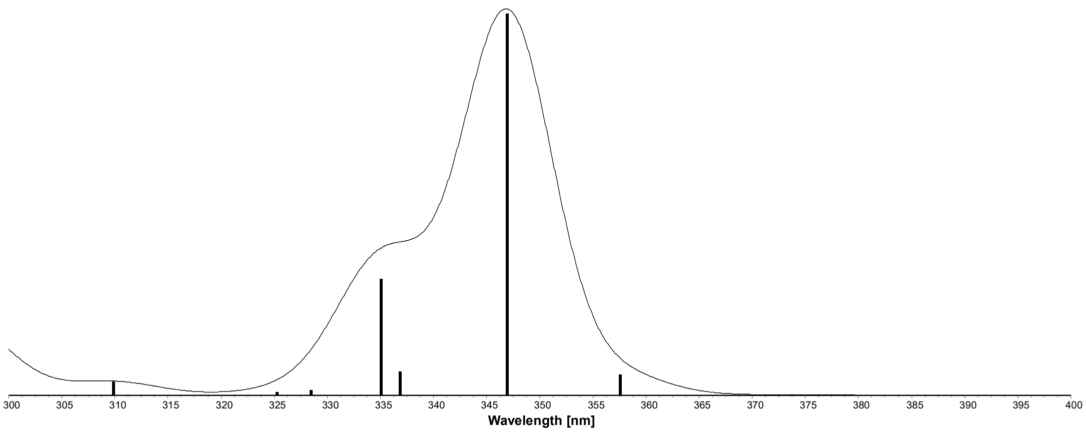

Figure S93: UV-Vis electronic absorption spectrum of **1** as obtained by TDDFT (PBE0/def2-TZVPP, gas-phase).

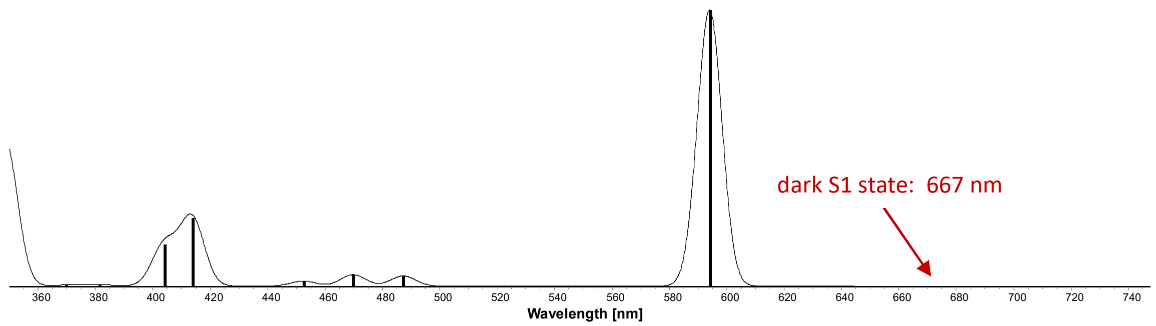

Figure S94: UV-Vis electronic absorption spectrum of $\mathbf{1}^{flat}$ as obtained by TDDFT (PBE0/def2-TZVPP, gas-phase).

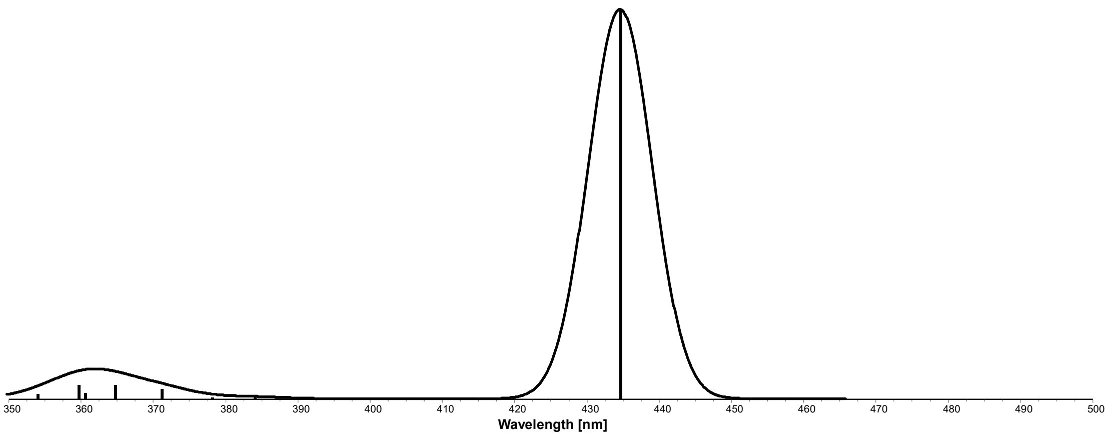

Figure S95: UV-Vis electronic absorption spectrum of **2** as obtained by TDDFT (PBE0/def2-TZVPP, gas-phase).

Table S14: Computed dipole moments in the gas-phase as obtained at various levels of theory.

Theory Level	1	1^{flat}	2
TDDFT: PBE0/def2-TZVPP	1.594 Debye	0.001 Debye	0.189 Debye
PBE0/def2-TZVPP//PBE0-D4/def2-SVP	1.795 Debye	0.006 Debye	0.005 Debye
DLPNO-CCSD(T1)/cc-pvtz//PBE0-D4/def2-SVP	1.971 Debye	0.001 Debye	0.001 Debye
ωB97X-V/def2-TZVPP//ωB97X-V/def2-SVP	1.910 Debye	0.0001 Debye	0.018 Debye
TPSSh/def2-TZVPP//TPSSh-D4/def2-SVP	1.820 Debye	0.001 Debye	0.016 Debye
CASSCF(14,14)/def2-TZVPP	1.752 Debye	0.001 Debye	0.188 Debye

Table S15: To approximate the Onsager-Radius, the vdW cavity obtained by the Gaussian charge scheme used by default in ORCA's CPCM module was used and converted to an ideal sphere to obtain the corresponding idealized radius.

	1	1^{flat}	2	[unit]
Cavity Volume	4790	4777	5338	$Å^3$
Cavity Surface Area	2211	2230	2431	$Å^2$
Sphere Radius	10.457	10.448	10.842	Å

Table S16: Computed energies for transition to "S1" (SE) excited states for compounds **1**–**3** according to TDDFT (PBE0/def2-TZVPP) in various solvents (gasphase, CPCM=pentane, CPCM=DCM).

compound	gasphase	pentane	DCM
1	357 nm	358 nm	358 nm
1^{flat}	667 nm (dark), 594 nm (bright)	667 nm (dark), 604 nm (bright)	667 nm (dark), 606 nm (bright)
2	435 nm	457 nm	460 nm
3	404 nm	422 nm	424 nm

Table S17: Computed energies of compounds **1**–**3** and reference compounds.

**Compound**	**ωB97XV/def2-SVP**					**ωB97X-V/def2-TZVPP//wB97X-V/def2-SVP**			
	**$\langle S^2 \rangle$**	***E***	**Δ*E***	**G**	**Δ*G***	***E*(SP)**	**Δ*E*(SP)**	**G(SP)**	**Δ*G*(SP)**
		[Eh]	[kJ mol^{-1}]	[Eh]	[kJ mol^{-1}]	[Eh]	[kJ mol^{-1}]	[Eh]	[kJ mol^{-1}]
**1** UKS (boat)	0.000	-4656.14759	0.0	-4655.92582	-3.8	-4660.00942	0.0	-4659.78765	-3.8
**1** t		-4656.14060	18.4	-4655.92347	6.2	-4660.00047	23.5	-4659.78333	11.3
**1^{flat}**	1.105	-4656.14161	15.7	-4655.92449	3.5	-4660.00113	21.8	-4659.78401	9.559
**1^{flat}** RKS		-4656.13174	41.6	-4655.90823	46.2	-4659.99219	45.2	-4659.76869	49.8
**2** UKS	0.000	-7141.78056	0.0	-7141.53943	-3.9	-7145.44271	0.1	-7145.20159	-3.9
**2** triplet		-7141.76028	53.2	-7141.52012	50.7	-7145.42062	58.0	-7145.18046	55.5
**2** boat (goes flat)	0.000	-7141.77922	3.5	-7141.53799	3.8	-7145.44102	4.4	-7145.19980	4.7
**3** UKS	0.000	-8685.06579	0.0	not finished		-8692.05050	0.0	n.a.	
**3** triplet		-8685.05649	24.4	-8684.62146		-8692.03229	47.8	-8691.59726	
**3** boat (goes flat)	0.000	-8685.063872	5.0	not finished		-8692.04513	14.1	n.a.	
**Parent Thiele** UKS	0.000	-1232.63701	0.0	-1232.22745	0.0	-1233.92538	0.0	-1233.51582	0.0
**Parent Thiele** t		-1232.61828	49.2	-1232.21226	39.9	-1233.90414	55.8	-1233.49813	46.5
**Parent Thiele** boat (goes flat)	0.000	-1232.63618	2.2	-1232.22662	2.2	-1233.92468	1.9	-1233.51509	1.9
**III** UKS	1.048	-8582.81376	-8.8	-8582.58413	-24.8	-8586.61652	-8.6	-8586.38689	-24.6
**III** RKS		-8582.81042	0	-8582.57469	0	-8586.61326	0	-8586.37753	
**III** t		-8582.81020	9.4	-8582.57828	15.4	-8586.61259	10.3	-8586.38067	16.3
**III** boat (goes flat)	0.00000	-8582.81263	3.0	-8582.57792	16.3	-8586.61222	11.3	-8586.37751	24.6
**IV** UKS	0.000	-3215.10771	0.0	-3214.87778	0.6	-3218.82526	0.0	-3218.59533	0.6
**IV** t		-3215.091681	42.1	-3214.86539	32.5	-3218.80586	50.9	-3218.57957	41.4
**IV** boat (goes flat)	0.000	-3215.106952	2.0	-3214.87854	-2.0	-3218.82096	-2.2	-3218.59768	-6.2

Table S18: Influence of solvation modelling by COSMO-RS.

**Compound**		**ωB97X-V/def2-TZVPP(COSMO-RS)//ωB97X-V/def2-SVP**			
	**COSMO-RS (DCM)**	**Δ*E*(SP, solv)**	**Δ*E*(SP, solution)**	***G*(solv)**	**Δ*G*(solv)**
	[Eh]	[Eh]	[kJ mol^{-1}]	[Eh]	[kJ mol^{-1}]
**1** UKS (boat)	-0.03514642	-4660.04457	-1.4	-4659.82279	-5.2
**1** t	-0.035266999	-4660.03573	23.2	-4659.81860	11.0
**1^{flat}**	-0.035009768	-4660.03614	22.1	-4659.81902	9.918
**1^{flat}** RKS	-0.034609677	-4660.02680	46.7	-4659.80330	51.2
**2** UKS	-0.046897453	-7145.48961	0.0	-7145.24848	-3.9
**2** triplet	-0.048034598	-7145.46866	55.0	-7145.22849	52.5
**2** boat	-0.046816716	-7145.48784	4.6	-7145.24661	4.9

**3** UKS	-0.06397967	-8692.11448	0.1	n.a.	n.a.
**3** triplet	-0.061002708	-8692.09330	55.6	-8691.65826	n.a.
**3** boat	-0.062908234	-8692.10803	16.9	n.a.	n.a.
**Parent Thiele** UKS	-0.036466817	-1233.96185	0.0	-1233.55229	0.0
**Parent Thiele** t	-0.035984197	-1233.94012	57.0	-1233.53411	47.7
**Parent Thiele** boat	-0.036354794	-1233.96100	2.2	-1233.55145	2.2
**III** UKS	-0.051359307	-8586.66788	-11.2	-8586.43825	-27.2
**III** RKS	-0.050365865	-8586.66362	0	-8586.42790	0
**III** t	-0.051334686	-8586.66392	10.4	-8586.43201	16.4
**III** boat	-0.048283899	-8586.66050	19.4	-8586.42579	32.7
**IV** UKS	-0.030072224	-3218.85533	2.0	-3218.62541	2.6
**IV** t	-0.029735469	-3218.83560	51.8	-3218.60931	42.3
**IV** boat	-0.029984426	-3218.85607	-1.9	-3218.62767	-5.9

XYZ Coordinates (ωB97X-V and CASSCF)

56
IV_t

F	-1.27137	4.83702	9.37109
C	0.22134	7.56682	7.49494
F	-0.01459	7.11871	9.80968
C	-0.21754	6.74875	8.54561
F	1.33918	11.14193	9.37259
C	-0.87995	5.54910	8.31481
F	3.16533	11.25113	11.30397
C	0.92108	8.84242	7.74009
C	1.93297	8.91521	8.79089
F	4.48487	6.82494	10.34440
F	2.68574	6.69569	8.38728
C	2.77022	7.82126	9.09518
C	3.71713	7.88659	10.11261
F	2.82775	10.80183	6.92989
C	3.87222	9.04103	10.87221
H	4.61668	9.08920	11.67011
F	2.20936	12.91048	5.43015
C	3.05702	10.13229	10.59184
F	-2.29242	11.50946	5.26865
C	2.10859	10.07354	9.57629
F	-1.70947	9.40608	6.78953
C	0.58651	10.01041	6.92919
C	1.56025	10.95802	6.55050
C	1.24263	12.05793	5.76074
C	-0.05727	12.26376	5.31132
H	-0.30454	13.12857	4.69159
C	-1.03507	11.34252	5.67036
C	-0.72396	10.24007	6.46022
F	0.34597	7.81741	5.14194
C	-1.14667	5.08756	7.01807
F	-0.91079	5.53570	4.70332
C	-0.70784	5.90566	5.96740
F	-2.26391	1.51216	5.14057
C	-0.04544	7.10531	6.19820
F	-4.08979	1.40265	3.20893
C	-1.84623	3.81187	6.77291
C	-2.85796	3.73888	5.72197
F	-5.40977	5.82883	4.16790
F	-3.61093	5.95838	6.12528
C	-3.69525	4.83273	5.41748
C	-4.64201	4.76726	4.39991
F	-3.75275	1.85219	7.58282
C	-4.79690	3.61273	3.64039
H	-5.54123	3.56444	2.84238
F	-3.13431	-0.25637	9.08266
C	-3.98168	2.52156	3.92099
F	1.36723	1.14530	9.24494
C	-3.03339	2.58047	4.93666
F	0.78426	3.24860	7.72398
C	-1.51162	2.64394	7.58391
C	-2.48529	1.69619	7.96243
C	-2.16766	0.59631	8.75224
C	-0.86779	0.39067	9.20187
H	-0.62050	-0.47411	9.82163
C	0.10993	1.31206	8.84302
C	-0.20121	2.41448	8.05311

56
IV

F	-0.45322	4.44856	9.22760
C	0.24268	7.61317	7.50378
F	0.77534	6.68078	9.65586
C	0.22016	6.50078	8.46001
F	0.63242	10.86638	9.63535
C	-0.42029	5.33567	8.23625
F	2.45367	11.34864	11.55417
C	0.88785	8.79253	7.73168
C	1.84890	8.99648	8.85185
F	4.98723	7.64737	10.08526
F	3.15802	7.15261	8.17560
C	2.97951	8.18560	8.99545
C	3.92391	8.44080	9.98578
F	3.03261	10.16840	6.51057
C	3.76233	9.51054	10.86018
H	4.50192	9.70682	11.63997
F	2.73202	12.45134	5.12411
C	2.64281	10.32273	10.72781
F	-1.93467	12.25308	5.80076
C	1.69906	10.07261	9.73211
F	-1.63845	9.96133	7.17792
C	0.70364	10.00444	6.88485
C	1.80917	10.67260	6.34877
C	1.65370	11.85611	5.62812
C	0.39191	12.40450	5.43428
H	0.26969	13.33144	4.86914
C	-0.71399	11.75103	5.96812
C	-0.56132	10.56656	6.68344
F	-0.47165	8.20558	5.28500
C	-1.16749	5.04092	7.00877
F	-1.70014	5.97336	4.85669
C	-1.14499	6.15333	6.05255
F	-1.55760	1.78807	4.87670
C	-0.50453	7.31844	6.27632
F	-3.37950	1.30595	2.95844
C	-1.81264	3.86154	6.78093
C	-2.77404	3.65766	5.66103
F	-5.91305	5.00655	4.42905
F	-4.08322	5.50116	6.33818
C	-3.90484	4.46840	5.51802
C	-4.84956	4.21326	4.52798
F	-3.95734	2.48541	8.00175
C	-4.68814	3.14374	3.65329
H	-5.42798	2.94750	2.87373
F	-3.65663	0.20269	9.38857
C	-3.56848	2.33166	3.78511
F	1.01013	0.40157	8.71258
C	-2.62439	2.58171	4.78051

F	0.71378	2.69313	7.33515
C	-1.62836	2.64969	7.62783
C	-2.73386	1.98141	8.16381
C	-2.57832	0.79802	8.88463
C	-1.31648	0.24984	9.07872
H	-1.19420	-0.67702	9.64400
C	-0.21060	0.90341	8.54496
C	-0.36334	2.08778	7.82949

56

Parent Thiele t

C	2.73917	14.55184	7.87363
C	8.10009	18.38626	11.81633
C	2.84019	16.96316	7.04324
C	6.45510	16.74879	10.78377
C	7.53048	17.03970	11.75949
C	7.99923	15.97494	12.64679
C	3.30889	15.89836	7.93052
C	5.33424	15.97577	11.14374
C	5.50519	16.96218	8.54627
C	1.38550	14.33490	7.52023
C	4.38429	16.18920	8.90625
C	3.52261	13.41571	8.18962
C	2.36733	16.68480	5.73818
C	2.98359	12.13252	8.14012
H	3.61675	11.27105	8.37636
C	6.51723	17.23556	9.46365
C	0.84930	13.04999	7.47932
H	-0.20439	12.91276	7.21509
C	1.64427	11.94051	7.78500
C	1.93375	17.70638	4.89641
H	1.58508	17.46305	3.88738
C	9.45377	18.60334	12.16958
C	2.86165	18.31734	7.45590
C	8.47204	16.25329	13.95187
C	7.31652	19.52230	11.50033
C	7.85543	20.80555	11.54973
H	7.22217	21.66695	11.31350
C	4.32219	15.70240	10.22637
C	7.97773	14.62075	12.23414
C	1.95362	19.03658	5.32833
C	2.42033	19.33433	6.61296
H	2.43551	20.37149	6.96363
C	8.90559	15.23171	14.79365
H	9.25422	15.47504	15.80270
C	8.41902	13.60375	13.07709
H	8.40383	12.56660	12.72642
C	9.19476	20.99771	11.90474
C	9.98986	19.88831	12.21039
H	11.04356	20.02565	12.47453
C	8.88573	13.90151	14.36173
H	1.22124	10.93160	7.75021
H	4.57507	13.55156	8.45750
H	0.74567	15.19522	7.30146
H	2.36819	15.65138	5.37845
H	1.61077	19.83751	4.66576
H	3.21366	18.56236	8.46282
H	3.45510	15.11119	10.53900
H	5.25219	15.59637	12.16766
H	5.58725	17.34156	7.52234
H	7.38432	17.82675	9.15101
H	6.26406	19.38634	11.23252
H	9.61769	22.00665	11.93944
H	10.09371	17.74310	12.38833
H	7.62571	14.37574	11.22723
H	8.47116	17.28671	14.31160
H	9.22854	13.10057	15.02431

56

Parent Thiele

C	2.67256	14.58884	7.91223
C	8.16634	18.34939	11.77796
C	2.88633	16.92097	6.98076
C	6.48404	16.75640	10.81192
C	7.47838	17.02560	11.71548
C	7.95307	16.01701	12.70888
C	3.36065	15.91255	7.97447
C	5.64619	15.55200	10.89159
C	5.19281	17.38616	8.79834
C	1.26862	14.52213	7.87040
C	4.35495	16.18178	8.87805
C	3.40168	13.38958	7.84320
C	2.74831	16.56917	5.62629
C	2.74672	12.15880	7.75990
H	3.33212	11.23536	7.70194
C	6.17272	17.65032	9.68805
C	0.61429	13.29278	7.79595
H	-0.47990	13.26012	7.77727
C	1.35150	12.10616	7.74163
C	2.28977	17.49644	4.69094
H	2.20101	17.20781	3.63857
C	9.57028	18.41623	11.81948
C	2.51970	18.21890	7.37534
C	8.09102	16.36846	14.06345
C	7.43712	19.54856	11.84770
C	8.09198	20.77936	11.93132
H	7.50652	21.70273	11.98985
C	4.66628	15.28785	10.00189
C	8.32022	14.71935	12.31389
C	1.94115	18.78873	5.09463
C	2.05244	19.14511	6.44016
H	1.76689	20.15010	6.76771
C	8.54998	15.44110	14.99850
H	8.63868	15.72945	16.05095
C	8.78789	13.79304	13.24878
H	9.07384	12.78828	12.92091
C	9.48721	20.83215	11.94923
C	10.22451	19.64562	11.89426
H	11.31870	19.67838	11.91270
C	8.89911	14.14907	14.59440
H	0.83788	11.14156	7.67685
H	4.49610	13.42939	7.84077
H	0.68824	15.45048	7.90290
H	3.01153	15.55470	5.30798

H	1.57551	19.51461	4.36123
H	2.59020	18.49561	8.43263
H	4.03407	14.40865	10.15309
H	5.78558	14.88070	11.74335
H	5.05342	18.05744	7.94656
H	6.80499	18.52947	9.53680
H	6.34271	19.50866	11.85049
H	10.00074	21.79677	12.01431
H	10.15076	17.48796	11.78650
H	8.24986	14.44293	11.25651
H	7.82741	17.38272	14.38208
H	9.26509	13.42313	15.32756

56

III t

C	5.12930	1.78307	12.07947
C	6.44070	2.13848	12.41306
C	6.81689	3.48815	12.53867
C	8.20190	3.86336	12.90915
C	8.80250	3.29950	14.12856
C	10.09776	2.73114	14.12730
C	10.68148	2.19014	15.26976
C	8.68021	2.73750	16.52237
C	8.12055	3.27809	15.36713
C	8.95695	4.79509	12.05651
C	9.64945	5.90444	12.59524
C	10.36720	6.79741	11.80401
C	10.40245	6.60121	10.42459
C	9.73161	5.52982	9.83832
C	9.02524	4.64735	10.65216
C	5.83512	4.46967	12.31173
C	4.52358	4.11424	11.97827
C	4.14592	2.76447	11.85865
C	2.75453	2.38771	11.51454
C	2.12832	2.94618	10.30567
C	2.78306	2.95802	9.05266
C	2.20022	3.49363	7.90663
C	0.92001	4.03746	7.98961
C	0.22813	4.05414	9.19939
C	2.01723	1.46090	12.38799
C	1.98019	1.61424	13.79310
C	1.29126	0.73552	14.62577
C	0.60681	-0.33769	14.05888
C	0.61149	-0.53949	12.67987
C	1.31229	0.34970	11.86941
Cl	4.74376	0.11833	11.83347
Cl	7.63507	0.90172	12.57035
Cl	10.99885	2.60349	12.65192
Cl	10.67367	1.52716	17.89164
Cl	6.55905	4.01441	15.52765
Cl	9.56135	6.25487	14.29060
Cl	11.28827	7.70285	9.42794
Cl	8.28824	3.28505	9.87366
Cl	6.21963	6.13455	12.55850
Cl	3.32960	5.35159	11.82250
Cl	4.33790	2.21477	8.86478
Cl	0.18114	4.70272	6.57432
Cl	-0.03330	3.65544	11.82603
Cl	2.73573	2.97810	14.55140
Cl	-0.25748	-1.43457	15.07937
Cl	1.36311	-0.00839	10.17410
H	11.67930	1.75004	15.21944
H	8.12280	2.75569	17.46092
H	10.87670	7.64775	12.26145
H	9.76982	5.36841	8.75923
H	2.73695	3.46825	6.95627
H	-0.76718	4.49736	9.26870
H	1.27718	0.90119	15.70479
H	0.09161	-1.39136	12.23720
C	9.96360	2.19803	16.46435
C	0.83491	3.51777	10.33199

56

III

C	5.11945	1.78191	12.13144
C	6.42543	2.13592	12.46478
C	6.84086	3.49130	12.49038
C	8.19719	3.86174	12.88429
C	8.79391	3.27763	14.10095
C	10.08635	2.70850	14.10784
C	10.66562	2.17612	15.25672
C	8.66026	2.73912	16.49756
C	8.10557	3.26963	15.33506
C	8.97473	4.82005	12.07610
C	9.63985	5.92514	12.65062
C	10.39691	6.81883	11.89750
C	10.50002	6.62275	10.52137
C	9.85783	5.55300	9.90073
C	9.11031	4.67132	10.67798
C	5.87755	4.46920	12.13715
C	4.57031	4.11505	11.80439
C	4.14856	2.76180	11.80285
C	2.77078	2.39101	11.49333
C	2.09275	2.95971	10.31283
C	2.69106	2.93834	9.03350
C	2.05976	3.45343	7.90374
C	0.78867	4.00942	8.03487
C	0.15296	4.05847	9.27434
C	2.04771	1.44737	12.36757
C	2.01206	1.61327	13.77038
C	1.31901	0.74338	14.60915
C	0.63151	-0.33148	14.04901
C	0.63633	-0.54448	12.67167
C	1.34058	0.33727	11.85587
Cl	4.72394	0.10791	11.97871
Cl	7.58980	0.88466	12.71149
Cl	10.98474	2.56465	12.63275
Cl	10.64671	1.53800	17.88438
Cl	6.54015	4.00300	15.47865
Cl	9.46035	6.26971	14.33959
Cl	11.43556	7.72413	9.57067
Cl	8.40187	3.31300	9.86577
Cl	6.27347	6.14432	12.27588
Cl	3.41172	5.36974	11.54768

Cl	4.23544	2.18568	8.79858
Cl	-0.00989	4.65091	6.64096
Cl	0.01996	3.71712	11.92180
Cl	2.78030	2.97691	14.51814
Cl	-0.23734	-1.41784	15.07710
Cl	1.39928	-0.03029	10.16326
H	11.66257	1.73321	15.21421
H	8.09953	2.76528	17.43393
H	10.88424	7.66826	12.38009
H	9.94869	5.39383	8.82447
H	2.55173	3.40507	6.93035
H	-0.83464	4.51178	9.37948
H	1.30535	0.91575	15.68712
H	0.11414	-1.39816	12.23522
C	9.94297	2.19707	16.44833
C	0.80809	3.54024	10.38845

102

3 t

C	5.70435	0.57542	25.68020
C	4.59012	0.93850	24.91308
C	3.45827	1.37685	25.60978
C	4.65533	1.03823	23.43547
C	5.11448	-0.01259	22.57989
C	5.20970	-1.36194	23.01310
C	5.66536	-2.36945	22.17249
C	6.04120	-2.09715	20.85937
H	6.39633	-2.89397	20.20224
C	5.95194	-0.78398	20.40469
C	5.50143	0.23447	21.23494
C	4.24501	2.37967	22.93466
C	3.06264	2.56083	22.20615
C	2.57829	3.84837	21.94419
C	3.26151	4.98897	22.39523
C	4.51106	4.80354	23.00618
C	4.98544	3.51780	23.28941
C	2.58203	6.35414	22.30612
H	1.51061	6.12013	22.19779
C	2.89907	7.18568	21.07282
C	3.96295	6.97452	20.19458
C	4.13294	7.78559	19.06753
C	3.25639	8.82296	18.78712
H	3.39915	9.45197	17.90559
C	2.18967	9.04045	19.65336
C	2.01749	8.22980	20.76874
C	2.64874	7.08598	23.64614
C	2.08110	6.43648	24.74831
C	2.11900	6.98537	26.02681
C	2.68483	8.23734	26.24082
H	2.70525	8.70017	27.22940
C	3.23135	8.90264	25.15147
C	3.22693	8.33528	23.87585
Cl	2.16526	1.17715	21.70298
Cl	6.51580	3.31710	24.06514
Cl	1.08927	4.01945	21.07975
Cl	5.50798	6.16115	23.38662
F	1.50265	5.24362	24.57807
F	1.59104	6.28536	27.03319
F	3.79617	10.09684	25.31336
F	3.82939	9.00748	22.89474
F	0.99094	8.46213	21.59307
F	1.32220	10.02293	19.42300
F	5.16145	7.53962	18.25962
F	4.84338	5.99222	20.38348
F	5.46160	1.47051	20.74368
F	6.30763	-0.48401	19.15854
F	5.72579	-3.61396	22.63833
F	4.83168	-1.69773	24.24427
F	6.81365	0.16491	25.07114
F	2.34981	1.70469	24.94912
C	5.70958	0.70344	27.06107
C	4.59414	1.19681	27.76385
C	3.45799	1.49948	26.99597
C	4.64479	1.50659	29.19219
C	5.41404	0.70803	30.13342
C	5.55763	-0.68780	29.98321
C	6.26744	-1.45124	30.90246
C	6.86292	-0.86098	32.01335
H	7.42016	-1.46328	32.73443
C	6.73398	0.51302	32.18372
C	6.02549	1.28521	31.26769
C	3.92750	2.71098	29.67771
C	2.99433	2.62049	30.72386
C	2.35171	3.76156	31.22594
C	2.59553	5.03147	30.67884
C	3.52593	5.11799	29.63094
C	4.17640	3.98433	29.13205
C	1.85099	6.25534	31.21284
H	1.09378	5.84737	31.90011
C	2.65961	7.20955	32.08846
C	4.01274	7.07765	32.41523
C	4.64031	7.98568	33.27468
C	3.94331	9.03580	33.85166
H	4.44218	9.73810	34.52310
C	2.59040	9.16384	33.55819
C	1.96594	8.25855	32.70783
C	1.00838	6.90926	30.11404
C	-0.09498	6.18079	29.65539
C	-0.97271	6.68569	28.69863
C	-0.77926	7.95309	28.16555
H	-1.45921	8.35774	27.41237
C	0.31172	8.68719	28.60875
C	1.19483	8.17769	29.56070
Cl	2.59713	1.07133	31.37808
Cl	5.35124	4.17338	27.87630
Cl	1.26435	3.57214	32.55889
Cl	3.98471	6.67009	29.03128
F	-0.32578	4.96605	30.15983
F	-2.00112	5.93804	28.30861
F	0.55957	9.89691	28.10272
F	2.22904	8.94791	29.91131
F	0.65858	8.40337	32.46806
F	1.87247	10.14560	34.09791

F	5.93296	7.81315	33.53888
F	4.76143	6.08091	31.94207
F	5.95527	2.59897	31.47489
F	7.29816	1.11387	33.22826
F	6.35949	-2.76505	30.71322
F	4.97979	-1.30787	28.95640
F	6.83675	0.41931	27.71136
F	2.34649	1.93762	27.58433

102

3

C	5.69203	0.96960	25.88863
C	4.84857	1.85460	25.07775
C	4.04679	2.77919	25.88835
C	4.82725	1.84772	23.71697
C	5.44003	0.77856	22.87989
C	5.12850	-0.57175	23.08926
C	5.62024	-1.56397	22.24683
C	6.43743	-1.23946	21.16891
H	6.82449	-2.01991	20.50965
C	6.75301	0.09459	20.94770
C	6.26012	1.09206	21.78945
C	4.13617	2.91460	22.92260
C	2.97398	2.61734	22.20412
C	2.34597	3.59238	21.41371
C	2.85802	4.89596	21.33287
C	4.03788	5.18031	22.04131
C	4.66071	4.21243	22.83517
C	2.10881	5.96138	20.53331
H	1.12656	5.51357	20.31706
C	2.67195	6.29022	19.15539
C	3.87436	5.82039	18.62128
C	4.26595	6.16030	17.32184
C	3.47407	6.96250	16.51495
H	3.78924	7.22193	15.50186
C	2.26585	7.42258	17.02765
C	1.87462	7.07990	18.31639
C	1.76180	7.16268	21.41469
C	0.85833	6.93261	22.45836
C	0.45711	7.94827	23.32318
C	0.94179	9.24021	23.16056
H	0.63028	10.04392	23.83144
C	1.83072	9.48686	22.12254
C	2.23880	8.46658	21.26227
Cl	2.28089	1.04201	22.34543
Cl	6.08713	4.61372	23.71798
Cl	0.92356	3.14829	20.53407
Cl	4.78521	6.72734	21.87889
F	0.36017	5.70452	22.62753
F	-0.40166	7.66721	24.29954
F	2.32057	10.71088	21.93678
F	3.10698	8.77553	20.29903
F	0.70451	7.53243	18.77836
F	1.46705	8.18839	16.28861
F	5.42394	5.68746	16.86754
F	4.68942	5.02123	19.31019
F	6.59511	2.35718	21.54441
F	7.53632	0.44170	19.92967
F	5.29293	-2.83235	22.48212
F	4.34217	-0.91930	24.10477
F	6.57829	0.19682	25.26610
F	3.21334	3.61021	25.26729
C	5.69184	0.96950	27.23623
C	4.84821	1.85445	28.04700
C	4.04659	2.77910	27.23629
C	4.82670	1.84751	29.40778
C	5.43929	0.77825	30.24486
C	5.12769	-0.57200	30.03533
C	5.61929	-1.56436	30.87769
C	6.43643	-1.24003	31.95570
H	6.82340	-2.02056	32.61490
C	6.75209	0.09399	32.17706
C	6.25932	1.09158	31.33539
C	4.13567	2.91446	30.20213
C	2.97344	2.61727	30.92057
C	2.34537	3.59239	31.71082
C	2.85743	4.89597	31.79160
C	4.03738	5.18023	31.08329
C	4.66027	4.21226	30.28955
C	2.10822	5.96145	32.59109
H	1.12573	5.51391	32.80685
C	2.67098	6.28971	33.96928
C	3.87331	5.81976	34.50344
C	4.26464	6.15920	35.80308
C	3.47259	6.96113	36.61008
H	3.78752	7.22023	37.62332
C	2.26446	7.42139	36.09730
C	1.87349	7.07914	34.80837
C	1.76194	7.16315	31.71000
C	0.85884	6.93376	30.66587
C	0.45833	7.94989	29.80126
C	0.94339	9.24159	29.96458
H	0.63243	10.04567	29.29388
C	1.83198	9.48755	31.00306
C	2.23936	8.46681	31.86311
Cl	2.28038	1.04191	30.77940
Cl	6.08676	4.61344	29.40681
Cl	0.92292	3.14836	32.59042
Cl	4.78481	6.72722	31.24571
F	0.36036	5.70589	30.49603
F	-0.40011	7.66950	28.82443
F	2.32221	10.71132	31.18942
F	3.10738	8.77502	32.82673
F	0.70352	7.53190	34.34626
F	1.46554	8.18697	36.83644
F	5.42254	5.68623	36.25744
F	4.68855	5.02092	33.81436
F	6.59436	2.35665	31.58060
F	7.53536	0.44095	33.19519
F	5.29192	-2.83270	30.64224
F	4.34145	-0.91938	29.01969
F	6.57793	0.19663	27.85889
F	3.21291	3.61001	27.85718

56

1 t

C	17.39688	7.41749	17.09603
C	16.03321	7.30678	17.58905
C	15.73450	7.42724	18.96397
C	14.42496	7.36656	19.43278
C	13.35896	7.18781	18.55835
H	12.33301	7.14018	18.93013
C	13.62970	7.07134	17.19815
C	14.93366	7.13209	16.72110
C	17.87325	6.72042	15.90748
C	18.86226	7.28299	15.07351
C	19.35808	6.59683	13.96761
C	18.89479	5.32598	13.64799
H	19.28551	4.79078	12.77964
C	17.92086	4.75117	14.45920
C	17.42097	5.42902	15.56495
C	18.35954	8.28153	17.83162
C	18.16793	9.66900	17.90729
C	19.08555	10.48135	18.58162
C	20.22147	9.92755	19.19077
C	20.41015	8.53965	19.11997
C	19.48792	7.72614	18.45292
C	21.20929	10.79464	19.88838
C	21.95527	11.76495	19.09900
C	22.33473	13.02104	19.61808
C	23.00213	13.96013	18.84047
C	23.31700	13.69456	17.51103
H	23.84099	14.43491	16.90236
C	22.94962	12.46538	16.97599
C	22.28218	11.51820	17.74825
C	21.38680	10.63193	21.32413
C	22.63960	10.80684	21.94952
C	22.80824	10.60218	23.31390
C	21.74063	10.20945	24.11576
H	21.87665	10.04930	25.18767
C	20.49743	10.02622	23.52129
C	20.31947	10.22983	22.15532
Cl	19.71756	6.01514	18.41809
Cl	16.80534	10.37992	17.12076
Cl	21.82038	7.83884	19.82789
Cl	18.80747	12.18141	18.69749
F	16.71291	7.57741	19.85481
F	14.20716	7.47302	20.74101
F	12.63433	6.91461	16.32877
F	15.12883	7.05547	15.40603
F	16.51640	4.81773	16.32750
F	17.46390	3.52995	14.19245
F	20.28058	7.18423	13.20987
F	19.32823	8.50767	15.31191
F	22.02431	13.34370	20.87221
F	23.32468	15.13189	19.38280
F	23.24189	12.17058	15.71197
F	21.98071	10.35009	17.18399
F	23.70393	11.14513	21.22417
F	24.01637	10.77270	23.84544
F	19.45044	9.66112	24.25660
F	19.09920	10.06176	21.64918

56

1

C	18.13683	7.94840	16.60220
C	16.85559	8.70895	16.66283
C	16.66553	9.71989	17.61163
C	15.45115	10.40135	17.68980
C	14.40376	10.08534	16.83280
H	13.45451	10.62267	16.89589
C	14.58070	9.07279	15.89574
C	15.79052	8.38681	15.81711
C	18.00038	6.50012	16.94035
C	18.05390	5.49388	15.97371
C	17.87914	4.15886	16.33509
C	17.63546	3.80551	17.65783
H	17.50095	2.75723	17.93488
C	17.55921	4.80481	18.62115
C	17.73049	6.14156	18.26385
C	19.31770	8.57060	16.40376
C	19.41211	10.03181	16.11460
C	20.30577	10.76465	16.80437
C	21.12675	10.03955	17.81709
C	21.54364	8.68957	17.33650
C	20.65882	7.95862	16.63379
C	21.33357	10.44922	19.08611
C	20.89546	11.77260	19.62175
C	19.75175	11.82756	20.42286
C	19.32624	13.03324	20.97893
C	20.04749	14.20094	20.75871
H	19.71711	15.14691	21.19433
C	21.19939	14.14762	19.98132
C	21.62697	12.94490	19.42084
C	21.87878	9.52525	20.12177
C	23.02541	9.85697	20.84872
C	23.51581	9.00912	21.83932
C	22.86152	7.81802	22.13600
H	23.24538	7.15351	22.91372
C	21.71059	7.48780	21.43072
C	21.21828	8.33210	20.43584
Cl	21.05722	6.41350	15.99856
Cl	18.41666	10.70977	14.88381
Cl	23.14614	8.14417	17.65286
Cl	20.52128	12.44392	16.51639
F	17.64379	10.04693	18.45164
F	15.30691	11.35969	18.60121
F	13.59530	8.74963	15.06198
F	15.94161	7.42488	14.90873
F	17.62147	7.09124	19.19066
F	17.31512	4.49835	19.89286
F	17.95452	3.22034	15.39384
F	18.28590	5.80186	14.70383
F	19.06959	10.71071	20.66817
F	18.22675	13.05075	21.72825
F	21.90896	15.25186	19.75850
F	22.72920	12.92383	18.68199

F	23.67058	10.98967	20.57614
F	24.62017	9.35088	22.49819
F	21.05934	6.35891	21.69827
F	20.11065	7.98419	19.78642

56

1flat

C	2.78737	14.54166	7.85262
C	8.05192	18.39655	11.83757
C	2.81737	16.98229	7.08839
C	6.45313	16.74683	10.78167
C	7.52922	17.03771	11.75749
C	8.02176	15.95576	12.60108
C	3.30990	15.90061	7.93233
C	5.20831	16.24728	11.20502
C	5.63072	16.69139	8.48480
C	1.43250	14.27231	7.56970
C	4.38592	16.19177	8.90816
C	3.61241	13.42518	8.10315
C	2.38999	16.76610	5.76194
C	3.11106	12.12657	8.06583
C	6.64663	16.96403	9.40559
C	0.93991	12.97298	7.53356
C	1.77195	11.88535	7.78172
C	1.95968	17.81285	4.95490
C	9.40672	18.66561	12.12108
C	2.79100	18.31568	7.54891
C	8.44944	16.17161	13.92749
C	7.22713	19.51321	11.58708
C	7.72865	20.81174	11.62495
C	4.19239	15.97465	10.28425
C	8.04793	14.62249	12.14022
C	1.94016	19.12077	5.42994
C	2.35977	19.35889	6.73360
C	8.87976	15.12461	14.73421
C	8.47918	13.57904	12.95521
C	9.06770	21.05269	11.90963
C	9.89948	19.96486	12.15779
C	8.89904	13.81680	14.25885
Cl	5.93520	16.89669	6.79712
Cl	8.18712	17.50072	8.83813
Cl	2.65191	15.43799	10.85172
Cl	4.90381	16.04204	12.89271
F	7.67798	14.33408	10.89376
F	0.58334	15.27663	7.36003
F	4.90873	13.59053	8.36008
F	2.42351	15.54008	5.24282
F	3.16072	18.60446	8.79535
F	2.34664	20.59470	7.22642
F	1.57847	17.54880	3.70743
F	10.25563	17.66112	12.33093
F	5.93087	19.34812	11.32968
F	6.89945	21.82579	11.39156
F	11.19012	20.15626	12.42046
F	8.41625	17.39753	14.44685
F	9.26126	15.38832	15.98167
F	3.94050	11.11272	8.29922
F	-0.35080	12.78132	7.27146
F	8.49212	12.34335	12.46207
H	1.60254	19.94130	4.79282
H	1.38151	10.86559	7.75307
H	9.23668	12.99608	14.89571
H	9.45827	22.07238	11.93872

56

1flat RKS

C	2.63663	14.60360	7.87800
C	8.20150	18.33392	11.81125
C	2.85465	16.89216	6.95675
C	6.50145	16.75613	10.82363
C	7.49696	17.02702	11.72979
C	7.98612	16.04564	12.73413
C	3.34327	15.90932	7.95991
C	5.44387	15.75177	11.06330
C	5.39835	17.18170	8.62504
C	1.24230	14.52925	7.78716
C	4.33997	16.17830	8.86526
C	3.36246	13.41144	7.76696
C	2.67693	16.54129	5.61400
C	2.71533	12.19217	7.58317
C	6.38851	17.44469	9.52063
C	0.59766	13.30721	7.61079
C	1.32873	12.12937	7.50544
C	2.12200	17.43817	4.70428
C	9.59561	18.41057	11.90401
C	2.43945	18.16725	7.35913
C	8.16220	16.39776	14.07677
C	7.47368	19.52512	11.91968
C	8.11866	20.74564	12.10281
C	4.45363	15.48884	10.16774
C	8.40360	14.77088	12.33300
C	1.71569	18.70288	5.11472
C	1.87729	19.05867	6.44884
C	8.71771	15.50236	14.98760
C	8.96632	13.88096	13.24442
C	9.50504	20.81068	12.18250
C	10.23808	19.63384	12.07977
C	9.12625	14.23796	14.57842
Cl	5.56069	17.84110	7.03448
Cl	7.71862	18.41132	8.98388
Cl	3.12440	14.52091	10.70422
Cl	5.28242	15.09092	12.65337
F	8.25012	14.38811	11.06779
F	0.50762	15.63550	7.90161
F	4.69016	13.43023	7.85506
F	3.07278	15.34430	5.18122
F	2.59449	18.54890	8.62448
F	1.49756	20.26134	6.87286
F	1.98878	17.06971	3.43231
F	10.33221	17.30534	11.79204
F	6.14612	19.50422	11.82967
F	7.38880	21.85430	12.19456
F	11.56720	19.66246	12.14317
F	7.76433	17.59453	14.50830

F	8.84935	15.87195	16.25940
F	3.44333	11.08249	7.48886
F	-0.73160	13.28072	7.54931
F	9.34818	12.67855	12.82158
H	1.27983	19.40573	4.40087
H	0.82251	11.17115	7.36738
H	9.56254	13.53628	15.29314
H	10.00958	21.76985	12.32006
56			
**1 t**			
Cl	5.21551	13.48532	7.77818
Cl	7.63123	17.74525	14.75496
Cl	10.58982	17.17100	11.63713
Cl	0.81646	10.41986	7.85323
Cl	8.25524	14.30215	10.55030
Cl	5.64191	19.47928	11.95048
Cl	2.53853	18.64458	9.12146
Cl	3.23435	15.18443	4.94352
Cl	0.24243	15.74884	8.03015
Cl	1.52336	20.19454	4.08914
Cl	9.29863	12.71324	15.56420
Cl	10.06906	22.50414	11.85400
F	3.30111	14.95658	10.61158
F	7.52158	18.00806	9.08877
F	5.22377	15.47300	12.36169
F	5.59905	17.49166	7.33879
C	2.70650	14.56795	7.90569
C	8.13881	18.37497	11.79251
C	2.87198	16.95390	6.99773
C	6.44965	16.76059	10.79462
C	7.51778	17.04123	11.76164
C	7.95751	15.98491	12.68673
C	3.31388	15.90801	7.93354
C	5.34156	15.96902	11.13087
C	5.48034	16.99693	8.57003
C	1.30625	14.38376	7.92076
C	4.37402	16.20251	8.90525
C	3.48638	13.38876	7.88032
C	2.77351	16.71821	5.60862
C	2.92204	12.11693	7.86477
H	3.56169	11.23294	7.82879
C	6.48835	17.26748	9.48727
C	0.71579	13.12160	7.90221
H	-0.37134	13.02465	7.92561
C	1.53295	11.99387	7.87514
C	2.36026	17.69922	4.70926
H	2.30812	17.47713	3.64167
C	9.54052	18.54564	11.76546
C	2.53507	18.25705	7.43067
C	8.07059	16.21105	14.07640
C	7.37062	19.56122	11.83283
C	7.94714	20.82753	11.85130
H	7.31639	21.71735	11.89902
C	4.33362	15.69850	10.21370
C	8.27744	14.68091	12.24315
C	2.03768	18.96541	5.19193

C	2.12307	19.25545	6.55336
H	1.85601	20.24374	6.93263
C	8.48173	15.22058	14.96627
H	8.54542	15.43577	16.03465
C	8.68702	13.67326	13.11099
H	8.94069	12.68462	12.72356
C	9.33720	20.93726	11.82865
C	10.14322	19.80197	11.78659
H	11.23096	19.88876	11.75358
C	8.78723	13.95415	14.47332
56			
**1**			
Cl	5.08898	13.39301	7.63210
Cl	7.27695	17.77453	14.72949
Cl	10.51291	17.01575	11.23065
Cl	0.52868	10.58055	7.57516
Cl	8.42229	14.29650	10.67283
Cl	5.80308	19.61417	12.18312
Cl	2.27412	18.65781	8.96624
Cl	3.66140	15.16012	5.00508
Cl	0.27940	15.87853	8.37221
Cl	1.31323	19.83616	3.82800
Cl	9.53069	13.01787	15.75642
Cl	10.43667	22.30526	12.11666
F	3.70454	14.42075	10.37494
F	7.04971	18.60447	9.38035
F	5.58178	14.93299	12.08834
F	5.17424	18.09279	7.66883
C	2.64790	14.59742	7.94035
C	8.19765	18.35279	11.76784
C	2.89704	16.91644	6.94670
C	6.46441	16.79376	10.84931
C	7.46962	17.04468	11.73568
C	7.92057	16.02660	12.73686
C	3.34007	15.92480	7.97747
C	5.53083	15.67092	10.98169
C	5.23144	17.34588	8.76908
C	1.25018	14.48173	8.04133
C	4.31313	16.20916	8.88928
C	3.35905	13.39302	7.75003
C	2.93938	16.63052	5.57063
C	2.72274	12.16037	7.63414
H	3.30925	11.25329	7.47653
C	6.20817	17.61161	9.65953
C	0.58515	13.25976	7.93188
H	-0.50184	13.21664	8.02276
C	1.33251	12.10552	7.72107
C	2.45837	17.51288	4.60229
H	2.51721	17.25108	3.54414
C	9.59328	18.43274	11.61749
C	2.36286	18.17304	7.30415
C	7.93597	16.29327	14.11746
C	7.52675	19.57209	12.00264
C	8.19987	20.78534	12.11411
H	7.64434	21.70503	12.30698
C	4.55453	15.40525	10.09154

C	8.40547	14.76057	12.34339
C	1.92082	18.72921	5.01056
C	1.87326	19.07212	6.36097
H	1.45849	20.03005	6.68034
C	8.42590	15.38368	15.05557
H	8.41255	15.63155	16.11863
C	8.90241	13.83433	13.25604
H	9.27797	12.86995	12.90882
C	9.58709	20.80466	11.97692
C	10.29482	19.63471	11.72107
H	11.37859	19.65039	11.59121
C	8.91322	14.15863	14.61176

56

1 (CASSCF)

C	18.06428	7.90069	16.59539
C	16.75644	8.63796	16.60689
C	16.46363	9.56117	17.60168
C	15.23908	10.20262	17.63863
C	14.27580	9.93877	16.68955
H	13.32093	10.44245	16.71977
C	14.55391	9.01777	15.69975
C	15.77332	8.37449	15.66156
C	17.93503	6.43449	16.89355
C	17.99118	5.46889	15.89817
C	17.80477	4.13385	16.19902
C	17.55364	3.72858	17.49341
H	17.41040	2.68308	17.72334
C	17.48569	4.68145	18.48791
C	17.66829	6.01785	18.19072
C	19.25335	8.53956	16.45141
C	19.36629	10.00727	16.18806
C	20.22794	10.73629	16.90602
C	21.04334	10.03370	17.92265
C	21.45990	8.67424	17.44388
C	20.59722	7.94140	16.71353
C	21.31998	10.47708	19.16092
C	20.94488	11.83203	19.68956
C	19.82882	11.95963	20.50504
C	19.48919	13.17607	21.06493
C	20.26396	14.29261	20.83364
H	20.00110	15.24451	21.27094
C	21.38278	14.17272	20.03516
C	21.72357	12.96002	19.46998
C	21.92325	9.58464	20.20697
C	23.13146	9.90070	20.81706
C	23.65878	9.10770	21.81430
C	22.99093	7.97779	22.24167
H	23.40364	7.35689	23.02302
C	21.78759	7.65793	21.65246
C	21.25736	8.44871	20.64939
Cl	21.06312	6.45839	15.96564
Cl	18.44910	10.67459	14.88532
Cl	23.10935	8.19803	17.65988
Cl	20.43095	12.42239	16.60656
F	17.35082	9.84621	18.52294
F	15.00210	11.07110	18.59300
F	13.65624	8.74815	14.78111
F	16.00606	7.50673	14.70558
F	17.57384	6.90688	19.15022
F	17.24053	4.32575	19.72712
F	17.87367	3.24526	15.23504
F	18.23618	5.81135	14.65944
F	19.08826	10.90788	20.76066
F	18.42280	13.25818	21.82548
F	22.13530	15.22244	19.79922
F	22.78300	12.89223	18.70570
F	23.79662	10.96387	20.43293
F	24.80894	9.43562	22.35460
F	21.12787	6.59215	22.03980
F	20.11134	8.10103	20.11811

56

1_S1 (CASSCF)

C	2.77960	14.55795	7.86783
C	8.06037	18.38037	11.82246
C	2.83708	16.96844	7.08265
C	6.45626	16.74882	10.78541
C	7.51876	17.03604	11.74940
C	8.00160	15.96966	12.60686
C	3.32049	15.90261	7.94047
C	5.24009	16.16368	11.19086
C	5.59884	16.77588	8.49906
C	1.41986	14.30930	7.60225
C	4.38276	16.19057	8.90449
C	3.59776	13.43244	8.08706
C	2.43744	16.73800	5.75273
C	3.08318	12.14231	8.04005
C	6.61093	17.04816	9.41708
C	0.91364	13.01784	7.55796
C	1.73877	11.92220	7.77638
C	1.99863	17.77183	4.93753
C	9.42023	18.62820	12.08825
C	2.77049	18.29824	7.54264
C	8.40118	16.19943	13.93690
C	7.24285	19.50640	11.60346
C	7.75814	20.79622	11.65086
C	4.22803	15.89139	10.27284
C	8.06758	14.63999	12.14641
C	1.93983	19.07576	5.41226
C	2.32876	19.32770	6.72023
C	8.83934	15.16508	14.75180
C	8.50865	13.61001	12.96852
C	9.10263	21.01553	11.91475
C	9.92715	19.91937	12.13296
C	8.89752	13.86129	14.27663
Cl	5.88825	17.06278	6.82667
Cl	8.11476	17.66076	8.84580
Cl	2.72416	15.27885	10.84409
Cl	4.95063	15.87691	12.86326
F	7.73017	14.34564	10.90056
F	0.58200	15.31943	7.42396
F	4.89228	13.58118	8.32114
F	2.51087	15.51929	5.23947

F	3.10793	18.59319	8.78834
F	2.27351	20.55768	7.20641
F	1.64807	17.50182	3.68957
F	10.25752	17.61757	12.26632
F	5.94829	19.35841	11.36918
F	6.94237	21.81895	11.44873
F	11.21738	20.09490	12.37342
F	8.32831	17.41800	14.45058
F	9.18986	15.43448	15.99991
F	3.89953	11.12009	8.24235
F	-0.37653	12.84155	7.31769
F	8.56334	12.38018	12.48190
H	1.59400	19.88571	4.76951
H	1.33781	10.90892	7.74077
H	9.24286	13.05094	14.91915
H	9.50415	22.02858	11.95070

56

1^{flat} (CASSCF)

C	2.73777	14.53144	7.84751
C	8.10221	18.40651	11.84195
C	2.80954	16.97309	7.03944
C	6.44760	16.74768	10.77795
C	7.53107	17.04110	11.76128
C	8.02900	15.96523	12.65100
C	3.30816	15.89713	7.92867
C	5.15824	16.41470	11.20596
C	5.68093	16.52376	8.48397
C	1.36266	14.32350	7.77284
C	4.39159	16.19071	8.91199
C	3.55157	13.40349	7.83834
C	2.60446	16.75274	5.67961
C	3.01576	12.13216	7.76109
C	6.69206	16.79762	9.40162
C	0.83205	13.05330	7.69254
C	1.65284	11.94377	7.68626
C	2.13611	17.75217	4.85307
C	9.47743	18.61369	11.91661
C	2.52247	18.24380	7.52679
C	8.23414	16.18606	14.01074
C	7.28906	19.53494	11.85057
C	7.82561	20.80599	11.92736
C	4.14715	16.14073	10.28831
C	8.31528	14.69410	12.16425
C	1.85743	19.00864	5.35151
C	2.05643	19.24359	6.69458
C	8.70187	15.18673	14.83775
C	8.78069	13.69441	12.99694
C	9.18863	20.99361	12.00225
C	10.00878	19.88360	11.99647
C	8.97980	13.92986	14.33990
Cl	6.03149	16.53757	6.78898
Cl	8.29086	17.14975	8.83952
Cl	2.54833	15.78874	10.85045
Cl	4.80777	16.40066	12.90097
F	8.15942	14.42529	10.88889
F	0.54249	15.34918	7.79464
F	4.85702	13.53123	7.88924
F	2.87407	15.57782	5.15801
F	2.67815	18.51207	8.80229
F	1.79544	20.42490	7.20216
F	1.96494	17.49909	3.57674
F	10.29700	17.58751	11.89531
F	5.98355	19.40795	11.79954
F	7.01718	21.83923	11.93692
F	11.31160	20.02927	12.05820
F	7.96524	17.36138	14.53179
F	8.87314	15.44029	16.11398
F	3.82479	11.09939	7.75103
F	-0.47070	12.90690	7.63086
F	9.04095	12.51270	12.48992
H	1.49164	19.79063	4.70283
H	1.23583	10.94960	7.62432
H	9.34509	13.14795	14.98896
H	9.60621	21.98756	12.06383

12. References

[1] A. Punzi, Y. Dai, C. N. Dibenedetto, E. Mesto, E. Schingaro, T. Ullrich, M. Striccoli, D. M. Guldi, F. Negri, G. M. Farinola, D. Blasi, "Dark State of the Thiele Hydrocarbon: Efficient Solvatochromic Emission from a Nonpolar Centrosymmetric Singlet Diradicaloid" *J. Am. Chem. Soc.* **2023**, *145*, 20229–20241.

[2] G. M. Sheldrick, "SHELXT – Integrated space-group and crystal-structure determination" *Acta Crystallogr. Sect. Found. Adv.* **2015**, *71*, 3–8.

[3] G. M. Sheldrick, "Crystal structure refinement with SHELXL" *Acta Crystallogr. Sect. C Struct. Chem.* **2015**, *71*, 3–8.

[4], Deposition numbers 2433178 (for **2-H**), 2433179 (for **1-H**), 2433180 (for **2**), 2433181 (for **1**) and 2433182 (for **3**) contain the supplementary crystallographic data for this paper. These data are provided free of charge by the joint Cambridge Crystallographic Data Centre and Fachinformationszentrum Karlsruhe Access Structures service.

[5] A. Belèn Meneses, S. Antonello, M. C. Arévalo, F. Maran, "Double-Layer Correction for Electron-Transfer Kinetics at Glassy Carbon and Mercury Electrodes in N,N-Dimethylformamide" *Electroanalysis* **2006**, *18*, 363–370.

[6] C.-H. Liu, Z. He, C. Ruchlin, Y. Che, K. Somers, D. F. Perepichka, "Thiele's Fluorocarbons: Stable Diradicaloids with Efficient Visible-to-Near-Infrared Fluorescence from a Zwitterionic Excited State" *J. Am. Chem. Soc.* **2023**, *145*, 15702–15707.

[7] A. Kawski, "On the Estimation of Excited-State Dipole Moments from Solvatochromic Shifts of Absorption and Fluorescence Spectra" *Z. Für Naturforschung A* **2002**, *57*, 255–262.

[8] E. Lippert, "Spektroskopische Bestimmung des Dipolmomentes aromatischer Verbindungen im ersten angeregten Singulettzustand" *Z. Für Elektrochem. Berichte Bunsenges. Für Phys. Chem.* **1957**, *61*, 962–975.

[9] N. Mataga, Y. Kaifu, M. Koizumi, "Solvent Effects upon Fluorescence Spectra and the Dipolemoments of Excited Molecules" *Bull. Chem. Soc. Jpn.* **1956**, *29*, 465–470.

[10] F. Neese, "The ORCA program system" *WIREs Comput. Mol. Sci.* **2012**, *2*, 73–78.

[11] J. P. Perdew, K. Burke, M. Ernzerhof, "Generalized Gradient Approximation Made Simple" *Phys. Rev. Lett.* **1996**, *77*, 3865–3868.

[12] E. Caldeweyher, S. Ehlert, A. Hansen, H. Neugebauer, S. Spicher, C. Bannwarth, S. Grimme, "A generally applicable atomic-charge dependent London dispersion correction" *J. Chem. Phys.* **2019**, *150*, 154122.

[13] C. Adamo, V. Barone, "Toward reliable density functional methods without adjustable parameters: The PBE0 model" *J. Chem. Phys.* **1999**, *110*, 6158–6170.

[14] V. N. Staroverov, G. E. Scuseria, J. Tao, J. P. Perdew, "Comparative assessment of a new nonempirical density functional: Molecules and hydrogen-bonded complexes" *J. Chem. Phys.* **2003**, *119*, 12129–12137.

[15] J. Tao, J. P. Perdew, V. N. Staroverov, G. E. Scuseria, "Climbing the Density Functional Ladder: Nonempirical Meta--Generalized Gradient Approximation Designed for Molecules and Solids" *Phys. Rev. Lett.* **2003**, *91*, 146401.

[16] O. A. Vydrov, T. Van Voorhis, "Nonlocal van der Waals density functional: The simpler the better" *J. Chem. Phys.* **2010**, *133*, 244103.

[17] N. Mardirossian, M. Head-Gordon, "ωB97X-V: A 10-parameter, range-separated hybrid, generalized gradient approximation density functional with nonlocal correlation, designed by a survival-of-the-fittest strategy" *Phys. Chem. Chem. Phys.* **2014**, *16*, 9904–9924.

[18] F. Weigend, R. Ahlrichs, "Balanced basis sets of split valence, triple zeta valence and quadruple zeta valence quality for H to Rn: Design and assessment of accuracy" *Phys. Chem. Chem. Phys.* **2005**, *7*, 3297–3305.

[19] F. Neese, F. Wennmohs, A. Hansen, U. Becker, "Efficient, approximate and parallel Hartree-Fock and hybrid DFT calculations. A 'chain-of-spheres' algorithm for the Hartree-Fock exchange" *Chem. Phys.* **2009**, *356*, 98–109.

[20] T. Gerlach, S. Müller, A. G. de Castilla, I. Smirnova, "An open source COSMO-RS implementation and parameterization supporting the efficient implementation of multiple segment descriptors" *Fluid Phase Equilibria* **2022**, *560*, 113472.

[21] Y. Guo, K. Sivalingam, F. Neese, "Approximations of density matrices in N-electron valence state second-order perturbation theory (NEVPT2). I. Revisiting the NEVPT2 construction" *J. Chem. Phys.* **2021**, *154*, 214111.

[22] C. Kollmar, K. Sivalingam, F. Neese, "An alternative choice of the zeroth-order Hamiltonian in CASPT2 theory" *J. Chem. Phys.* **2020**, *152*, 214110.

[23] G. Li Manni, R. K. Carlson, S. Luo, D. Ma, J. Olsen, D. G. Truhlar, L. Gagliardi, "Multiconfiguration Pair-Density Functional Theory" *J. Chem. Theory Comput.* **2014**, *10*, 3669–3680.

[24] B. Helmich-Paris, E. R. Kjellgren, H. J. A. Jensen, "Excited-state methods based on state-averaged long-range CASSCF short-range DFT" *Phys. Chem. Chem. Phys.* **2025**, *27*, 15331–15349.

[25] E. Fromager, J. Toulouse, H. J. Aa. Jensen, "On the universality of the long-/short-range separation in multiconfigurational density-functional theory" *J. Chem. Phys.* **2007**, *126*, 074111.

[26] G. L. S. Rodrigues, M. Scott, M. G. Delcey, "Multiconfigurational Pair-Density Functional Theory Is More Complex than You May Think" *J. Phys. Chem. A* **2023**, *127*, 9381–9388.

[27] G. Knizia, "Intrinsic Atomic Orbitals: An Unbiased Bridge between Quantum Theory and Chemical Concepts" *J. Chem. Theory Comput.* **2013**, *9*, 4834–4843.

[28], Chemcraft - graphical software for visualization of quantum chemistry computations. Version 1.8, build 682. https://www.chemcraftprog.com.

[29] D. Doehnert, J. Koutecky, "Occupation numbers of natural orbitals as a criterion for biradical character. Different kinds of biradicals" *J. Am. Chem. Soc.* **1980**, *102*, 1789–1796.

[30] J. Messelberger, A. Grünwald, P. Pinter, M. M. Hansmann, D. Munz, "Carbene derived diradicaloids – building blocks for singlet fission?" *Chem. Sci.* **2018**, *9*, 6107–6117.

[31] R. Kishi, Y. Murata, M. Saito, K. Morita, M. Abe, M. Nakano, "Theoretical Study on Diradical Characters and Nonlinear Optical Properties of 1,3-Diradical Compounds" *J. Phys. Chem. A* **2014**, *118*, 10837–10848.

[32] K. Yamaguchi, T. Fueno, H. Fukutome, "A molecular-orbital theoretical classification of reactions of singlet ground-state molecules" *Chem. Phys. Lett.* **1973**, *22*, 461–465.

[33] L. Salem, C. Rowland, "The Electronic Properties of Diradicals" *Angew. Chem. Int. Ed. Engl.* **1972**, *11*, 92–111.

[34] D. Herebian, K. E. Wieghardt, F. Neese, "Analysis and Interpretation of Metal-Radical Coupling in a Series of Square Planar Nickel Complexes: Correlated Ab Initio and Density Functional Investigation of [Ni(LISQ)2] (LISQ=3,5-di-tert-butyl-o-diiminobenzosemiquinonate(1-))" *J. Am. Chem. Soc.* **2003**, *125*, 10997–11005.

[35] W. D. Laidig, H. F. Schaefer III, "Large multiconfiguration self-consistent-field wave functions for the ozone molecule" *J. Chem. Phys.* **1981**, *74*, 3411–3414.
